# Common alleles associated with connectivity within and across the brain’s main functional networks

**DOI:** 10.1038/s41467-026-77476-2

**Published:** 2026-09-02

**Authors:** Xavier Caseras, Tom Chambers, Lynsey Hall, Antonio F. Pardiñas, Kevin Murphy, Richard JL Anney

**Affiliations:** 1 Centre for Neuropsychiatric Genetics and Genomics, Division of Psychological Medicine and Clinical Neurosciences, School of Medicine, Cardiff University, Cardiff Wales, UK; 2 Cardiff University Brain Research Imaging Centre (CUBRIC), School of Physics and Astronomy, Cardiff University, Cardiff Wales, UK

**Keywords:** Genetics of the nervous system, Genome-wide association studies, Neural circuits

## Abstract

Previous GWAS of brain’s connectivity have been challenged by the difficulty of best representing the complexity of this phenotype. Here we report the results from a multivariate GWAS approach that allows for a better representation of this complexity, while boosting statistical power. This strategy notably increases the detection of significant signals to 114 independent SNPs, pointing at 315 candidate genes. Our results show a large overlap of the genetic makeup associated with functional connectivity across brain networks but also identify network specific signals, suggesting a potential genetic stratification between cognitive and sensory-motor networks. We also identify a large genetic overlap between functional connectivity and risk for neuropsychiatric disorders, suggesting that regions of the genome important for neuronal communication are enriched for neuropsychiatric risk SNPs. The work presented here expands our understanding of the common allele architecture of brain connectivity, as well as provides novel targets to functional genomics research.

## Abstract (web summary)

This study identified 114 independent SNPs (315 candidate genes) associated with brain’s functional connectivity, showing a large genetic commonality affecting connectivity across different brain networks and with several neuropsychiatric conditions.

## Introduction

Resting state functional connectivity refers to temporally synchronized fluctuations of Blood Oxygenation Level-Dependent (BOLD) signals occurring across distal regions of the brain during rest. These signals have proven to be associated with underlying neural activity^1,2^ and have enabled the identification of distinct and replicable distributed systems of brain areas supporting specific functions, the so-called resting-state networks. Seven of these networks, initially characterised by ref. ^3^—i.e., limbic (LN), visual (VN), ventral attention (VAN), dorsal attention (DAN), default mode (DMN), somatosensory (SMN) and frontoparietal (FPN)—have been consistently replicated. Connectivity in these networks is disrupted in participants with psychiatric disorders^4–6^ and in unaffected participants at elevated genetic risk for mental illness^7–9^, suggesting that changes within network communication could be at the core of these disorders’ neurobiological causes.

Despite twin studies showing that the functional connectivity of the above resting state networks is moderately heritable^10,11^, Genome Wide Association Studies (GWAS) have only captured a very small proportion of this heritability, offering very limited results compared to GWAS of structural brain markers^12^. Improving our understanding of the genetic makeup of these networks’ connectivity is important to realise the potential neurobiological mechanisms of the psychiatric conditions in which the communication within these networks appears disrupted. So far, GWAS studies on the functional connectivity within these networks have attempted to summarise the complexity of their distributed connections into a single metric^13–15^, or have looked at each possible pair-connection within them individually^16,17^. Both these strategies do not properly reflect the network’s internal complexity, limiting the validity of the phenotype under study and likely leading to false negatives in downstream research. Recently developed multivariate GWAS approaches, with the Multivariate Omnibus Statistical Test (MOSTest)^18^ being the latest, can detect common allele influences on related phenotypes by leveraging cross-trait correlations in genetic effects. When these correlations are non-zero, as expected for biologically related phenotypes, this increases statistical power for variant discovery in comparison to only considering one phenotype at a time. Indeed, MOSTest has recently been used to investigate the pleiotropy of common variants influencing brain connectivity and cognitive function or mental conditions^19,20^.

To our knowledge, no multivariate GWAS method has been applied to best understand the common genetic determinants of functional connectivity within each resting-state network. In this study, we applied MOSTest to each of the networks described above^3^. We identified brain structures within each network (nodes) and calculated their correlation with each other node within the network (edges). All edges within a predefined network were entered as the multivariate phenotypes in the GWAS. As our main aim, we investigated the genetic overlap across the 7 networks. We predicted an important genetic overlap across them reflecting common biological processes linked to neural communication; however, we also expected certain degree of uniqueness due to their different functionality. Given prior evidence of functional alterations in these networks in neuropsychiatric conditions, we also investigated the genetic overlap between each network with common variants associated with severe mental disorders. Based on previous research, we predicted the DMN to show the largest genetic overlap with most mental disorders, with sensory networks showing the lowest.

## Results

### MOSTest GWAS results

**Fig. 1:**
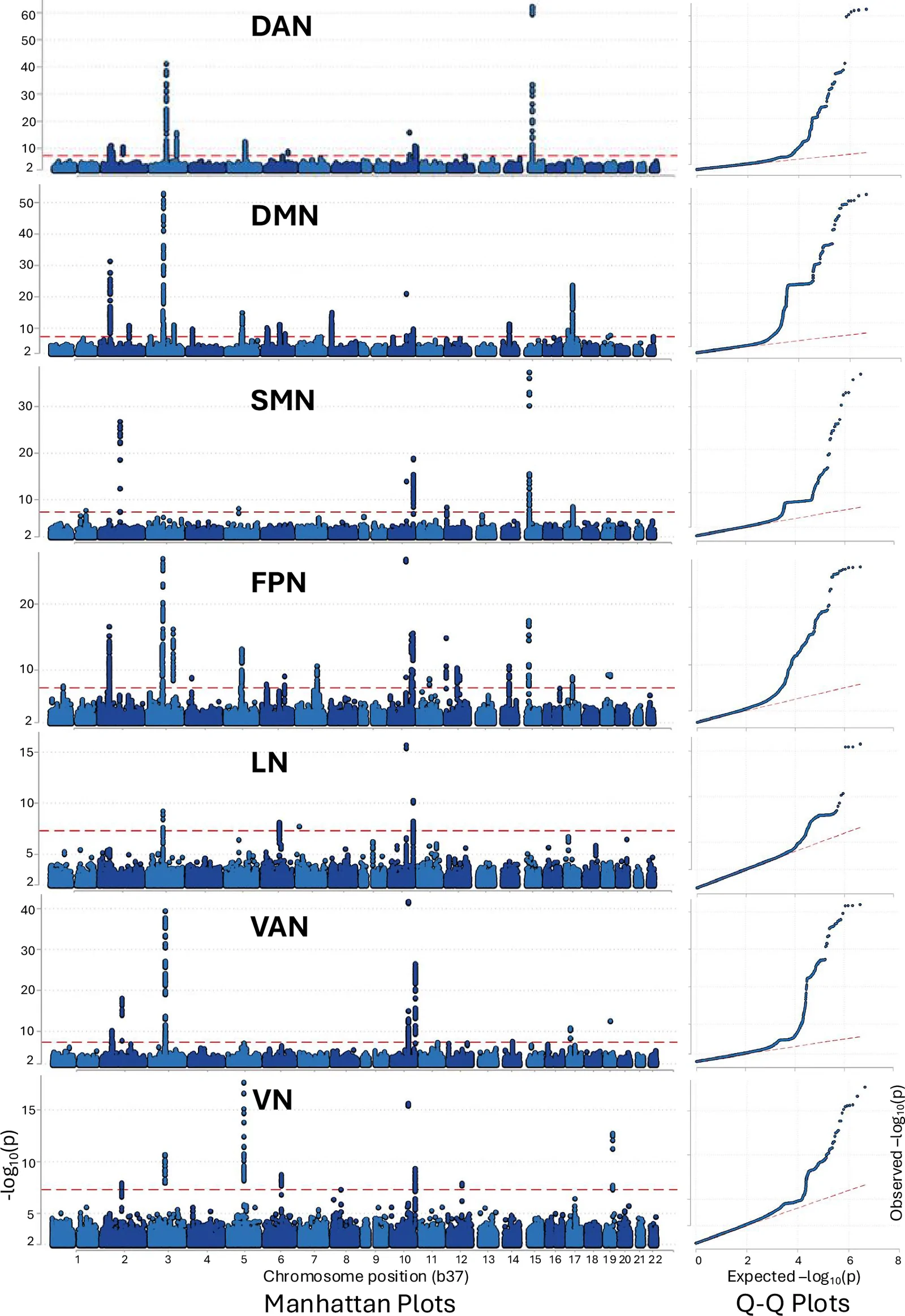
MOSTest results from each rfMRI network. Manhattan plots along with the Q–Q plots from the MOSTest GWAS of each of the 7 Yeo Resting State Networks. The dotted red line indicates the threshold for GWAS significance *p* < 5 × 10^−8^. DAN Dorsal attention network, DMN Default mode network, VAN Ventral attention network, FPN Frontoparietal network, SMN Somatosensory network, LN Limbic network, VN Visual network. Source data for this figure can be obtained from https://www.ebi.ac.uk/gwas/; hyperlinks to the data for each network are provided in the “Data Availability” section.

After applying exclusion criteria, we retained a sample of 30,820 unrelated participants of British and Irish ancestry for our analyses. Demographic characteristics and covariate values for the final sample are presented in Supplementary Table 1. Each network’s multivariate MOSTest GWAS included all potential pairwise connections (edges) between its constituent pre-determined brain regions (nodes). Edges were defined as the partial correlation between the average BOLD timeseries of any given node-pair. The number of nodes and edges, therefore of partial correlations included in each network’s GWAS *MOSTest*, are presented in Supplementary Table 2, ranging from 5 nodes (10 pairwise correlations) for LN to 24 nodes (276 pairwise correlations) for the DMN. The resulting Manhattan plots and their Q–Q plots are presented in Fig. 1. All GWAS analyses identified significant loci at *p* < 5 × 10^−8^, ranging from 153 loci for SMN to 49 for LN (Supplementary Data 1–7). As expected, this multivariate approach substantially increased our ability to detect GWAS significant signals compared to a univariate approach of a summary metric such could be the within network average rfMRI connectivity (Supplementary Fig. 2). After accounting for linkage disequilibrium (LD), the number of significant SNPs was reduced to 114 across all networks, ranging from 34 independent SNPs for DMN to 5 SNPs for LN (see full list for each network in Supplementary Data 8, and locus zooms in Supplementary Figs. 3–9). Of these 114 significant independent SNPs, 93 appear unique to a network, 9 were share by two networks, 3 were shared across three networks and 2 across four networks.

Interestingly, in all networks but SMN, one of the strongest signals came from the same LD region containing gene *EPHA3* (most significant SNP in the region being *rs12636275* for DAN, DMN and VAN; *rs2117137* for FPN and LN; *rs7637670* for VN). The *EPHA3* gene encodes for the ephrin type-A receptor 3 protein, and its expression is enriched in excitatory and inhibitory neurons, microglia and oligodendrocytes across the brain, showing little regional specificity (https://www.proteinatlas.org/ENSG00000044524-EPHA3). Likewise, an LD region in chromosome 10 containing gene *PLCE1* showed highly significant hits across all networks (*rs3891783* for DAN, FPN; *rs10786156* for DMN; and *rs11187838* for LN, SMN, VAN and VN). This gene codes for a phospholipase enzyme that catalyzes the hydrolysis of phosphatidylinositol-4,5-bisphosphate to generate two second messengers: inositol 1,4,5-triphosphate (IP3) and diacylglycerol. These second messengers regulate various processes affecting cell growth, differentiation, and gene expression (https://www.ncbi.nlm.nih.gov/gene/51196). PLCE1 is mostly expressed in inhibitory neurons and oligodendrocyte precursor cells with no regional specificity within the brain. Both these genes have previously been associated with different rfMRI phenotypes related to network function and connectivity^15,17,21,22^ and to the integrity of white matter tracts sustaining brain connectivity^23–25^.

### Results replicability and networks’ heritability

**Fig. 2:**
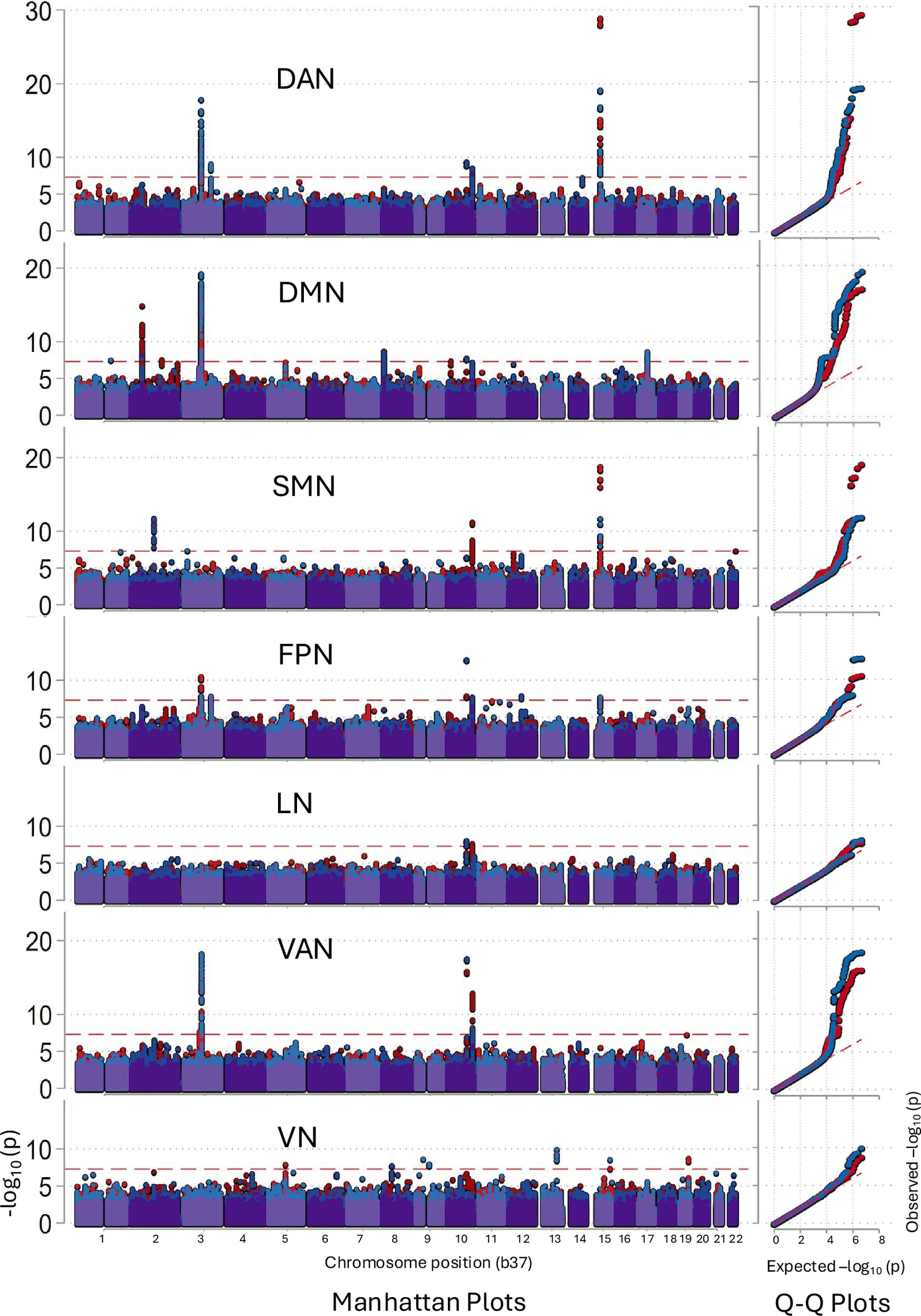
MOSTest results from a random split of the sample. Manhattan plots and Q–Q plots resulting from the MOSTest GWAS from each randomly created half-split of the original sample. Results from each subsample are coloured red and blue, respectively and presented superimposed, purple indicating overlap. The dotted red line indicates the threshold for GWAS significance *p* < 5 × 10^−8^. Visual and Limbic networks show lower coincidence of results between sub-samples and lower depart from the null hypothesis in their Q–Q Plots. DAN Dorsal attention network, DMN Default mode network, VAN Ventral attention network, FPN Frontoparietal network; SMN: Somatosensory network, LN Limbic network, VN Visual network. Source data for this figure can be freely obtained from https://git.cardiff.ac.uk/imagenes/source-data-figure-caseras-et-al-nat-comm-paper/-/tree/332d30d47b67a993aa8e63bd38c10ddd8e04a4ea/.

To explore the replicability of the results above, and due to the lack of a sizeable independent cohort of similar characteristics, we randomly split our sample into two halves and run MOSTest on each subsample (Fig. 2). Halving the sample size caused an expected reduction in statistical power; however, the Manhattan plots for the overall GWAS and the subsamples GWAS overlap substantially for DMN, VAN, DAN & FPN. In all these cases, at least 4 of the most significant hits in the overall GWAS were also significant (*p* < 5 × 10^−8^) in at least one of the two subsamples, with all hits from the overall GWAS being significant at a nominal *p*-value (*p* < 0.05) in the two subsample GWASes, except for four hits (one on each network but only for one of the subsamples) (Supplementary Data 8). None of the subsample analyses for VAN, DAN or FPN produced any significant hits that did not overlap with significant hits in the overall analyses (i.e., false positives). In the case of DMN we only found 2 false positives out of 12 significant hits from the subsample analyses. For SMN, 3 hits replicated at *p* < 5 × 10^−8^ in at least one of the subsamples; although all hits replicated at nominal *p* < 0.05 (Supplementary Data 8). In this case, the subsample analyses produced 3 false positives out of 8 hits in total. The LN produced limited results, only 1 of the 5 hits detected in the overall GWAS was significant in at least one subsample; although, they all were significant at *p* < 0.05. The scarcity of results meant that no false positives were detected. Finally, the VN analyses exhibited poor replicability. Of the nine genome-wide significant hits identified in the combined analysis, only two replicated at genome-wide significance in the subsamples. One of this was not observed in one subsample, likely due to its low minor allele frequency in the combined samples (< 0.5%). The subsample analyses also identified five additional genome-wide significant hits that were not present in the combined GWAS results (i.e., false positives). This combination of limited replication and inconsistent detection across subsamples suggests reduced reliability of the VN GWAS signals.

We used MiXer to calculate the SNP *h*^2^ for each network (Table 1) along with an index of polygenicity or genetic sparsity (i.e., π_1_). The DMN showed the highest heritability and a substantial polygenicity that exceeded that from recent GWAS on schizophrenia^26^ and major depression^27^, both considered extremely polygenic or “omnigenic” phenotypes^28^. DAN, VAN an FPN showed moderate SNP-h^2^ (*h*^2^ > 0.2), this being of similar magnitude to that of schizophrenia, major depression, and structural brain phenotypes^29–31^, but larger than previously reported by GWAS of rfMRI connectivity indices^14,17^. The polygenicity indices from FPN, DAN and VAN were of similar magnitude to the ones showed by putative polygenic phenotypes like schizophrenia (π_1_ = 0.022 (0.002)), major depression (π_1_ = 0.048 (0.004)) and autism (π_1_ = 0.022 (0.001)). SMN and VN presented low heritability (*h*^2^ ~ 0.1), and LN the lowest (*h*^2^ = 0.06). Probably due to its low heritability and scarcity of significant results, LN also showed a very low polygenicity value, suggesting a regional concentration of most causal signals. On the other hand, VN showed a very high polygenicity index, indicating large sparsity of causal signals along the genome.

**Table 1:** Network SNP heritability and polygenicity

	*h*^2^ (SE)	π_1 (SE)_
DMN FPN DAN VAN VN SMN LN	0.40 (0.009) 0.34 (0.002) 0.26 (0.04) 0.23 (0.005) 0.14 (0.002) 0.13 (0.04) 0.06 (0.004)	0.085 (0.005) 0.012 (0.0008) 0.024 (0.005) 0.005 (0.001) 0.415 (0.106) 0.012 (0.005) 0.001 (0.002)

*DMN* Default Mode Network, *FPN* Fronto-Parietal Network, *DAN* Dorsal Attention Network, *VAN* Ventral Attention Network, *VN* Visual Network, *SMN* Somatosensory Network, *LN* Limbic Network, *SE* Standard Error.

Source data for this table can be obtained from https://www.ebi.ac.uk/gwas/; hyperlinks to the data for each network are provided in the Data Availability section.

The LN network was excluded from further analyses due to its very low SNP-based heritability, limited polygenicity and a scarcity of significant GWAS signals. Similarly, the VN network was not analysed further, given its low SNP heritability and poor replicability across subsamples. In both cases, the MiXer model demonstrated poor fit (as indicated by low AIC/BIC values), further supporting their exclusion from downstream analyses.

### Genetic overlap and singularity across networks

**Fig. 3:**
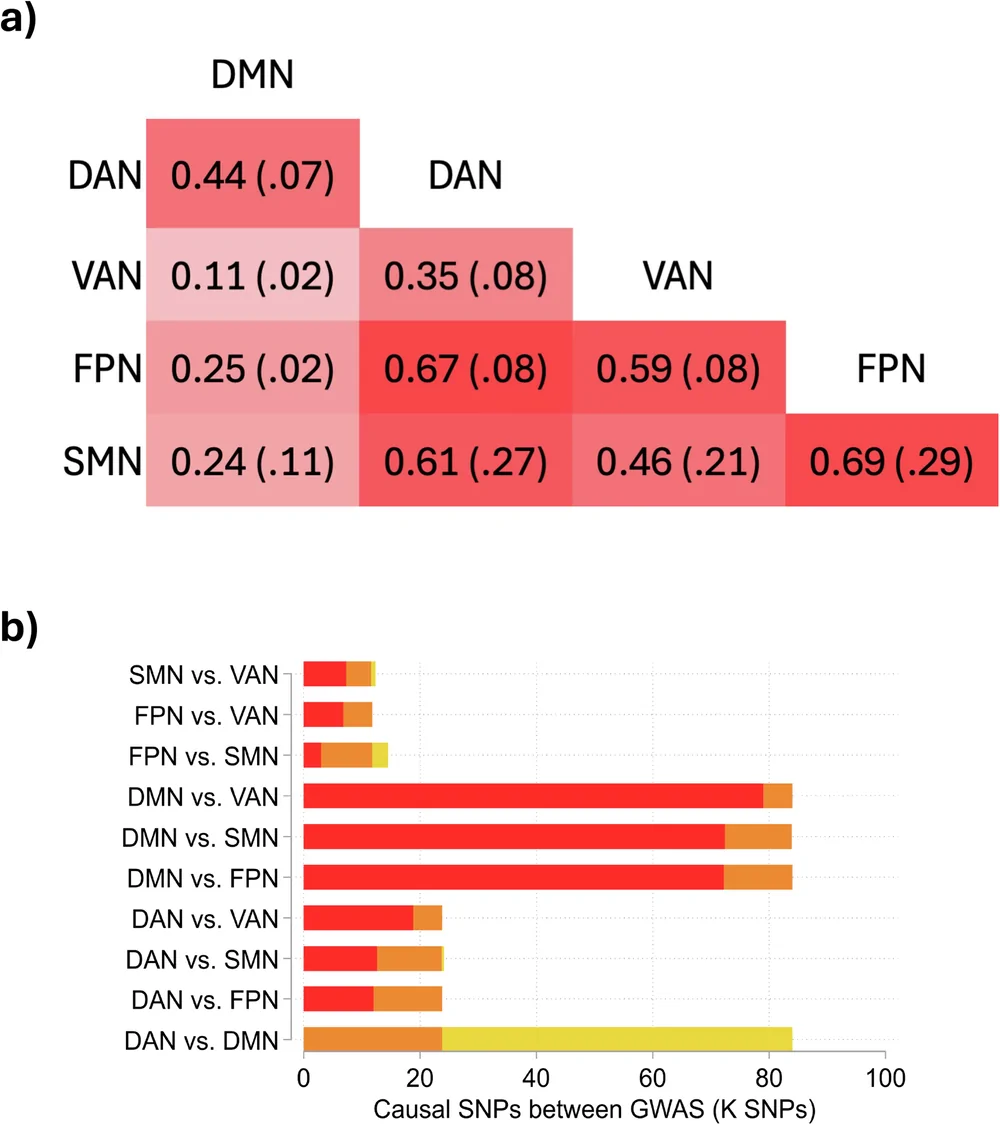
Genetic overlap across the different rfMRI networks. **a** DICE coefficient (standard error), indicating the proportion of shared causal common variants relative to the total number of causal variants contributing to each trait. This metric ranges between 0 (no sharing of causal variants) and 1 (all causal variants for each trait are shared). **b** The estimated number of causal SNPs unique to each network and shared between pairs of networks. Red indicates number of SNPs unique to the first network named in that raw and yellow number of unique SNPs for the second named network, orange indicated the number of SNPs shared across each pair of networks. The number in the *x*-axis indicated thousands (K SNPs). DMN Default mode network, DAN Dorsal attention network, VAN Ventral attention network, FPN Frontoparietal network, SMN Somatosensory network. Source data are provided as a Source Data file.

Genetic overlap across networks was assessed using the DICE index implemented in MiXer. The DICE index quantifies the degree of overlap between two traits by estimating the proportion of shared causal variants, relative to the total number of causal variants contributing to each trait. It ranges from 0 (no overlap) to 1 (complete overlap), and accounts for both the number of shared variants and the polygenicity of each trait. In general, there was a substantial overlap of causal SNPs across the different networks, with the DMN showing the lower DICE index with most other networks investigated, but with DAN (Fig. 3a). However, this needs to be interpreted with caution, DMN was the network with the highest heritability and the largest number of LD-independent significant SNPs; most causal SNPs identified by MiXer for any other network were included within the causal SNP set for DMN (Fig. 3b and Supplementary Table 3). Therefore, the lower DICE value does not really indicate lack of causal SNP overlap between all other networks and DMN, but DMN to capture most SNPs identified by other networks and yet to present many “unique” causal SNPs.

To identify specific genomic regions shared across networks or unique to one of them, we divided the genome in LD-blocks based on work by Berisa & Pickrell^32^, of which 56 contained at least one GWAS significant loci (Supplementary data 9 and Supplementary fig. 10 for locus zoom of each block). We identified 13 (23%) LD-blocks unique to a network (i.e., *p* < 5 × 10^−8^ for that network and *p* > 1 × 10^−5^ for any other network), 7 of which were unique to DMN (representing 16% of the total LD-blocks with significant hits for this network). VAN, DAN and SMN had two unique LD-blocks each (6%, 9%, 18% of the total number of LD-blocks with significant hits, respectively). FPN did not show any unique LD-blocks despite having significant hits in 41 LD-blocks. Four blocks were shared across all networks, and another 8 were shared by all networks but SMN.

### Gene-set annotation

We applied MAGMA to annotate significant GWAS signals into genes for each network. In total, we identified 240 significant genes for DMN, 101 for FPN, 49 for DAN, 45 for VAN and 21 for SMN (Supplementary Data 10–14). We identified 5 genes that were significant in all rfMRI networks: PLCE1, STH, MAPT, KANSL1 and INPP5A. As mentioned earlier, PLCE1 has previously been associated to functional and structural brain connectivity phenotypes, as it has been the case for MAPT and KANSL1^23–25^. However, STH and INPP5A have not previously been associated with brain connectivity, albeit the latter had been associated to cortical morphology (area and thickness)^33,34^. According to GeneCards^35^ and the GTEx platform^36^, STH is a protein coding gene preferentially expressed in brain, involved in positive regulation of mRNA splicing that has been associated with neural conditions such are tauopathy and progressive supranuclear palsy. INPP5A encodes for the protein InsP3 5-phosphatases, involved in the mobilization of intracellular calcium, acting as a second messenger mediating cell responses to various stimulation. This gene has low tissue specificity, but it is highly expressed in brain and has been associated with neurological conditions such are Joubert Syndrome 1 and MORMS.

Further, 11 genes were significant in all networks but SMN: *EPHA3*, *ANKRD32*, *FAM172A* (synonym of *ARB2A*), *FAM175B*, *FHL5*, *KIAA0825*, *METTL10*, *NR2F1*, *UFL1*, *ZIC1* and *ZIC4*. Seven of these had previously been associated to different functional or structural brain connectivity phenotypes^15,17,22–25,37^, whereas four are newly associated to connectivity here: *ANKRD32* is a synonym of *SLF1*, a highly expressed in brain protein-coding gene that enables ubiquitin protein ligase binding activity and that has been associated with From Agnosia and Aicardi Syndrome. *FAM175B* is a synonym of *ABRAXAS2*, a protein-coding gene that enables microtubule binding activity and polyubiquitin modification-dependent protein binding activity. This gene has low tissue specificity, and it is expressed across the brain. *METTL10* is a synonym of *EEF1AKMT2*, which enables protein-lysine N-methyltransferase activity, with no tissue expression specificity but highly expressed in brain. Finally, *NR2F1* encodes for a protein that is a nuclear hormone receptor and transcriptional regulator, it is expressed in brain and across many other tissues.

Another 2 genes were significant in all networks but FPN: *CRHR1*, that had been previously associated to network connectivity phenotypes^17,24^, and *SPPL2C*, newly associated to connectivity in our results. The latter is a protein-coding gene that enables protein homodimerization activity. Previous GWAS studies have linked variation mapped to this gene with Parkinson’s disease^38^, age-of-onset of Alzheimer’s disease^39^, neurodegeneration^40^ and neurodevelopmental conditions^41^.

The Gene Ontology (GO) enrichment analyses showed a significant (FDR *p* < 0.05) enrichment of genes associated with neurogenesis and neural maturation terms for the DMN network (Supplementary Data 15), and with general cell growth and migration for the FPN network. For VAN and SMN, there was enrichment of genes in GO terms associated with immune response and embryonic development, respectively. There were no GO enrichments surviving multiple testing correction for DAN.

### Genetic association with psychiatric traits

**Fig. 4:**
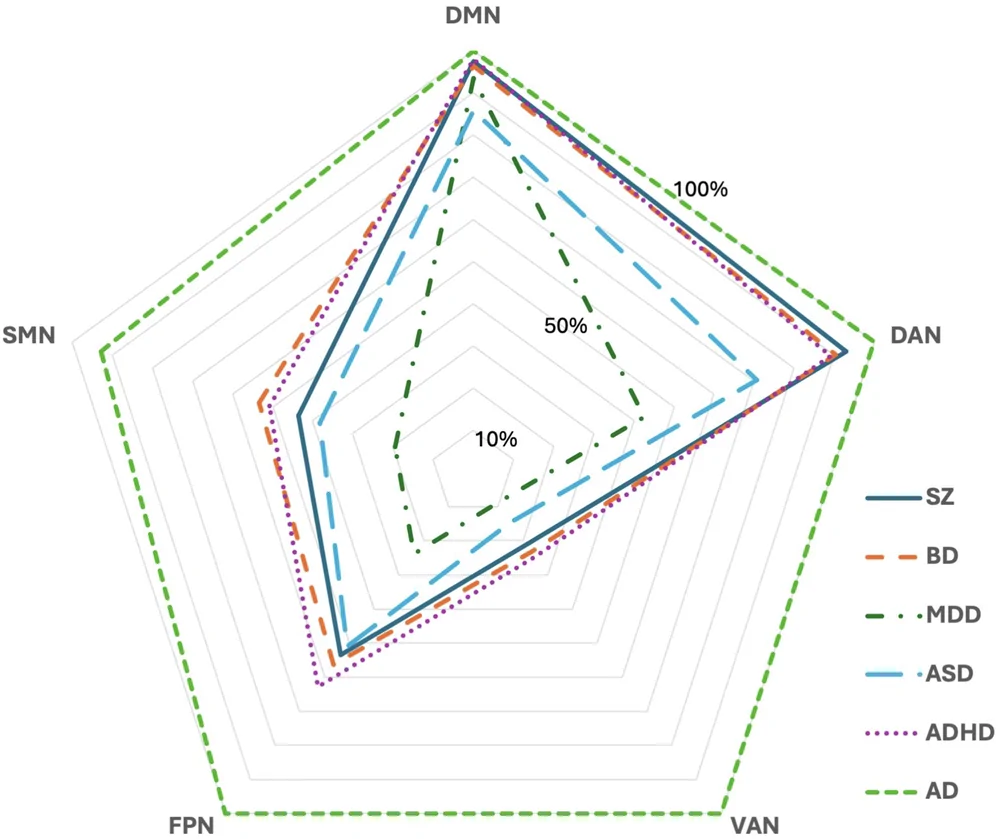
Genetic overlap between rfMRI networks and neuropsychiatric condition. Spider chart indicating proportion of causal SNPs shared between rfMRI networks and each neuropsychiatric condition, relative to the total number of estimated causal SNPs for that neuropsychiatric condition. Values are presented as percentage, in 10% increments. The outer line representing 100% (i.e., all estimated causal SNPs for that neuropsychiatric condition are also estimated to be causal SNPs for that fMRI network) and the centre of the chart 0%. DMN Default mode network, DAN Dorsal attention network, VAN Ventral attention network, FPN Frontoparietal Network, SMN Somatosensory network, SZ Schizophrenia, BD Bipolar disorder, MDD Major depression, ASD Autism spectrum disorder, ADHD Attention deficit hyperactivity disorder, AD Alzheimer’s disease. Source data are provided as a Source Data file.

The same methods to investigate cross-network genetic overlap were deployed to study genetic overlap of each network against mental disorders. The DICE metric from MiXer—proportion of shared causal SNPs relative to total number of causal SNPs for both traits—showed a moderate to high genetic overlap between all networks and all mental disorders, but for Alzheimer’s Disease, where it was low (Table 2). However, this metric is dependent on the polygenicity (i.e., the estimated number of causal SNPs) of the traits included in the analyses. The polygenicity for AD based on ref. ^42^ is low, and much lower than the polygenicity of rfMRI connectivity; therefore, erroneously suggesting lack of genetic overlap. Using alternative metrics, such as proportion of share causal SNPs relative to total causal SNPs for the neuropsychiatric condition (Fig. 4) or total number of causal SNPs unique or shared between pairs of traits (Supplementary Table 4), it can be appreciated that most estimated causal SNPs for AD appeared included within the estimated causal SNPs for each rfMRI network, therefore showing the larger overlap. Overall, there was a substantial overlap of estimated causal SNPs between all rfMRI networks and neuropsychiatric conditions. Nevertheless, slightly different patterns of association were seen across conditions. For SZ, BD and ADHD, most of the estimated causal SNPs for the disorder were shared with DMN and DAN; whereas for MDD, this was only true for DMN (Fig. 4 and Supplementary Table 4).

**Table 2:** DICE coefficient (Standard error) between rfMRI networks and mental disorders

	SZ	BD	MDD	ASD	ADHD	AD
DAN	0.89 (0.06)	0.82 (0.08)	0.56 (0.17)	0.67 (0.25)	0.78 (0.10)	0.003 (0.001)
DMN	0.40 (0.02)	0.36 (0.02)	0.67 (0.07)	0.35 (0.10)	0.35 (0.02)	0.000 (0.000)
FPN	0.69 (0.03)	0.70 (0.06)	0.38 (0.05)	0.66 (0.12)	0.76 (0.03)	0.006 (0.001)
VAN	0.34 (0.07)	0.37 (0.08)	0.16 (0.04)	0.24 (0.13)	0.39 (0.05)	0.02 (0.003)
SMN	0.53 (0.26)	0.62 (0.30)	0.30 (0.16)	0.46 (0.29)	0.58 (0.30)	0.12 (0.27)

*DAN* Dorsal Attention Network, *DMN* Default Mode Network, *FPN* Fronto-Parietal Network, *VAN* Ventral Attention Network, *SMN* Somatosensory Network, *SZ* Schizophrenia, *BD* Bipolar Disorder, *MDD* Major Depression, *ASD* Autistic Spectrum Disorder, *ADHD* Attention Deficit Hyperactivity Disorder, *AD* Alzheimer’s Disease.

Source data are provided as a Source Data file

Of the 56 LD-blocks with at least one GWAS significant hit from any of the rfMRI networks, 49 showed at least one significant hit at *p* < 1 × 10^−5^ from one of the mental disorders GWAS investigated (Supplementary Data 9). SZ showed the highest overlap, with 32 LD-blocks out of the 56 showing a SZ-associated hit at *p* < 1 × 10^−5^ (17 of them at *p* < 5 × 10^−8^). The disorder with lowest overlap was AD, with only 5 LD-blocks.

## Discussion

In this study, we implemented a distinctive approach to investigate genetic common variant influences on brain’s functional connectivity by including each node-pair partial correlation (edge) within a given rfMRI network in a multivariate GWAS (MOSTest). The primary aim of our study was unveiling the genetic makeup of functional connectivity within rfMRI networks and examining their genetic commonality and/or uniqueness. Our multivariate approach improves the representation of complexity within brain networks compared to previous approaches focused on individual nodes or summaries of network properties, as well as facilitates the interpretability of the results by allowing us to analyse each rfMRI network separately based on a predefined parcellation, as opposed to ICA data driven approaches^13–15,17^. It was expected this would increase the statistical power to detect significant genetic associations, and indeed, across all networks, we identified 114 independent genome-wide significant index SNPs. Our results also prove a large genetic overlap across all networks, as predicted, with the SMN showing the lowest common variant coincidence with the other networks, albeit still sharing a large proportion of causal SNPs as defined by MiXer (VN and LN were excluded from these analyses). Via MAGMA analyses, we identified 315 candidate genes associated with functional connectivity within these rfMRI networks, representing a large increase from previous research. Five of those genes were shared across the five networks analysed and 13 shared by four. Another 73 candidate genes were shared across 2–3 networks. Addressing our secondary aim, our results also highlight a substantial genetic overlap between network’s connectivity and neuropsychiatric disorders, identifying relevant regions of the genome for future research.

We excluded the LN and the VN networks from downstream analyses, which limits our ability to draw conclusions about them. This decision was based on three factors: (i) low estimated heritability and polygenicity, (ii) poor reproducibility in internal validation using split-sample analyses and (iii) difficulties fitting the MiXer model to their data, which calls for caution when interpreting any of the results obtained for these two networks. The remaining five networks (i.e., DMN, FPN, DAN, VAN and SMN) showed substantial genetic overlap. MiXer analyses indicated a high proportion of shared causal SNPs across networks, as reflected in the DICE index, with the results for the DMN capturing most causal variants detected for all other networks. We found that 44 out of the 56 independent LD blocks^32^ across the genome with a genome-wide significant hit in at least one network, also showed association at *p* < 1 × 10^−5^ with connectivity in at least one other network. Thirteen of these showed association across four networks, and four across all five. The SMN showed the least overlap: 18% (*n* = 2) of its significant LD blocks were unique to this network, while eight LD blocks were shared by all networks except SMN (*p* < 1 × 10^−5^). To further examine whether these different associations across LD blocks could be simply explained by statistical power, we closely inspected their SNP-level signals via Locus Zoom plots (Supplementary Fig. 10). For those eight blocks where only SMN had no significant associations at *p* < 1 × 10^−5^, there were no clear elevations that could suggest a subthreshold association. Likewise, in the two LD blocks “uniquely” significant for SMN, there were no obvious associations that could have just fallen shorth of *p* < 1 × 10^−5^ for any other network; suggesting that these truly represented a “unique” genetic profile for SMN. This might be suggesting differences in common variant architecture between sensory (i.e., SMN) and executive (i.e., DMN, DAN, VAN & FPN) systems. The interpretation of DMN “unique” LD blocks was less straightforward. Several regions showed subthreshold signals in other networks, implying that the DMN’s apparent distinctiveness may partly reflect its greater statistical power. As the DMN included the largest number of contributing edges in the multivariate GWAS, it was likely more sensitive to detecting polygenic signals that are present, but weaker, in other networks. Future studies with greater statistical power will be essential to clarify the extent of genetic uniqueness across brain networks. This could be achieved through larger sample sizes (e.g., upcoming UK Biobank releases) or by increasing the resolution of network definitions to include more edges, though the latter comes at a substantial computational cost. Overall, our findings point to more shared than unique genetic influences, supporting a common pleiotropic architecture underlying overall brain connectivity.

Our gene-set annotation analyses highlighted 315 unique genes involved in network connectivity. These results supported again the role of *PLCE1* and *EPHA3* in facilitating network connectivity across the brain, along with other genes significant in all or at least in 4 of the 5 networks included in these analyses: *STH, MAPT, KANSL1, INPP5, ANKRD32, FAM172A, FAM175B, FHL5, KIAA0825, METTL10, NR2F1, UFL1, ZIC1, ZIC4, CRHR1* and *SPPL2C*. Most these genes had previously been associated with different brain phenotypes in GWAS studies, some of these accounting for metrics related to functional or anatomical connectivity^15,17,21–25^. Our results bring these together as significant genes sustaining functional connectivity across the brain and identify six new genes not before associated with brain connectivity. Once more, SMN appeared genetically more distinct to the other four networks, with 11 genes shared across all four networks, but not with SMN. This substantiated the idea of a potential genetic stratification between cognitive and sensory-motor networks.

We also report a substantial overlap between common variants associated with connectivity in rfMRI networks and risk for neuropsychiatric conditions. Alzheimer’s disease appeared as the disorder with the least common genetic overlap with connectivity—as defined by DICE—in the different rfMRI networks; however, this might be expected given the low polygenicity observed in the GWAS of Alzheimer’s. In fact, there was near complete overlap of all Alzheimer’s causal variants with causal variants for each of the rfMRI networks. Although no mechanistic interpretation can be drawn from our results, one could speculate that this substantial overlap indicates that risk variants predisposing a person to develop AD, and ultimately the presence of the condition, would also be associated with the brain’s ability to maintain an adequate functional integration, facilitating the communication across distinct areas of the brain.

The pattern of genetic association with neuropsychiatric conditions across rfMRI networks also showed similarities; however, some singularities are also identified. For example, the largest overlap for DMN was shown with MDD, most estimated causal variants for MDD were shared with DMN, whereas for most other disorders (i.e., SZ, BD, ADHD and ASD) DAN appeared more prominent. The pattern of common pleiotropic architecture between MDD and rfMRI networks differed slightly from that of SZ, BD, ADHD and ASD. Notice also that ADHD showed the largest overlap with the FPN network. The shared common genetic overlap between brain connectivity and neuropsychiatric conditions did not appear to concentrate in any chromosome or genomic location, but to be scattered across most LD blocks, including genome-wide significant SNPs for rfMRI networks. Of the 56 LD blocks significant for rfMRI connectivity, 49 also included common allele risk (p1 × 10^−5^) for any of the disorders investigated, SZ showing the largest overlap. Interestingly, of all the LD blocks in which BD showed significant hits, 61% of them also included significant risk alleles for SZ, as could be predicted based on the high genetic correlation reported between these two disorders. However, despite their modest reported genetic correlation, of the 8 blocks hit by ASD, 6 (75%) also included significant risk SNPs for SZ^43^. This result may suggest that pleiotropic effects across neuropsychiatric disorders could concentrate in areas of the genome also associated with brain’s connectivity. Nevertheless, we note that our analyses of causal variant overlap are based on a correlational method (MiXeR). Given the output of MOSTest (unsigned summary statistics) is not sufficient for more complex modelling approaches, at present, we cannot perform a more detailed exploration of the degree to which our results reflect shared biological causality (i.e., true or horizontal pleiotropy) versus phenotype-driven correlations (i.e., vertical pleiotropy).

There are limitations to our approach that should also be noted, such is the fact that networks are defined by different number of nodes and therefore edges included as phenotypes in our multivariate analyses; this, as well as the patterns of associations between underlying nodes in each network and genetic variants, translates into differences in statistical power across networks^44^. The richer results obtained for DMN may be explained by the fact that the multivariate approach for this network includes twice as many edges (i.e., phenotypes) as the second largest network (i.e., VN). This particularly affects our ability to identify unique genetic components to each network, but it should have a lesser impact on recognising shared genetic variation. It is also important to note, though, that network size does not fully explain the different results obtained across networks; for example, VN was the second largest network in terms of edge number, but offered low SNPh^2^ and limited significant SNPs (along with poor replicability) while FPN with just slightly over half the number of edges than VN showed modest SNPh^2^ and three times the number of independent significant SNPs. Therefore, there are still valid between network comparisons that can be drawn from our results. In addition, despite our results show that using the multivariate MOSTest approach improved our ability to best represent each predefined network and increased statistical power, this approach comes with downstream analytical limitations. MOSTest produces unsigned allele weights, which prevents the use of common fine-mapping and LD-score regression-based methods, as well as the generation of polygenic scores. We have overcome this limitation by implementing different approaches that do not rely on a signed SNP weights to identify leading SNPs, and genetic overlap between networks and with neuropsychiatric conditions; however, admittedly, there is a limitation in the depth of post-GWAS analyses that can be performed and the interpretation of the results. Due to the intrinsic complexity of properly capturing this phenotype (i.e., rfMRI connectivity), there is unlikely to be a single approach that will best suit the investigation of its common genetic makeup. Our results add to previous and potential future approaches (e.g., genomic SEM), all carrying their own advantages and limitations, that in concert should bring clarity to the important question of the genetic determinants of brain’s networks connectivity and its pleiotropy with neuropsychiatric conditions. Whereas genomic SEM can be applied to further investigate latent common factors across the different networks using as starting point univariate GWASes from each of them, MOSTest allowed us to identify SNPs with significant effects on any edges within the same network. Our approach assumes that any SNP affecting the communication between a pair of nodes within a network would affect that network’s overall function. Finally, we were also limited in the rfMRI networks that we could investigate. VN and LN did not result in robust enough results in terms of heritability, polygenicity, replicability and model fitting to confidently allow us to include them in our post-GWAS analyses; therefore, what can be concluded about these networks from our results is limited. Future research with improved methods/sample sizes should shed light on their genetic makeup and association with the other brain networks. From our results, VN and LN pose challenges with regards of their potential limited heritability (mostly for LN) and very large polygenicity (mostly for VN), which might require of specific analytical approaches in their case.

In conclusion, we implement an approach to investigate common genetic influences in brain network’s functional connectivity that successfully allowed us to best represent the diversity and complexity of connections within previously predefined and well-described networks, and to identify novel genes at the core of brain’s connectivity. Our results highlight a large overlap on the genetic makeup of these networks, with a potential genetic stratification between executive and sensory-motor networks. We provide evidence that some genes previously reported to be associated with specific brain phenotypes, most likely play an overall role facilitating functional communication across the brain, our work also brings to the forefront new genes associated with brain’s connectivity that grant further follow-up research. Finally, we show a substantial overlap between the common genetic architecture of brain connectivity and that of neuropsychiatric conditions, suggesting that genomic regions implicated in functional connectivity might be enriched for genes with pleiotropic effects across disorders.

## Methods

### Ethics

This project used data collected and retained by the UK Biobank, released to us under project ref 13310. Ethics for data collection, storage and sharing was granted by the North West Multi-Centre Ethics Committee, UK; fully conforming with the Declaration of Helsinki.

### Participants

A subsample of 37,716 UK Biobank (https://www.ukbiobank.ac.uk) participants for whom genetic and resting-state functional MRI (rfMRI) data were available at the onset of this study, and who have consented for their data to be retained and shared by UK Biobank, were included. After completing the quality control and computation steps describe below for the rfMRI and the genetic data, and exclusion of participants with severe mental/neurological conditions and those with missing data in relevant covariates (see Supplementary Methods). the sample was reduced to 30,820 participants.

### RSN definition and connectivity computation

We utilised pre-processed rfMRI data generated by an image-processing pipeline developed and run on behalf of UK Biobank (https://biobank.ctsu.ox.ac.uk/ukb/ukb/docs/brain_mri.pdf; ref. ^45^). Further description can be found in Supplementary Methods.

We registered a 100-node resting-state atlas^46^—for which each node can be allocated to one of the 7 resting state networks—onto each participant’s rfMRI scan (further information on this atlas is provided in the Supplementary Methods). Using AFNI’s 3DNetcorr^47^, we obtained the Fisher Z-transformed functional connectivity between each node-pair within each network by estimating partial correlation between the time series. Partial correlation measures were used instead of full correlation measures (i.e., Pearson correlation) as they: (1) intrinsically remove global confounds from any pair of timeseries^48^ since all other timeseries are regressed from the pair before the correlation is calculated, as global signal regression would do; (2) emphasise direct (rather than indirect) connections between node pairs^49^; (3) reflect conditional dependencies and improve sensitivity to individual pairwise node differences; (4) for big data, scale more meaningfully with large numbers of regions and participant datasets; (5) avoid genomic inflation due to the expected strong correlations across the phenotypes (node-pairs) included in the MOSTest. Outlier node-node partial correlations for each individual pair (defined as 5× median absolute deviation) were excluded from further analyses. Most node-pairs did not have outlier values across the population (70% of node-pairs), and the maximum number of data-points (partial correlation values) removed from any node-pair was 32. The remaining correlations were regressed on age^2^ (2nd degree orthogonal polynomial), sex, age^2^ × sex, mean resting-state functional MRI head motion averaged across space and time points (log-transformed), imaging centre attended, date attended imaging centre, X-, Y- and Z-head position in the scanner, starting table-Z position and the first 10 principal components from genetic population stratification. The resulting residuals were normalised using a rank-based inverse normal transformation^50^ and entered into MOSTest.

### Genetic data quality control

Genotyping, imputation, and quality control for UK Biobank participants have been described previously (https://biobank.ctsu.ox.ac.uk/crystal/crystal/docs/genotyping_qc.pdf)^51^. We applied additional genetic quality control including restricting the dataset to variants with a minor allele count of at least five, removing SNPs with significant deviation from Hardy–Weinberg Equilibrium (*p* < 1 × 10^−20^, due to the large sample size we applied this stringent *p*-value to retain sensitivity to gross departures consistent with technical artefact while reducing unnecessary variant loss driven by power), and excluding SNPs and individuals with more than 2% missingness. As result, 4,341,165 SNPs were retained and examined in each MOSTest GWAS analysis. Individuals with excessive homozygosity or heterozygosity—defined as more than four standard deviations from the sample mean—were removed, as were individuals showing excessive relatedness to non-family members, also defined as greater than four standard deviations from the kinship estimate mean. To minimise confounding due to population stratification, genetic ancestry was assessed using principal components (PCs) derived from genotype data. Individuals were excluded if they deviated by more than 4 standard deviations from the 1000 Genomes Phase 3 GBR reference population mean on the first three PCs. This approach retained participants of White British/Irish ancestries, which make up most of the UK Biobank cohort. Only a small number of participants with rfMRI data were excluded, and due to their considerable ancestral diversity, our inclusion criteria is conservative and aimed to reduce confounding and increase statistical power, as done by previous UK Biobank neuroimaging studies. We also aimed to ensure compatibility with procedures for GWAS downstream analyses, which require accurate estimates of LD that often rely on single-population reference panels^52^.

### Multivariate GWAS approach

The Multivariate Omnibus Statistical test (MOSTest) was used as GWAS approach for each rfMRI network. MOSTest is a statistical method designed to boost the detection of genetic associations by jointly analysing multiple traits or phenotypes that may be correlated^53^. Rather than running separate univariate GWAS for each trait (which can miss shared signals), MOSTest combines them in a multivariate framework to identify genetic variants influencing any combination of the traits. For each network, univariate GWAS was performed on each normalised node-to-node partial correlation. Following combination using MOSTest a single joint test statistic that captures the overall genetic signal across traits was produced. The resulting z-score reflects the strength of association but does not provide a direction of effect (i.e., the statistic is unsigned). The code required to run MOSTest is available from the original authors at https://github.com/precimed/mostest.

### GWAS results replicability

To assess the replicability of GWAS findings and mitigate the absence of an independent external dataset with equivalent imaging and genetic data, we implemented an internal replication approach. The final sample of 30,820 participants was randomly split into two equally sized subsamples. Independent MOSTest GWAS analyses were conducted for each subsample using the same pipeline as applied to the full dataset. Concordance of significant signals across subsamples and with the full-sample analysis was then evaluated to identify replicated associations and estimate the rate of false positives. After this replication step, the full dataset was used for all downstream analyses, including gene mapping, pathway enrichment, and cross-trait comparisons.

### Post-GWAS analyses

Given MOSTest does not return effect size metric comparable to those of other GWAS methods (i.e., betas and standard errors), we conducted a simplified post-GWAS analysis to explore the genetic signals identified. As a basic signal refinement step, we applied LD pruning to identify independent lead SNPs using PLINK. Starting with the most significant genome-wide SNP (*p* < 5 × 10⁻⁸), we iteratively removed other significant SNPs within 5000 kilobases and in LD (*r*. > 0.2) with the lead SNP. This process continued until no additional genome-wide significant SNPs remained. For each resulting LD-independent signal, we identified overlapping genes based on genomic coordinates as potential candidates for further investigation. Due to the MOSTest output lacking effect size and direction, probabilistic fine-mapping methods such as FINEMAP could not be applied. Our LD pruning approach, therefore, served as a pragmatic alternative to identify approximately independent genomic loci and associated candidate genes.

We used MiXer^54^ to quantify each network’s SNP heritability, polygenicity (i.e., the proportion of SNPs contributing to causal effects), and the degree of causal variant overlap across rfMRI networks. MiXeR does not require effect directions to estimate whether variants are shared, as previously noted^55–57^, but the interpretation is limited as bivariate MiXeR with unsigned data estimates only reveal shared genetic involvement, not shared biological direction. MiXer was also applied to quantify the shared polygenic architecture between each rfMRI network and between these and several psychiatric and neurodevelopmental disorders, including schizophrenia^26^, bipolar disorder^58^, major depressive disorder^27^, autism spectrum disorder^59^, attention-deficit/hyperactivity disorder^60^ and Alzheimer’s disease^42^. Because MOSTest produces unsigned omnibus association statistics, bivariate MiXeR analyses in this study quantify shared genetic involvement between traits but do not permit inference regarding concordant or discordant effect directions. Overlap estimates should therefore be interpreted as reflecting shared causal variant participation rather than directional pleiotropy.

To further investigate genomic convergence, we partitioned the genome into LD blocks based on the LDetect algorithm, using the European reference panel from the 1000 Genomes Project^32^. We then identified LD blocks that contained genome-wide significant associations (*p* < 5 × 10⁻⁸) for RSNs. For LD blocks that contained at least one genome-wide significant association, we subsequently assessed whether or any other RSN or psychiatric disorder, the GWAS included in our analysis showed an association signal at *p* > 1 × 10⁻⁵.

For each set of summary statistics derived from RSN MOSTest, we performed gene-based and gene-set association analyses using the Multi-marker Analysis of GenoMic Annotation software (MAGMA)^61^ to further interpret the results of our GWAS. First, SNP-level *p*-values were mapped to genes using a window of 35 kilobases 5′ of the gene start and 10 kilobases 3′ of the gene end coordinates. MAGMA uses LD between variants to calculate a gene-level association statistics reflecting the joint effect of all SNPs within each gene. LD was calculated from the 1000 genomes reference genotypes limited to populations of European ancestry. Gene-level *p*-value, specifically the Multi Model *P*-values are reported in Supplementary Data 10–14. We used these gene-based statistics to test for enrichment in predefined biological pathways and functional categories. Specifically, we assessed whether gene sets from the GO annotations in the Molecular Signatures Database (release 2024-04-24), which include biological processes, cellular components, and molecular function, were significantly associated with our trait of interest. This allowed us to identify GO terms that may be enriched for polygenic signals across the genome. We restricted analyses to gene sets containing at least 10 genes represented in the MOSTest summary statistics, representing a total of 7210 terms across the three ontology categories. Gene set *p*-values were corrected using the False Discovery Rate (FDR) method within each ontology node^62^.

### Reporting summary

Further information on research design is available in the Nature Portfolio Reporting Summary linked to this article.

## Supplementary information

Supplementary Information

Description of Additional Supplementary Files

Supplementary Data 1

Supplementary Data 2

Supplementary Data 3

Supplementary Data 4

Supplementary Data 5

Supplementary Data 6

Supplementary Data 7

Supplementary Data 8

Supplementary Data 9

Supplementary Data 10

Supplementary Data 11

Supplementary Data 12

Supplementary Data 13

Supplementary Data 14

Supplementary Data 15

Reporting Summary

Transparent Peer Review file

## Source data

Source Data

## Data Availability

Data used in this project is publicly available via the UK Biobank: https://www.ukbiobank.ac.uk. A data showcase for this cohort can be found at: https://biobank.ndph.ox.ac.uk/showcase/. Genetic Variants within UK Biobank were harmonised to the hg19 reference genome co-ordinates and standardised to the Haplotype Reference Consortium (HRC) r1.1 allele and marker nomenclature: ftp://ngs.sanger.ac.uk/production/hrc/HRC.r1-1/HRC.r1-1.GRCh37.wgs.mac5.sites.vcf.gz. The 1000 Genomes Project Phase 3 genotypes were used as linkage disequilibrium references as part of the genetic QC protocol: http://ftp.1000genomes.ebi.ac.uk/vol1/ftp/release/20130502/. The summary statistics from the genome-wide association analyses performed in this study have been deposited in the GWAS Catalog (https://www.ebi.ac.uk/gwas/): DAN—https://ftp.ebi.ac.uk/pub/databases/gwas/summary_statistics/GCST90809001-GCST90810000/GCST90809456. DMN—https://ftp.ebi.ac.uk/pub/databases/gwas/summary_statistics/GCST90809001-GCST90810000/GCST90809457 FPN—https://ftp.ebi.ac.uk/pub/databases/gwas/summary_statistics/GCST90809001-GCST90810000/GCST90809458. LN—https://ftp.ebi.ac.uk/pub/databases/gwas/summary_statistics/GCST90809001-GCST90810000/GCST90809459 SMN—https://ftp.ebi.ac.uk/pub/databases/gwas/summary_statistics/GCST90809001-GCST90810000/GCST90809460. VAN—https://ftp.ebi.ac.uk/pub/databases/gwas/summary_statistics/GCST90809001-GCST90810000/GCST90809461. VN—https://ftp.ebi.ac.uk/pub/databases/gwas/summary_statistics/GCST90809001-GCST90810000/GCST90809462. Summary statistics from Neuropsychiatric conditions but AD were obtained from the Psychiatric Genomics Consortium: https://pgc.unc.edu/for-researchers/download-results/ Schizophrenia filename: daner_PGC_SCZ_w3_90_0418b_ukbbdedupe.trios * Bipolar filename: daner_bip_pgc3_nm_noukbiobank * ADHD filename: ADHD2022_iPSYCH_deCODE_PGC.meta https://figshare.com/articles/dataset/adhd2022/22564390. ASD filename: iPSYCH-PGC_ASD_Nov2017 https://figshare.com/articles/dataset/asd2019/14671989. MDD filename: daner_pgc_mdd_meta_w2_no23andMe_rmUKBB * * For the samples Schizophrenia, Bipolar and MDD - requests were made to the PGC Data Access committee to provide SUMSTATS, which excluded participants who were identified as part of the UKBB to avoid sample overlap. Summary statistics used for our analyses did not include 23andMe samples. AD summary statistics data was obtained upon request to the original authors^42^. Source data are provided with this paper in the Supplementary Data. Source data are provided with this paper.

## Code availability

Scripts created to produce the connectivity phenotypes included in the MOSTest analyses can be accessed in: https://git.cardiff.ac.uk/imagenes/rfmri_networks_phenotype.git^63^. Custom code developed for this study is available under the MIT No Attribution License.
