# Supplementary material for: Common alleles associated with connectivity within and across the brain’s main functional networks
Source: Nat Commun. 2026 Sep 2;17:10427. doi: 10.1038/s41467-026-77476-2 (PMC13631237; doi:10.1038/s41467-026-77476-2)
Supplement: Supplementary file 1 [file 41467_2026_77476_MOESM18_ESM.pdf]

nature portfolio

Corresponding author(s): Xavier Caseras

Last updated by author(s): Apr 17, 2026

Reporting Summary

Nature Portfolio wishes to improve the reproducibility of the work that we publish. This form provides structure for consistency and transparency in reporting. For further information on Nature Portfolio policies, see our Editorial Policies and the Editorial Policy Checklist.

Statistics

For all statistical analyses, confirm that the following items are present in the figure legend, table legend, main text, or Methods section.

n/a	Confirmed	
☐	☒	The exact sample size (*n*) for each experimental group/condition, given as a discrete number and unit of measurement
☐	☒	A statement on whether measurements were taken from distinct samples or whether the same sample was measured repeatedly
☐	☒	The statistical test(s) used AND whether they are one- or two-sided<br>*Only common tests should be described solely by name; describe more complex techniques in the Methods section.*
☐	☒	A description of all covariates tested
☐	☒	A description of any assumptions or corrections, such as tests of normality and adjustment for multiple comparisons
☐	☒	A full description of the statistical parameters including central tendency (e.g. means) or other basic estimates (e.g. regression coefficient) AND variation (e.g. standard deviation) or associated estimates of uncertainty (e.g. confidence intervals)
☐	☒	For null hypothesis testing, the test statistic (e.g. *F*, *t*, *r*) with confidence intervals, effect sizes, degrees of freedom and *P* value noted<br>*Give P values as exact values whenever suitable.*
☒	☐	For Bayesian analysis, information on the choice of priors and Markov chain Monte Carlo settings
☒	☐	For hierarchical and complex designs, identification of the appropriate level for tests and full reporting of outcomes
☐	☒	Estimates of effect sizes (e.g. Cohen's *d*, Pearson's *r*), indicating how they were calculated

Our web collection on statistics for biologists contains articles on many of the points above.

Software and code

Policy information about availability of computer code

Data collection	data use in this manuscript is publicaly acccessible via UK Biobank
Data analysis	previously published methods/alogarithms were used in this manuscript

For manuscripts utilizing custom algorithms or software that are central to the research but not yet described in published literature, software must be made available to editors and reviewers. We strongly encourage code deposition in a community repository (e.g. GitHub). See the Nature Portfolio guidelines for submitting code & software for further information.

Data

Policy information about availability of data

All manuscripts must include a data availability statement. This statement should provide the following information, where applicable:

- Accession codes, unique identifiers, or web links for publicly available datasets
- A description of any restrictions on data availability
- For clinical datasets or third party data, please ensure that the statement adheres to our policy

we include a data availability statement: Data used in this project is publicly available via the UK Biobank: https://www.ukbiobank.ac.uk/

Research involving human participants, their data, or biological material

Policy information about studies with human participants or human data. See also policy information about sex, gender (identity/presentation), and sexual orientation and race, ethnicity and racism.

Reporting on sex and gender	We report on the sex distribution of our sample, but do not conduct separate analyses per sex since we consider this variable not to be relevant for the hypotheses/analyses conducted. We do not have access to gender identification variables, we do not consider this variable to be of relevance for our analyses either. For methodological reasons and data accessibility we limit our analyses to participants of British and Irish ancestry.
Reporting on race, ethnicity, or other socially relevant groupings	Due to methodological reason and lack of diversity in the dataset used, we limited our analyses to participants of British and Irish ancestry. This is clearly stated within the Methods section.
Population characteristics	We used age as covariate in our analyses, as well as the 10 first principal components of population stratification as per standard methods within GWAS analyses.
Recruitment	We used data from an existing large cohort o- UK Biobank. We provide reference to the original research where recruitment methods are explained.
Ethics oversight	As stated in the manuscript, UK Biobank received ethical approval and data was released to us after approval: North West Multi-Centre Ethics Committee and data was released to us under project ref 13310

Note that full information on the approval of the study protocol must also be provided in the manuscript.

Field-specific reporting

Please select the one below that is the best fit for your research. If you are not sure, read the appropriate sections before making your selection.

☒ Life sciences ☐ Behavioural & social sciences ☐ Ecological, evolutionary & environmental sciences

For a reference copy of the document with all sections, see nature.com/documents/nr-reporting-summary-flat.pdf

Life sciences study design

All studies must disclose on these points even when the disclosure is negative.

Sample size	all available participants with MRI imaging and genetic data that passed our QC protocol and complied with our inclusion/exclusion criteria were included in the analyses: n= 30,820
Data exclusions	We excluded participants that were related to each other, of other than British & Irish ancestry, with a recorded diagnosis of severe mental disorder, or from whom data was considered not of sufficient quality
Replication	We replicated our main results via a random split of our original sample in two equal sized subsamples.
Randomization	NA
Blinding	NA

Reporting for specific materials, systems and methods

We require information from authors about some types of materials, experimental systems and methods used in many studies. Here, indicate whether each material, system or method listed is relevant to your study. If you are not sure if a list item applies to your research, read the appropriate section before selecting a response.

Materials & experimental systems

n/a	Involved in the study
☒	☐ Antibodies
☒	☐ Eukaryotic cell lines
☒	☐ Palaeontology and archaeology
☒	☐ Animals and other organisms
☒	☐ Clinical data
☒	☐ Dual use research of concern
☒	☐ Plants

Methods

n/a	Involved in the study
☒	☐ ChIP-seq
☒	☐ Flow cytometry
☐	☒ MRI-based neuroimaging

Plants

Seed stocks	NA
Novel plant genotypes	NA
Authentication	NA

Magnetic resonance imaging

Experimental design

Design type	resting fMRI data was used
Design specifications	resting-state functional MRI data was obtained via three equivalent Siemens Skyra 3T scanners with standard Siemens 32-channel receive head coils during a 6-minute scan in the absence of any explicit task/stimulation, using a voxel size of 2.4mm isotropic, field-of-view: 88x88x64 matrix, repetition time of 0.735s , echo-time of 39ms, GE-EPI with x8 multislice acceleration and flip angle 52o
Behavioral performance measures	NA

Acquisition

Imaging type(s)	functional (resting)
Field strength	3T
Sequence & imaging parameters	repetition time of 0.735s , echo-time of 39ms, GE-EPI with x8 multislice acceleration and flip angle 52o
Area of acquisition	whole brain - field-of-view: 88x88x64 matrix

Diffusion MRI ☐ Used ☒ Not used

Preprocessing

Preprocessing software	Pre-processing included motion correction using MCFLIRT (Jenkinson et al. 2002), grand-mean intensity normalisation, high-pass temporal filtering, EPI unwarping, GDC unwarping and structural artefact removal by ICA+FIX processing
Normalization	grand-mean intensity normalisation
Normalization template	MNI to individual space
Noise and artifact removal	artefact removal by ICA+FIX processing
Volume censoring	not applied

Statistical modeling & inference

Model type and settings	We applied a multivariate GWAS approach previously published: MOSTest
Effect(s) tested	NA

Specify type of analysis: ☒ Whole brain ☐ ROI-based ☐ Both

Statistic type for inference (See Eklund et al. 2016)	partial correlations between each node pair within brain networks were calculated and entered in the MOSTest GWAS
Correction	GWAs corrected p value is used

Models & analysis

n/a	Involved in the study
☐	☒ Functional and/or effective connectivity
☒	☐ Graph analysis
☒	☐ Multivariate modeling or predictive analysis

Functional and/or effective connectivity: partial correlations between average time series within ROIs
