# Supplementary material for: Common alleles associated with connectivity within and across the brain’s main functional networks
Source: Nat Commun. 2026 Sep 2;17:10427. doi: 10.1038/s41467-026-77476-2 (PMC13631237; doi:10.1038/s41467-026-77476-2)
Supplement: Supplementary file 2 [file 41467_2026_77476_MOESM19_ESM.pdf]

nature portfolio

Peer Review File

Common alleles associated with connectivity within and across the brain's main functional networks

Corresponding Author: Dr Xavier Caseras

This file contains all reviewer reports in order by version, followed by all author rebuttals in order by version.

Version 0:

Reviewer comments:

Reviewer #1

(Remarks to the Author)
This manuscript by Caseras et al, investigates the genetics of fMRI networks, in particular the common variant influences on the functional connectivity of seven canonical brain networks (Yeo et al.,). The authors perform multivariate GWAS using MOSTest to account for the fact that each network is composed of many (hundreds) of nodes, hence many thousands of node-pair functional connectivities.
The authors identify 114 independent genome-wide significant loci, map 315 candidate genes, and report both widely shared and network-specific genetic contributions to connectivity. They also highlight pleiotropic overlap between brain connectivity and risk for psychiatric disorders.
The article is well written and easy to follow, and the analyses seem sound, even if some more details could be provided (see below).

Major comments:

One of the main limitations pertains to the use of MOSTest, which does not provide easily interpretable effect sizes and prevents the use of the traditional post-GWAS methods. Other approaches, such as genomic SEM, allow for a more standard GWAS on the latent common factor, which could have solved the issue. This limitation is acknowledged in the discussion, but the authors may want to discuss ways to overcome this.
The theoretical advantages of MOSTest are mentioned but not necessarily demonstrated. I think it would help the narrative to focus 1 paragraph on this. What have you discovered that is new, and is it attributable to MOSTest? Some simple sensitivity analyses could further support this point, e.g. GWAS of average FC per network.

Did you perform global signal regression when calculating FC? Sorry if I missed it. This can be an important point to clarify and discuss, in terms of what it means for the generalisability of results and the genetic overlap you observe between networks.

Beyond the limitations of MOSTest, you still report intriguing evidence of pleiotropy with disorders of the brain.
It could be interesting to discuss what that pleiotropy might represent (true pleiotropy, confounders, causality, endophenotype).
Could you clarify or emphasise what new knowledge your manuscript is providing? Is it novel candidate genes for brain disorders? Pleiotropic loci? New insight into the biology or brain biomarkers of neuropsychiatric disorders? I felt that the discussion was more about the genetics of the brain than about what it tells us about how the brain relates to disorders.

I found table 2 and Figure 4 confusing, in that they tell very different stories about the genetic overlap between rsfmri and brain disorders. More detailed legends could help the reader make sense of them. I wonder if the two are really necessary? Could you pick the most informative metric and focus on it in the main text? Also numbers in Figure 4 are odd, if it is the fraction of the number of causal SNPs in rsfMRI, it should not be 1 for AD rather <<1 because the rsfMRI is a lot more pleiotropic than AD.

You mention in the limitation that the variable number of nodes per network may influence results. In light of this, can you draw conclusions about different association strengths with executive and sensory-motor networks? Overall, I feel that this could be a big problem, and sensitivity analyses would be welcome to evaluate how much the h2, pi1 and results may

change as you increase the number of nodes in a network. GWAS of the mean FC in each network may also be telling.

I did not know one could use MiXer on outputs from multivariate GWAS, it may be good to flag this for the reader.
What does it mean to get the h2 of a network? More generally what is the h2 of a multivariate GWAS? I find this difficult to interpret.

Minor:

In methods, clarify what covariates are used in the main text.

Provide number of SNPs used in the analysis

HWE is very stringent (we usually use 1e-6, is there a reason you used e-20?) and may not exclude many SNPs, have you checked your top SNPs do not have smaller HWE pvalues?

Provide number of nodes in each network

What relatedness cutoff did you use for exclusion? What does 4sd correspond to ? We typically use GRM<0.025 or <0.05.

A polygenicity of 45% for the visual network is quite striking, any thoughts on that?

Legends would benefit from being more detailed – to help the reader understand them in isolation.

“we implemented a new GWAS method (MOSTest) to investigate genetic common variant”
“We report on a novel multivariate approach to investigate common”
I found this misleading – you used a method that is a few years old now and did not develop a novel methodology (or I missed it).

“Our strategy increased considerably the detection of genome-wide significant index SNPs relative to previous literature”
“Our results widely expand our understanding of the common allele architecture of brain connectivity”
It would be good to properly quantify this, or tone it down, see my comment above.

Reviewer #2

(Remarks to the Author)
I co-reviewed this manuscript with one of the reviewers who provided the listed reports. This is part of the Nature Communications initiative to facilitate training in peer review and to provide appropriate recognition for Early Career Researchers who co-review manuscripts.

Reviewer #3

(Remarks to the Author)
Overall summary:
This manuscript addresses an important question: the extent to which common genetic variants affect functional connectivity within established brain networks, and how these connectivity architectures relate genetically to major neuropsychiatric disorders. The authors used a multivariate GWAS approach (MOSTest) to node-pair connectivity measures and reported substantial locus discovery, cross-network genetic overlap, gene-set enrichments, and shared architecture with psychiatric traits. The framing and biological motivation hold the potential to offer valuable insights into the genetic organisation of functional networks.
However, some of the methodological choices and interpretations raise concerns, particularly the use of MOSTest output in downstream MiXeR analyses that require signed effect estimates. These issues can affect the robustness and interpretability of some of the central findings. With clarification, methodological correction, and more cautious interpretation, the study could make a meaningful contribution to imaging-genetics research.

Major concerns:
1. Phenotype definition and justification of partial vs full correlations:
The description of the imaging phenotype requires clarification. The phrase “mean z-score of functional connectivity (i.e., partial correlation)” is ambiguous. Please clarify whether this refers to the Fisher z-transform of the partial correlation between mean region of interest (ROI) time series.
In addition, the rationale for preferring partial over full correlations should be strengthened. The manuscript states that full correlations are prone to genomic inflation, but this is not clearly justified within the MOSTest framework. It would help to (i) define what is meant by “full correlation,” (ii) explain why partial correlations alleviate the stated issue, or (iii) rephrase this as a conceptual rather than methodological necessity.
Clarification is needed in supplementary description of the phenotype definition:
“Once rfMRI individual data was mapped into this 100-node atlas, we applied AFNI’s 3Dnetcorr (Taylor & Saad, 2013) to obtain the Fisher Z-transformed functional partial correlation between each node pair assigned to the same RSN (Supplementary Table 2). Outlier node-node partial correlations (defined as 5x median absolute deviation) were declared

missing in further analyses. Most node pairs had not outlier values (70%), and the maximum datapoints removed from any node-pair was 32. …"
(i) For the partial correlation, was it calculated per node-pair per individual or per node-pair across the study population?
(ii) Related to (i), which distribution was referred to when defining outlier (outside 5x deviation)? E.g., the distribution of all node-node partial correlations per individual or across the entire study population?
(iii) "maximum datapoints removed from any node-pair was 32" – 32 out of how many? The current Supplementary Table 2 shows the final number of nodes per network; it would be helpful to have the total number before node filtering.

2. ROI coordinates and sizes:
Please provide a supplementary table listing the MNI coordinates and sizes of all ROIs included in each network. Although a reference is provided, including this information directly in the supplement would aid reproducibility.

3. A major concern is using MOSTest output as the input for bivariate MiXeR model:
MiXeR requires signed SNP effect-size estimates (beta or signed Z) to evaluate cross-trait overlap, which is derived from standard GWAS, as the mixture model distinguishes concordant and discordant genetic effects. As the authors correctly pointed out, MOSTest, however, produces unsigned omnibus test statistics that do not correspond to interpretable effect directions.
While univariate MiXeR can be applied to unsigned Z-scores, as shown in the original MOSTest paper (link below, where the authors explicitly noted that they derived estimates that do not requiring signed statistics), bivariate MiXeR cannot, and it is unclear how the authors ensured validity of the overlap estimates across networks or with psychiatric disorders.
The authors should clarify exactly what was supplied to MiXeR, justify the use of unsigned statistics mathematically, and/or provide sensitivity analyses using standard univariate GWAS summary statistics. Methods such as LDSC, which do not require signed effects, may be more appropriate for quantifying genetic overlap.
Also, the citation of the MOSTest method in main text (#18 in the current manuscript) appears incorrect; shouldn't it be PMID 32665545?
https://pubmed.ncbi.nlm.nih.gov/32665545/

4. Regarding association with neuropsychiatric disorders:
Because the MiXeR-based overlap analyses depend on the validity of the input statistics (see point #3), all downstream overlap findings should be reconsidered or interpreted cautiously unless a suitable method is used.
Currently the results show an apparent overlap with Alzheimer's disease (AD); if this persists under a method compatible with MOSTest output, the biological interpretation would be interesting and warrants further discussion. For example, do the AD-related genetic variants capture effects on neuronal loss or general brain integrity? Could these influence rfMRI connectivity? It would be interesting to clarify (i) how AD (and neurological diagnoses) were handled in the inclusion/exclusion of participants, and (ii) whether the authors view the rfMRI phenotype as capturing network-specific connectivity or more general brain integrity.

5. Discussion is very long and could be reduced by e.g., moving some detailed gene-by-gene descriptions to the supplementary text, to focus on conceptual interpretation and methodological limitations

Minor points:
1. MOSTest: please write the full name at the first apperance of the abbreviation.
2. Z-score or z-score, please use one style.
3. Supplementary: there is an unaddressed internal edit right before Reference; please clean up.

Version 1:

Reviewer comments:

Reviewer #1

(Remarks to the Author)
Thank you for the revisions and the response to comments. I feel that the changes to the manuscript remained minimal and that several points of discussion could have benefited from additional elements or details mentioned in the author's response. It is okay to slightly exceed the work count if it is to address comments.

In particular, I found that the changes in the text fell short of the explanations provided in the response letter for:

Reviewer 1 point 1 (side note genomic SEM allows to fit several latent factors),2, 3 (be more explicit about GSR), 7
R2: point 3 (justify in detail MIXER on unsigned effects, and include all references you mention in the response)

Reviewer #2

(Remarks to the Author)
I co-reviewed this manuscript with one of the reviewers who provided the listed reports. This is part of the Nature Communications initiative to facilitate training in peer review and to provide appropriate recognition for Early Career Researchers who co-review manuscripts.

Reviewer #3

(Remarks to the Author)
The authors have addressed the review comments.

Open Access This Peer Review File is licensed under a Creative Commons Attribution 4.0 International License, which permits use, sharing, adaptation, distribution and reproduction in any medium or format, as long as you give appropriate credit to the original author(s) and the source, provide a link to the Creative Commons license, and indicate if changes were made.
In cases where reviewers are anonymous, credit should be given to 'Anonymous Referee' and the source.
The images or other third party material in this Peer Review File are included in the article's Creative Commons license, unless indicated otherwise in a credit line to the material. If material is not included in the article's Creative Commons license and your intended use is not permitted by statutory regulation or exceeds the permitted use, you will need to obtain permission directly from the copyright holder.
To view a copy of this license, visit https://creativecommons.org/licenses/by/4.0/

Comments Reviewer #1

This manuscript by Caseras et al, investigates the genetics of fMRI networks, in particular the common variant influences on the functional connectivity of seven canonical brain networks (Yeo et al.,). The authors perform multivariate GWAS using MOSTest to account for the fact that each network is composed of many (hundreds) of nodes, hence many thousands of node-pair functional connectivities.
The authors identify 114 independent genome-wide significant loci, map 315 candidate genes, and report both widely shared and network-specific genetic contributions to connectivity. They also highlight pleiotropic overlap between brain connectivity and risk for psychiatric disorders.
The article is well written and easy to follow, and the analyses seem sound, even if some more details could be provided (see below).

Major comments:

1. One of the main limitations pertains to the use of MOSTest, which does not provide easily interpretable effect sizes and prevents the use of the traditional post-GWAS methods. Other approaches, such as genomic SEM, allow for a more standard GWAS on the latent common factor, which could have solved the issue. This limitation is acknowledged in the discussion, but the authors may want to discuss ways to overcome this.

The reviewer is correct, as we acknowledged in the manuscript, MOSTest carries post-GWAS analysis limitations; and as we also pointed in the introduction, other useful approaches to uncover common variants associated to the connectivity of functional brain networks have previously been applied (e.g. univariate GWAS of average functional correlation [Tissink et al. 2023, doi:10.1523/ENEURO.0242-22.2023], or of graph theory metrics [Foo et al 2021, doi: 10.1038/s41598-021-94182-9]). Genomic SEM (GSEM) is certainly another method that could contribute to it, albeit carrying other analytical limitations. GSEM would be useful to further investigate latent common factors across the different fMRI networks using as starting point univariate GWASes for each of them, or within each network if starting from univariate GWASes of each pair of nodes within the network. However, in our study, MOSTest was used for a slightly different purpose. Rather than focusing on common factors across all edges (each node-pair correlation), MOSTest allowed us to identify SNPs with significant effects on any edges within the same network. The assumption being that a SNP affecting the communication between any pair of nodes within a given network, would ultimately be relevant for the functioning of the overall network. We argue that this approach increases the power to detect relevant signals within rfMRI networks (as shown in the original MOSTest paper, van der Meer et al 2020 [doi: 10.1038/s41467-020-17368-1]).

We have now added the following to the revised manuscript:

Lines 367-372: "*Due to the intrinsic complexity of properly capturing this phenotype (i.e. rfMRI connectivity), there is unlikely to be a single approach that will best suit the investigation of its common genetic makeup. Our results add to previous and potential future approaches (e.g. genomic SEM), all carrying their own advantages and limitations, that in concert should bring clarity to the important question of the genetic determinants of brain's networks connectivity and its pleiotropy with neuropsychiatric conditions.*"

2. The theoretical advantages of MOSTest are mentioned but not necessarily demonstrated. I think it would help the narrative to focus 1 paragraph on this. What

have you discovered that is new, and is it attributable to MOSTest? Some simple sensitivity analyses could further support this point, e.g. GWAS of average FC per network

As the reviewer points out, we highlighted in the manuscript that the use of this multivariate approach that accounts for all edges within a network rather than a summary metric, would allow us to best capture the complexity of connectivity within each network and increase the statistical power. The argument of increased power is based on the results reported in the original MOSTest paper (van der Meer et al 2020; doi: 10.1038/s41467-020-17368-1), where this is shown. However, we agree with the reviewer that we have not necessarily proven that this is the case here. Therefore, following this suggestion, we have run a univariate GWAS of the average rfMRI within each network, showing these to detect far less significant hits. We have included the Manhattan plots for these univariate results in a supplementary figure, and refer to this sensitivity analysis within the Results section (lines 86-90): "*As expected, this multivariate approach substantially increased our ability to detect GWAS significant signals compared to a univariate approach of a summary metric such could be the within network average rfMRI connectivity (Supplementary figure 1).*"

3. Did you perform global signal regression when calculating FC? Sorry if I missed it. This can be an important point to clarify and discuss, in terms of what it means for the generalisability of results and the genetic overlap you observe between networks.

Global signal regression was not performed since partial correlation values were used to estimate pairwise node connectivity. Partial correlation intrinsically removes the global confound from any pair of timeseries, since all other timeseries are regressed from the pair before the correlation is calculated (Marrelec et al., 2006; doi: 10.1016/j.neuroimage.2005.12.057). For the study, partial correlations were used because they distinguish between direct and indirect connections between pairs of nodes (Smith et al., 2011; doi: 10.1016/j.neuroimage.2010.08.063). These have the advantage of reflecting conditional dependencies and improving sensitivity to individual pairwise node differences. For big data, they scale more meaningfully with large numbers of regions and participant datasets. We have added this into the methods (lines 412-418): "*Partial correlation measures were used instead of full correlation measures (i.e. Pearson correlation) as they: 1) intrinsically remove global confounds from any pair of timeseries[24]; 2) emphasise direct (rather than indirect) connections between node pairs[25]; 3) reflect conditional dependencies and improve sensitivity to individual pairwise node differences; 4) for big data, scale more meaningfully with large numbers of regions and participant datasets; 5) avoid genomic inflation due to the expected strong correlations across the phenotypes (node-pairs) included in the MOSTest*.."

4. Beyond the limitations of MOSTest, you still report intriguing evidence of pleiotropy with disorders of the brain.
It could be interesting to discuss what that pleiotropy might represent (true pleiotropy, confounders, causality, endophenotype).
Could you clarify or emphasise what new knowledge your manuscript is providing? Is it novel candidate genes for brain disorders? Pleiotropic loci? New insight into the biology or brain biomarkers of neuropsychiatric disorders? I felt that the discussion was more about the genetics of the brain than about what it tells us about how the brain relates to disorders

As outlined in the Introduction, the primary objective of this study was to characterise the genetic common architecture underlying functional brain connectivity. Specifically, we aimed

to identify common variants associated with rfMRI connectivity within each network, and to subsequently assess the extent of genetic network-specificity or commonality across them. As a secondary objective, given prior evidence of functional alterations in these networks in neuropsychiatric disorders, we examined the degree of shared genetic liability between these rfMRI networks and neuropsychiatric conditions.

We have now made this clearer in the Introduction (lines 66-73): "*As our main aim, we investigated the genetic overlap across the 7 networks. We predicted an important genetic overlap across them reflecting common biological processes linked to neural communication; however, we also expected certain degree of uniqueness due to their different functionality. Given prior evidence of functional alterations in these networks in neuropsychiatric conditions, we also investigated the genetic overlap between each network with common variants associated with severe mental disorders. Based on previous research, we predicted the DMN to show the largest genetic overlap with most mental disorders, with sensory networks showing the lowest."*

and in the discussion (lines 252-256): *"In this study we implemented a distinctive approach to investigate genetic common variant influences on brain's functional connectivity by including each node-pair partial correlation (edge) within a given rfMRI network in a multivariate GWAS (MOSTest). The primary aim of our study was unveiling the genetic makeup of functional connectivity within rfMRI networks and examining their genetic commonality and/or uniqueness(...)"* (and in lines 268-269): *"Addressing our secondary aim, we observed substantial genetic overlap between network's connectivity and neuropsychiatric disorders."*

As per discussing the pleiotropy between rfMRI networks and neuropsychiatric disorders, we thank the reviewer for this suggestion. This indeed reflects an interesting point of discussion in the human genomics literature. However, due to space limitations and the fact that the analyses we can perform on MOSTtest output are limited, we have now added to our Discussion a mention of types of pleiotropy compatible with our observed results, which allows a more nuanced interpretation of our general findings (lines 340-345): *"Nevertheless, we note that our analyses of causal variant overlap are based on a correlational method (MiXeR). Given the output of MOSTest (unsigned summary statistics) is not sufficient for more complex modelling approaches, at present we cannot perform a more detailed exploration of the degree to which our results reflect shared biological causality (i.e. true or horizontal pleiotropy) versus phenotype-driven correlations (i.e. vertical pleiotropy)."*

5. I found table 2 and Figure 4 confusing, in that they tell very different stories about the genetic overlap between rsfmri and brain disorders. More detailed legends could help the reader make sense of them. I wonder if the two are really necessary? Could you pick the most informative metric and focus on it in the main text? Also numbers in Figure 4 are odd, if it is the fraction of the number of causal SNPs in rsfMRI, it should not be 1 for AD rather <<1 because the rsfMRI is a lot more pleiotropic than AD.

We understand how the results presented in Table 2 and Figure 4 might appear contradictory at first; however, we believe that retaining both these metrics is important to paint a better picture of shared genetic effects across pairs of phenotypes.

Thanks to this reviewer's comment, we have revisited the Figure and made two important changes: (1). We realised that there was a mistake in the description of the metric presented which we have now resolved: this indicates the proportion of shared causal SNPs relative to the total number of estimated causal SNPs for the neuropsychiatric condition and not the

total causal SNPs for the rfMI network, as originally indicated. We understand that this error could have confused the reviewer, and we apologise for this. (2). We have moved the rfMRI networks to the vertices of the Spider chart and the neuropsychiatric conditions to be represented by the lines, since this more clearly displays our results. It can now be more noticeably appreciated that all estimated causal SNPs for AD based on Kunkle et al (2019) are shared estimated causal SNPs with most rfMRI network (100%), but for AD-SMN where ~90% of causal SNPs for AD are also estimated to be causal SNPs for this network.

Therefore, despite the DICE values (Table 2) indicating a low sharing of causal SNPs of AD with rfMRI networks, this was mostly due to differences in polygenicity, with AD being far less polygenic than rfMRI networks and therefore containing a low number of estimated causal SNPs.

We have now made this interpretation clearer in the manuscript (lines 226-237): "*The same methods to investigate cross-network genetic overlap were deployed to study genetic overlap of each network against mental disorders. The DICE metric from MiXer – proportion of shared causal SNPs relative to total number of causal SNPs for both traits - showed a moderate to high genetic overlap between all networks and all mental disorders, but for Alzheimer's Disease where it was low (Table 2). However, this metric is dependent on the polygenicity (i.e. the estimated number of causal SNPs) of the traits included in the analyses. The polygenicity for AD based on Kunkle et al[36] is low, and much lower than the polygenicity of rfMRI connectivity; therefore, erroneously suggesting lack of genetic overlap. Using alternative metrics, such as proportion of share causal SNPs relative to total causal SNPs for the neuropsychiatric condition (Figure 4) or total number of causal SNPs unique or shared between pairs of traits (Supplementary table 19), it can be appreciated that most estimated causal SNPs for AD appeared included within the estimated causal SNPs for each rfMRI network, therefore showing the larger overlap.*"

6. You mention in the limitation that the variable number of nodes per network may influence results. In light of this, can you draw conclusions about different association strengths with executive and sensory-motor networks? Overall, I feel that this could be a big problem, and sensitivity analyses would be welcome to evaluate how much the h2, pi1 and results may change as you increase the number of nodes in a network. GWAS of the mean FC in each network may also be telling.

Indeed, as discussed, heterogeneity in the number of edges across networks leads to differences in statistical power among the multivariate GWAS analyses. Consequently, the observation that a given association reaches significance in a GWAS based on a network with a higher edge density, but not in a GWAS conducted on networks with substantially fewer edges, should not be interpreted as evidence of network-specific or "unique" genetic effects. In light of this consideration, we adopted a conservative criterion for 'uniqueness' and a cautious interpretative framework. To be considered "unique", any signal should reach GWAS significance level in one network, but show a p-value > $1x10^{-5}$ in all other networks. Moreover, we exercised caution when attributing uniqueness to network-specific signals and placed greater emphasis on genetic associations that are shared across networks rather than those that appear unique.

Following the above, when interpreting the potential differences between sensory-motor and executive networks, we took into consideration that the SMN network was only smaller than DMN and DAN, but larger than FPN and VAN (see Supplemental table 2). Our interpretation came after comparing this network to all other executive networks and not only to the two with the largest number of edges. Moreover, we also used the locus-zoom (Supplemental

Figure 8) to ascertain whether there was still a possibility of subthreshold ($p > 1x10^{-5}$) elevations in those regions that appeared different between executive and sensory-motor networks. Locus-zoom were also inspected when considering potential ‘unique’ signals from any other network.

In summary, despite agreeing that edge density difference across networks is a weakness inherent to our approach that limits our interpretation of the results - as we noted in the Discussion-, we also believe that we have exerted the necessary caution in our interpretations and implemented the required strategies to limit the probability of spurious conclusions.

7. I did not know one could use MiXer on outputs from multivariate GWAS, it may be good to flag this for the reader.
What does it mean to get the h2 of a network? More generally what is the h2 of a multivariate GWAS? I find this difficult to interpret.

The outputs of MOSTest are summary stats like the outputs of any univariate GWAS, with the particularity that all effects sizes are positive (not signed); therefore, the interpretation of outputs is the same as applying MiXer after univariate GWAS. Please see reply to Reviewer 3 3rd point for further information on the use of MiXer after MOSTest.

Minor:
- In methods, clarify what covariates are used in the main text.

We have moved the information on the covariates from the supplemental methods to now appear in the main text (lines 421-427): “*The remaining correlations were regressed on age^2 (2nd degree orthogonal polynomial), sex, age^2×sex, mean resting-state functional MRI head motion averaged across space and time points (log-transformed), imaging centre attended, date attended imaging centre, X-, Y- and Z-head position in the scanner, starting table-Z position and the first 10 principal components from genetic population stratification. The resulting residuals were normalised using a rank-based inverse normal transformation[26] and enetred into MOSTest.*”

- Provide number of SNPs used in the analysis

We *have added this information in the revised version of the manuscript (lines 436-437): “As result, 4,341,165 SNPs were retained and examined in each MOSTest GWAS analysis.*”

- HWE is very stringent (we usually use 1e-6, is there a reason you used e-20?) and may not exclude many SNPs, have you checked your top SNPs do not have smaller HWE pvalues?

We applied a stringent Hardy–Weinberg equilibrium (HWE) filter of $P < 1×10^{-20}$ (rather than the more lenient $P < 1×10^{-6}$) as a pragmatic quality-control choice tailored to the design and scale of this analysis. In very large datasets, even trivial departures from HWE can achieve extremely small P-values, such that a threshold like $1×10^{-6}$ may exclude substantial numbers of well-imputed, high-quality variants purely because of sample size rather than genuine genotyping error. By adopting a more stringent cut-off ($1×10^{-20}$), we retain sensitivity to gross departures consistent with technical artefact while reducing unnecessary variant loss driven by power and study design, thereby minimising the risk of discarding true associations through overly aggressive HWE filtering.

We now mention this in the Methods section (lines: 431-436): " *We applied additional genetic quality control including restricting the dataset to variants with a minor allele count of at least five, removing SNPs with significant deviation from Hardy-Weinberg Equilibrium (p < 1 × 10* $^{-20}$*, due to the large sample size we applied this stringent p-value to retain sensitivity to gross departures consistent with technical artefact while reducing unnecessary variant loss driven by power), and excluding SNPs and individuals with more than 2% missingness.*"

- Provide number of nodes in each network

Number of nodes and edges for each network is presented in Supplemental table 2, which is referred to the beginning of the Results section (lines 79-84): *"Each network's multivariate MOSTest GWAS included all potential pairwise connections (edges) between its constituent pre-determined brain regions (nodes). Edges were defined as the partial correlation between the average BOLD timeseries of any given node-pair. The number of nodes and edges, therefore of partial correlations included in each network's GWAS MOSTest, are presented in supplementary table 2, ranging from 5 nodes (10 pairwise correlations) for LN to 24 nodes (276 pairwise correlations) for the DMN.".*

S2: The number of nodes and edges per resting-state network

rfMRI network	Nodes	Edges
DMN	24	276
VN	17	136
DAN	15	105
SMN	14	91
FPN	13	78
VAN	12	66
LN	5	10

- What relatedness cutoff did you use for exclusion? What does 4sd correspond to ? We typically use GRM<0.025 or <0.05.

Using PLINK2, we applied a --king-cutoff 0.0442. A cut-off of 0.0442 corresponds approximately to the boundary between third-degree relatives and more distant/unrelated individuals. The four-standard-deviation threshold - for the between individual kinship estimate - was applied to identify individuals whose apparent relatedness was likely driven by genotyping error rather than true biological relationships. In previous studies we have noted that systematic genotyping artefacts can produce spurious inflation of pairwise relatedness estimates, leading to individuals appearing overly related to multiple, otherwise unrelated, cohort members. To detect this pattern, we calculated pairwise relatedness across the full cohort and identified individuals involved in an excess of relatedness estimates lying more than four standard deviations above the cohort mean. These individuals were excluded to minimise the impact of technical artefacts on downstream association analyses. This is part of our standard genotyping QC pipeline. We have rewritten this section of the methods to make it clearer (lines 437-440): "*Individuals with excessive homozygosity or heterozygosity - defined as more than four standard deviations from the sample mean - were removed, as were individuals showing excessive relatedness to non-*

family members, also defined as greater than four standard deviations from the kinship estimate mean."

- A polygenicity of 45% for the visual network is quite striking, any thoughts on that?

As mentioned in the manuscript, the Visual Network showed poor replicability across subsamples, and a poor fit of the MiXer (line 265). Therefore, this metric should be interpreted with caution, which is the reason why we did not discuss it further and we excluded VN from subsequent analyses. To stress this further, we have now emphasised caution when interpreting these results (lines 271-275): *"We excluded the LN and the VN networks from downstream analyses, which limits our ability to draw conclusions about them. This decision was based on three factors: (i) low estimated heritability and polygenicity, (ii) poor reproducibility in internal validation using split-sample analyses, and (iii) difficulties fitting the MiXer model to their data, which calls for caution when interpreting any of the results obtained for these two networks*."

- Legends would benefit from being more detailed – to help the reader understand them in isolation.

We have now extended the Figure legends to make them more self-explanatory

- "we implemented a new GWAS method (MOSTest) to investigate genetic common variant"
"We report on a novel multivariate approach to investigate common"
I found this misleading – you used a method that is a few years old now and did not develop a novel methodology (or I missed it).

We agree with the reviewer, we intended to highlight that our approach of using MOSTest to investigate common genetic variants associated with rfMRI connectivity was novel, not that MOSTest is a novel method. We see that our choice of wording was not always clear in that respect and have now corrected this.

Lines 22-23 in the Abstract now read: "*We report on a multivariate approach allowing for a better representation of this complexity, while boosting statistical power*."

Lines 252-254 at the beginning of the Discussion now read "*In this study we implemented a distinctive approach to investigate genetic common variant influences on brain's functional connectivity by including each node-pair partial correlation (edge) within a given rfMRI network in a multivariate GWAS (MOSTest).*"

- "Our strategy increased considerably the detection of genome-wide significant index SNPs relative to previous literature"
"Our results widely expand our understanding of the common allele architecture of brain connectivity"
It would be good to properly quantify this, or tone it down, see my comment above.

We have now included univariate GWAS of average connectivity within each network to prove that our multivariate approach considerably increases the detection of genome-wide significant SNPs.

Comments Reviewer #2

I co-reviewed this manuscript with one of the reviewers who provided the listed reports. This is part of the Nature Communications initiative to facilitate training in peer review and to provide appropriate recognition for Early Career Researchers who co-review manuscripts.

Thanks for undertaking the review of our manuscript; we appreciate the contribution of all reviewers.

Comments Reviewer #3

Overall summary:
This manuscript addresses an important question: the extent to which common genetic variants affect functional connectivity within established brain networks, and how these connectivity architectures relate genetically to major neuropsychiatric disorders. The authors used a multivariate GWAS approach (MOSTest) to node-pair connectivity measures and reported substantial locus discovery, cross-network genetic overlap, gene-set enrichments, and shared architecture with psychiatric traits. The framing and biological motivation hold the potential to offer valuable insights into the genetic organisation of functional networks.
However, some of the methodological choices and interpretations raise concerns, particularly the use of MOSTest output in downstream MiXeR analyses that require signed effect estimates. These issues can affect the robustness and interpretability of some of the central findings. With clarification, methodological correction, and more cautious interpretation, the study could make a meaningful contribution to imaging-genetics research.

Major concerns:

1. Phenotype definition and justification of partial vs full correlations:
The description of the imaging phenotype requires clarification. The phrase “mean z-score of functional connectivity (i.e., partial correlation)” is ambiguous. Please clarify whether this refers to the Fisher z-transform of the partial correlation between mean region of interest (ROI) time series.
In addition, the rationale for preferring partial over full correlations should be strengthened. The manuscript states that full correlations are prone to genomic inflation, but this is not clearly justified within the MOSTest framework. It would help to (i) define what is meant by “full correlation,” (ii) explain why partial correlations alleviate the stated issue, or (iii) rephrase this as a conceptual rather than methodological necessity.
Clarification is needed in supplementary description of the phenotype definition: “Once rfMRI individual data was mapped into this 100-node atlas, we applied AFNI’s 3Dnetcorr (Taylor & Saad, 2013) to obtain the Fisher Z-transformed functional partial correlation between each node pair assigned to the same RSN (Supplementary Table 2). Outlier node-node partial correlations (defined as 5x median absolute deviation) were declared missing in further analyses. Most node pairs had not outlier values (70%), and the maximum datapoints removed from any node-pair was 32. …”
(i) For the partial correlation, was it calculated per node-pair per individual or per node-pair across the study population?

(ii) Related to (i), which distribution was referred to when defining outlier (outside 5x deviation)? E.g., the distribution of all node-node partial correlations per individual or across the entire study population?
(iii) "maximum datapoints removed from any node-pair was 32" – 32 out of how many? The current Supplementary Table 2 shows the final number of nodes per network; it would be helpful to have the total number before node filtering.

To clarify all the above points and made them more accessible to the reader, we have moved all the methodological information relative to the calculation of the phenotype into the main document, rather than presenting part of it into the supplements. We have re-written this section to improve its clarity.

The Methods section referring to the above points now reads (lines 409-428):

"*We registered a 100-node resting-state atlas*[22] *- for which each node can be allocated to one of the 7 resting state networks - onto each participant's rfMRI scan (further information on this atlas is provided in the supplementary methods). Using AFNI's 3DNetcorr*[23]*, we obtained the Fisher Z-transformed functional connectivity between each node-pair within each network by estimating partial correlation between the time series. Partial correlation measures were used instead of full correlation measures (i.e. Pearson correlation) as they: 1) intrinsically remove global confounds from any pair of timeseries*[24]*; 2) emphasise direct (rather than indirect) connections between node pairs*[25]*; 3) reflect conditional dependencies and improve sensitivity to individual pairwise node differences; 4) for big data, scale more meaningfully with large numbers of regions and participant datasets; 5) avoid genomic inflation due to the expected strong correlations across the phenotypes (node-pairs) included in the MOSTest. Outlier node-node partial correlations for each individual pair (defined as 5x median absolute deviation) were excluded from further analyses. Most node-pairs did not have outlier values across the population (70% of node-pairs), and the maximum number of data-points (partial correlation values) removed from any node-pair was 32. The remaining correlations were regressed on age*^{2} *(2nd degree orthogonal polynomial), sex, age*^{2}*×sex, mean resting-state functional MRI head motion averaged across space and time points (log-transformed), imaging centre attended, date attended imaging centre, X-, Y- and Z-head position in the scanner, starting table-Z position and the first 10 principal components from genetic population stratification. The resulting residuals were normalised using a rank-based inverse normal transformation*[26] *and entered into MOSTest.*"

2. ROI coordinates and sizes:
Please provide a supplementary table listing the MNI coordinates and sizes of all ROIs included in each network. Although a reference is provided, including this information directly in the supplement would aid reproducibility.

The atlas used to segment the brain into 100 nodes in this study is widely used in the field and is known as the "Schaefer 100". As pointed in the methods, we obtained this atlas from its original source and after transforming this template from MNI into each participant's individual space, we overlaid this atlas onto the participants' functional data. Rather than reproducing the original information about the atlas in this manuscript, we think it is better to provide a direct link to the primary source for it, which will provide the required information for replication in the same or any alternative sample. We've modified the relevant supplementary methods section to provide more detail, as well as added the direct link to the Shaefer 100 atlas: "*Resting-state fMRI data was transformed to T1 space via FLIRT using an individual's T1-weighted structural image (3D MPRAGE, sagittal, in-plane acceleration*

iPAT=2, voxel size 1mm isotropic, field-of-view 208x256x256), and from T1 space to standard MNI152 space via FNIRT (Andersson, Smith et al 2007, Andersson, Jenkinson et al 2007). To segment the brain into 100 nodes, a widely used public brain atlas was employed (Schaefer et al., 2018). Information about this atlas (including download) can be found at https://github.com/ThomasYeoLab/CBIG/tree/master/stable_projects/brain_parcellation/Schaefer2018_LocalGlobal. This 100-node atlas preserves the 7 RNS parcellation (Yeo et al., 2011) implemented in this study, and uses a gradient-weighted Markov random field model to integrate between local gradient and global similarity approaches, to produce further homogenously grouped rfMRI signals whose boundaries agree with histological and visuospatial divisions. This atlas was transformed from MNI space to each individual participant's rsfMRI space by inverting the individual-to-MNI registration matrix. This allowed to calculate each participant's average rsfMRI time series within each of the 100-node ROIs."

3. A major concern is using MOSTest output as the input for bivariate MiXeR model: MiXeR requires signed SNP effect-size estimates (beta or signed Z) to evaluate cross-trait overlap, which is derived from standard GWAS, as the mixture model distinguishes concordant and discordant genetic effects. As the authors correctly pointed out, MOSTest, however, produces unsigned omnibus test statistics that do not correspond to interpretable effect directions.
While univariate MiXeR can be applied to unsigned Z-scores, as shown in the original MOSTest paper (link below, where the authors explicitly noted that they derived estimates that do not requiring signed statistics), bivariate MiXeR cannot, and it is unclear how the authors ensured validity of the overlap estimates across networks or with psychiatric disorders.
The authors should clarify exactly what was supplied to MiXeR, justify the use of unsigned statistics mathematically, and/or provide sensitivity analyses using standard univariate GWAS summary statistics. Methods such as LDSC, which do not require signed effects, may be more appropriate for quantifying genetic overlap.
Also, the citation of the MOSTest method in main text (#18 in the current manuscript) appears incorrect; shouldn't it be PMID 32665545?
https://pubmed.ncbi.nlm.nih.gov/32665545/

Thank you for your comment, however we wish to clarify a misunderstanding and provide an update to the manuscript accordingly. One of the reasons for choosing MiXeR as a downstream analysis over LDSC for these MOSTest summary statistics is that you can run bivariate MiXeR with unsigned data. MiXeR does not require effect directions to estimate whether variants are shared - but the interpretation is limited as bivariate MiXeR with unsigned data estimates only reveal shared genetic involvement, not shared biological direction. It answers "Do the same SNPs influence both traits?" not "Do they influence them in the same direction? This use of MiXeR independent of direction of effect is noted in Hindley et al, 2022; doi: 10.1176/appi.ajp.21101051, Shadrin et al., 2025; doi: 10.1186/s13073-025-01528-3, and Wu et al., 2025; doi: 10.1093/schbul/sbaf096. We note that while LDSC heritability can be estimated using unsigned statistics, cross-trait LDSC genetic correlation requires signed effect estimates. Therefore, LDSC is not directly applicable to MOSTest output for estimating directional genetic correlation.

To clarify this point we have included the following in our Methods section (lines 488-492): *"Because MOSTest produces unsigned omnibus association statistics, bivariate MiXeR analyses in this study quantify shared genetic involvement between traits but do not permit inference regarding concordant or discordant effect directions. Overlap estimates should*

therefore be interpreted as reflecting shared causal variant participation rather than directional pleiotropy."

Regarding the citation of the original MOST paper, the reviewer is right, we mistakenly used the incorrect reference. We thank the reviewer for noticing this and we apologise for this mistake. The correct paper is now referenced in the manuscript.

4. Regarding association with neuropsychiatric disorders:
Because the MiXeR-based overlap analyses depend on the validity of the input statistics (see point #3), all downstream overlap findings should be reconsidered or interpreted cautiously unless a suitable method is used.
Currently the results show an apparent overlap with Alzheimer's disease (AD); if this persists under a method compatible with MOSTest output, the biological interpretation would be interesting and warrants further discussion. For example, do the AD-related genetic variants capture effects on neuronal loss or general brain integrity? Could these influence rfMRI connectivity? It would be interesting to clarify (i) how AD (and neurological diagnoses) were handled in the inclusion/exclusion of participants, and (ii) whether the authors view the rfMRI phenotype as capturing network-specific connectivity or more general brain integrity.

Please see above with regards of the adequacy of MiXer to examine the overlap between rfMRI network connectivity and neuropsychiatric conditions.

Regarding the reviewer interest on the overlap between AD and network connectivity, unfortunately our results do not allow a mechanistic interpretation of this pleiotropy. We still find it interesting and informative, though, that risk variants for AD importantly overlap with common variants that influence brain's connectivity. As such, one could speculate that genetic risk for AD, and ultimately the presence of this condition, would be associated with the genetic makeup of the brain facilitating its overall functional integration and synchronous firing. We have now introduced the following in the Discussion (lines 445-449): "*Although no mechanistic interpretation can be drawn from our results, one could speculate that this substantial overlap indicates that risk variants predisposing a person to develop AD, and ultimately the presence of the condition, would also be associated with the brain's ability to maintain an adequate functional integration, facilitating the communication across distinct areas of the brain.* "

Regarding this reviewer's specific comments on**: (i) how neurological conditions were handled?** As we mention in the manuscript, we excluded participants on the basis of "*self-reported or hospital-record diagnoses of neuropsychiatric conditions known to affect brain structure or function (i.e. schizophrenia, psychosis, bipolar disorder, autism spectrum disorder or intellectual disability, alcohol or substances dependence, dementia, Parkinson's, Alzheimer's, Huntington's, neurodegenerative disorders, or brain cancer)*." **(ii) whether the authors view the rfMRI phenotype as capturing network-specific connectivity or more general brain integrity.** Our phenotypes as compute with the aim to capture connectivity within each network. However, we interpret the substantial genetic overlap found across networks as suggestive of most signals detected to participate of the general brain integrity.

5. Discussion is very long and could be reduced by e.g., moving some detailed gene-by-gene descriptions to the supplementary text, to focus on conceptual interpretation and methodological limitations

The Discussion has been reduced while reformatting the manuscript according to Nature Communications' submission guidelines.

Minor points:
- MOSTest: please write the full name at the first apperance of the abbreviation.

We thank the reviewer for noticing this oversight. We have now introduced this test full name (Multivariate Omnibus Statistical Test) in its first appearance in our manuscript.

- Z-score or z-score, please use one style.

We have homogenised on z-score

- Supplementary: there is an unaddressed internal edit right before Reference; please clean up.

This has now been resolved.

We want to thank the reviewers for their comments to our revisited version of the manuscript. We have accommodated all their suggestions.

Reviewer #1 (Remarks to the Author):

> *Thank you for the revisions and the response to comments. I feel that the changes to the manuscript remained minimal and that several points of discussion could have benefited from additional elements or details mentioned in the author's response. It is okay to slightly exceed the work count if it is to address comments.*
>
> *In particular, I found that the changes in the text fell short of the explanations provided in the response letter for:*
>
> *Reviewer 1 point 1 (side note genomic SEM allows to fit several latent factors),2, 3 (be more explicit about GSR), 7-R2: point 3 (justify in detail MIXER on unsigned effects, and include all references you mention in the response)*

Regarding this reviewer's original point 1 (justification of MOST and mention to genomic SEM), we have now added in the manuscript the wording that appeared in our response letter, lines (372-376): "*Whereas genomic SEM can be applied to further investigate latent common factors across the different networks using as starting point univariate GWASes from each of them, MOSTest allowed us to identify SNPs with significant effects on any edges within the same network, assuming that any SNP affecting the communication between a pair of nodes within a network would affect that network's overall function.*"

Regarding their original point 2 (advantages of MOST and novelty of results), we have edited the first paragraph of the discussion to more explicitly highlight the main contributions of this study to the field, as requested.

As per original point 3 (global signal regression), to be more explicit, we have now added to the manuscript (lines 423-425): ***"****1) intrinsically remove global confounds from any pair of timeseries[48] since all other timeseries are regressed from the pair before the correlation is calculated, as global signal regression would dol; 2) (…)"*

Finally, in response to their point 7 (coincident with the other reviewer's point 3), we have now added to the manuscript (lines 496-499): "*MiXeR does not require effect directions to estimate whether variants are shared, as previously noted[55-57], but the interpretation is limited as bivariate MiXeR with unsigned data estimates only reveal shared genetic involvement, not shared biological direction*.", which includes the references to justify MiXeR use with unsigned estimates.

Reviewer #2 (Remarks to the Author):

> I co-reviewed this manuscript with one of the reviewers who provided the listed reports. This is part of the Nature Communications initiative to facilitate training in peer review and to provide appropriate recognition for Early Career Researchers who co-review manuscripts.

We thank this reviewer for revising our re-submission of this manuscript.

Reviewer #3 (Remarks to the Author):

> The authors have addressed the review comments.

We thank the reviewer for their time reviewing the revised version of this manuscript and their previous comments
