# Supplementary material for: Common alleles associated with connectivity within and across the brain’s main functional networks
Source: Nat Commun. 2026 Sep 2;17:10427. doi: 10.1038/s41467-026-77476-2 (PMC13631237; doi:10.1038/s41467-026-77476-2)
Supplement: Supplementary file 3 [file 41467_2026_77476_MOESM1_ESM.pdf]

Common alleles associated with connectivity within and across the brain's main functional networks

SUPPLEMENTARY INFORMATION

Supplementary Methods
Supplementary Figures 1-10
Supplementary Tables 1- 4

Supplementary Methods

UK Biobank sample

UK Biobank (https://www.ukbiobank.ac.uk)[1] is a large-scale, population-based biomedical resource designed to support health-related research worldwide. Between 2006 and 2010, it recruited approximately 500,000 participants who provided informed consent for the collection and long-term use of detailed health, lifestyle, biometric, and genetic data. A subset of these participants also underwent brain MRI imaging. At the time of this study, resting-state fMRI and genotype data were available for 37,716 individuals. Following quality control procedures and computational steps described in the main document and below, 33,173 participants remained in our sample. Of these, 587 participants were excluded due to self-reported or hospital-record diagnoses of neuropsychiatric conditions known to affect brain structure or function (i.e. schizophrenia, psychosis, bipolar disorder, autism spectrum disorder or intellectual disability, alcohol or substances dependence, dementia, Parkinson's, Alzheimer's, Huntington's, neurodegenerative disorders, or brain cancer). An additional 1,766 participants were excluded due to missing data on relevant covariates or for exhibiting outlier values (defined as >5 times the median absolute deviation) for intracranial volume, considered a proxy for scanning data quality.
The final analytic sample included 30,820 participants (53% female, mean age 63 [range 45-81]), scanned at three UK Biobank centres: Cheadle (61.6%), Newcastle (25.7%) and Reading (12.6%). Further demographic details are provided in Supplemental Table 1.

Resting-state fMRI data acquisition

Full information on UK Biobank acquisition and pre-processing steps is provided elsewhere: https://biobank.ctsu.ox.ac.uk/crystal/crystal/docs/brain_mri.pdf. In brief, resting-state functional MRI data was obtained via three equivalent Siemens Skyra 3T scanners with standard Siemens 32-channel receive head coils during a 6-minute scan in the absence of any explicit task/stimulation, using a voxel size of 2.4mm isotropic, field-of-view: 88x88x64 matrix, repetition time of 0.735s , echo-time of 39ms, GE-EPI with x8 multislice acceleration and flip angle 52°. Pre-processing included motion correction using MCFLIRT[2], grand-mean intensity normalisation, high-pass temporal filtering, EPI unwarping, GDC unwarping and structural artefact removal by ICA+FIX processing[3-5].

Resting-state fMRI data was transformed to T1 space via FLIRT using an individual's T1-weighted structural image (3D MPRAGE, sagittal, in-plane acceleration iPAT=2, voxel size 1mm isotropic, field-of-view 208x256x256), and from T1 space to standard MNI152 space via FNIRT[2,6] . To segment the brain into 100 nodes, a widely used public brain atlas was employed[7]. Information about this atlas (including download) can be found at https://github.com/ThomasYeoLab/CBIG/tree/master/stable_projects/brain_parcellation/Schaefer2018_LocalGlobal. This 100-node atlas preserves the 7 RNS parcellation[8] implemented in this study, and uses a gradient-weighted Markov random field model to integrate between local gradient and global similarity approaches, to produce further homogenously grouped rfMRI signals whose boundaries agree with histological and visuospatial divisions. This atlas was transformed from MNI space to each individual participant's rsfMRI space by inverting the individual-to-MNI registration matrix. This allowed to calculate each participant's average rsfMRI time series within each of the 100-node ROIs.

Genetic quality control

Genotyping for UK Biobank was performed on two similar arrays (Affymetrix UK BiLEVE Axiom Array and UK Biobank Axiom Array), covering over 800,000 markers, with data

available for approximately 488,000 individuals. Imputation, using IMPUTE4 (https://jmarchini.org/software/) and three reference panels (HRC[9], UK10K[10], and 1000 Genomes Phase 3[11]), expanded the dataset to ~96 million variants.

We applied additional quality control steps included filtering SNPs with minor allele count <5, Hardy-Weinberg disequilibrium ($p < 1 \times 10^{-20}$), or >2% missingness. Individuals were excluded for >2% genotype missingness, extreme homozygosity/heterozygosity (greater than ±4 SD), or excess relatedness. Ancestry principal components were calculated in PLINK, and individuals within ±4 SD of the GBR reference population (from 1000 Genomes Phase 3) on the first three PCs were retained to ensure a genetically homogeneous sample (see Figure 1 below).

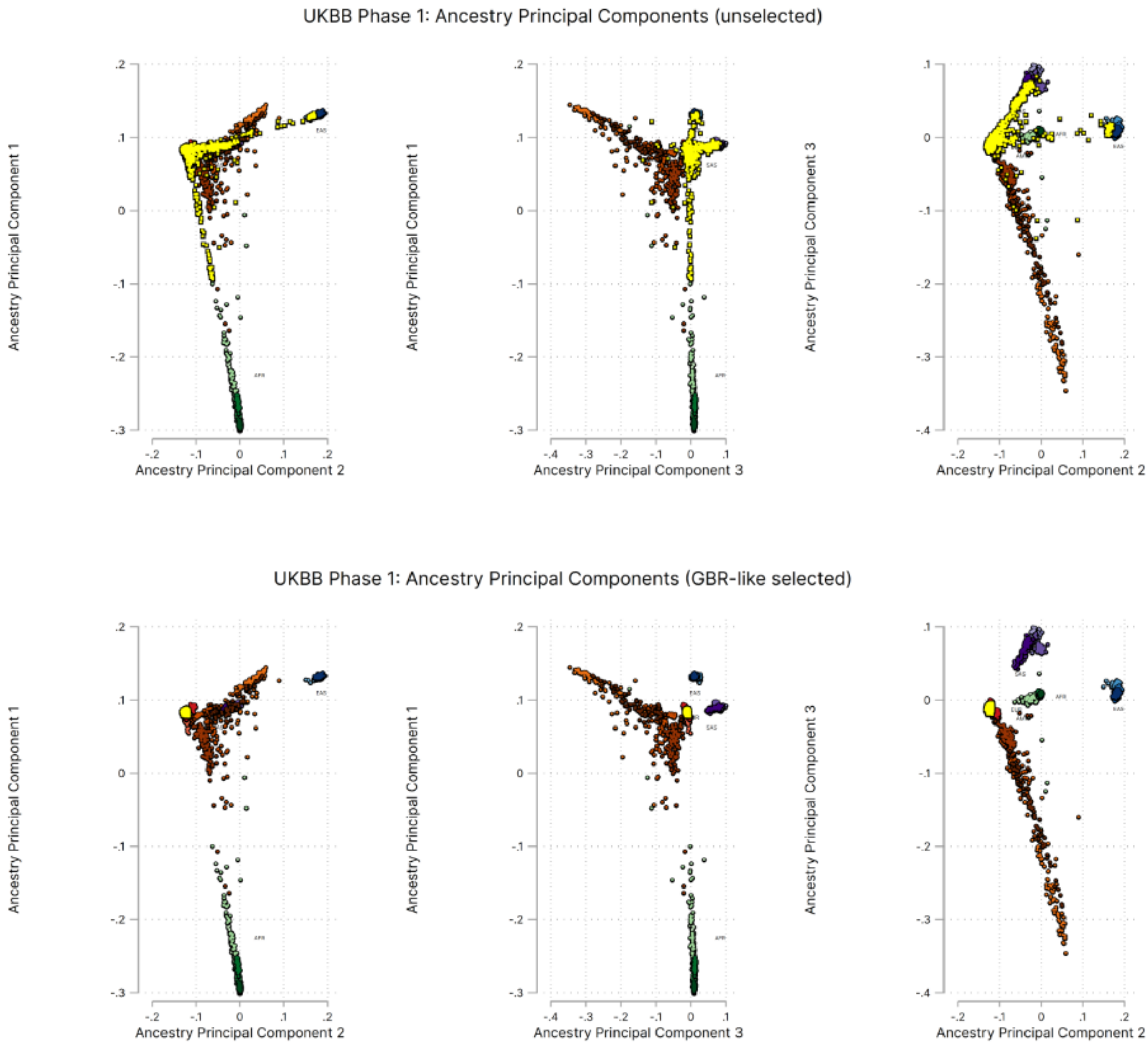

Supplementary Figure 1. **Ancestral Principal Components (PCs) of the UK Biobank Phase 1 Subset.** Each panel displays UK Biobank (UKBB) participants (yellow) in comparison to reference populations from the 1000 Genomes Project: East Asian and South Asian (blue), African (green), and European/Latino (red/orange). The top panel shows the first three PCs for the full UKBB subset. The bottom panel shows only UKBB individuals genetically similar to the GBR (British) reference group.

MiXer Analysis

To model the polygenic architecture of resting-state fMRI (rsfMRI) networks, we used the `MiXeR` software[12], which estimates SNP-based heritability, polygenicity (the estimated number of causal variants), and the extent of genetic overlap between traits. Summary statistics from each network's multivariate GWAS were first harmonised and filtered to

retain high-quality, common variants shared across datasets. These were intersected with the 1000 Genomes Phase 3 European reference panel (MAC ≥ 5), and regions of long-range linkage disequilibrium were excluded (https://genome.sph.umich.edu/wiki/Regions_of_high_linkage_disequilibrium_\(LD\). SNPs were further limited to those present in all relevant networks, and the reference panel was split by chromosome using `PLINK` to generate MiXeR-compatible inputs.
For each network, we ran the `fit1` and `test1` `MiXeR` models across 20 replicates to estimate trait-specific (univariate) genetic architecture parameters, including the proportion of causal variants and residual inflation. We then applied the `fit2` and `test2` models to each pair of networks (bivariate) to estimate the number of shared causal variants and the correlation of effect sizes in shared components. Model outputs were aggregated and summarised to produce heritability estimates, polygenicity indices, overlap measures, and genetic correlations.

Supplementary Figures

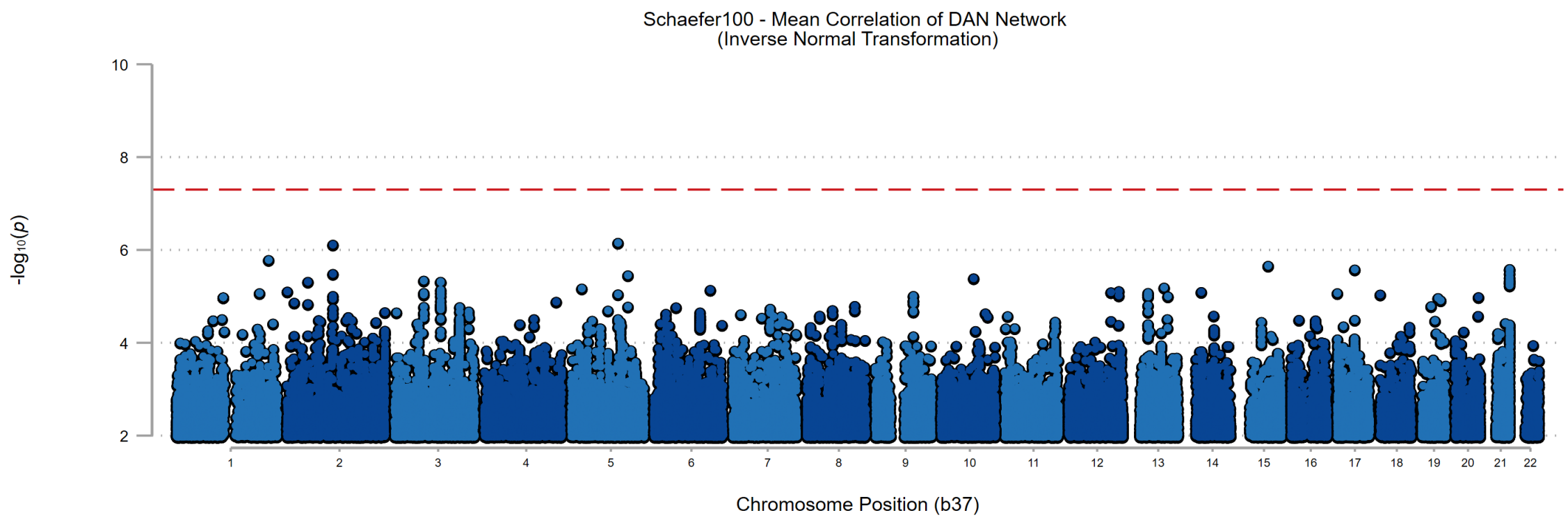

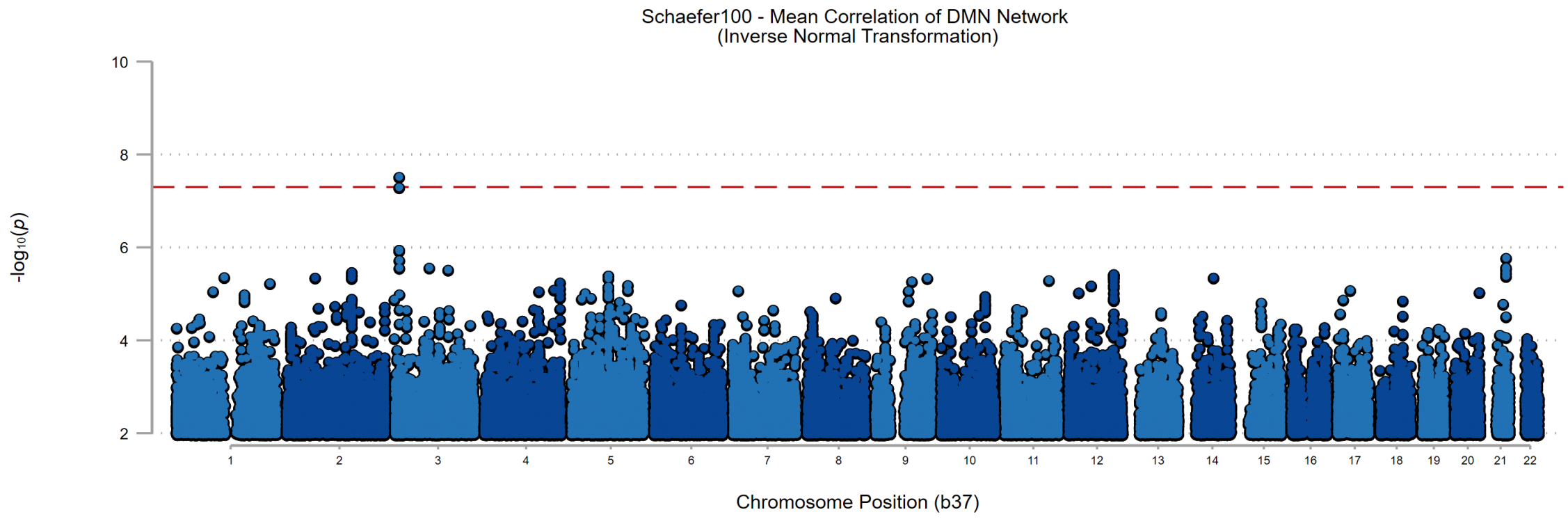

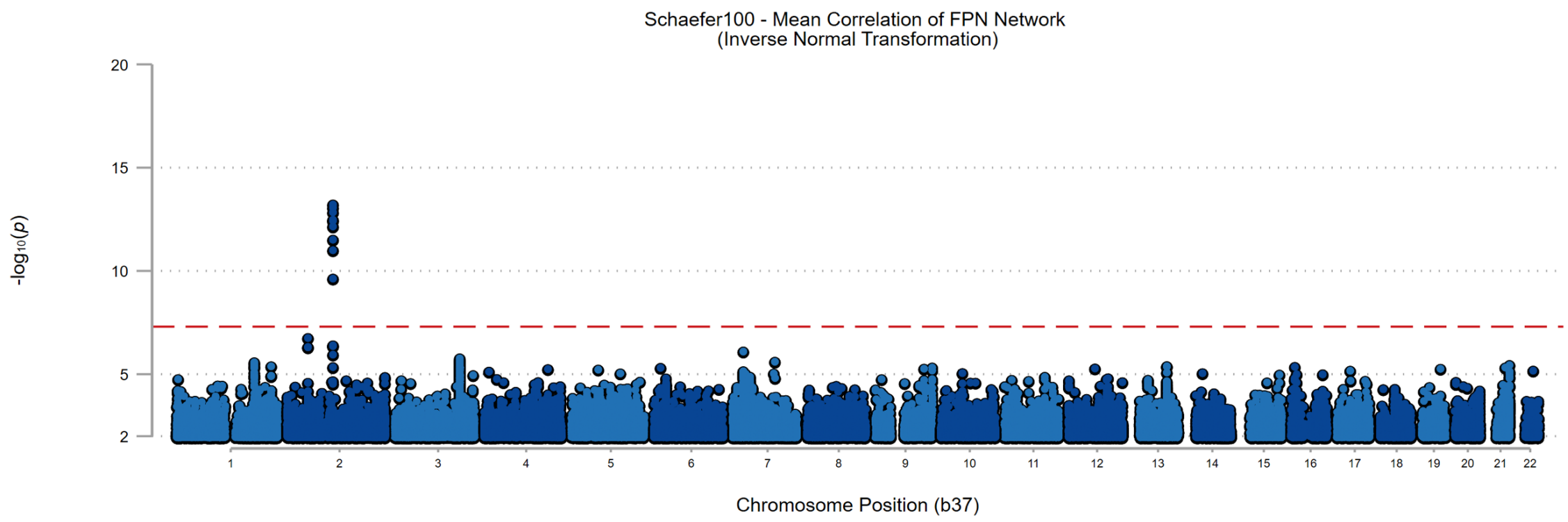

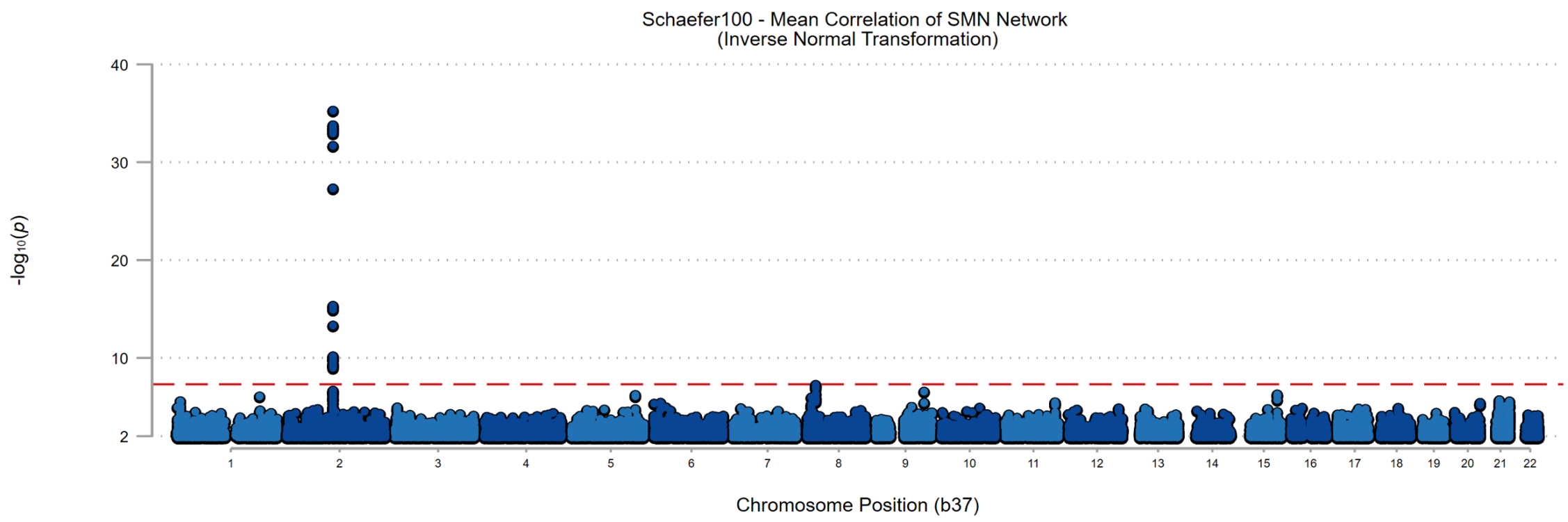
Schaefer100 - Mean Correlation of SMN Network
(Inverse Normal Transformation)
-log10(p)
40
30
20
10
2
1 2 3 4 5 6 7 8 9 10 11 12 13 14 15 16 17 18 19 20 21 22
Chromosome Position (b37)

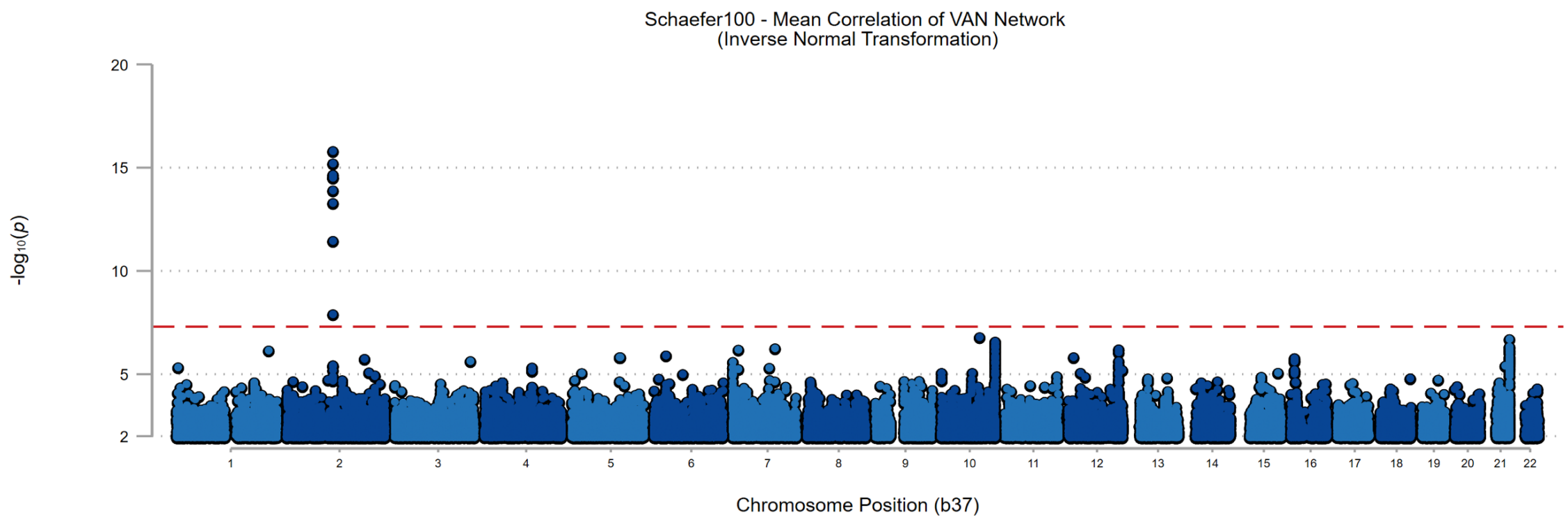
Schaefer100 - Mean Correlation of VAN Network
(Inverse Normal Transformation)
-log10(p)
20
15
10
5
2
1 2 3 4 5 6 7 8 9 10 11 12 13 14 15 16 17 18 19 20 21 22
Chromosome Position (b37)

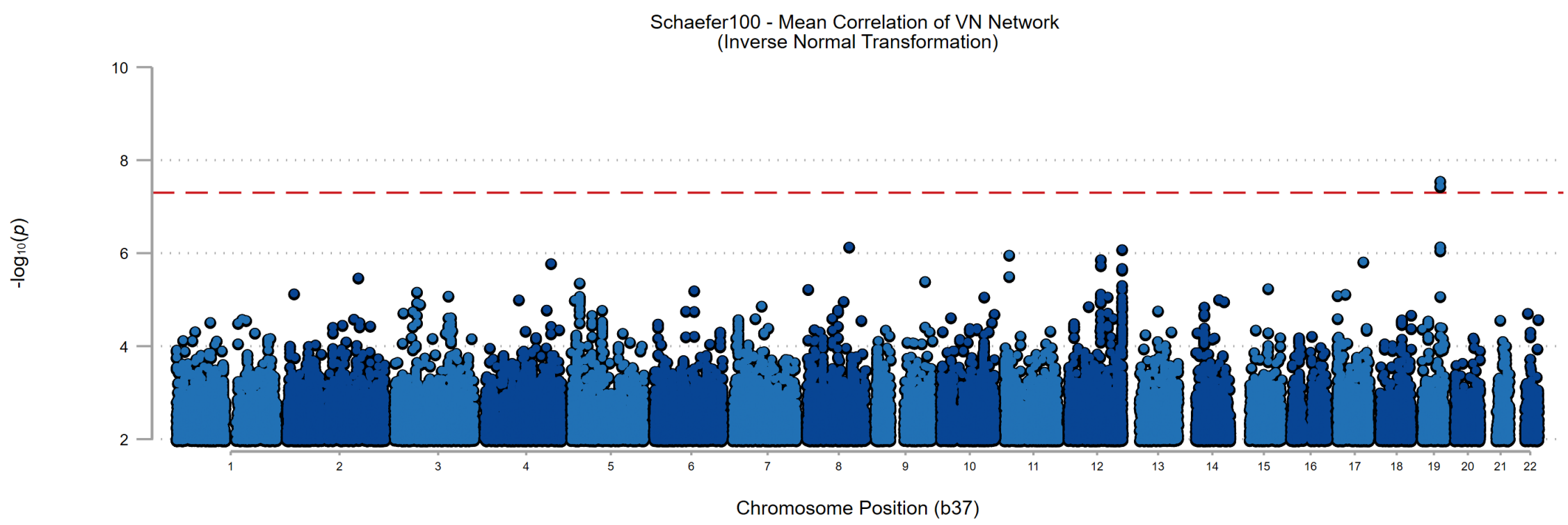
Schaefer100 - Mean Correlation of VN Network
(Inverse Normal Transformation)
-log10(p)
10
8
6
4
2
1 2 3 4 5 6 7 8 9 10 11 12 13 14 15 16 17 18 19 20 21 22
Chromosome Position (b37)

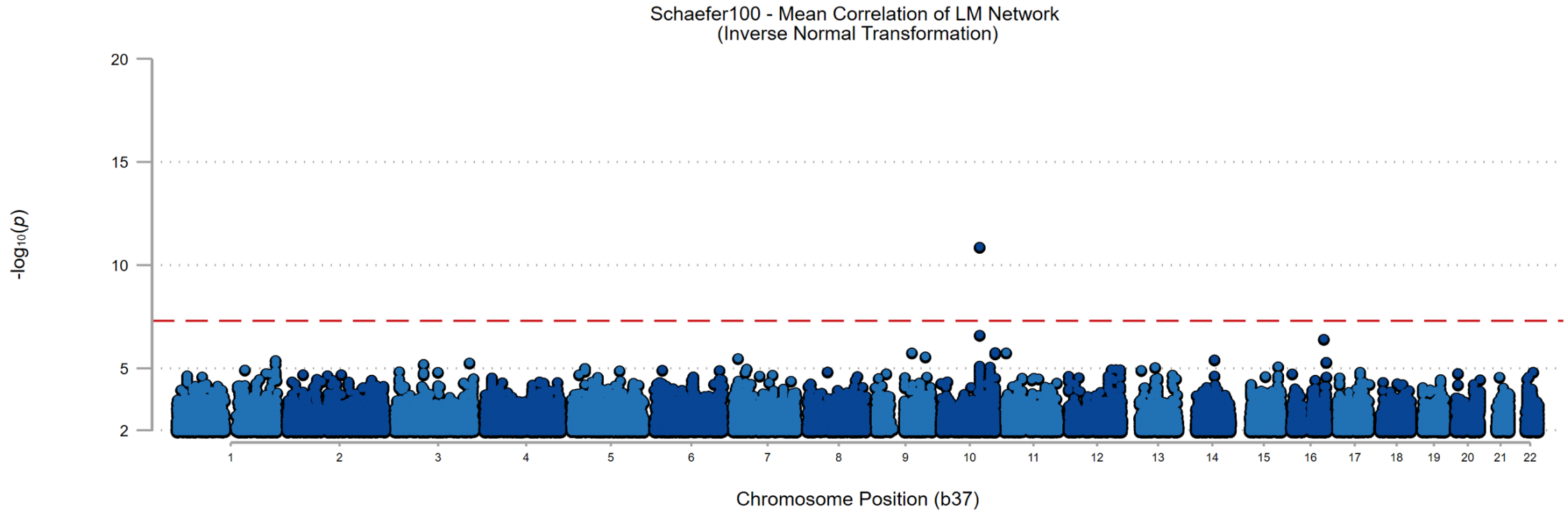

Supplementary Figure 2. Manhattan plots for each rfMRI network resulting from a univariate GWAS of the average rfMRI connectivity (mean partial correlation) on the same sample than our main multivariate GWAS approach. The exact same analytica pre-GWAS steps were followed, but instead of entering each edge within the network into a MOSTest GWAS, the mean of all edges within each network was entered into a univariate GWAS. Source data for this figure can be obtained from https://git.cardiff.ac.uk/imagenes/

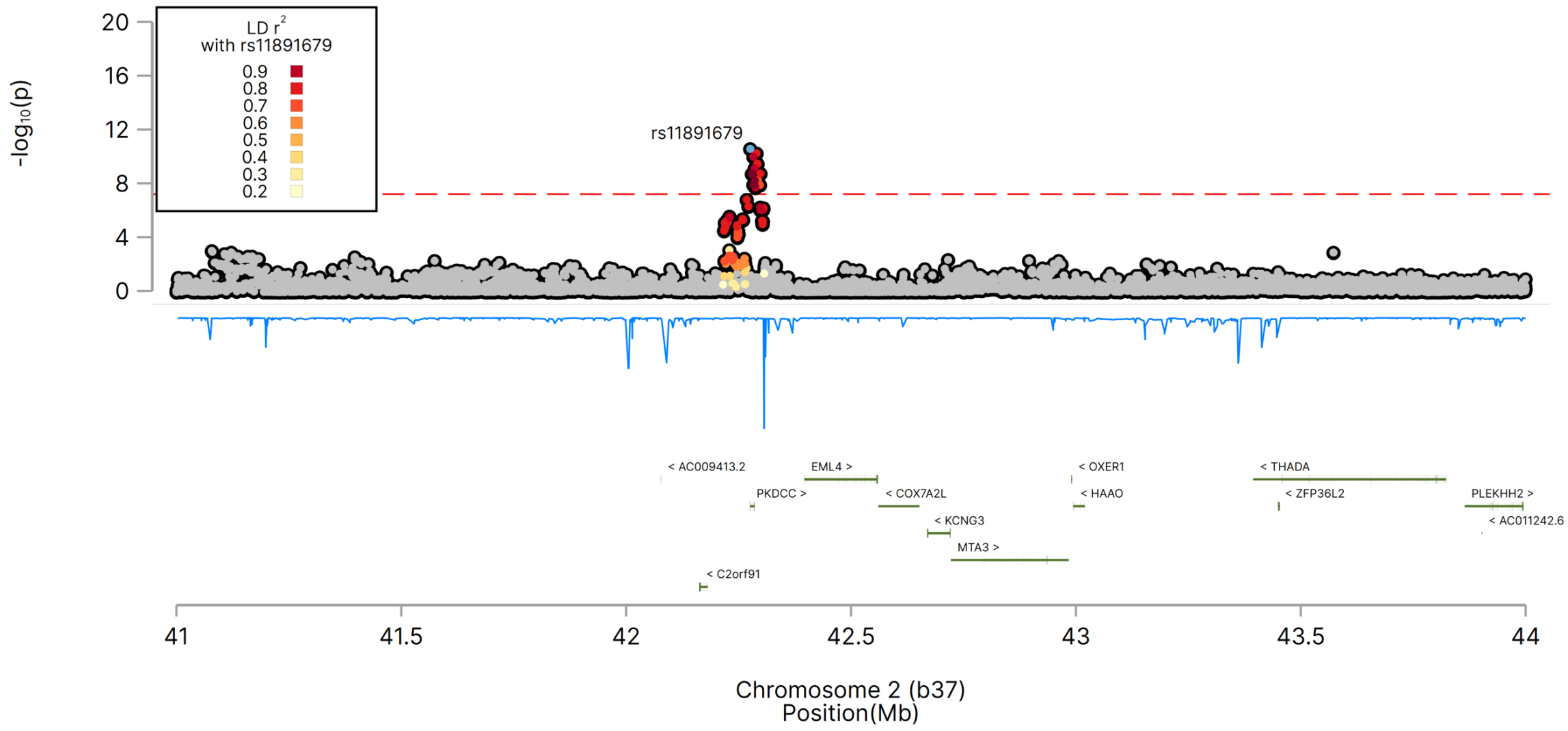

dataset: ukbb-fmri-rsn-DAN

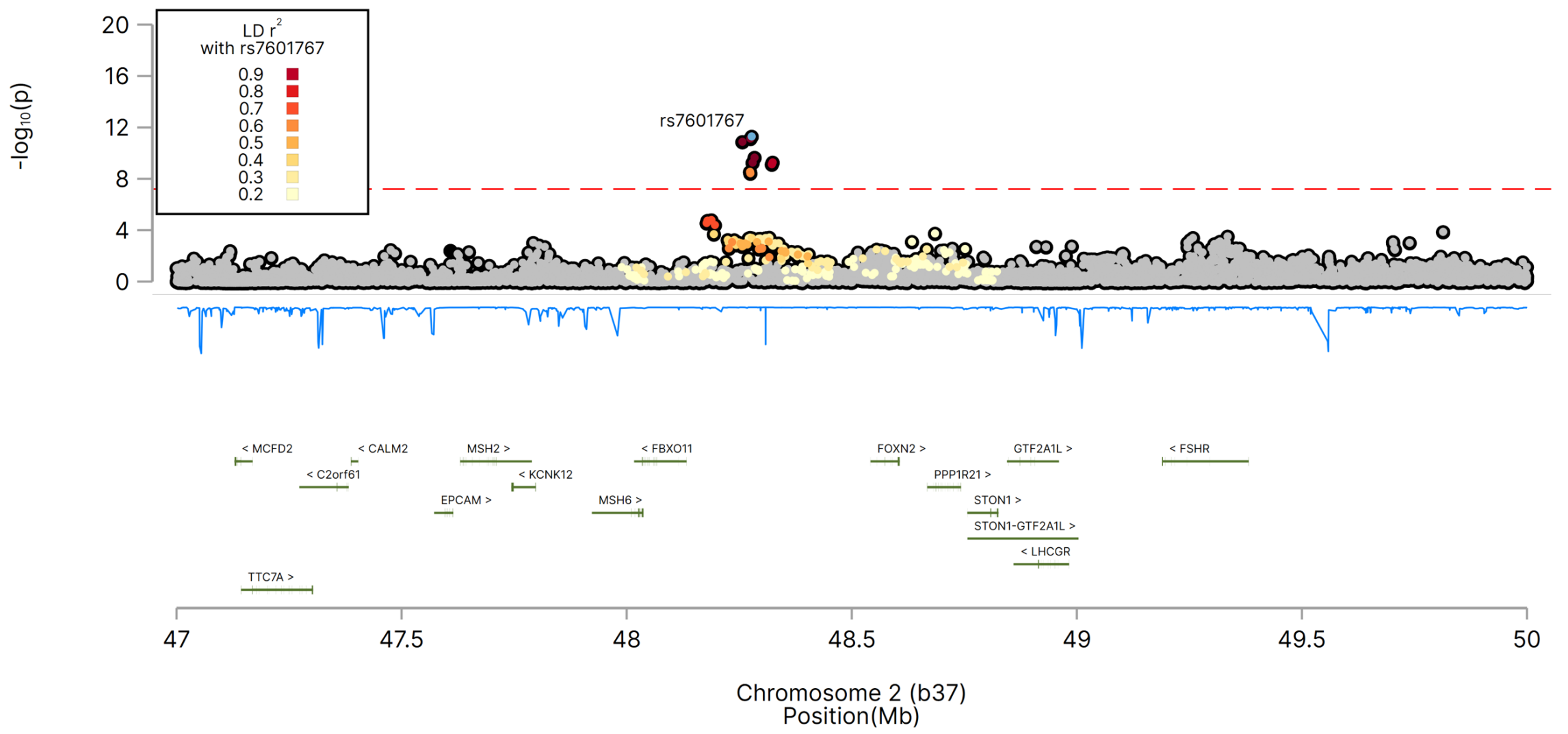

dataset: ukbb-fmri-rsn-DAN

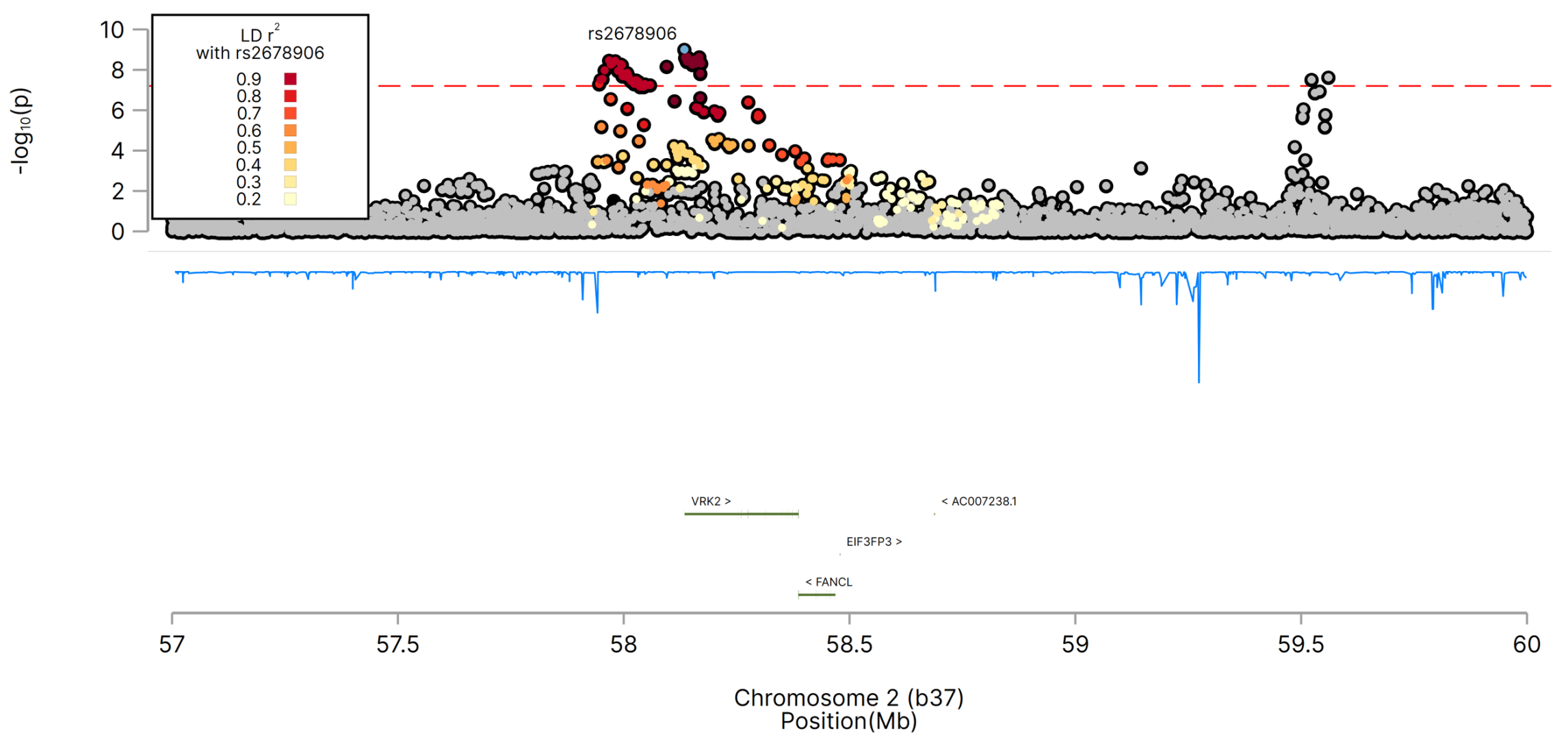

dataset: ukbb-fmri-rsn-DAN

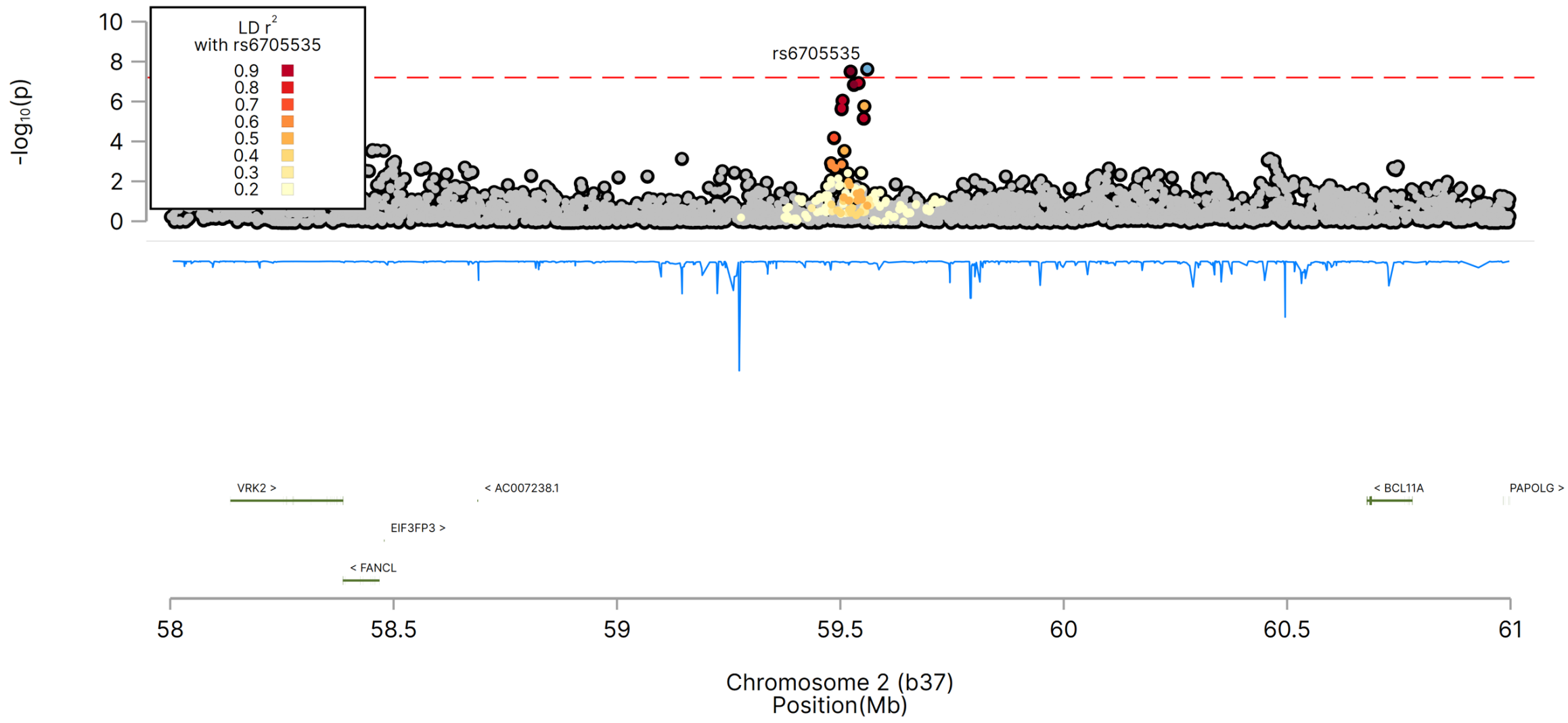

dataset: ukbb-fmri-rsn-DAN

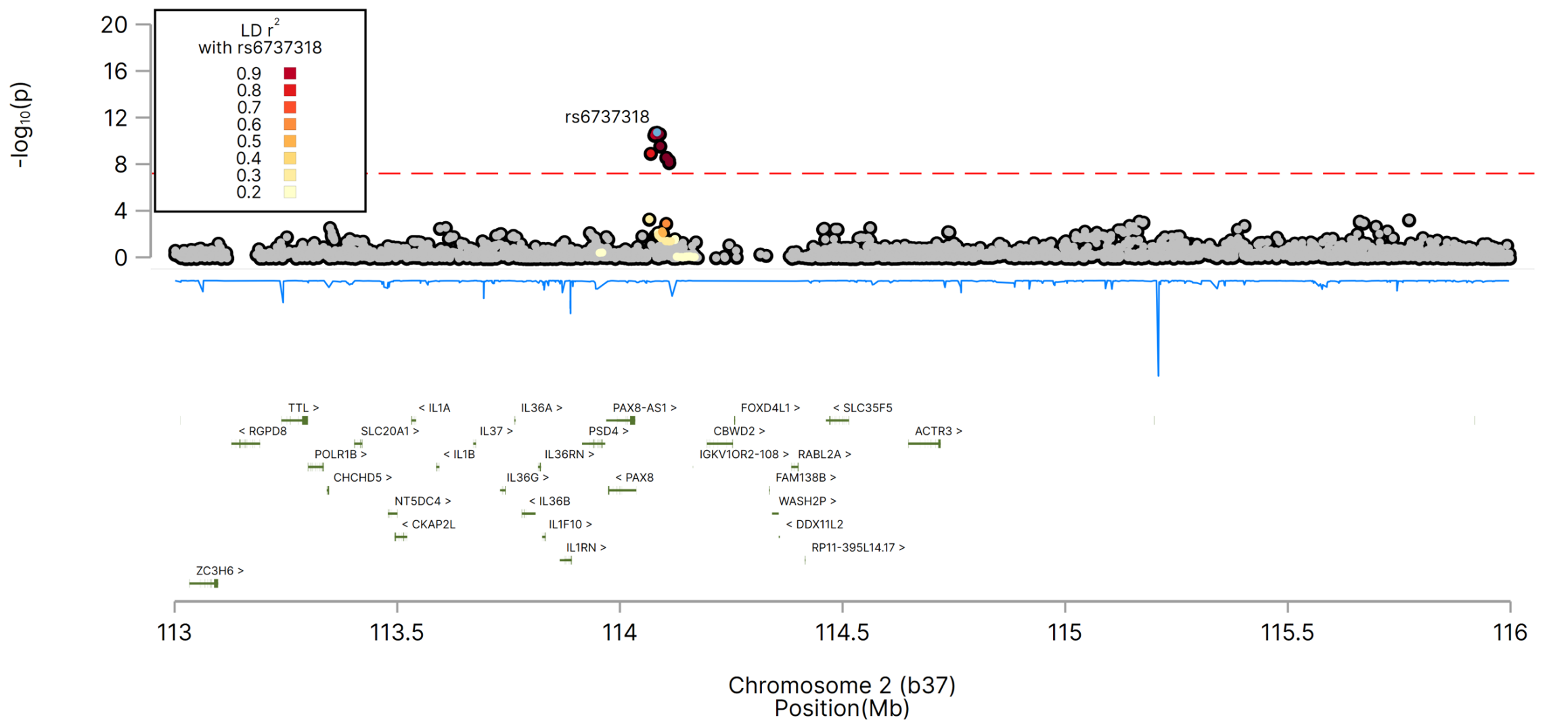

dataset: ukbb-fmri-rsn-DAN

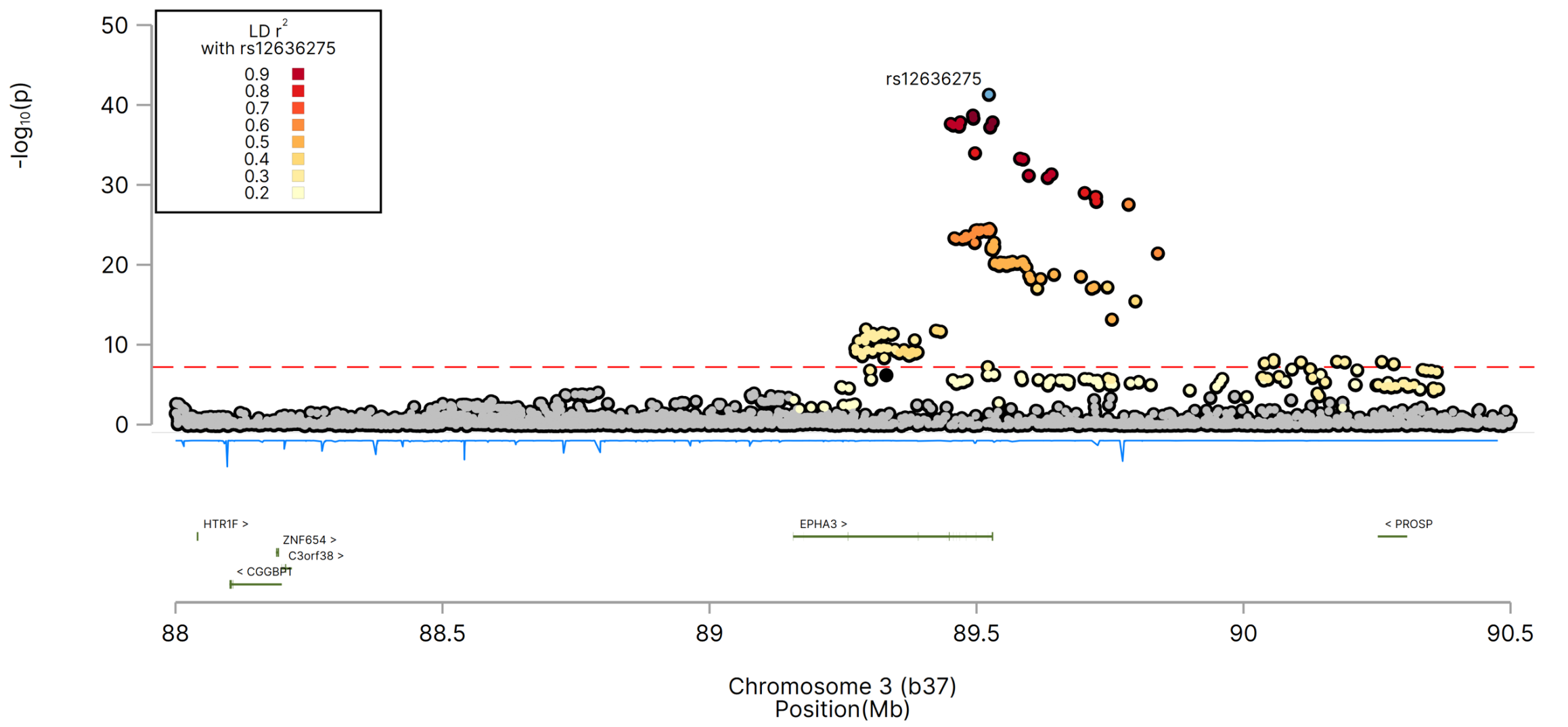

dataset: ukbb-fmri-rsn-DAN

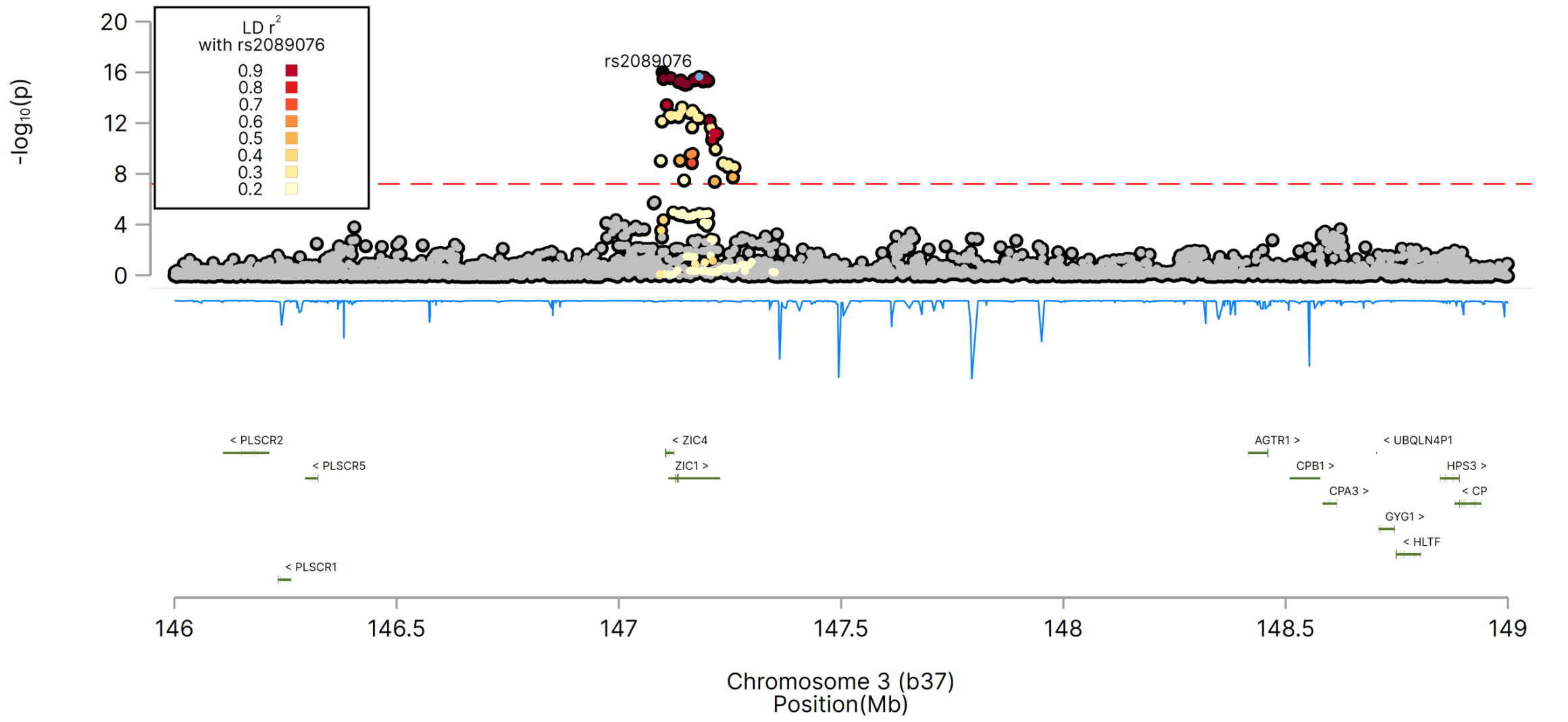

dataset: ukbb-fmri-rsn-DAN

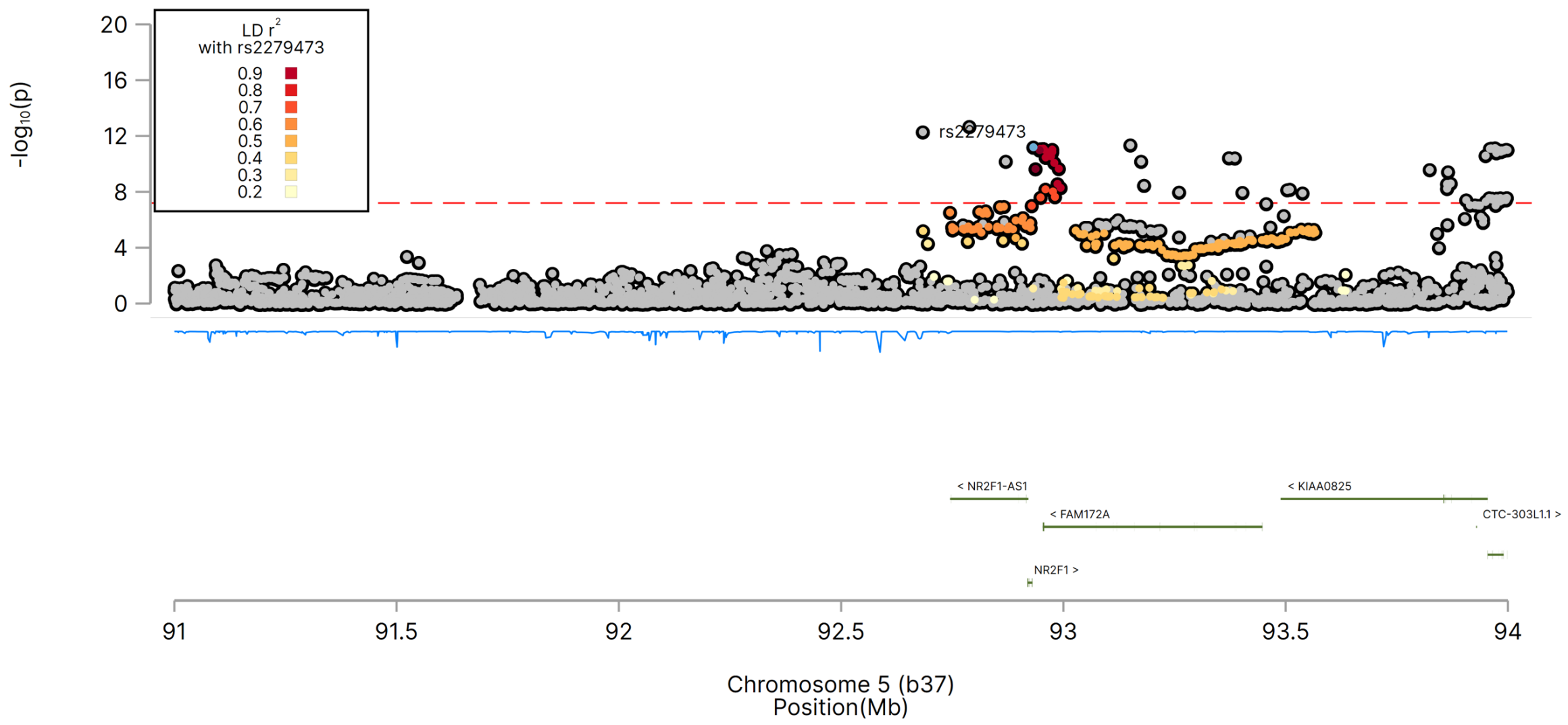

dataset: ukbb-fmri-rsn-DAN

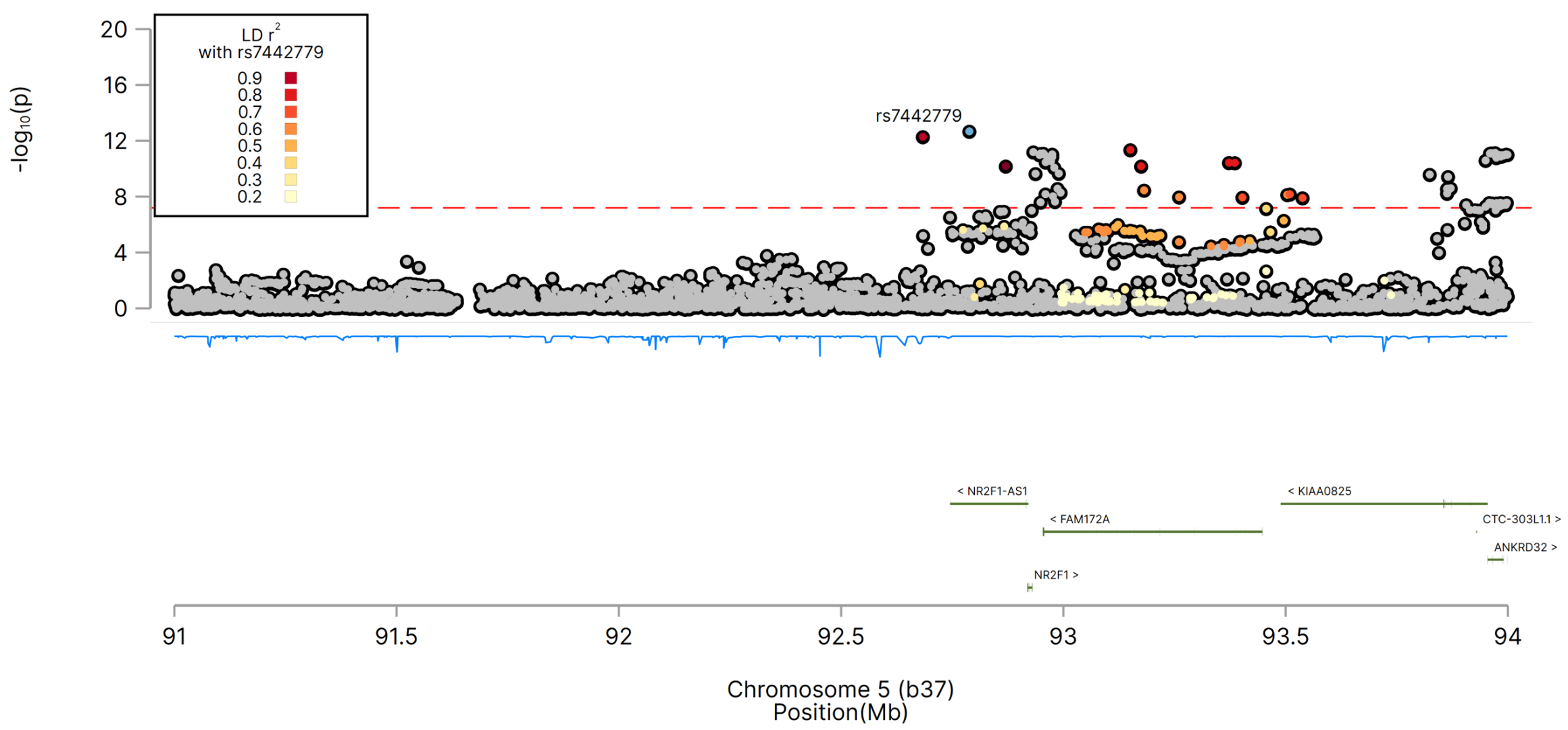

dataset: ukbb-fmri-rsn-DAN

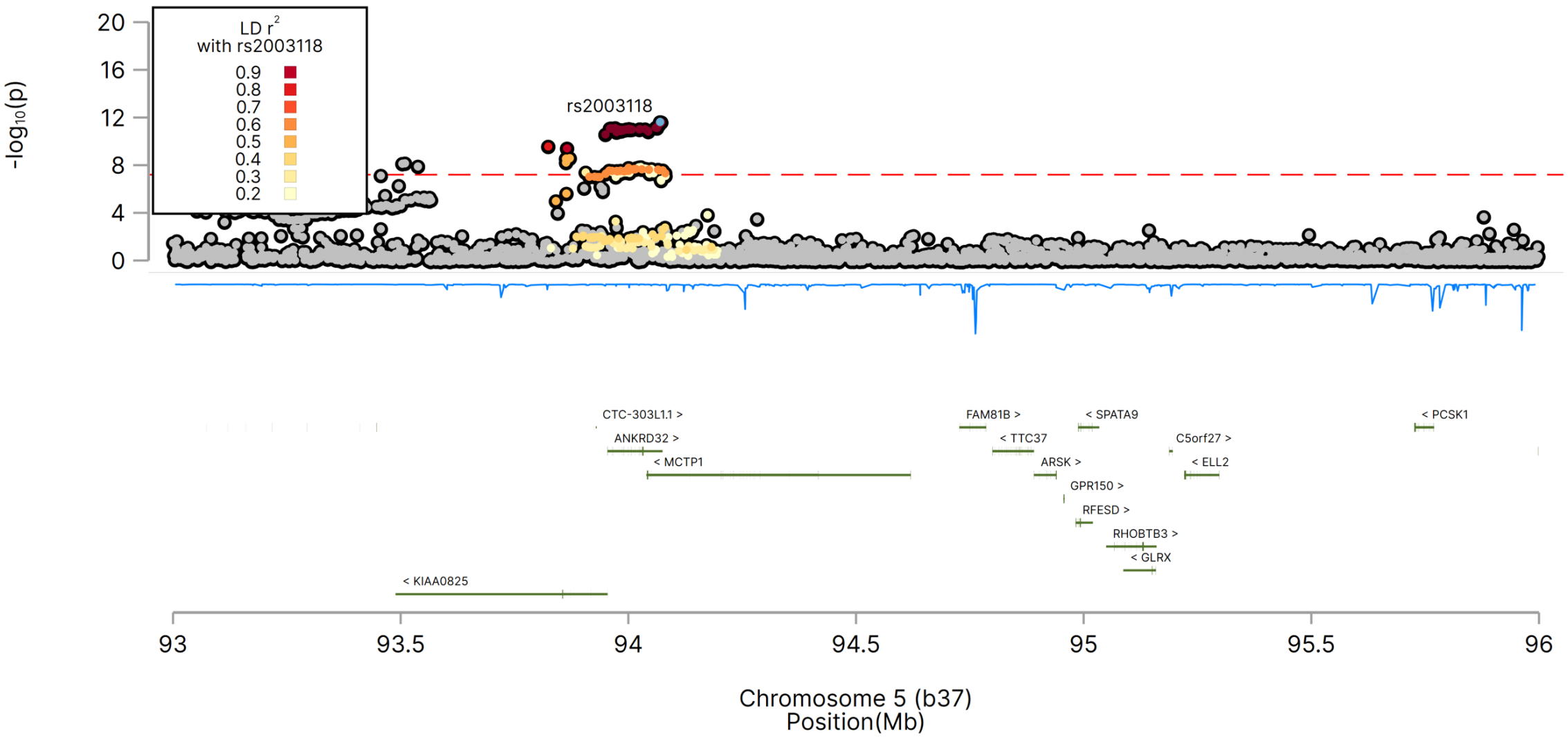

dataset: ukbb-fmri-rsn-DAN

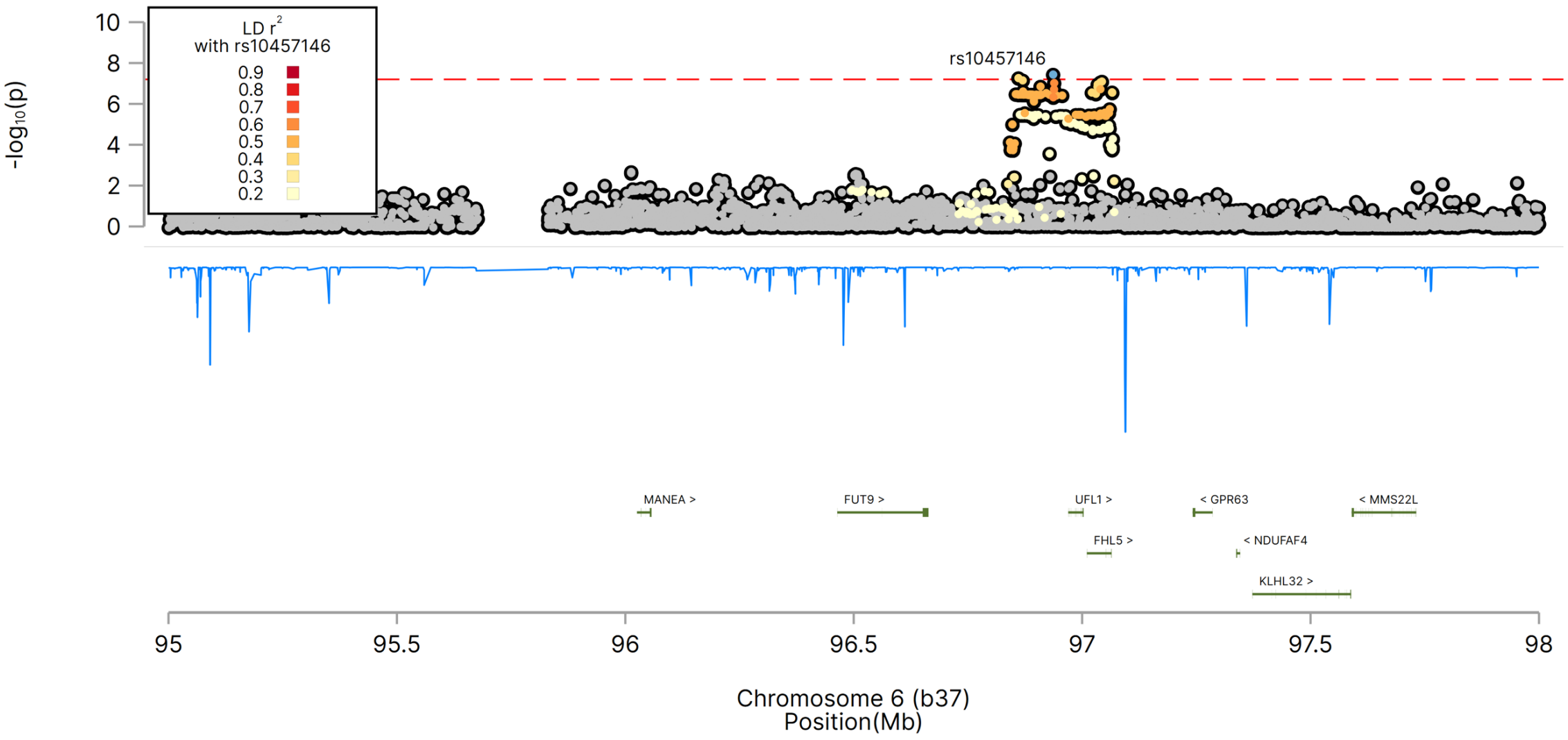

dataset: ukbb-fmri-rsn-DAN

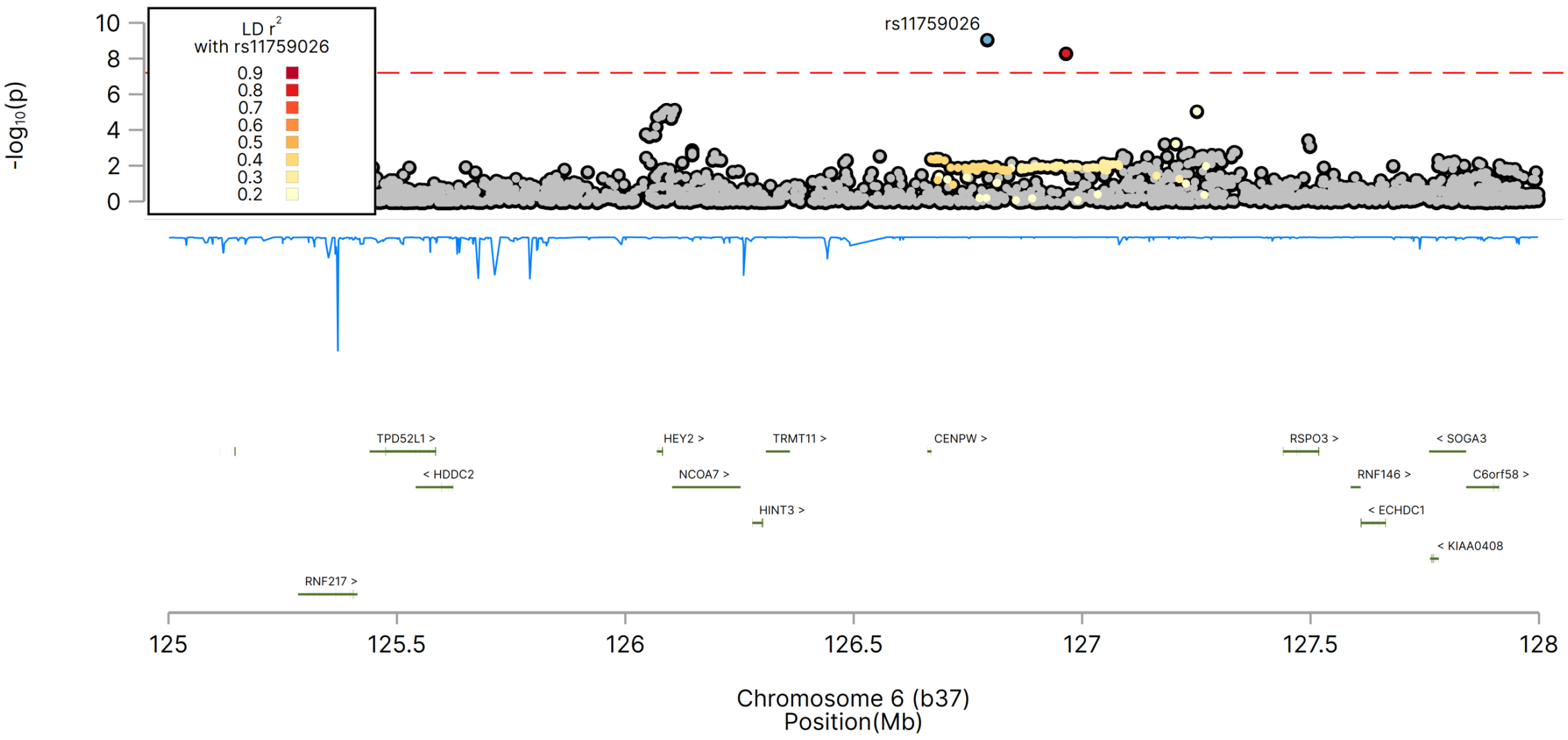

dataset: ukbb-fmri-rsn-DAN

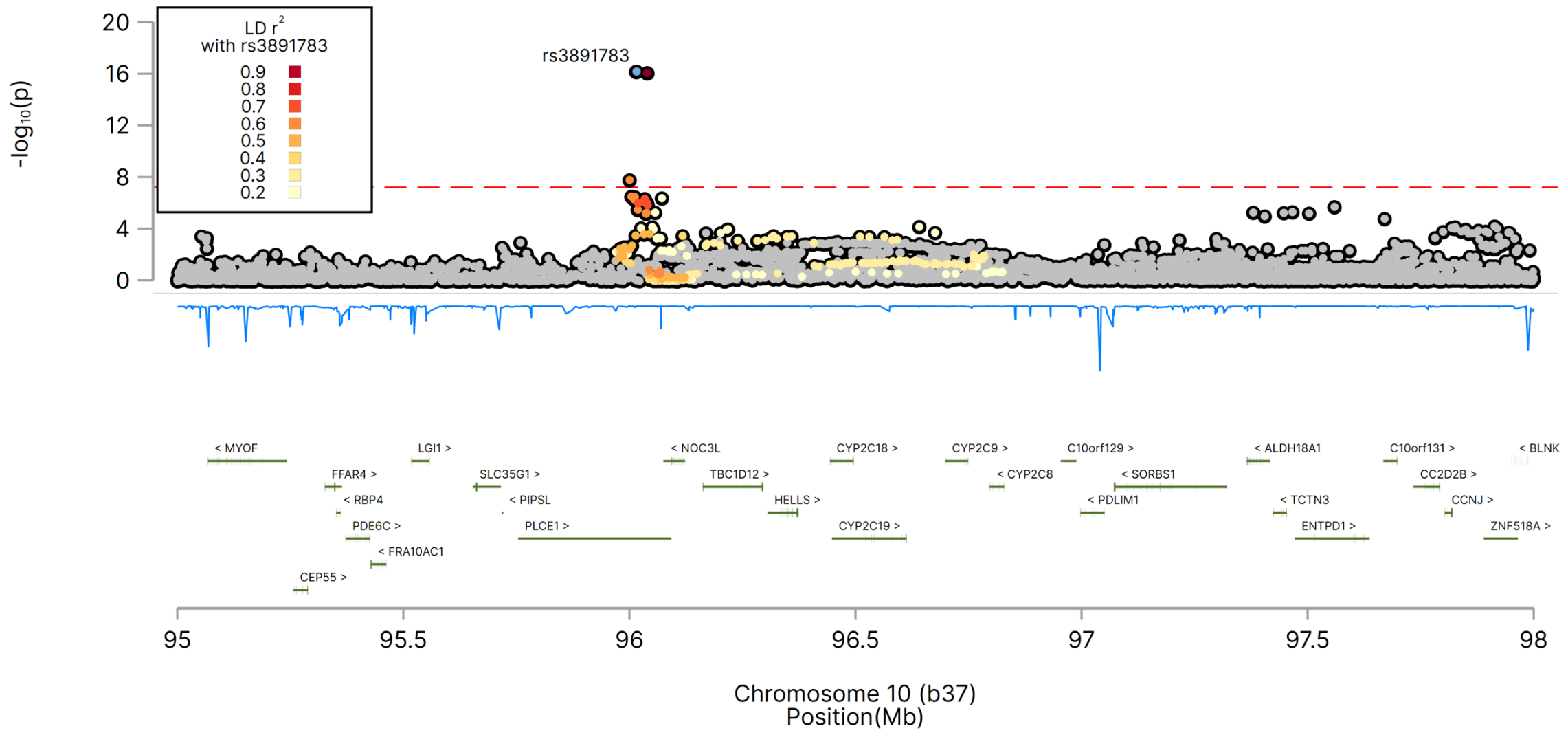

dataset: ukbb-fmri-rsn-DAN

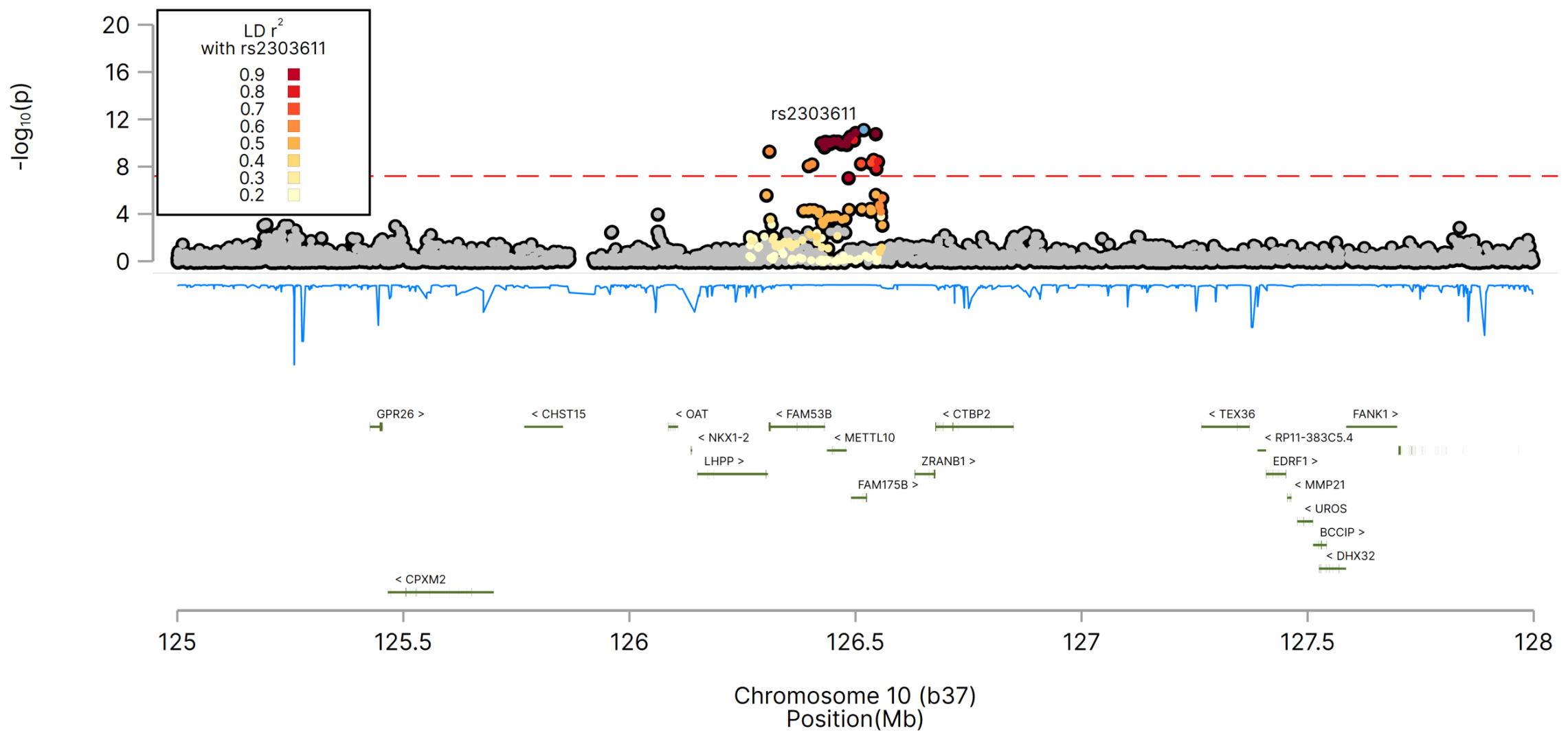

dataset: ukbb-fmri-rsn-DAN

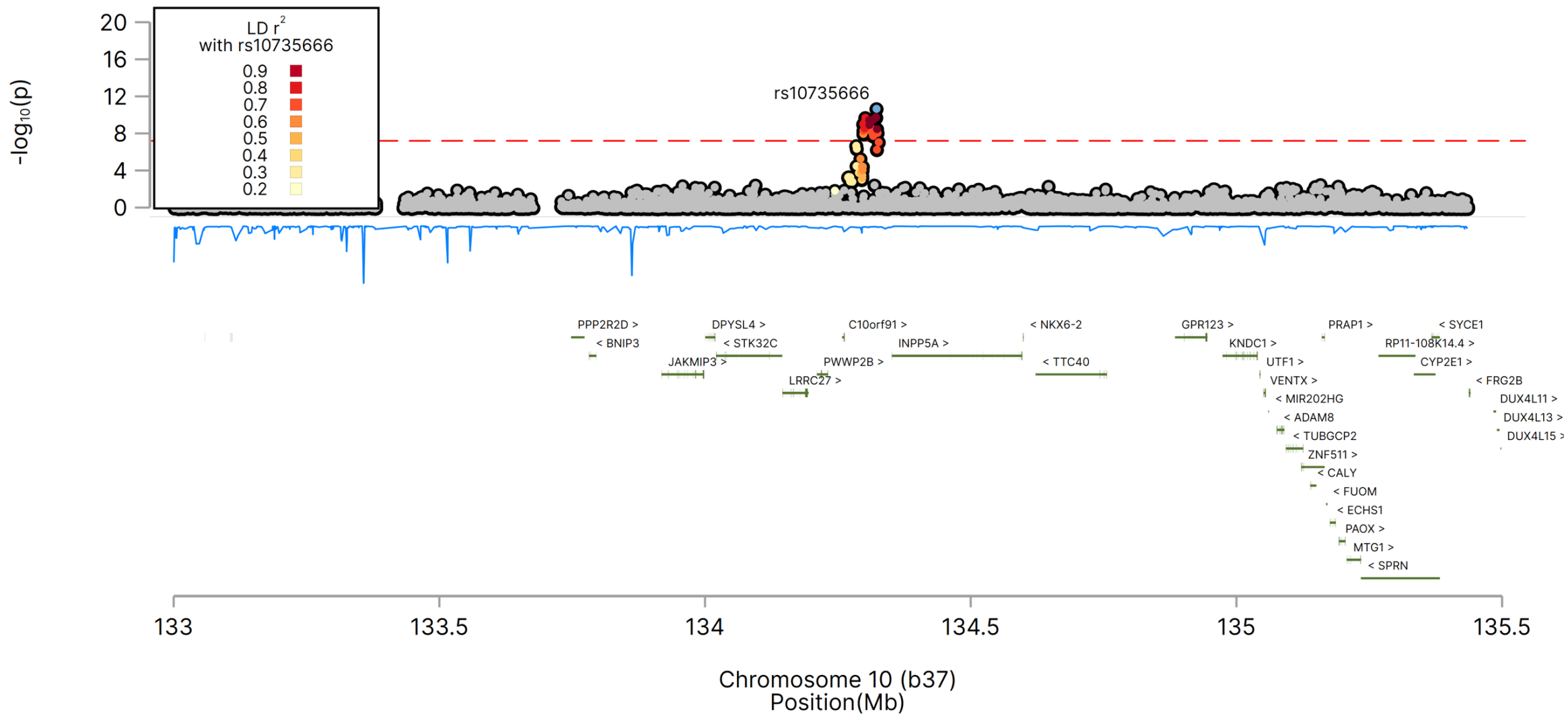

dataset: ukbb-fmri-rsn-DAN

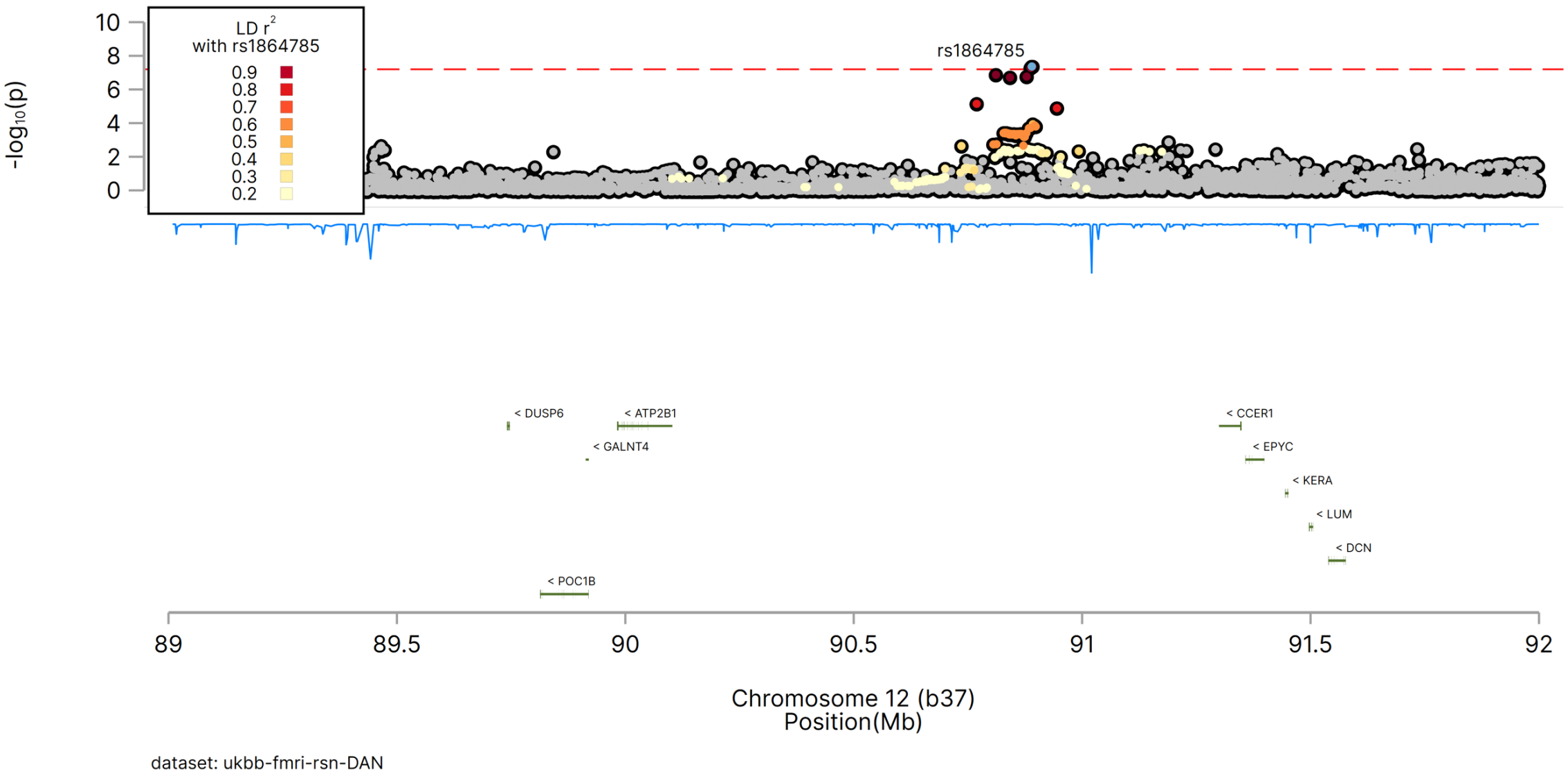

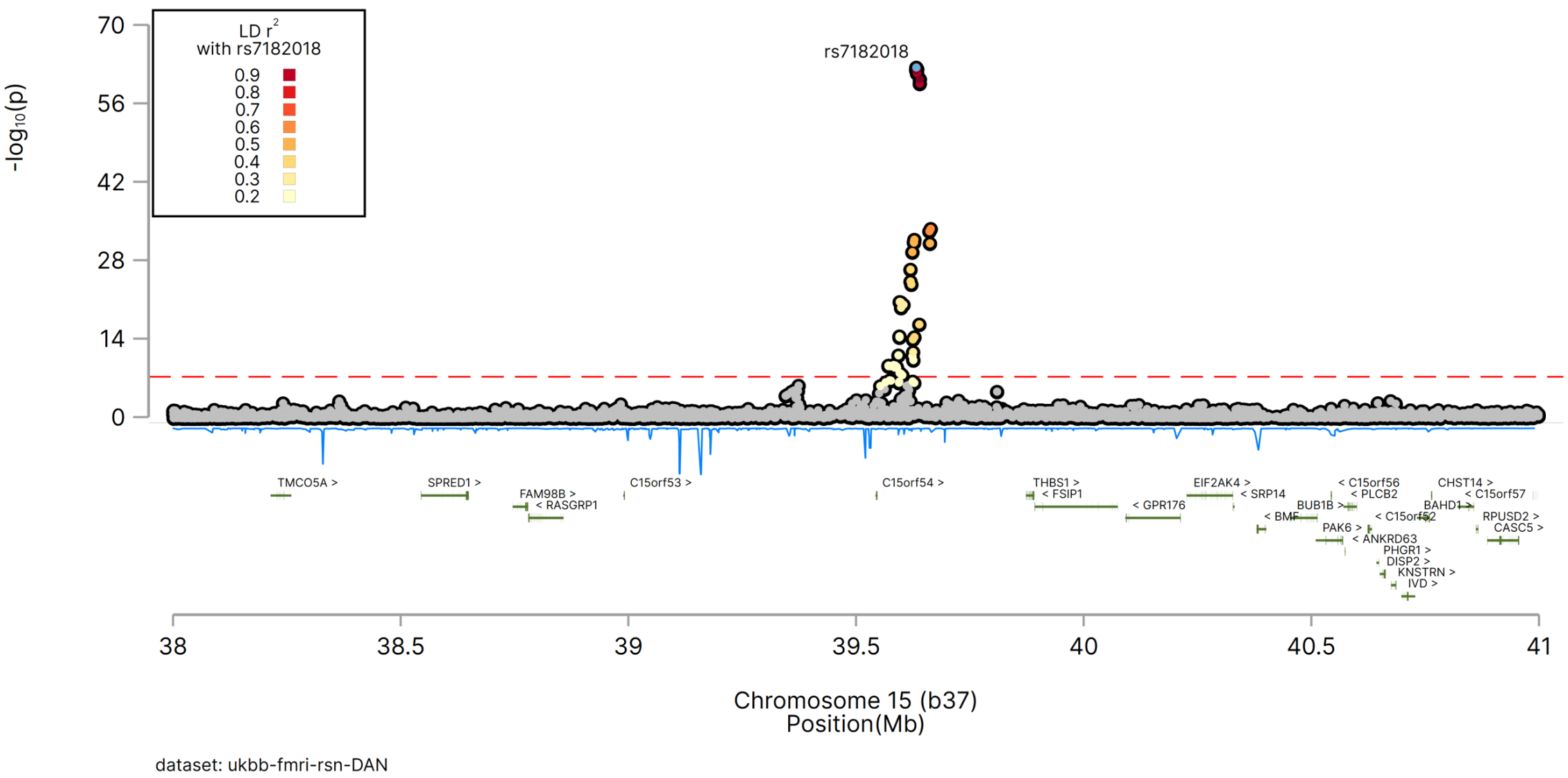

Supplementary Figure 3. Locus-zoom for all significant LD-independent signals coming from the MOST GWAS test for the Dorsal Attention Network (DAN). Source data for this figure can be obtained from https://www.ebi.ac.uk/gwas/; hyperlinks to the data for each network are provided in the Data Availability section.

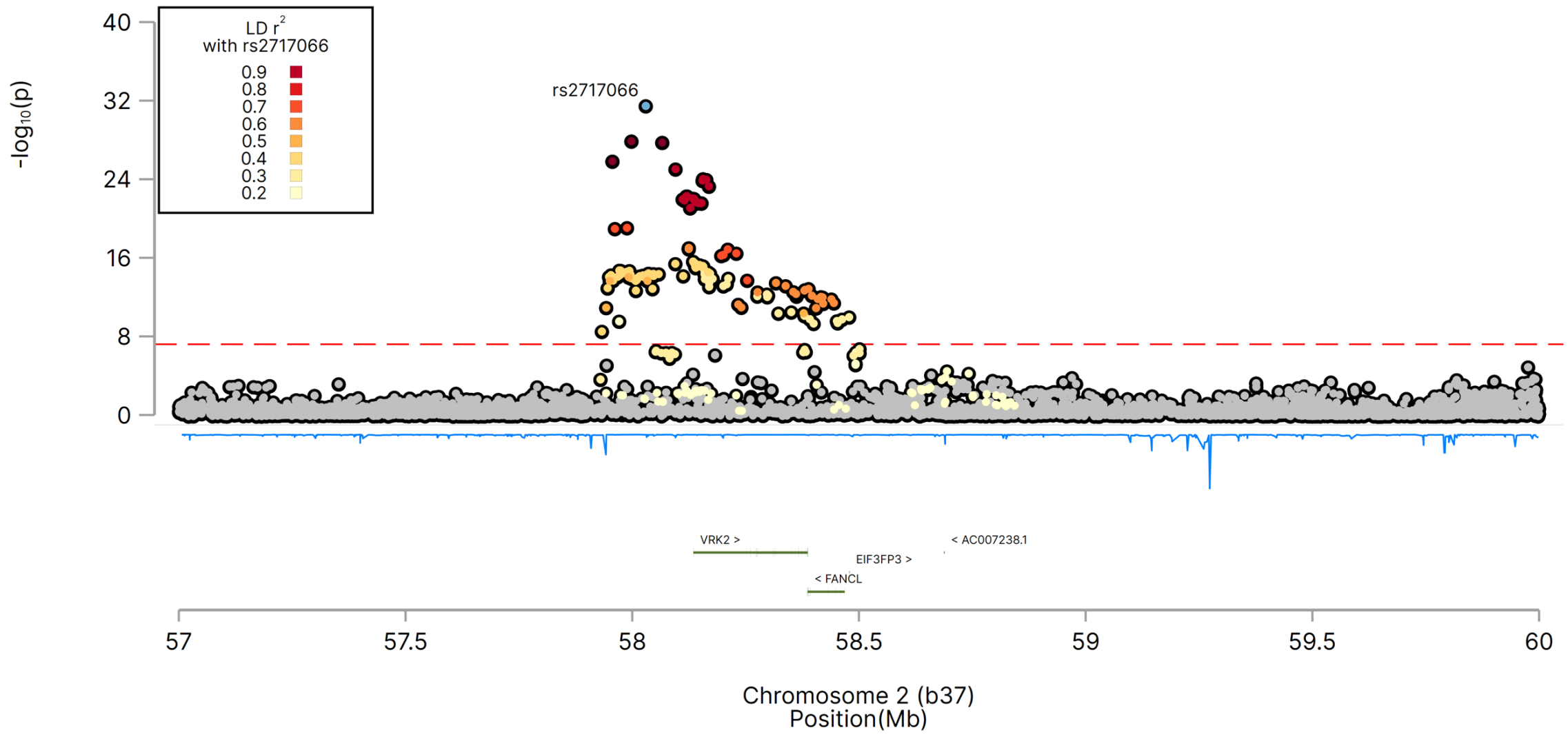

dataset: ukbb-fmri-rsn-DMN

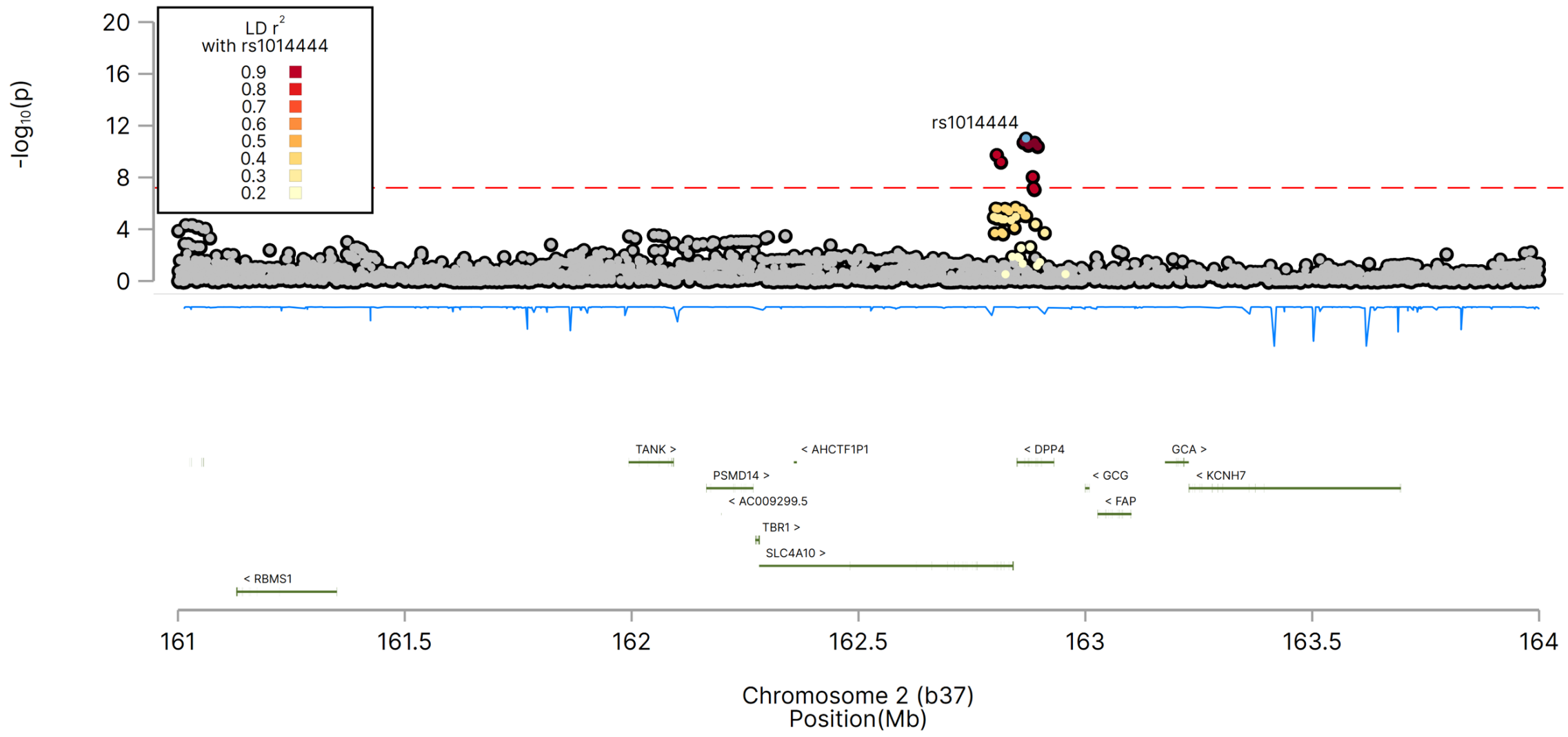

dataset: ukbb-fmri-rsn-DMN

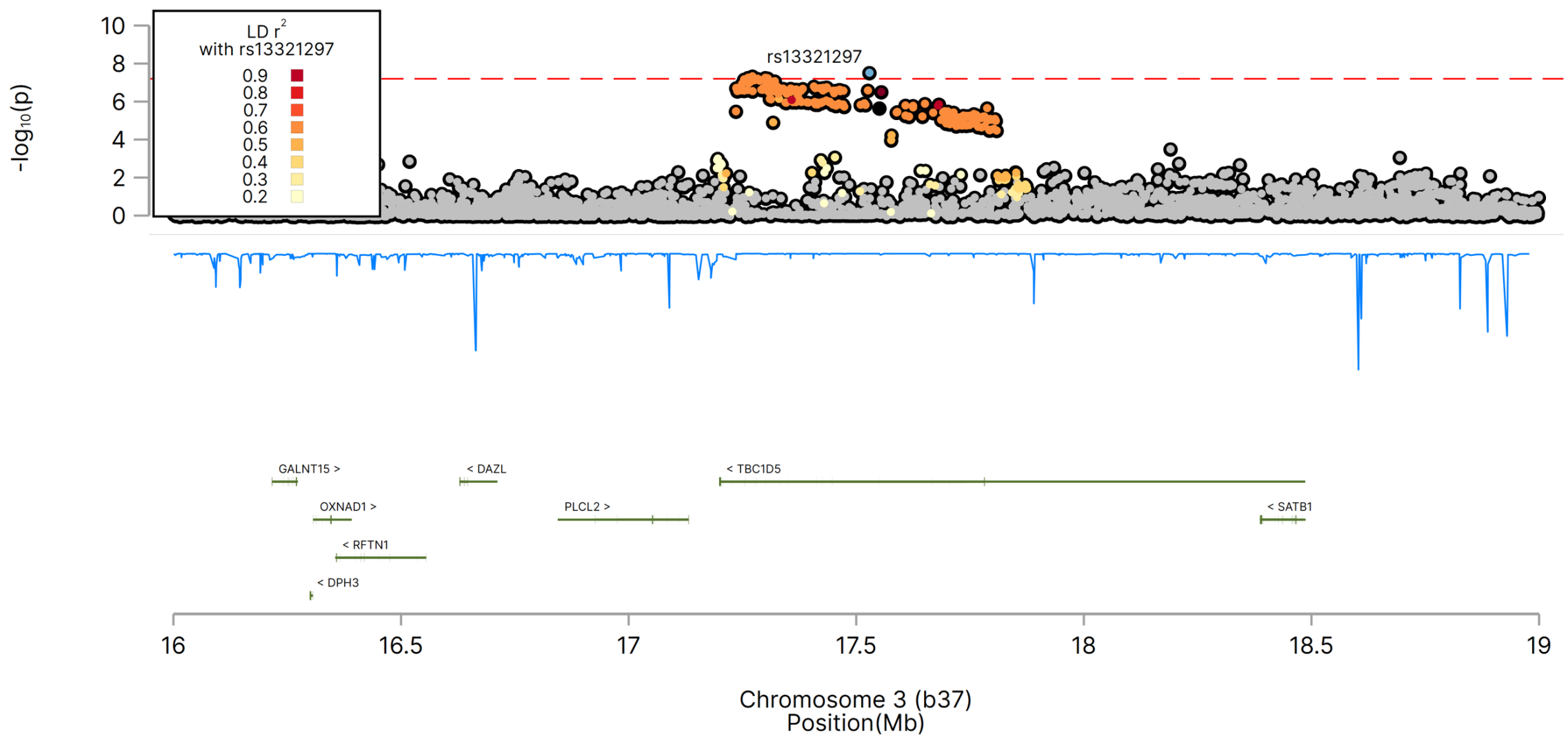

dataset: ukbb-fmri-rsn-DMN

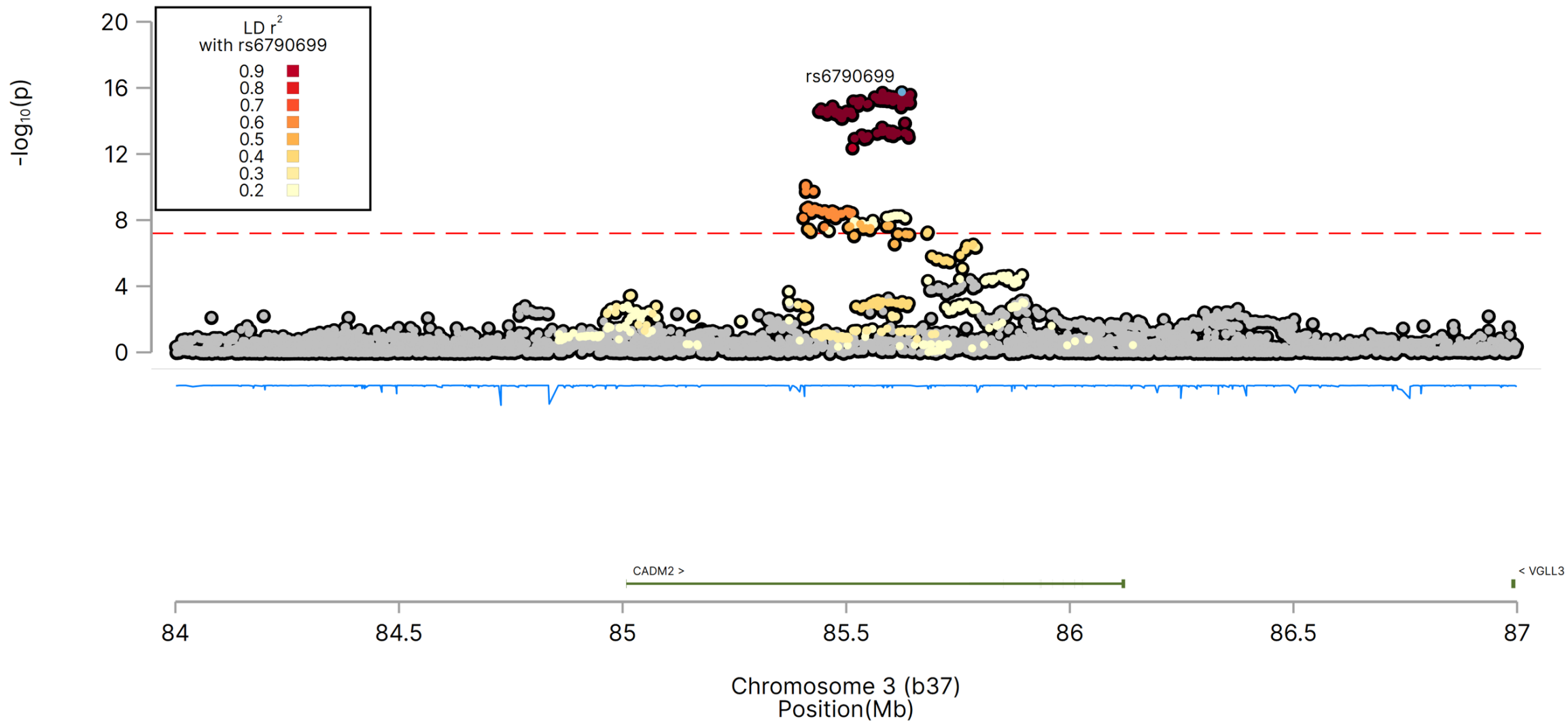

dataset: ukbb-fmri-rsn-DMN

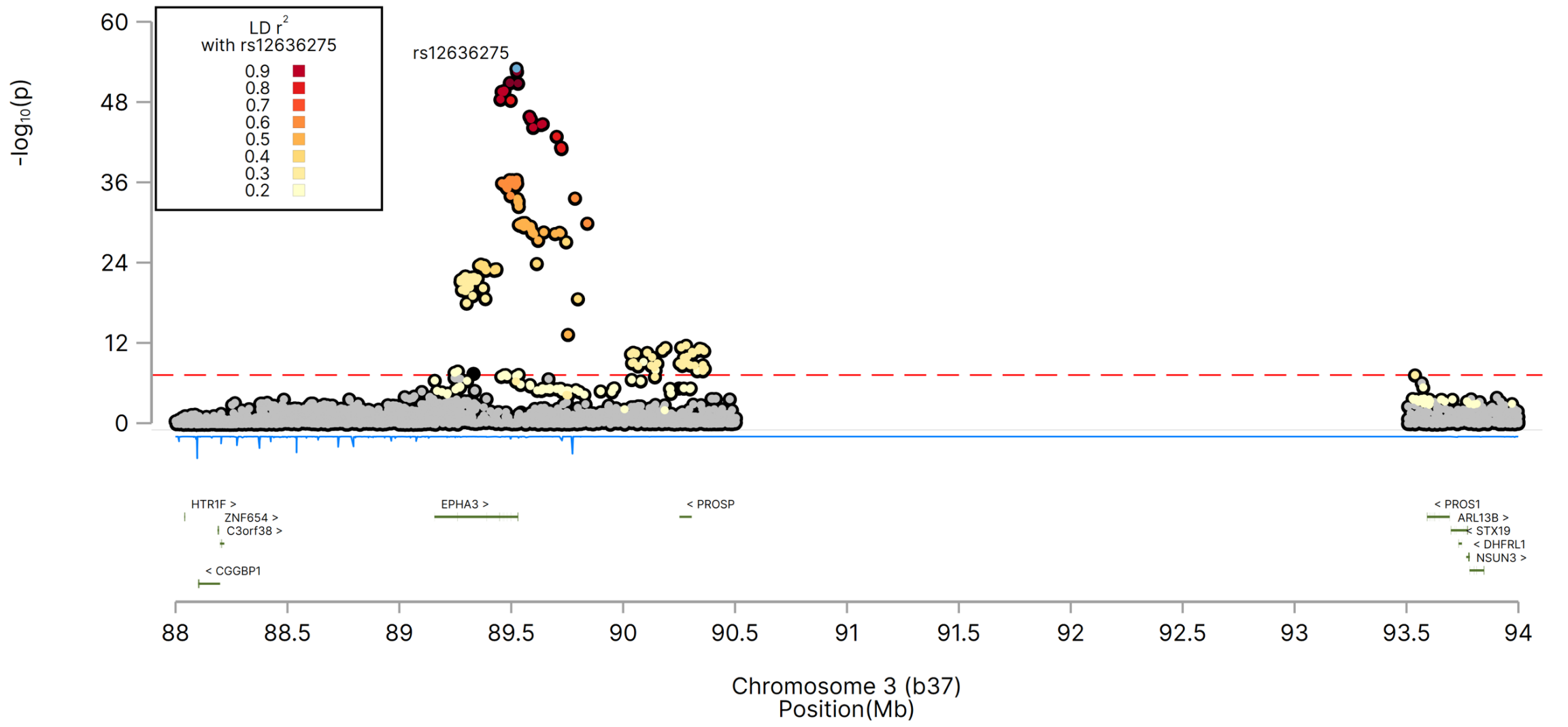

dataset: ukbb-fmri-rsn-DMN

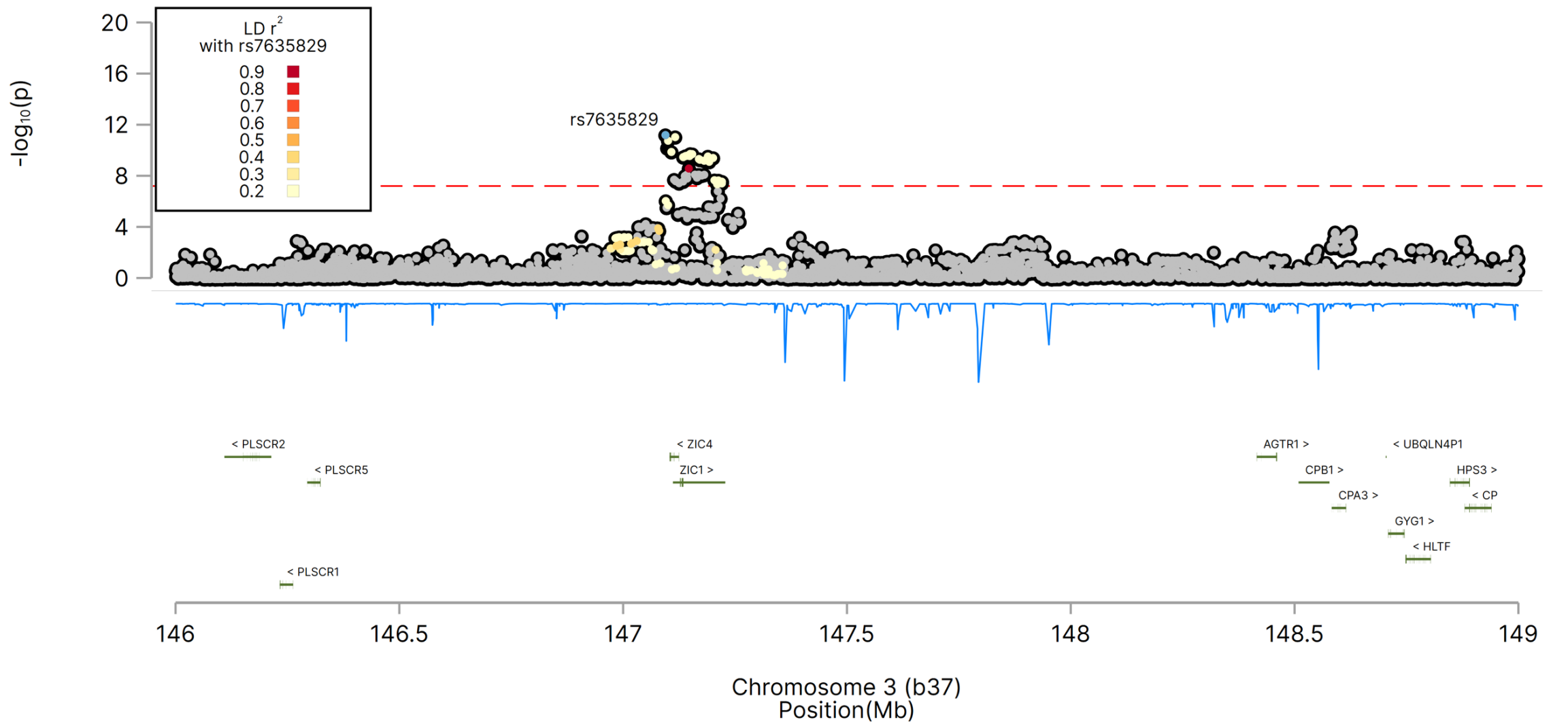

dataset: ukbb-fmri-rsn-DMN

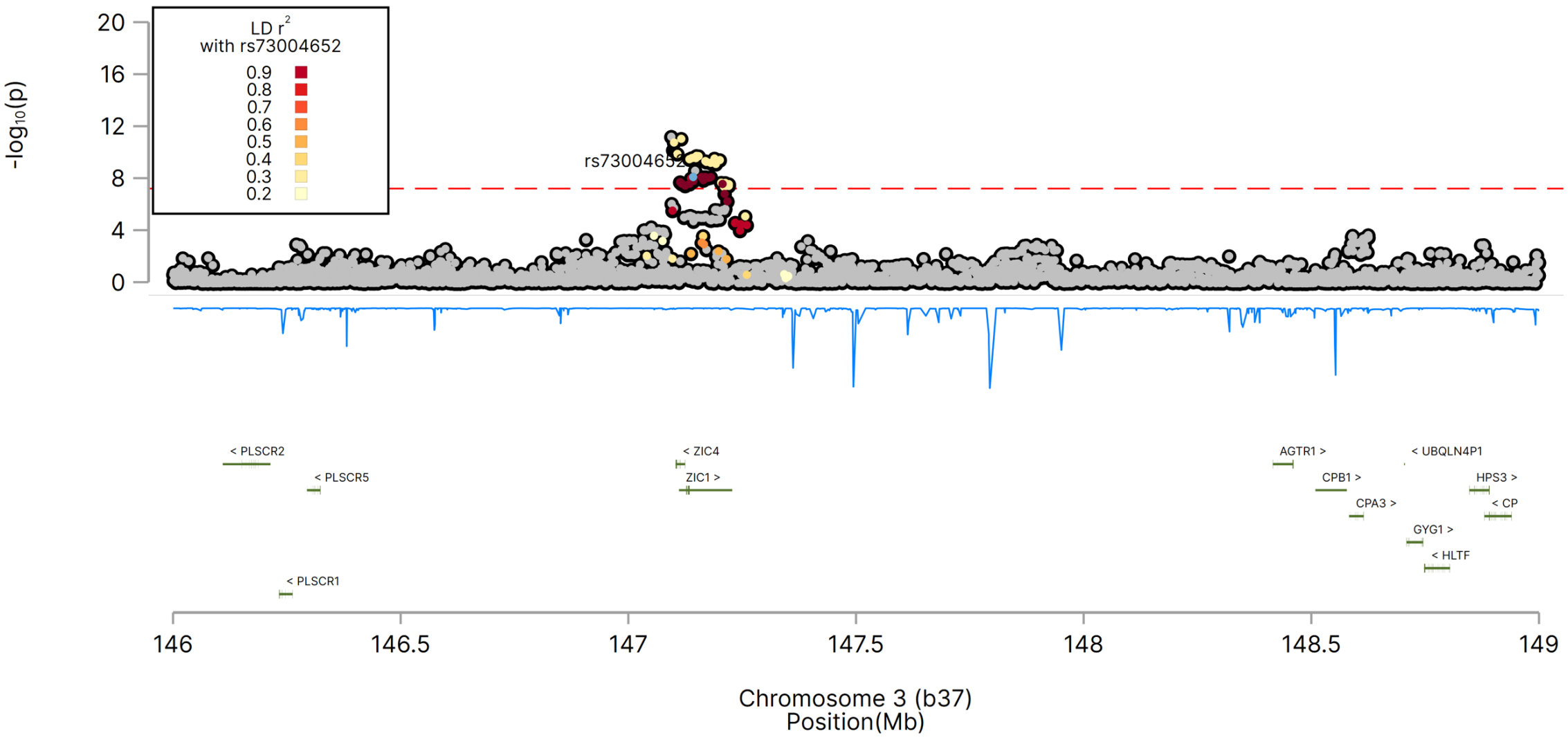

dataset: ukbb-fmri-rsn-DMN

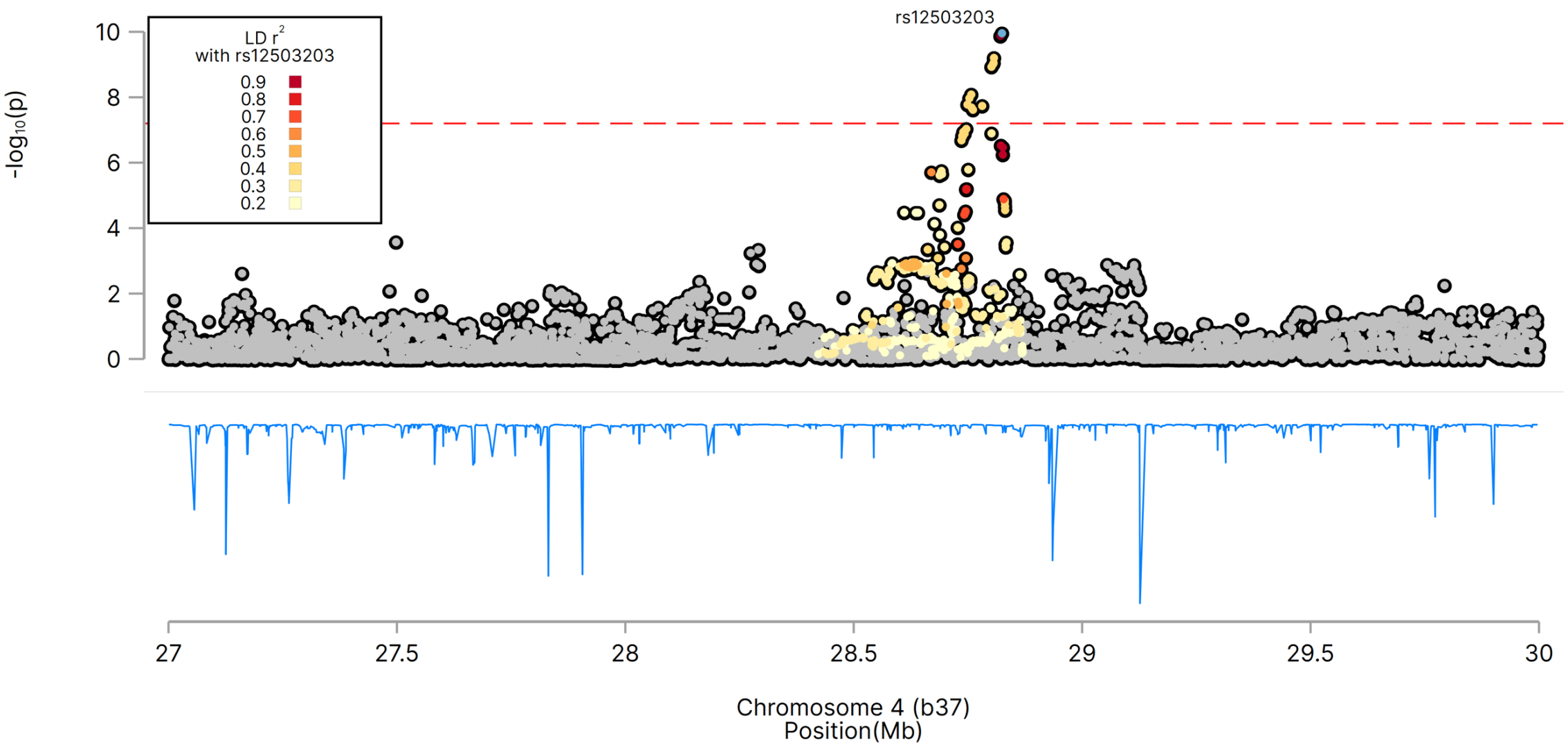

dataset: ukbb-fmri-rsn-DMN

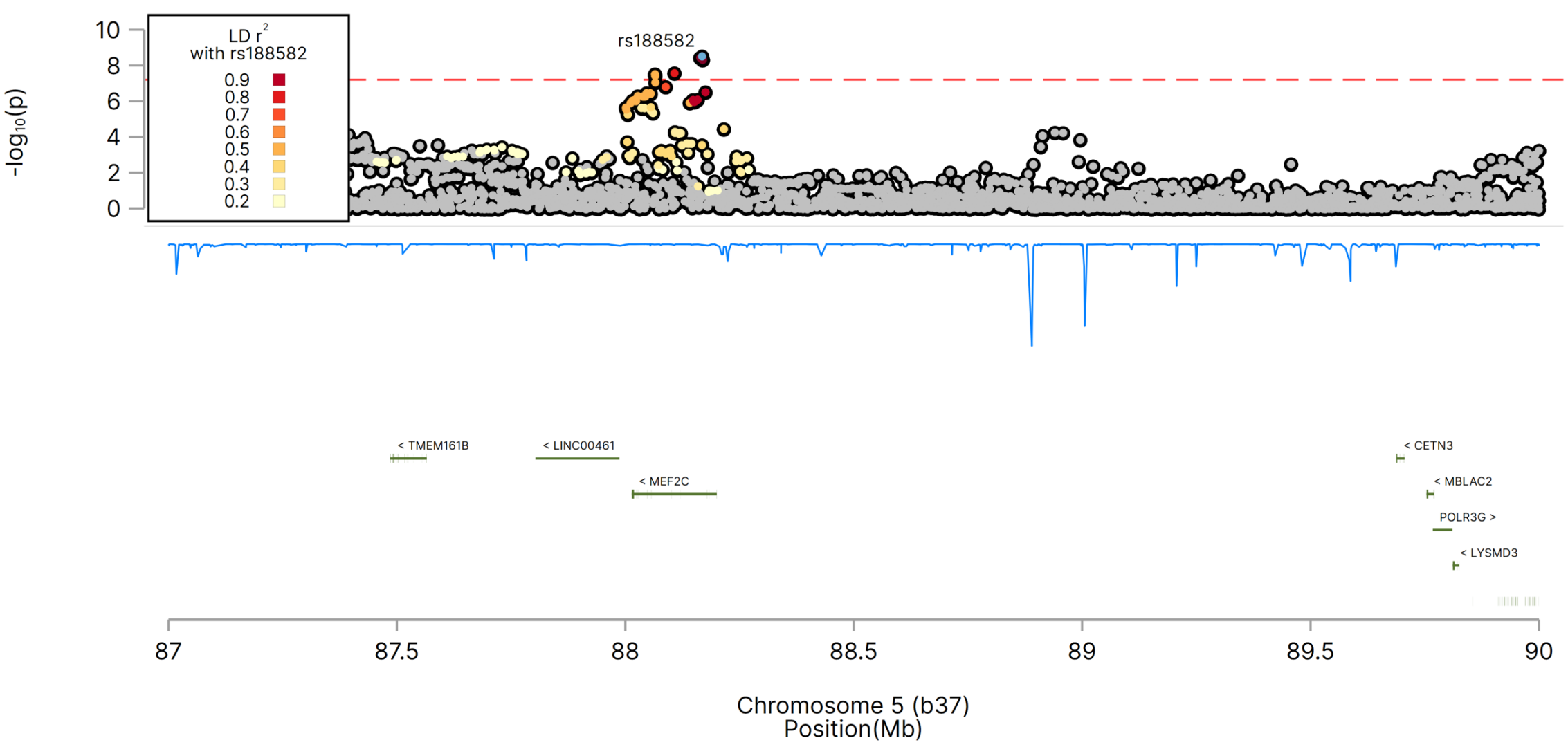

dataset: ukbb-fmri-rsn-DMN

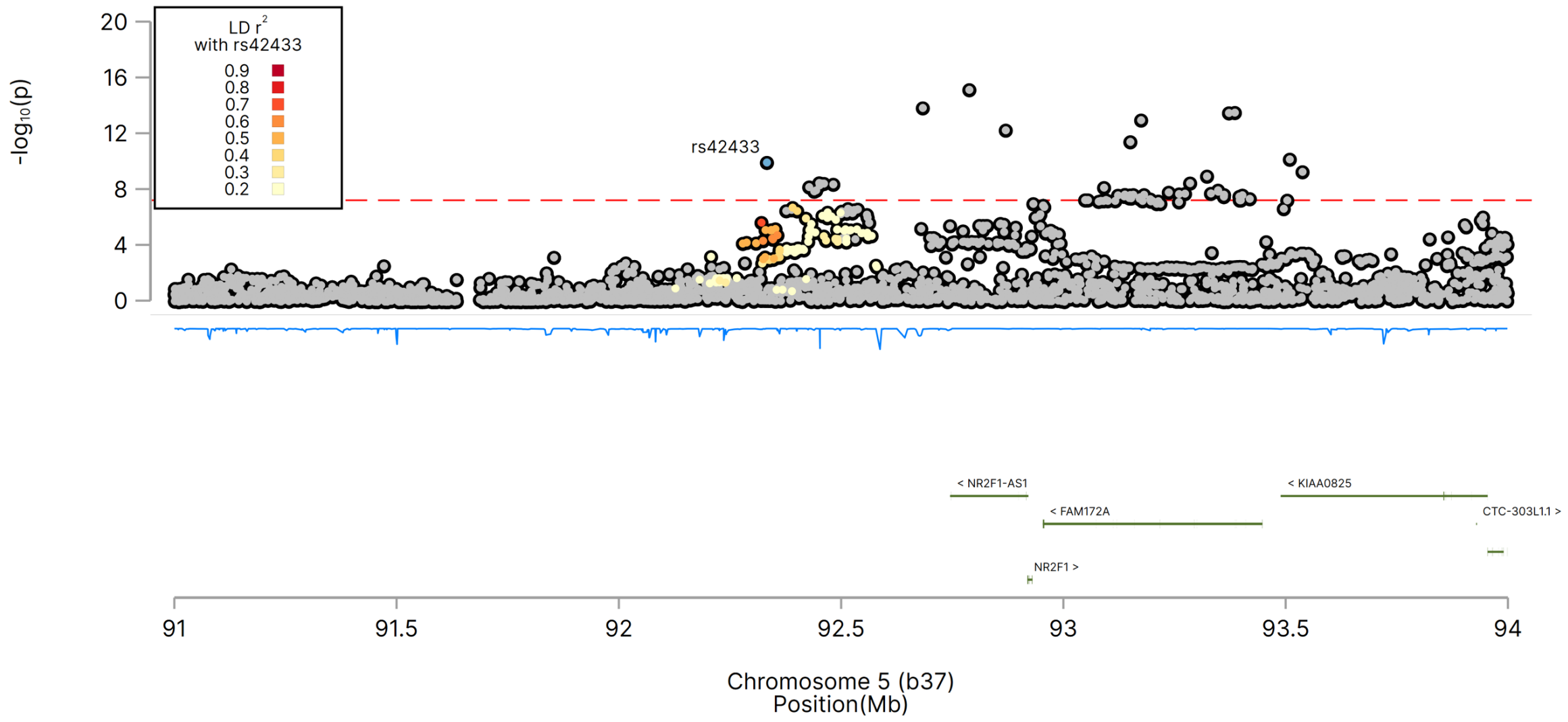

dataset: ukbb-fmri-rsn-DMN

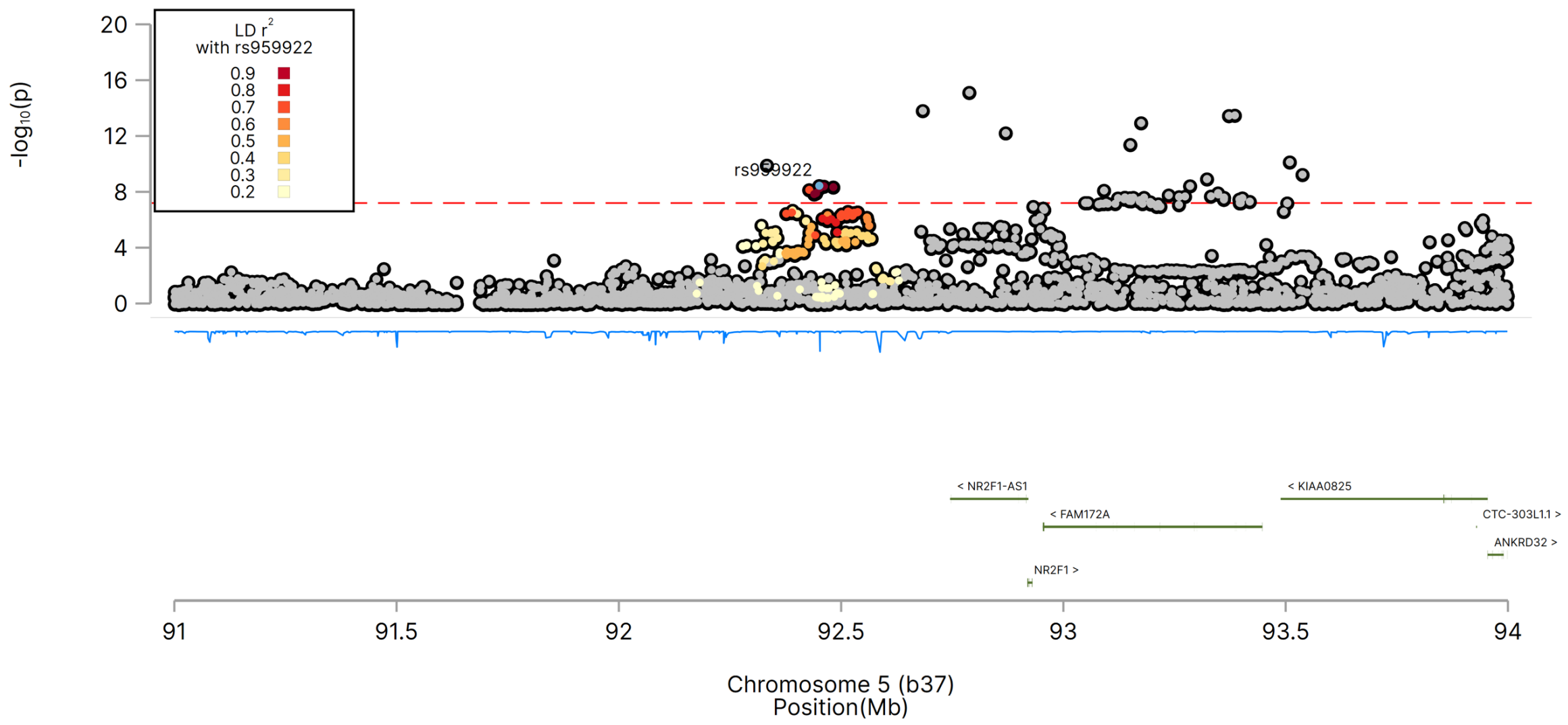

dataset: ukbb-fmri-rsn-DMN

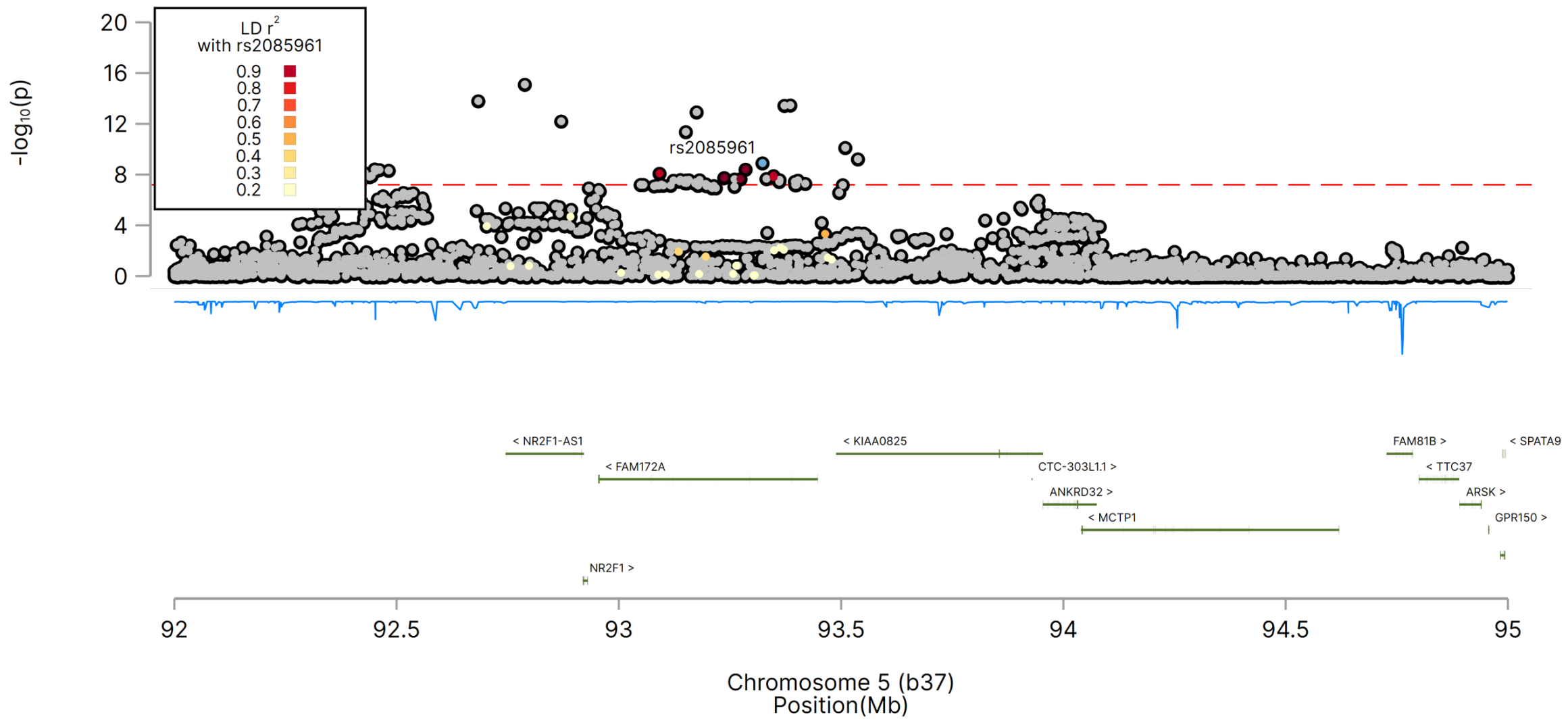

dataset: ukbb-fmri-rsn-DMN

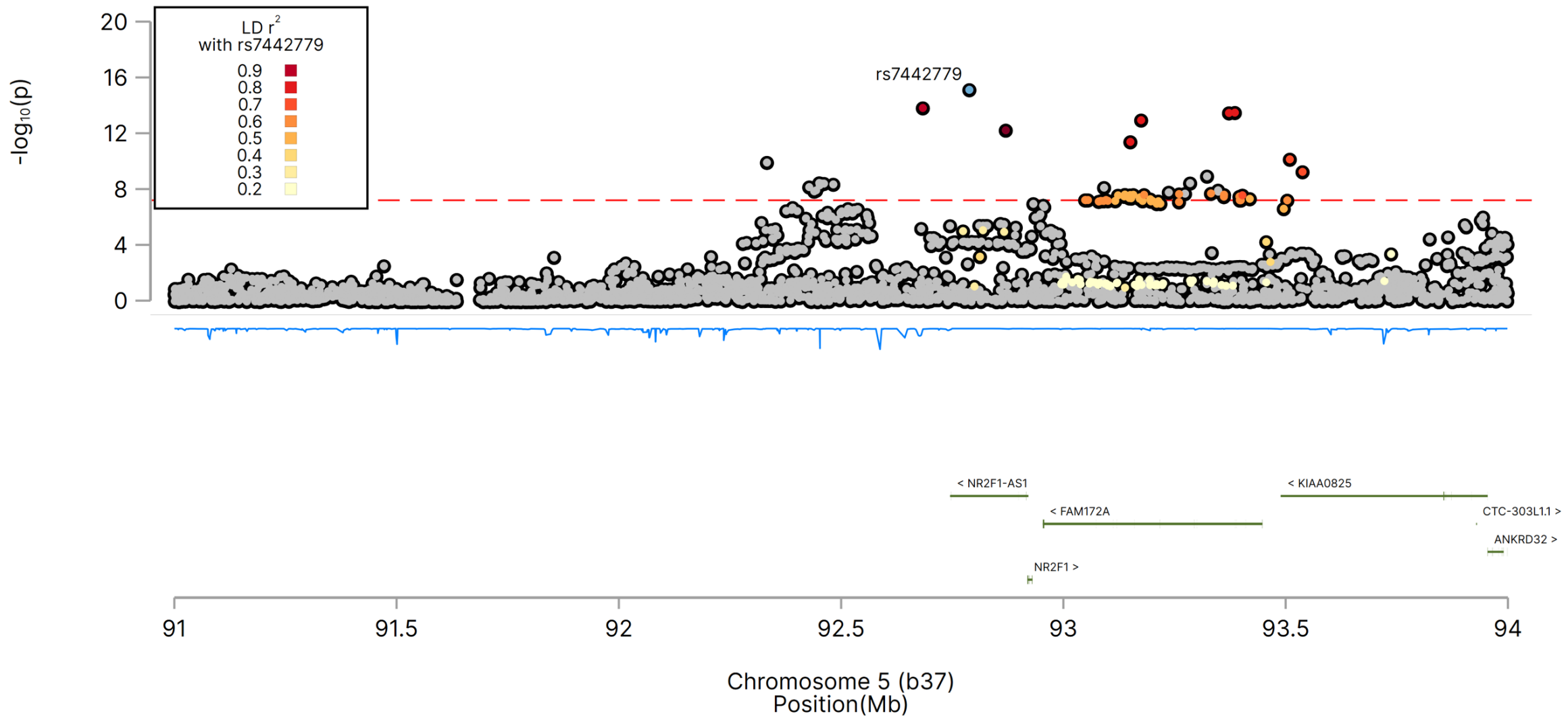

dataset: ukbb-fmri-rsn-DMN

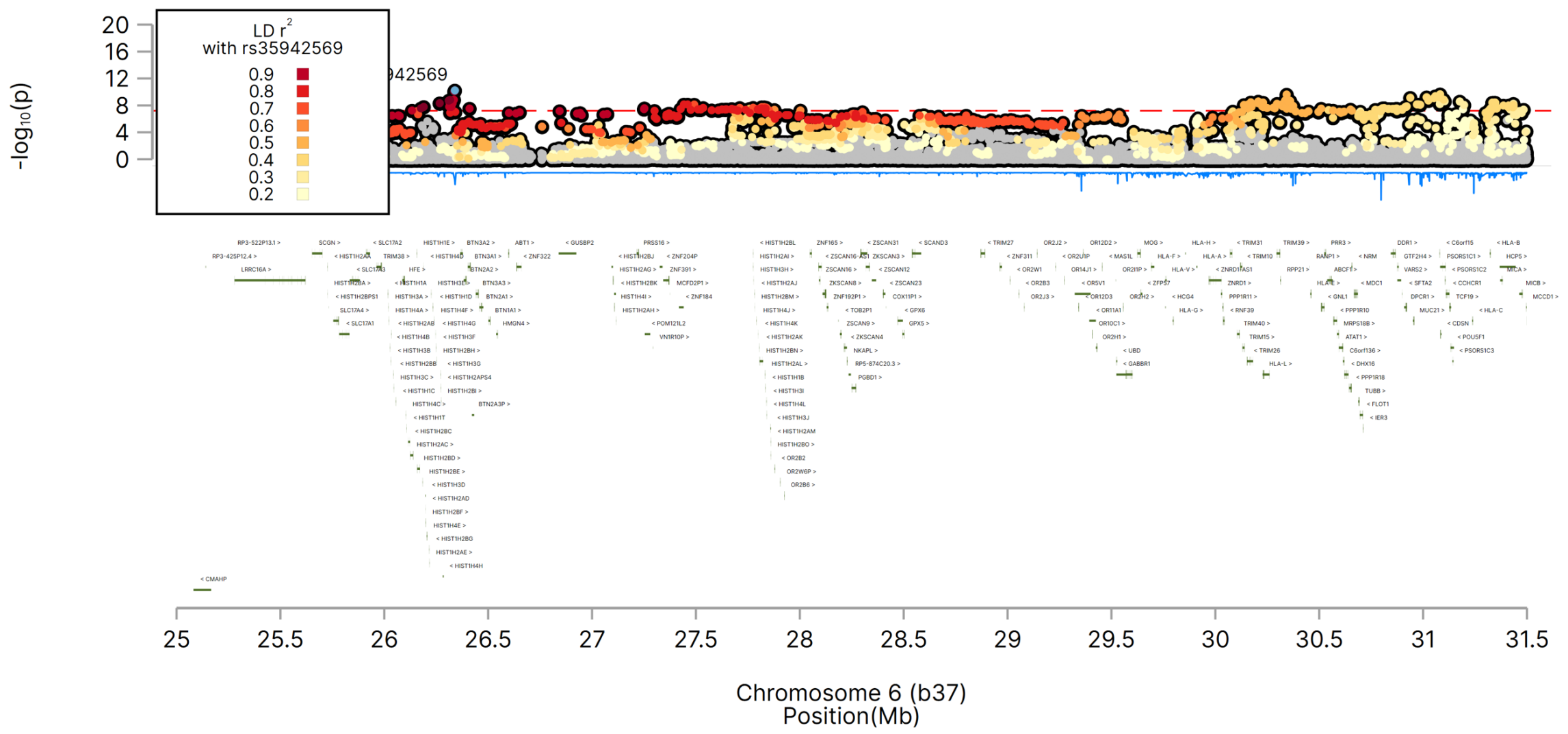

dataset: ukbb-fmri-rsn-DMN

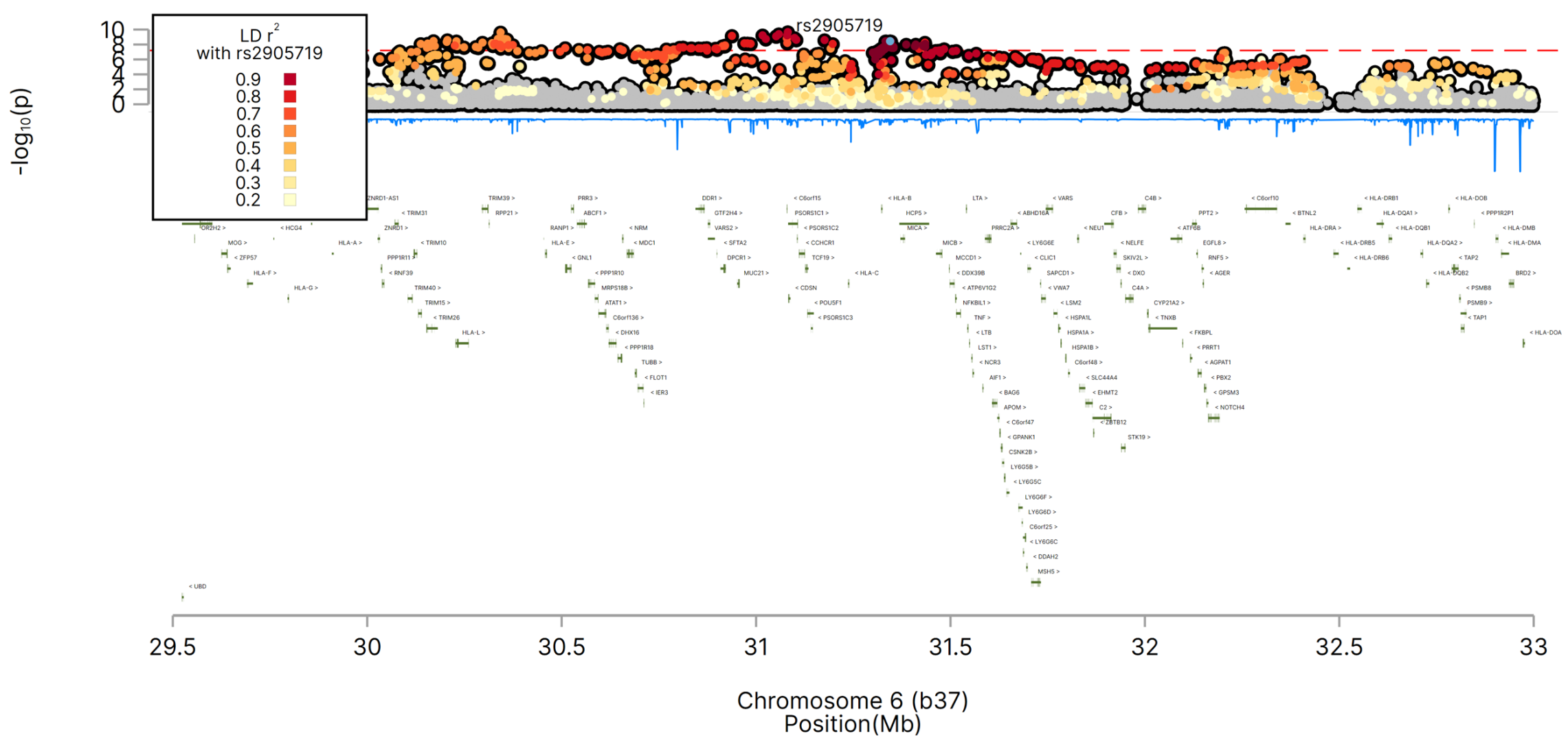

dataset: ukbb-fmri-rsn-DMN

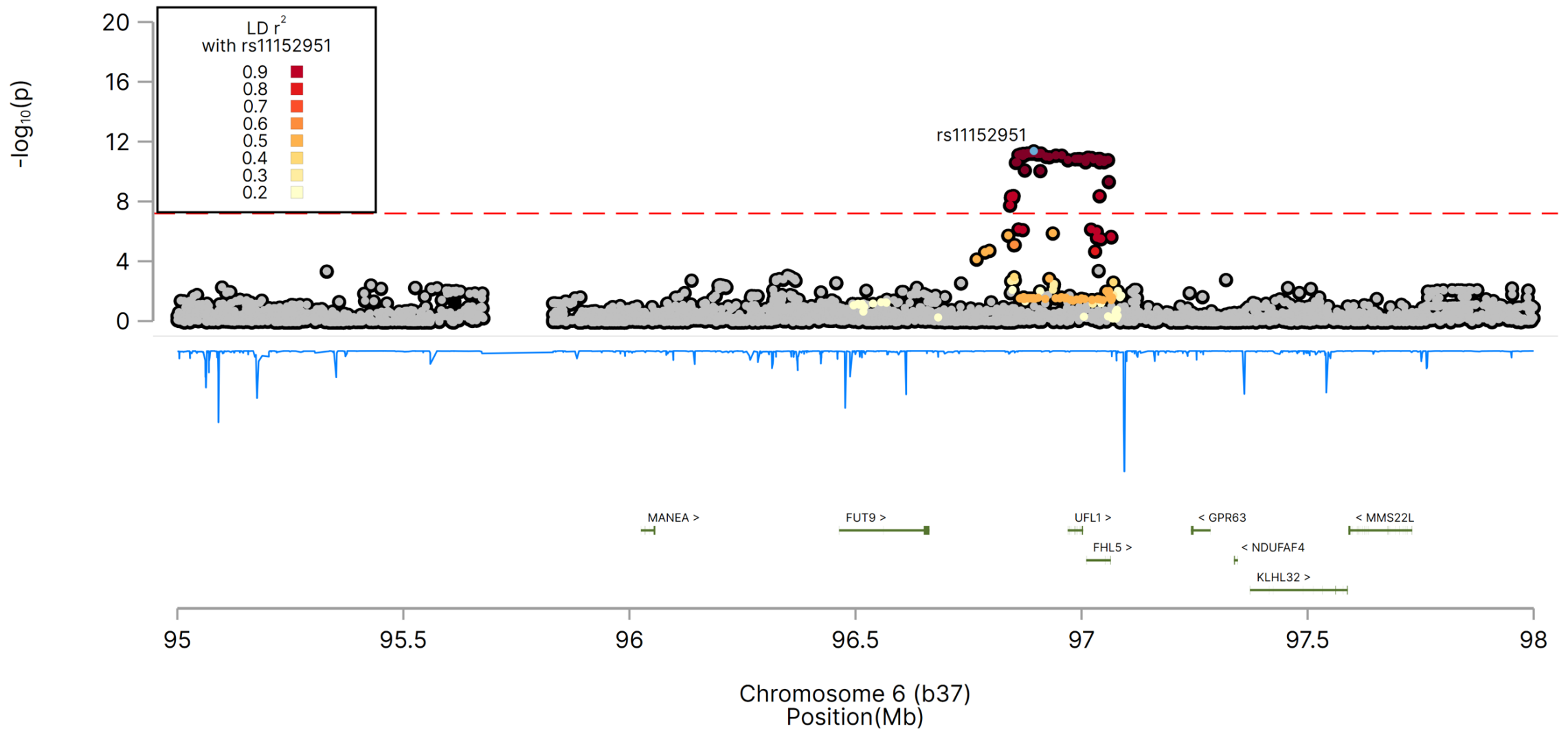

dataset: ukbb-fmri-rsn-DMN

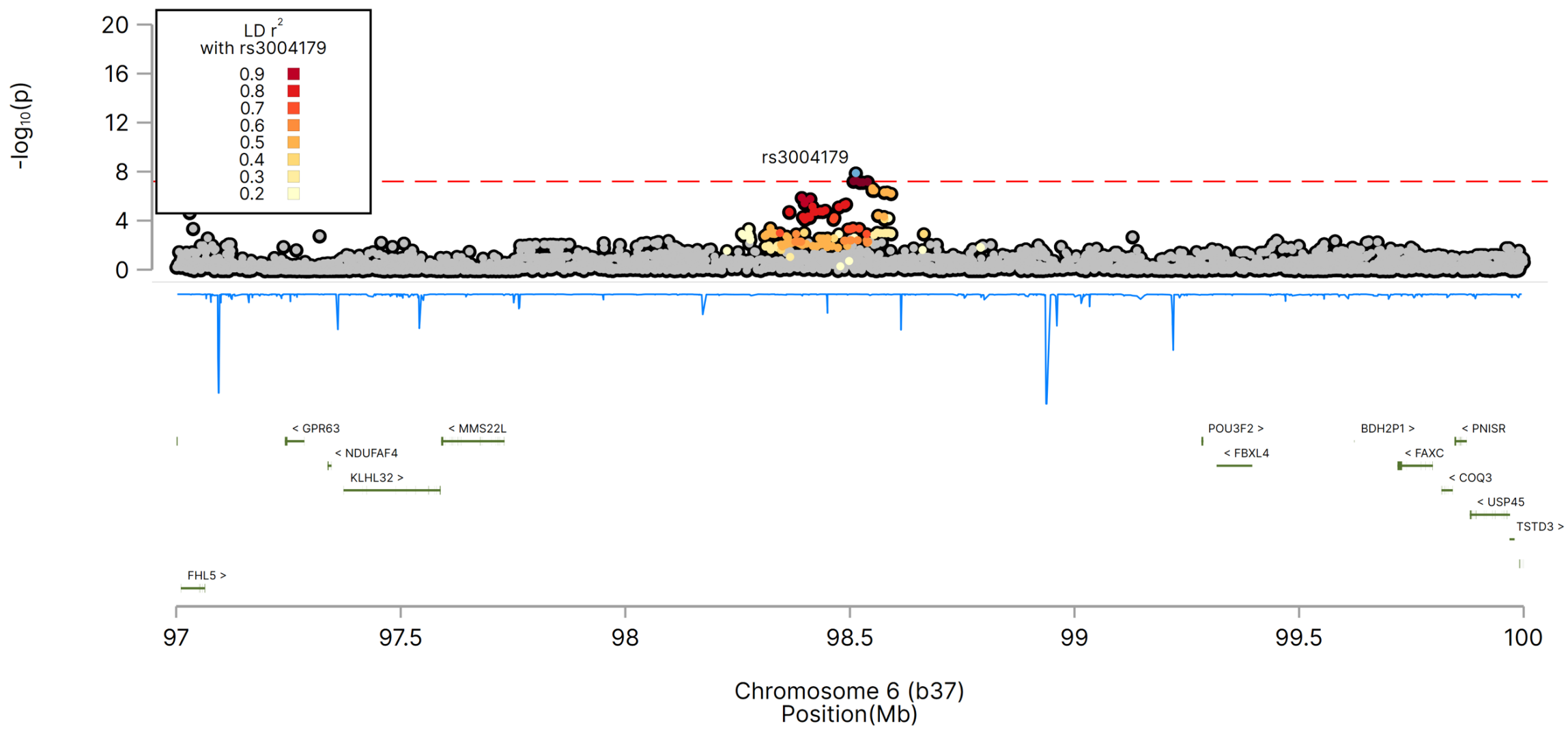

dataset: ukbb-fmri-rsn-DMN

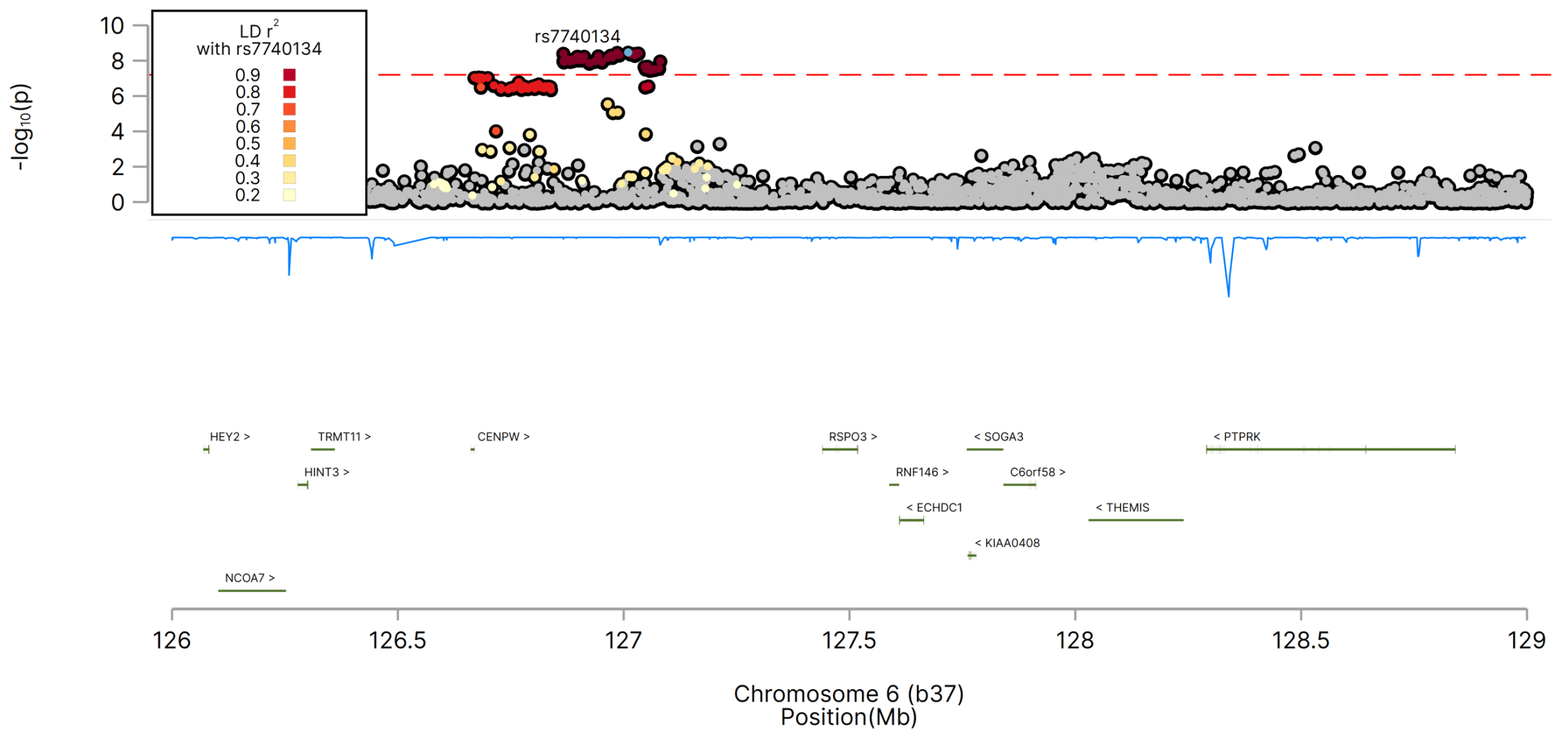

dataset: ukbb-fmri-rsn-DMN

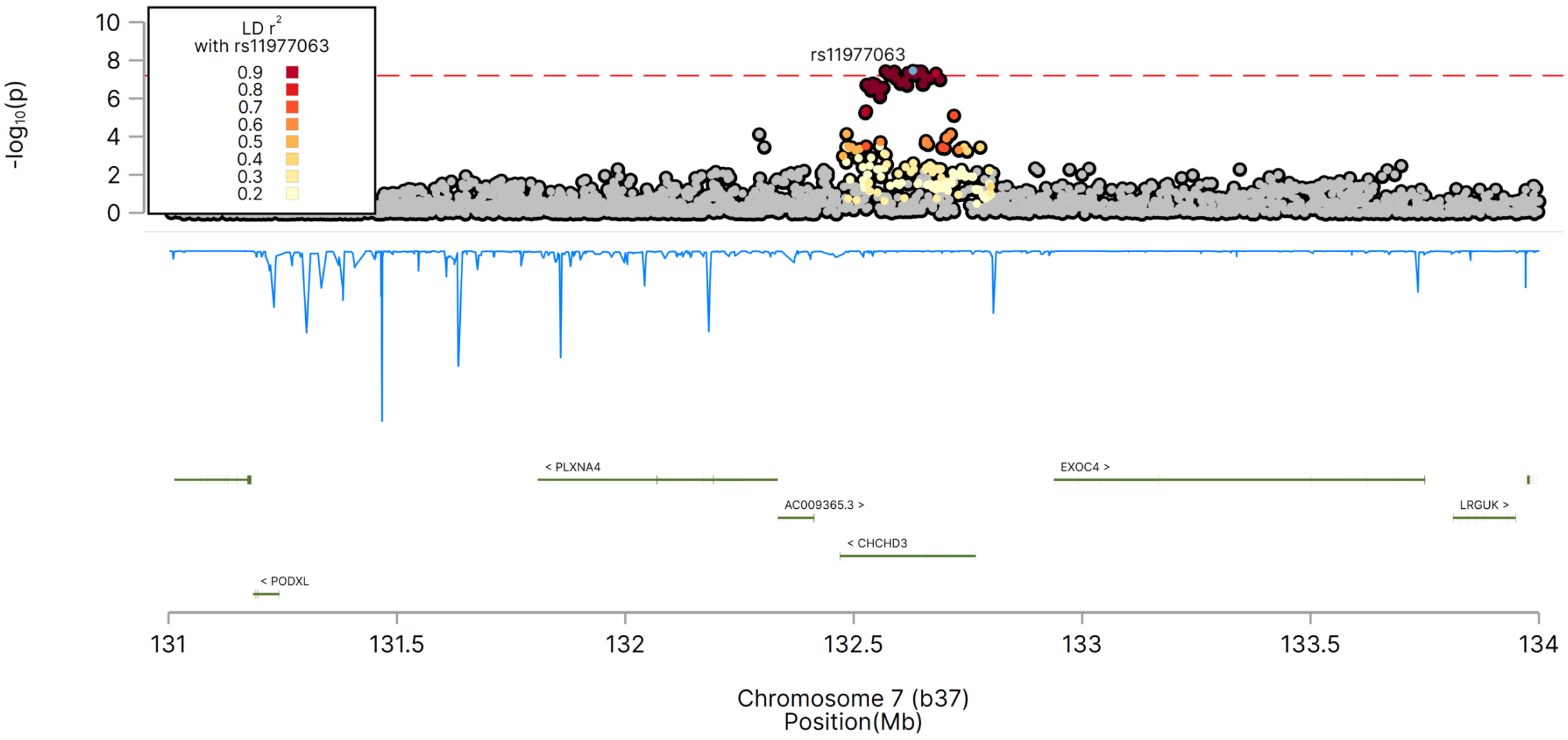

dataset: ukbb-fmri-rsn-DMN

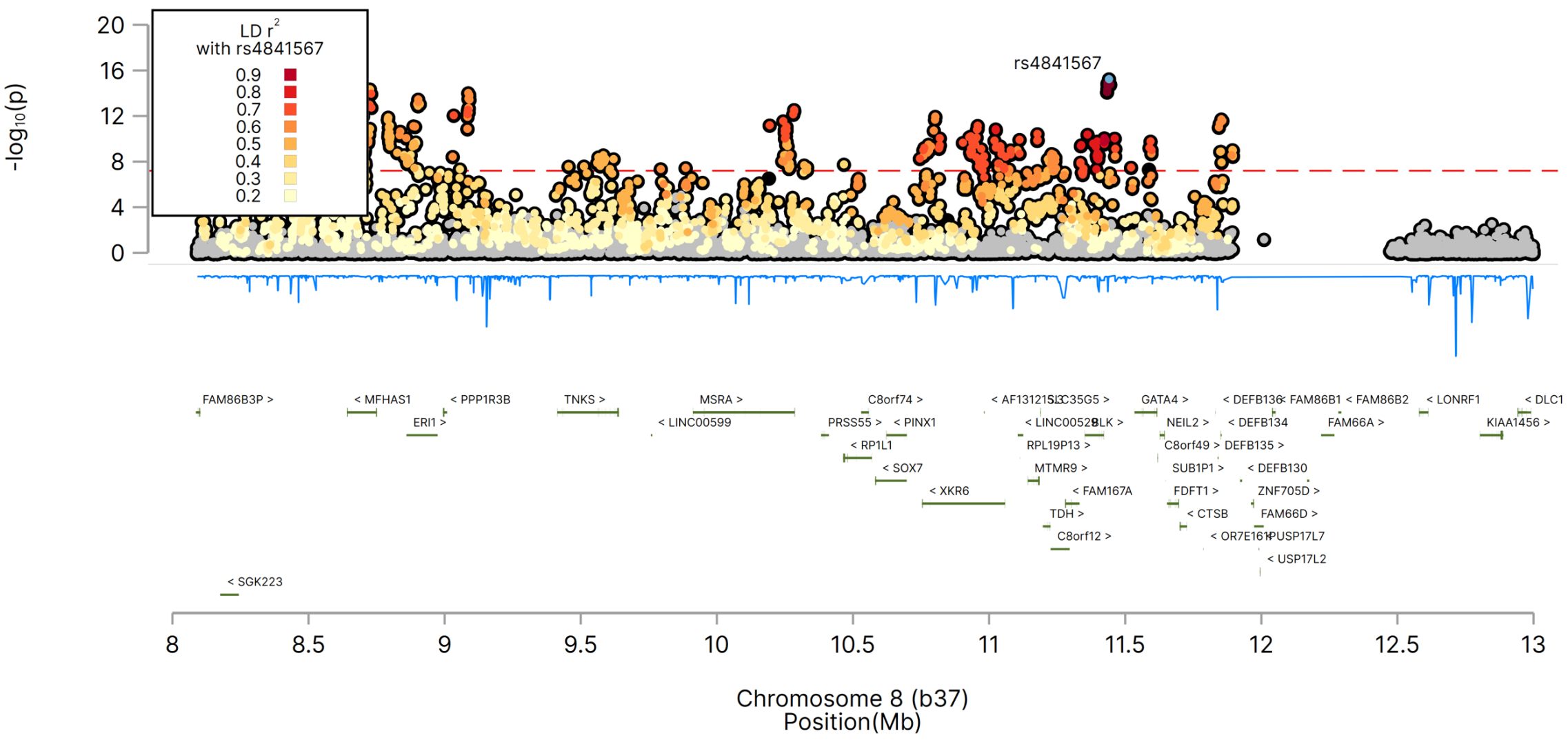

dataset: ukbb-fmri-rsn-DMN

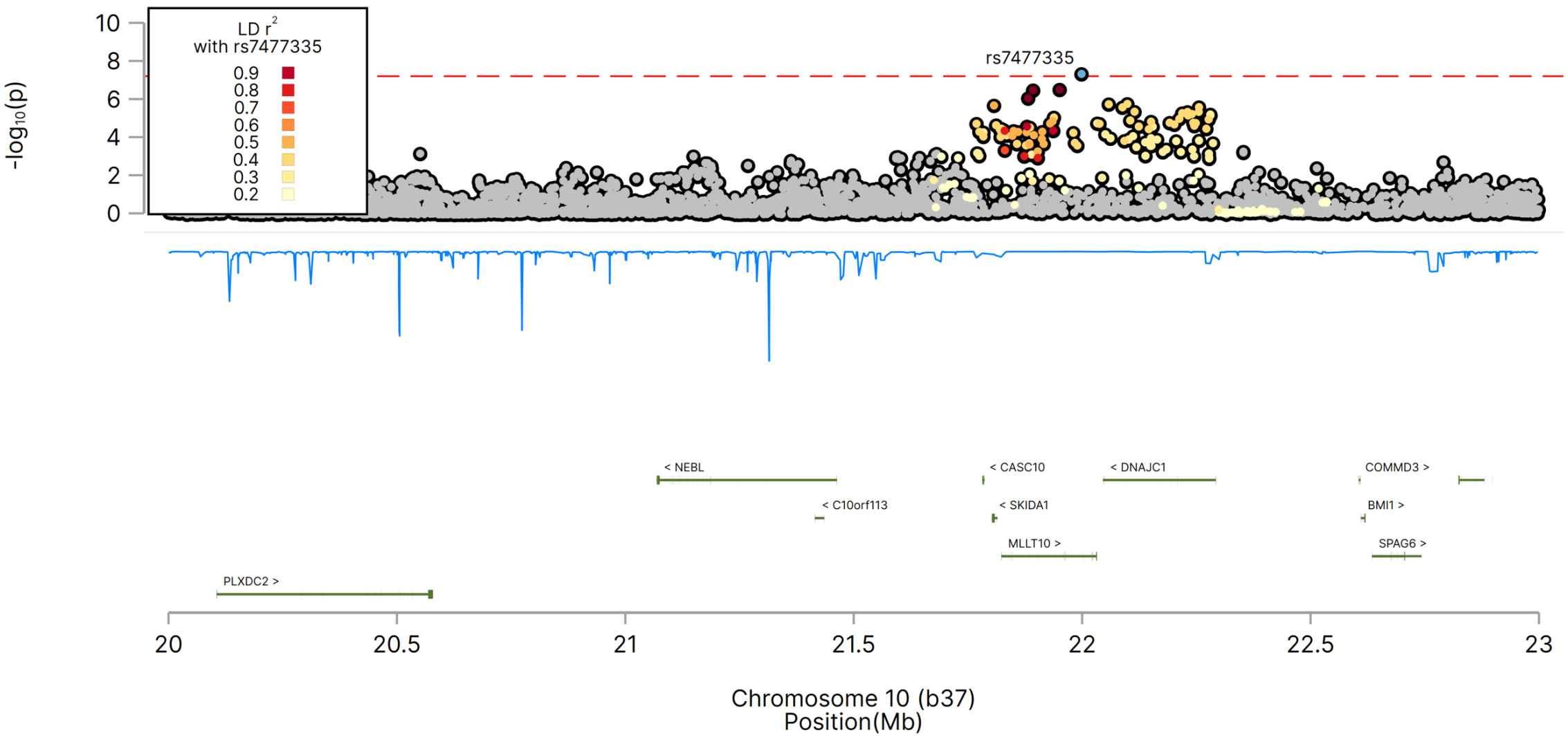

dataset: ukbb-fmri-rsn-DMN

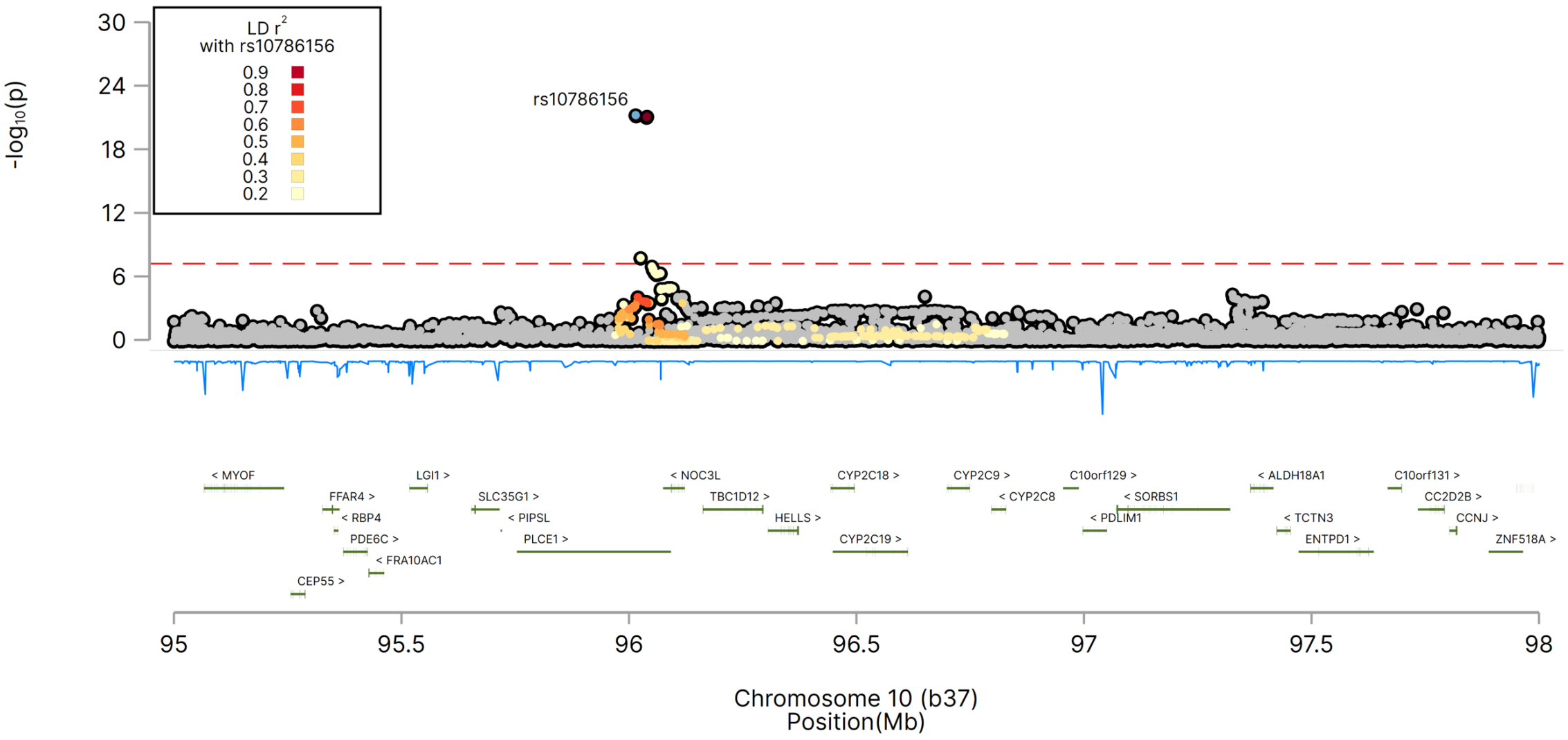

dataset: ukbb-fmri-rsn-DMN

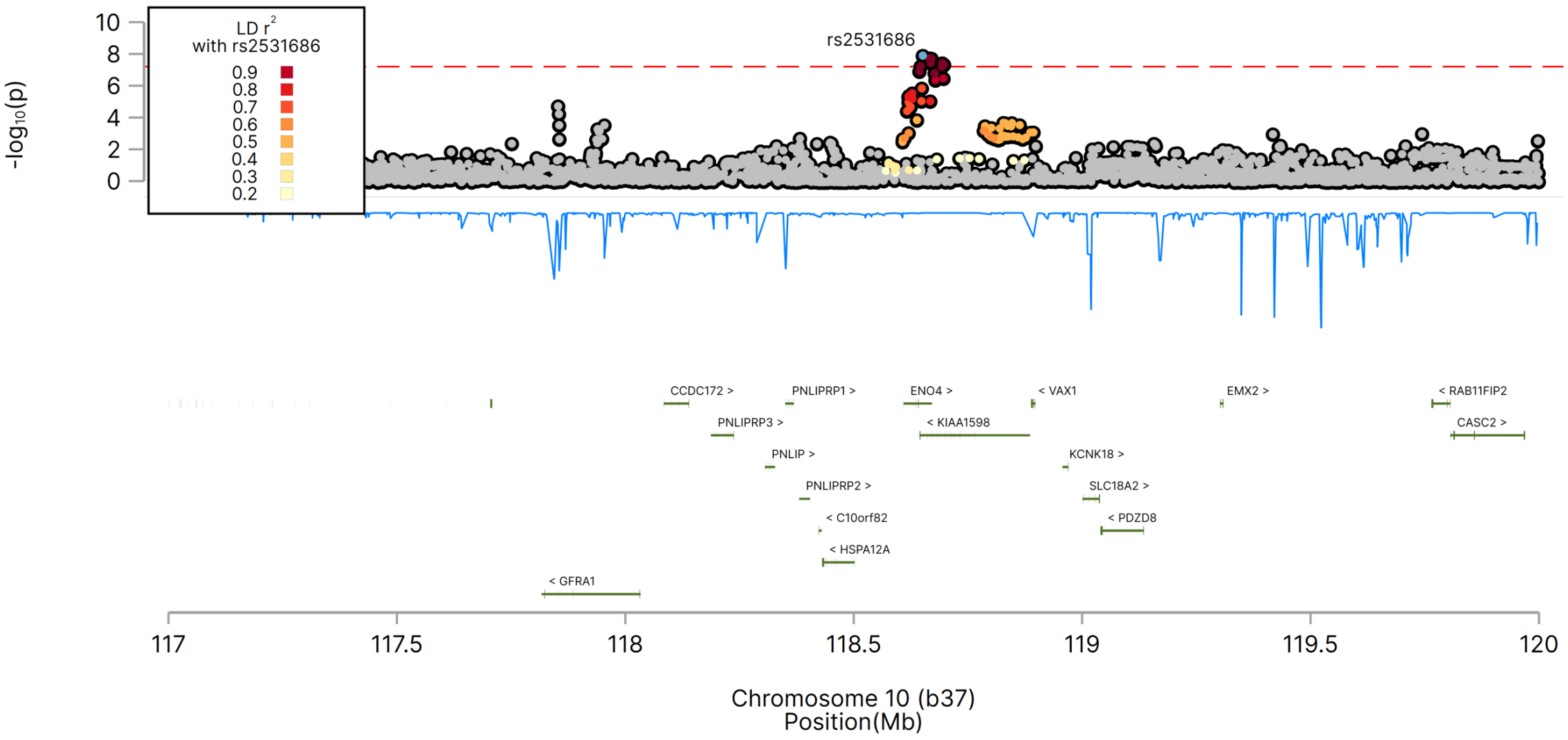

dataset: ukbb-fmri-rsn-DMN

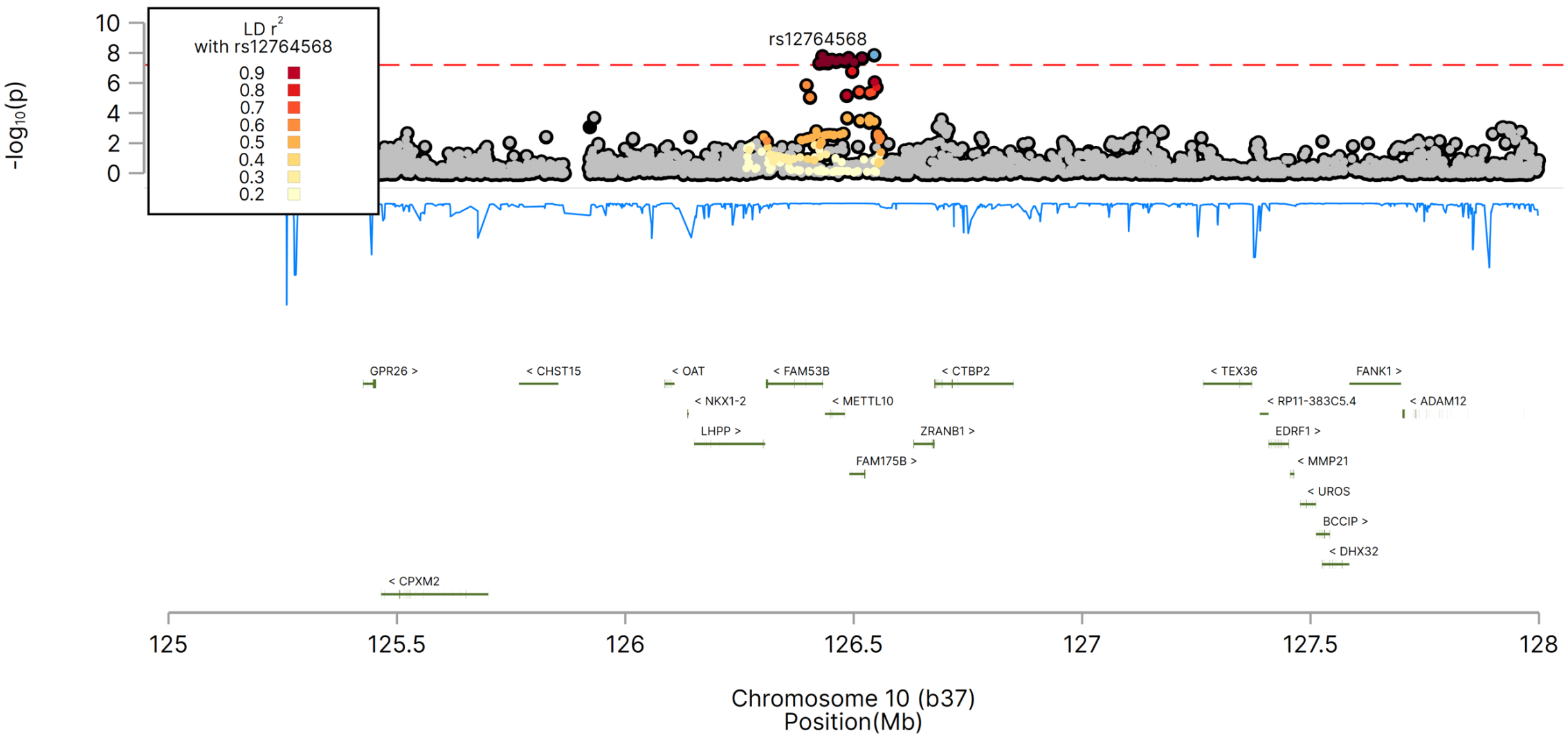

dataset: ukbb-fmri-rsn-DMN

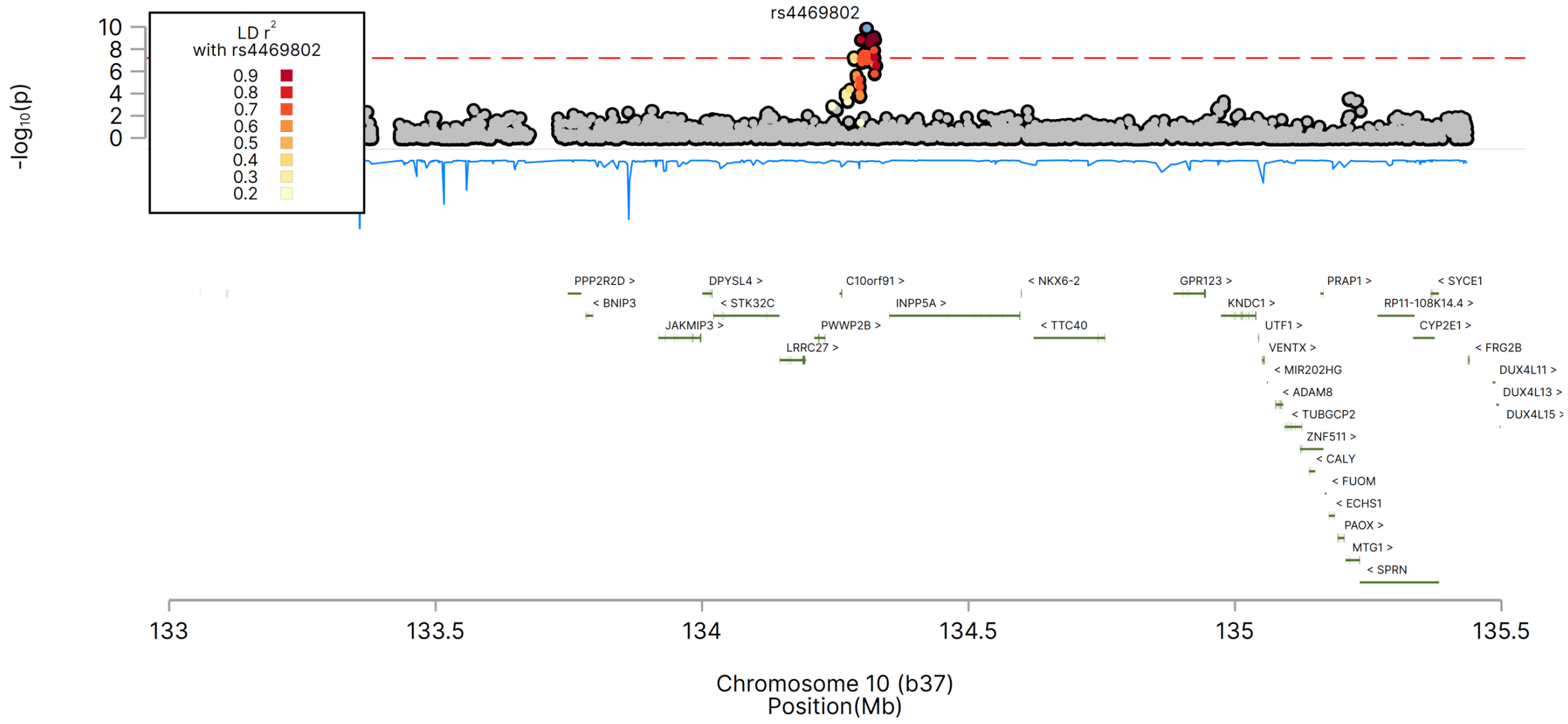

dataset: ukbb-fmri-rsn-DMN

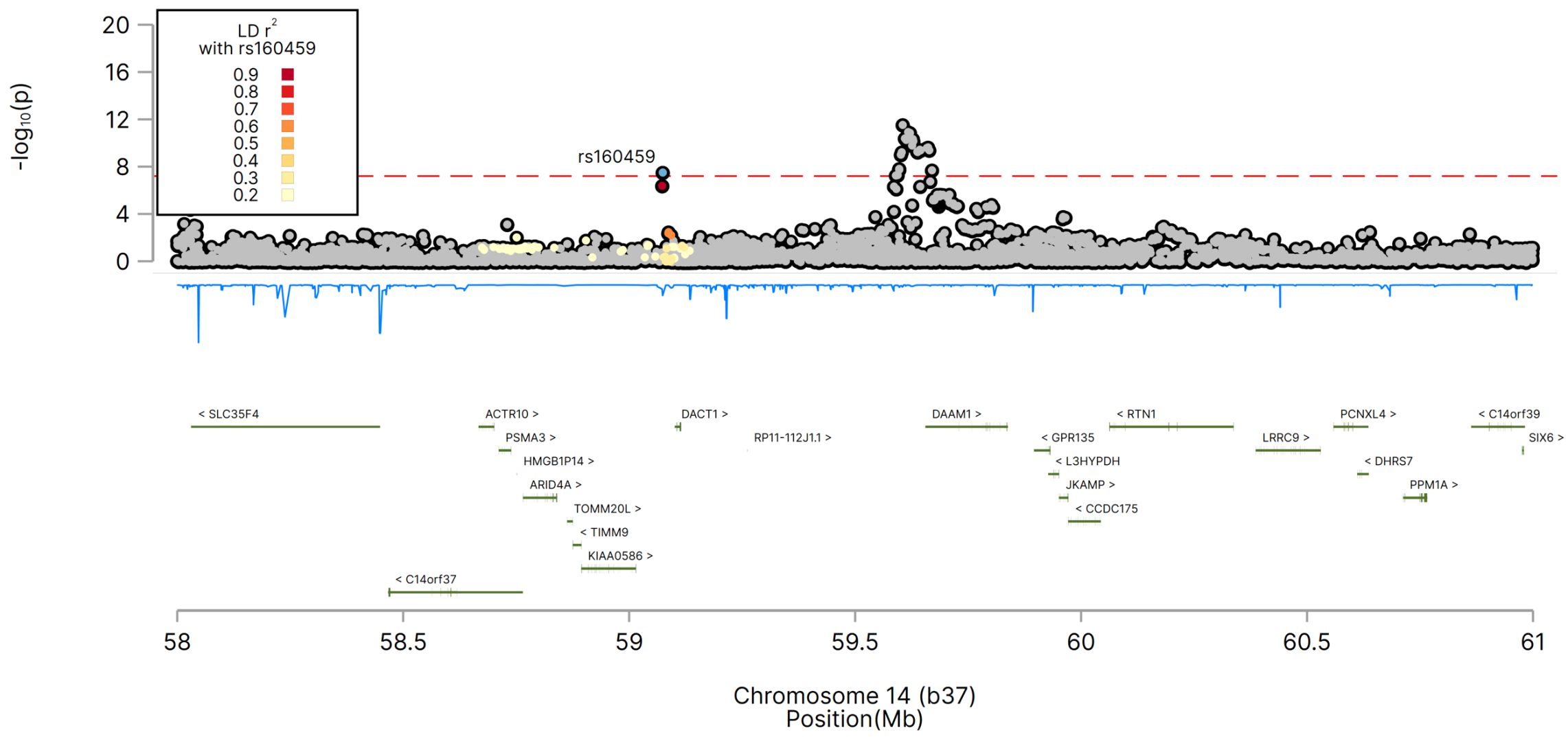

dataset: ukbb-fmri-rsn-DMN

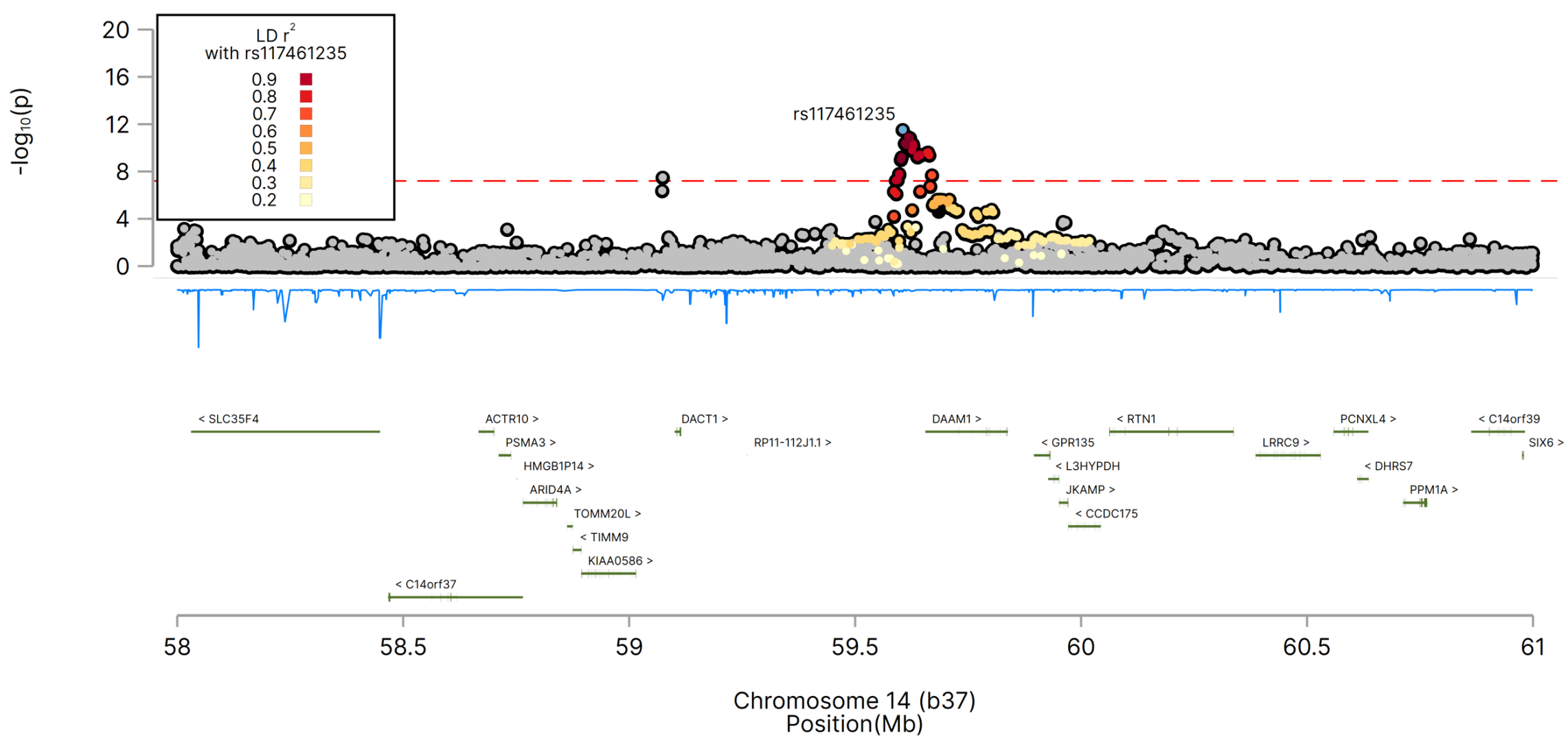

dataset: ukbb-fmri-rsn-DMN

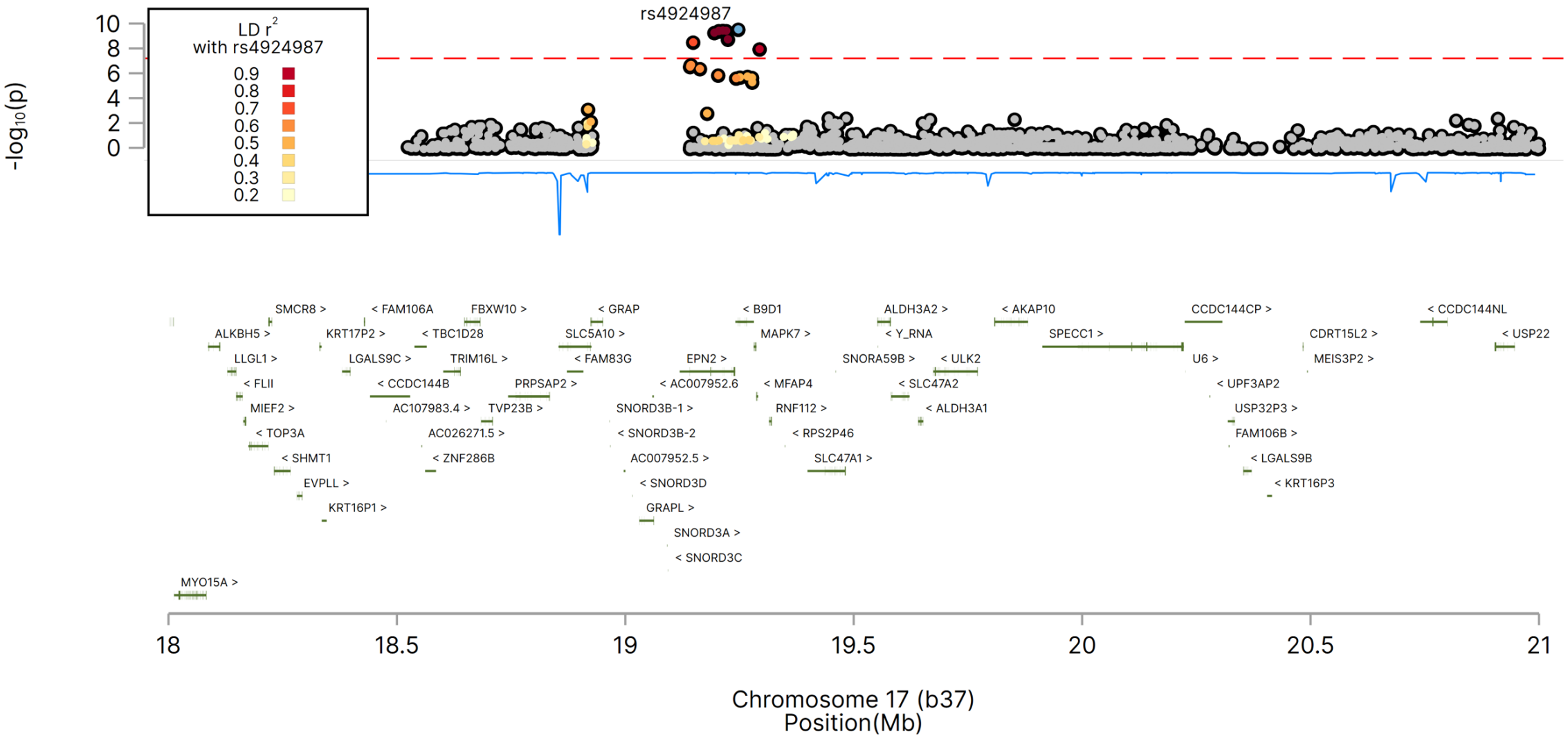

dataset: ukbb-fmri-rsn-DMN

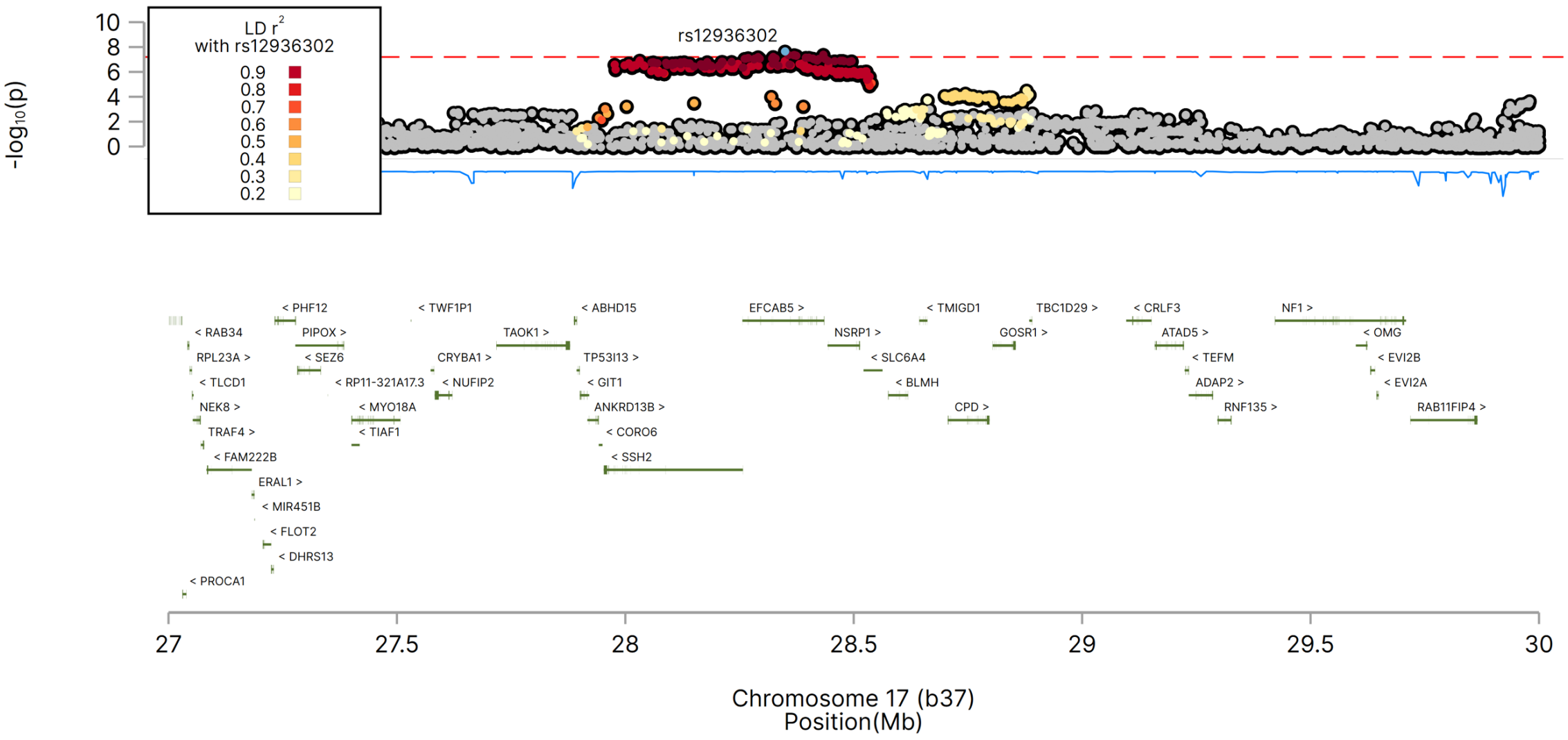

dataset: ukbb-fmri-rsn-DMN

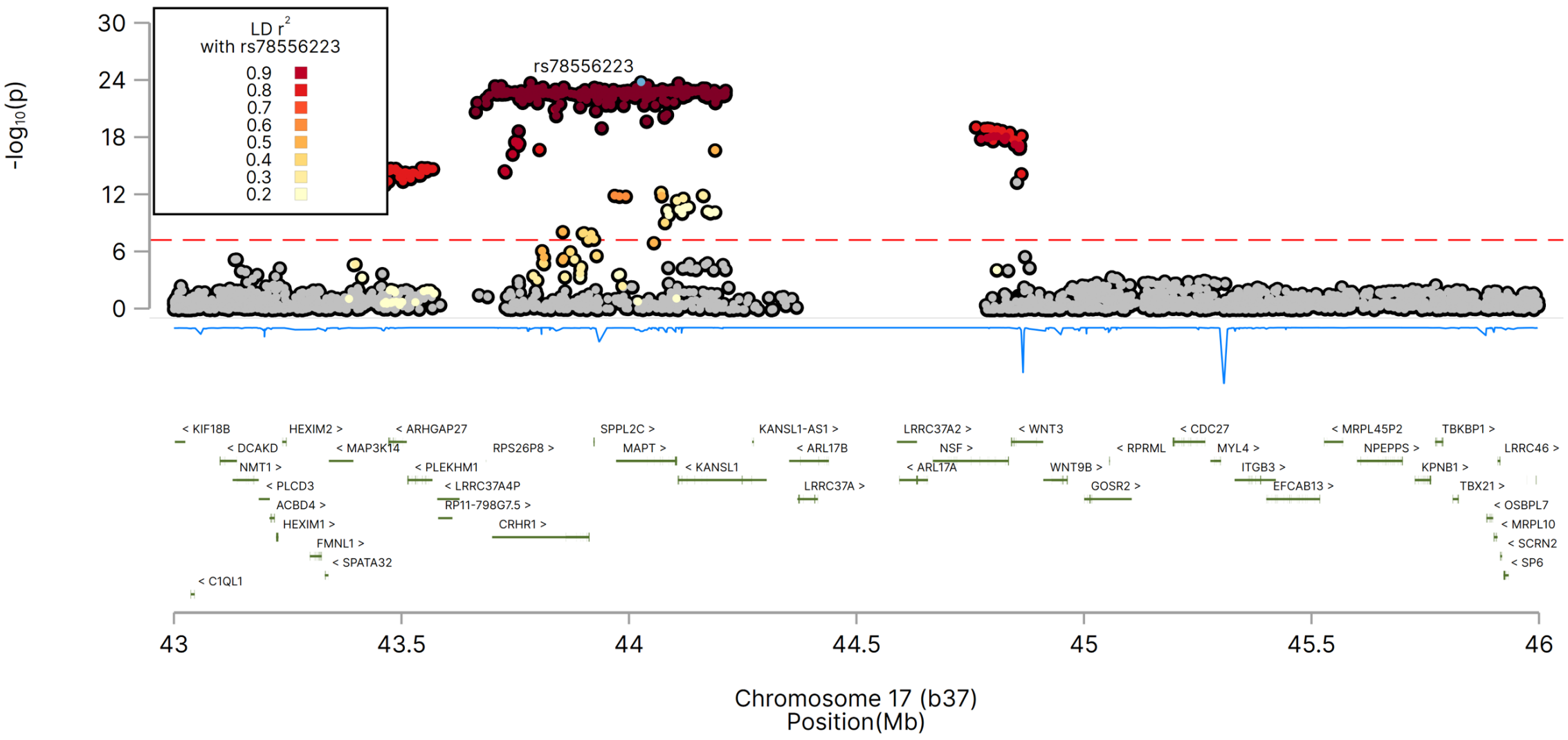

dataset: ukbb-fmri-rsn-DMN

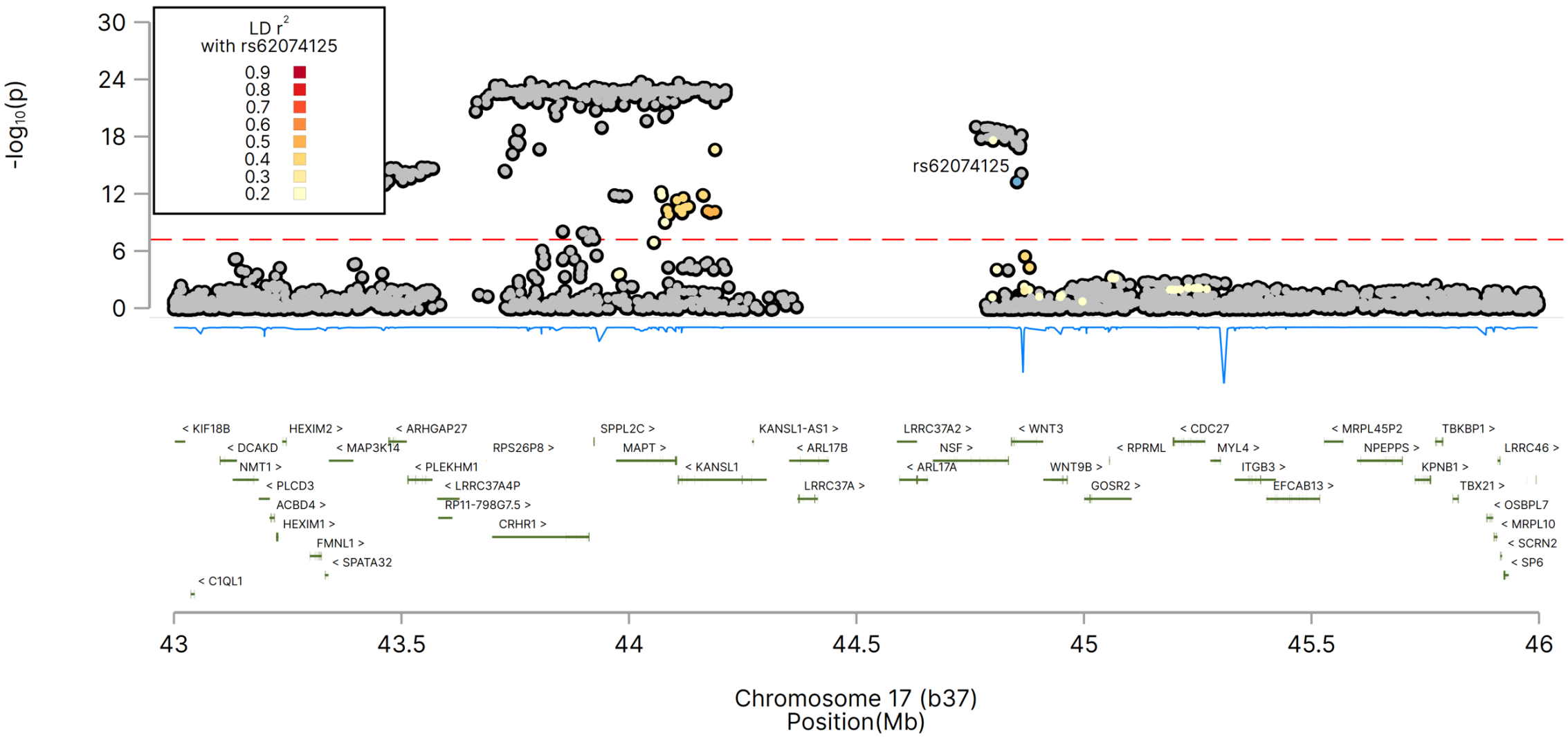

dataset: ukbb-fmri-rsn-DMN

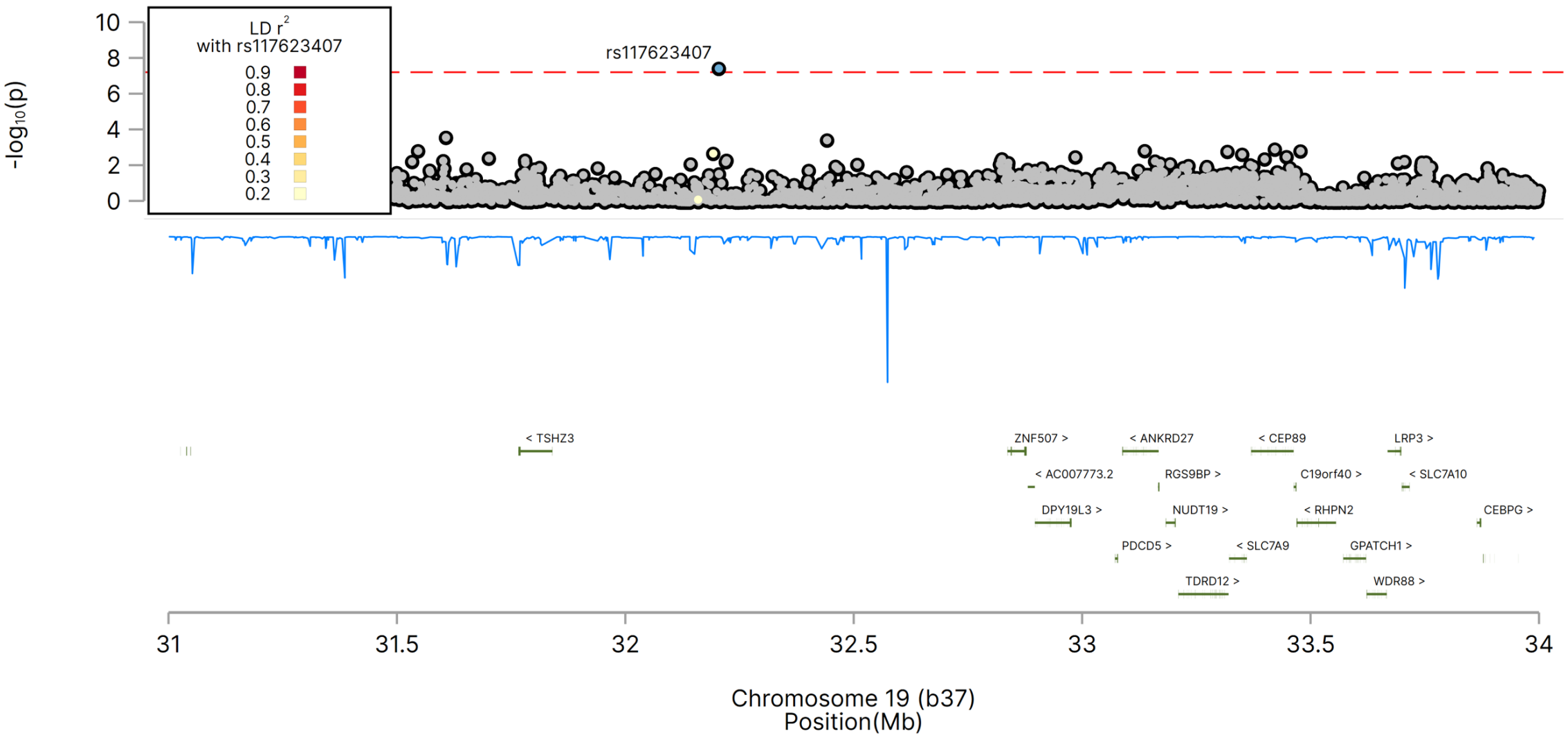

dataset: ukbb-fmri-rsn-DMN

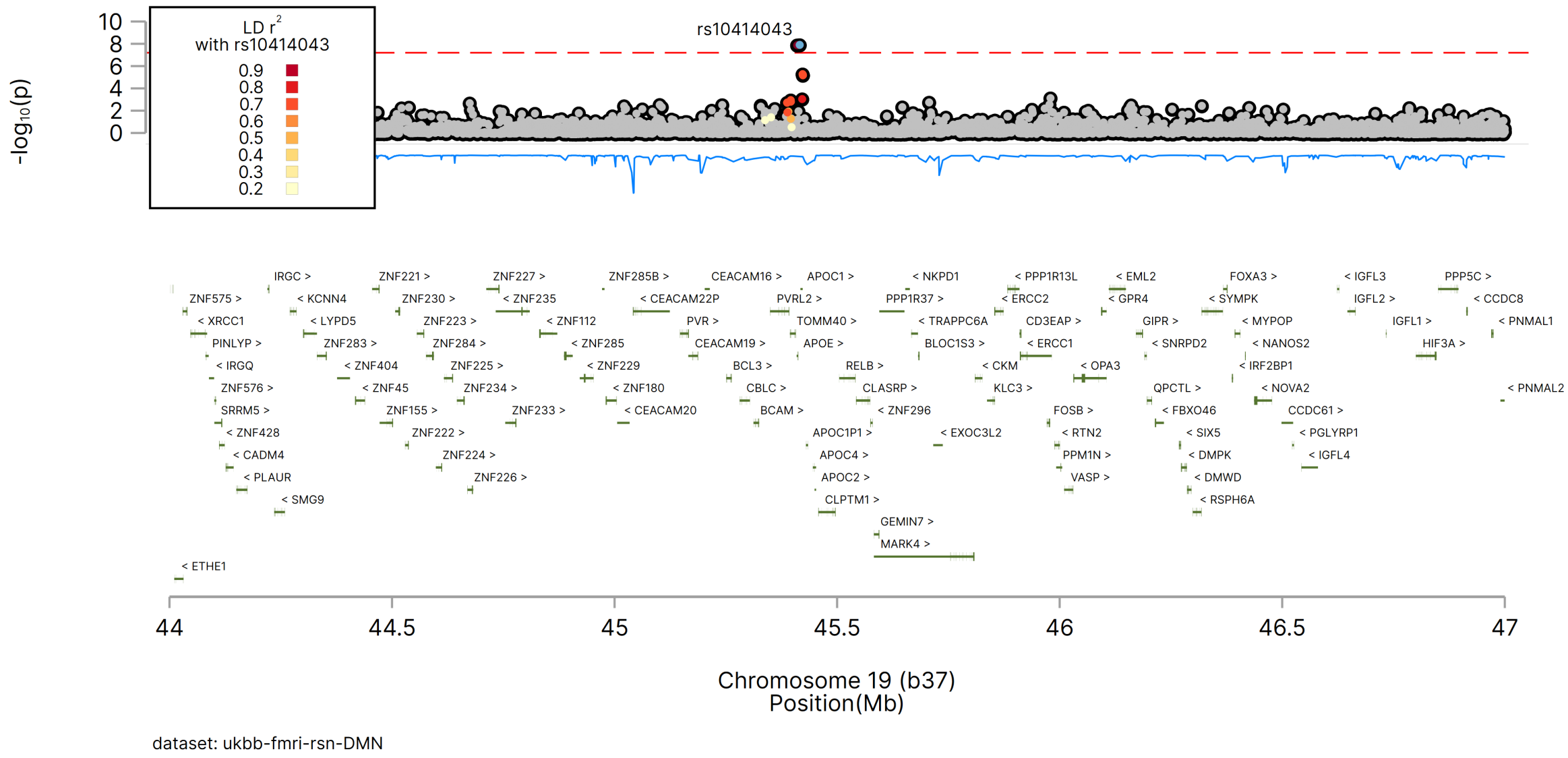

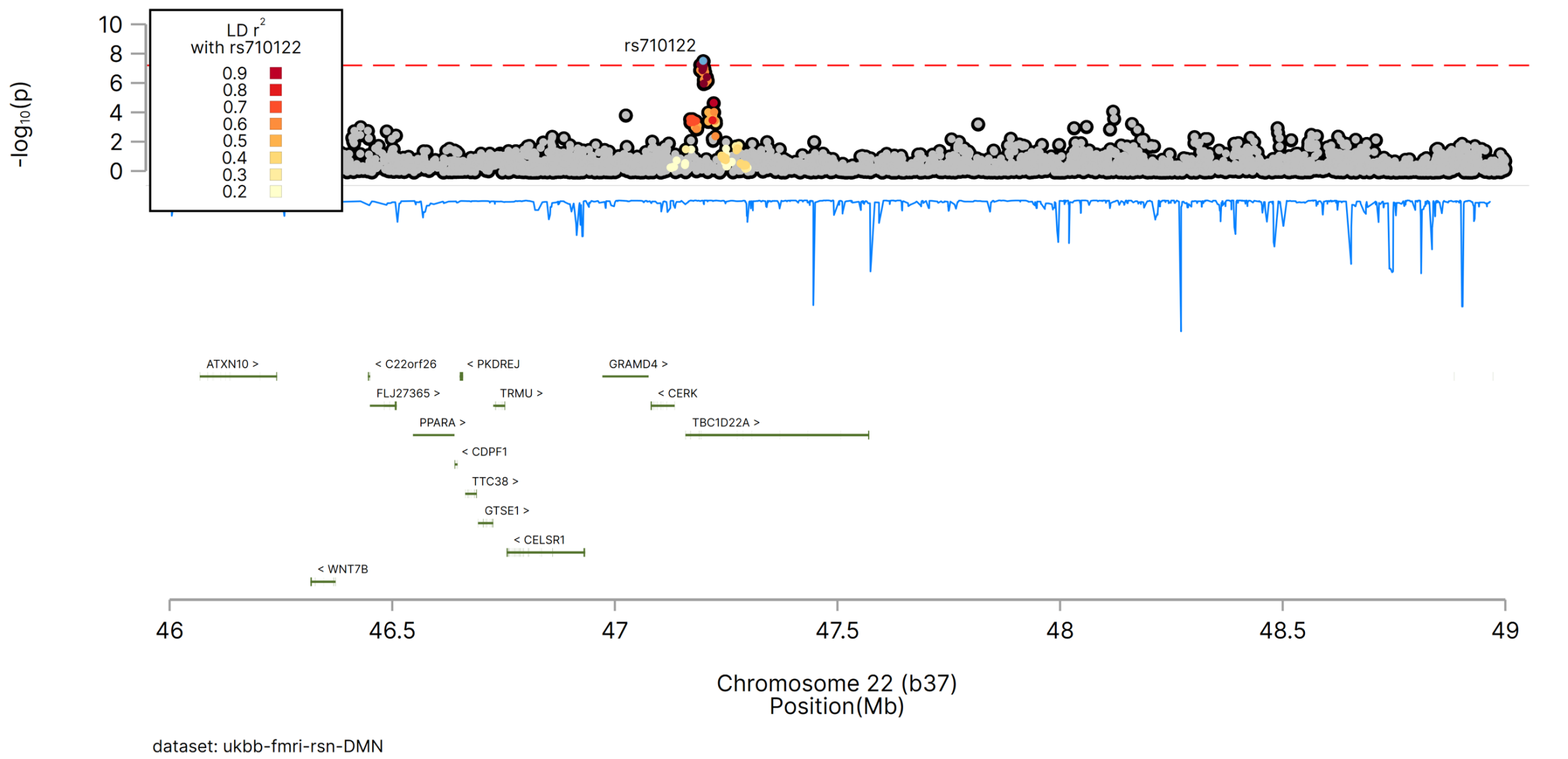

Supplementary Figure 4. Locus-zoom for all significant LD-independent signals coming from the MOST GWAS test for the Default Mode Network (DMN). Source data for this figure can be obtained from https://www.ebi.ac.uk/gwas/; hyperlinks to the data for each network are provided in the Data Availability section.

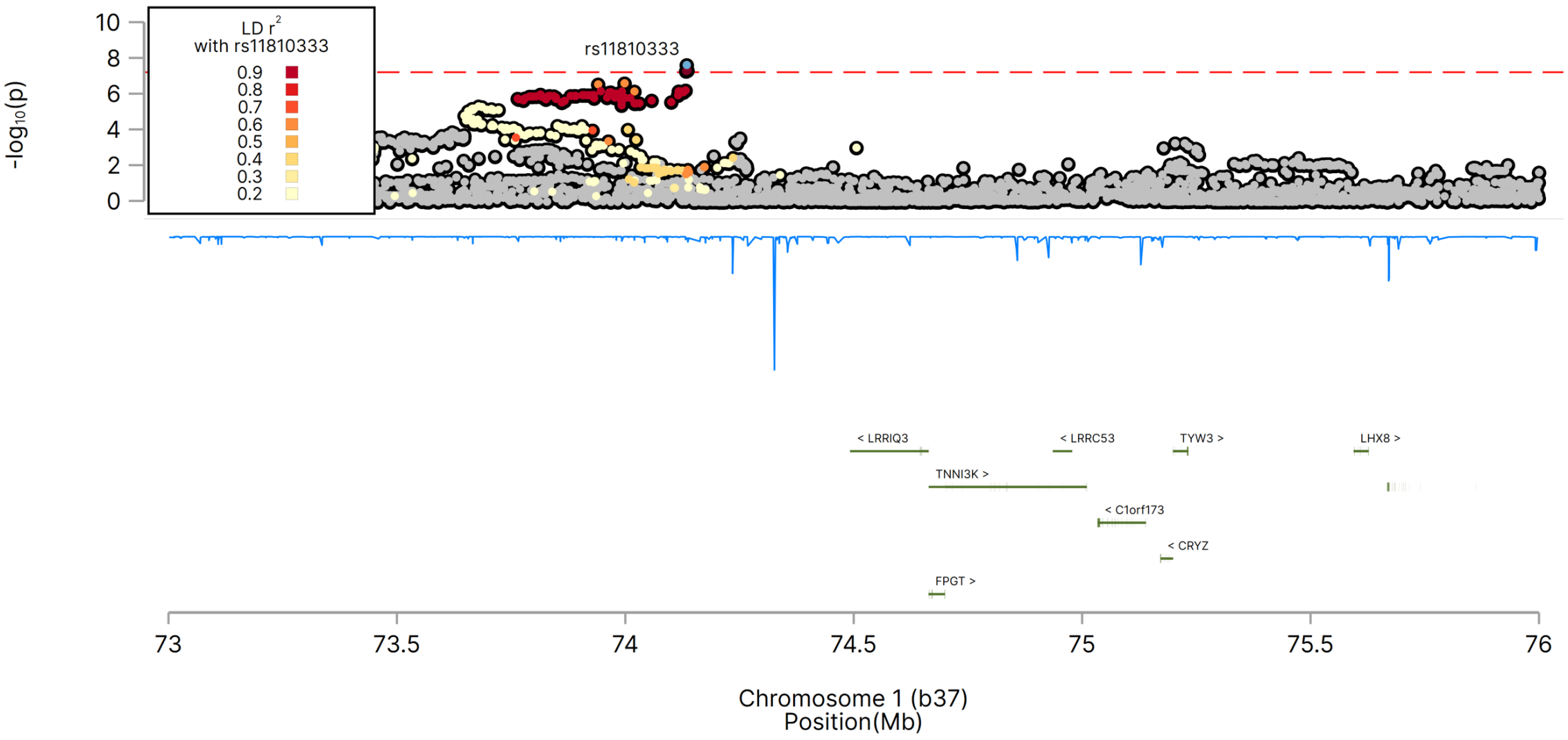

dataset: ukbb-fmri-rsn-FPN

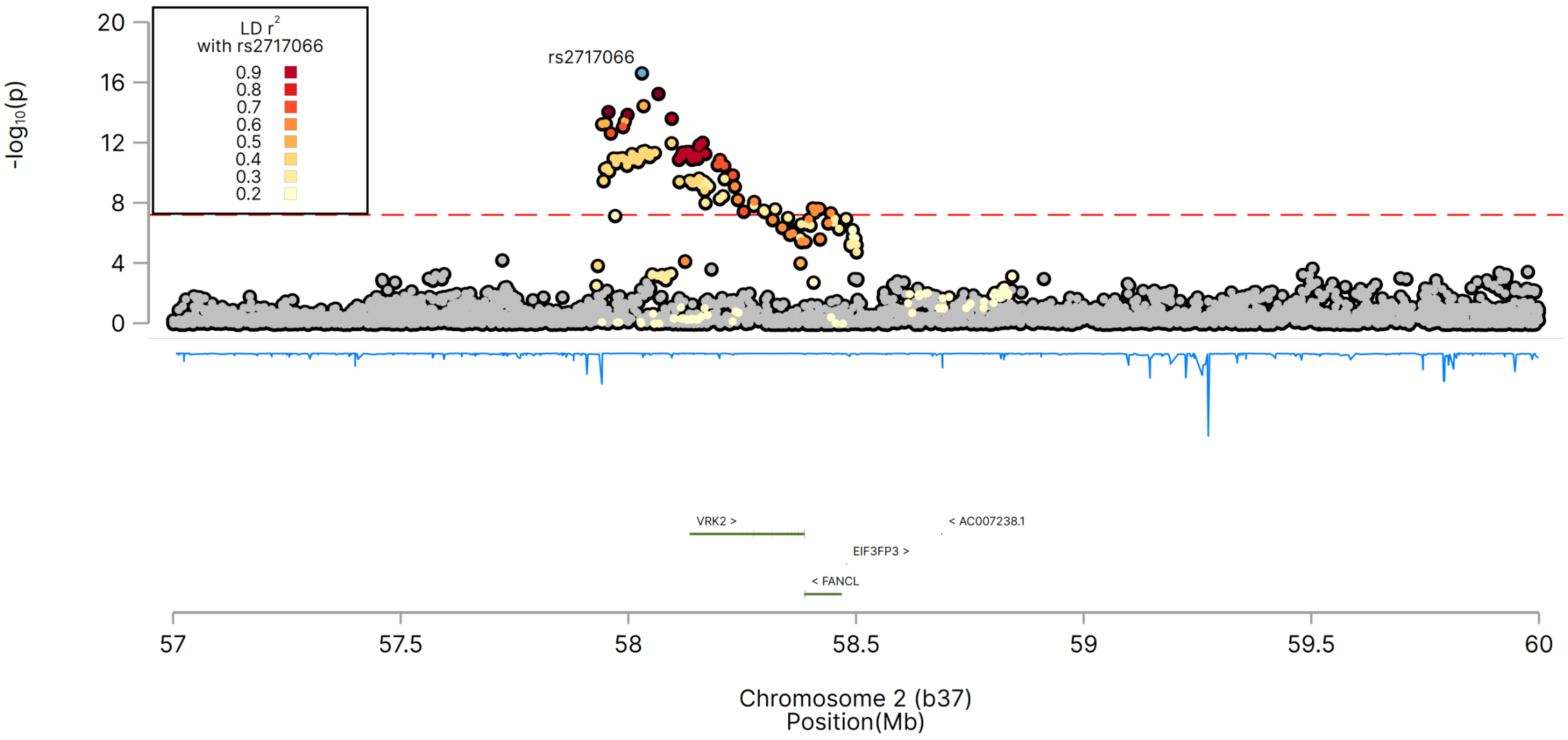

dataset: ukbb-fmri-rsn-FPN

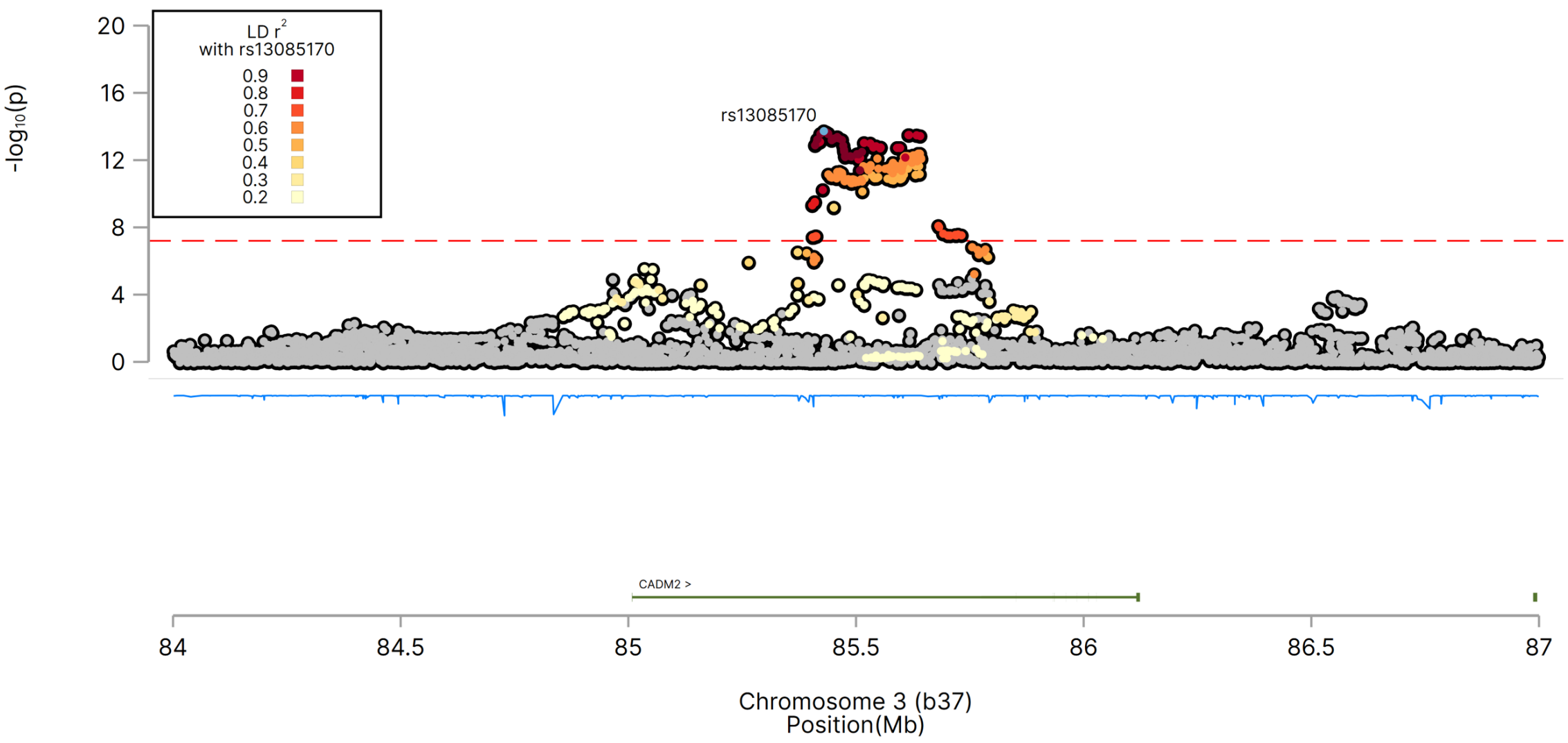

dataset: ukbb-fmri-rsn-FPN

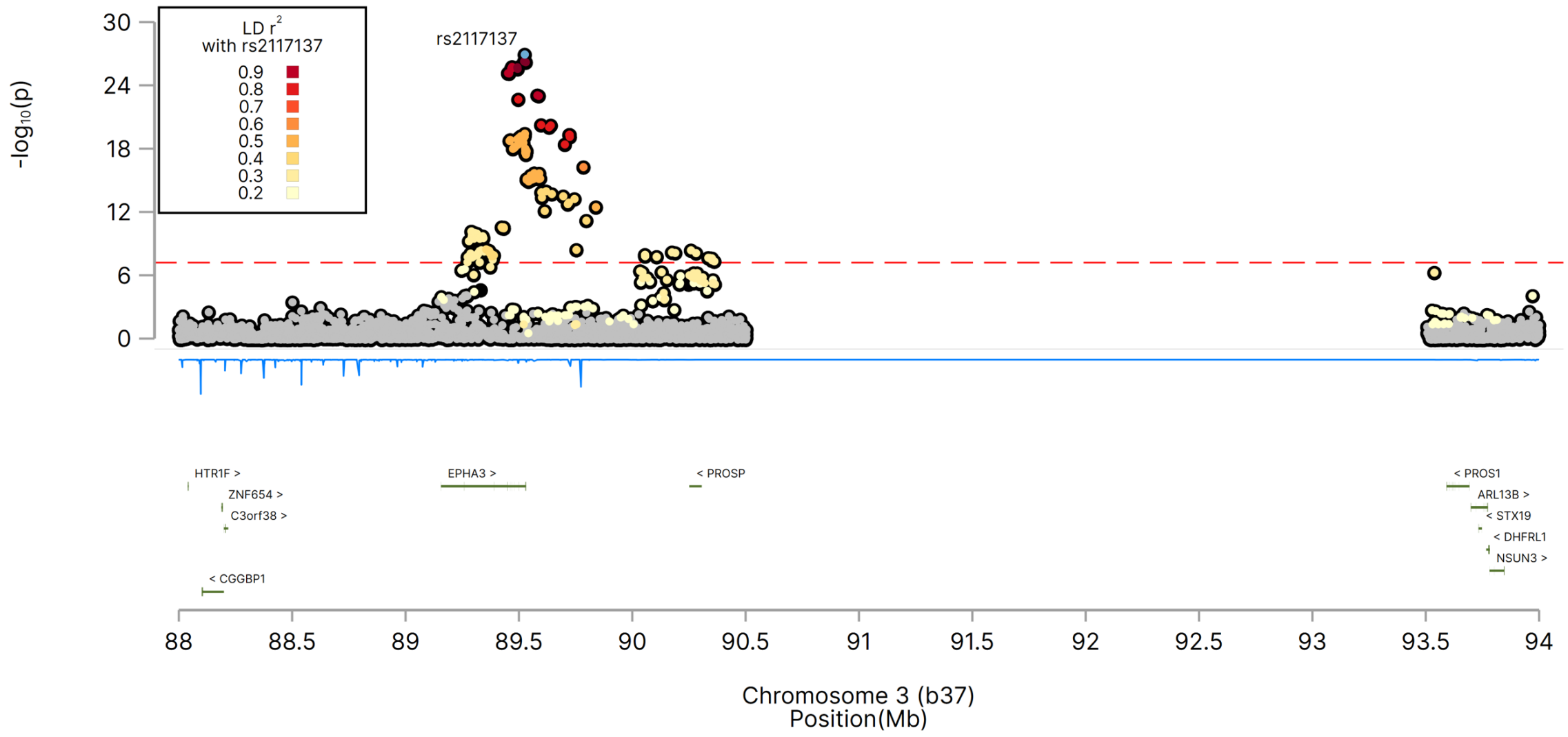

dataset: ukbb-fmri-rsn-FPN

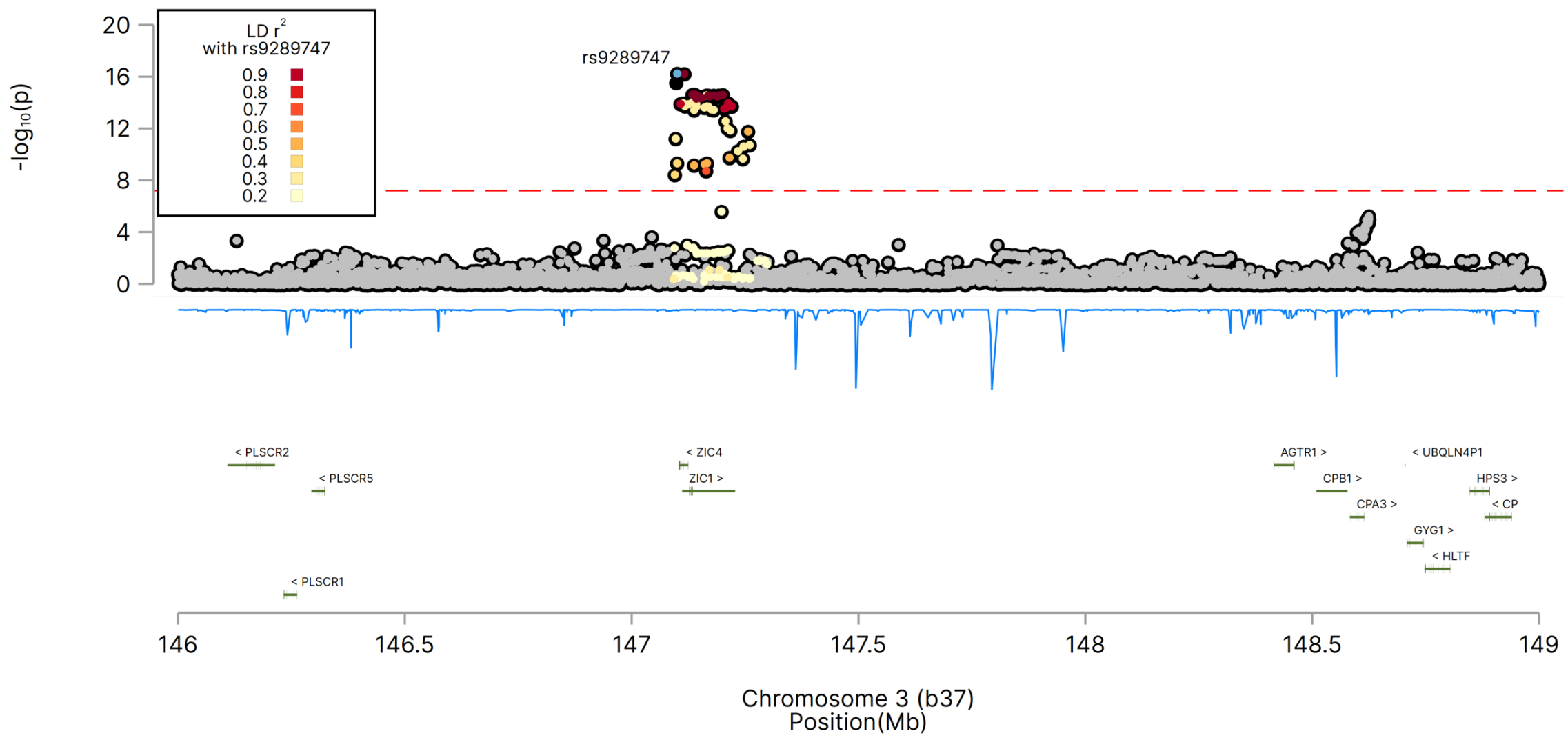

dataset: ukbb-fmri-rsn-FPN

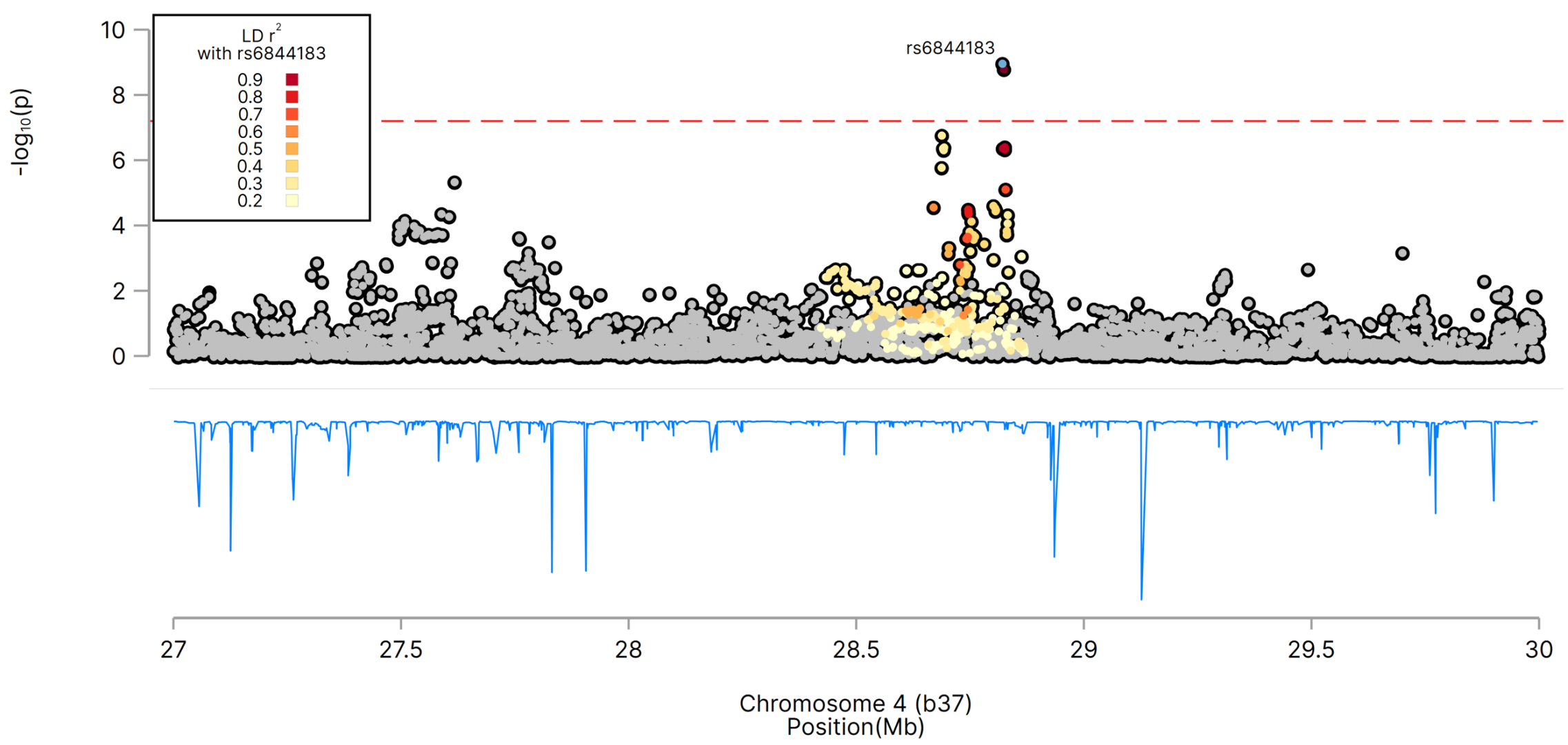

dataset: ukbb-fmri-rsn-FPN

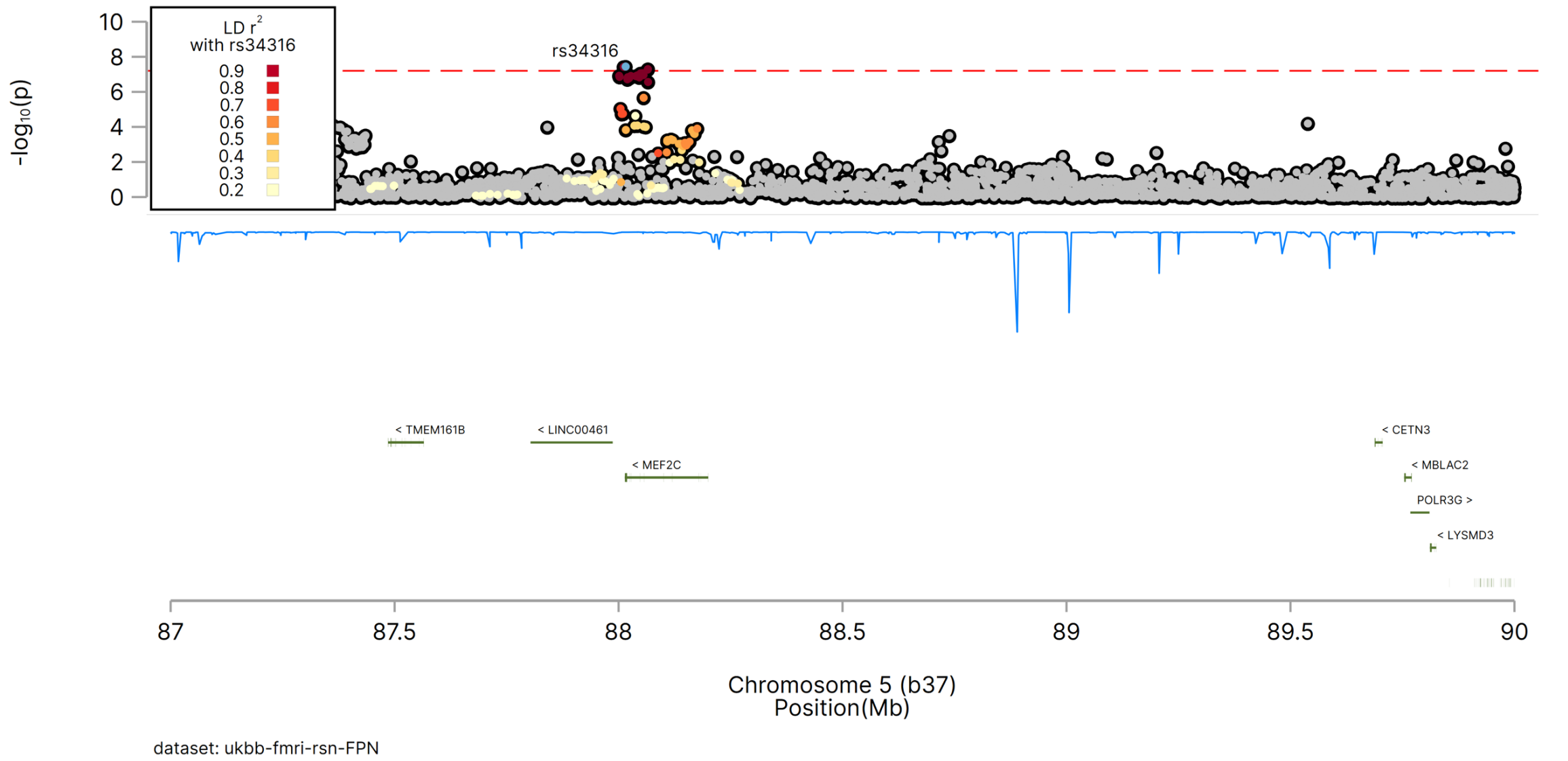

LD r²
with rs34316
0.9
0.8
0.7
0.6
0.5
0.4
0.3
0.2
-log10(p)
10
8
6
4
2
0
rs34316
< TMEM161B
< LINC00461
< MEF2C
< CETN3
< MBLAC2
POLR3G >
< LYSMD3
87
87.5
88
88.5
89
89.5
90
Chromosome 5 (b37)
Position(Mb)
dataset: ukbb-fmri-rsn-FPN

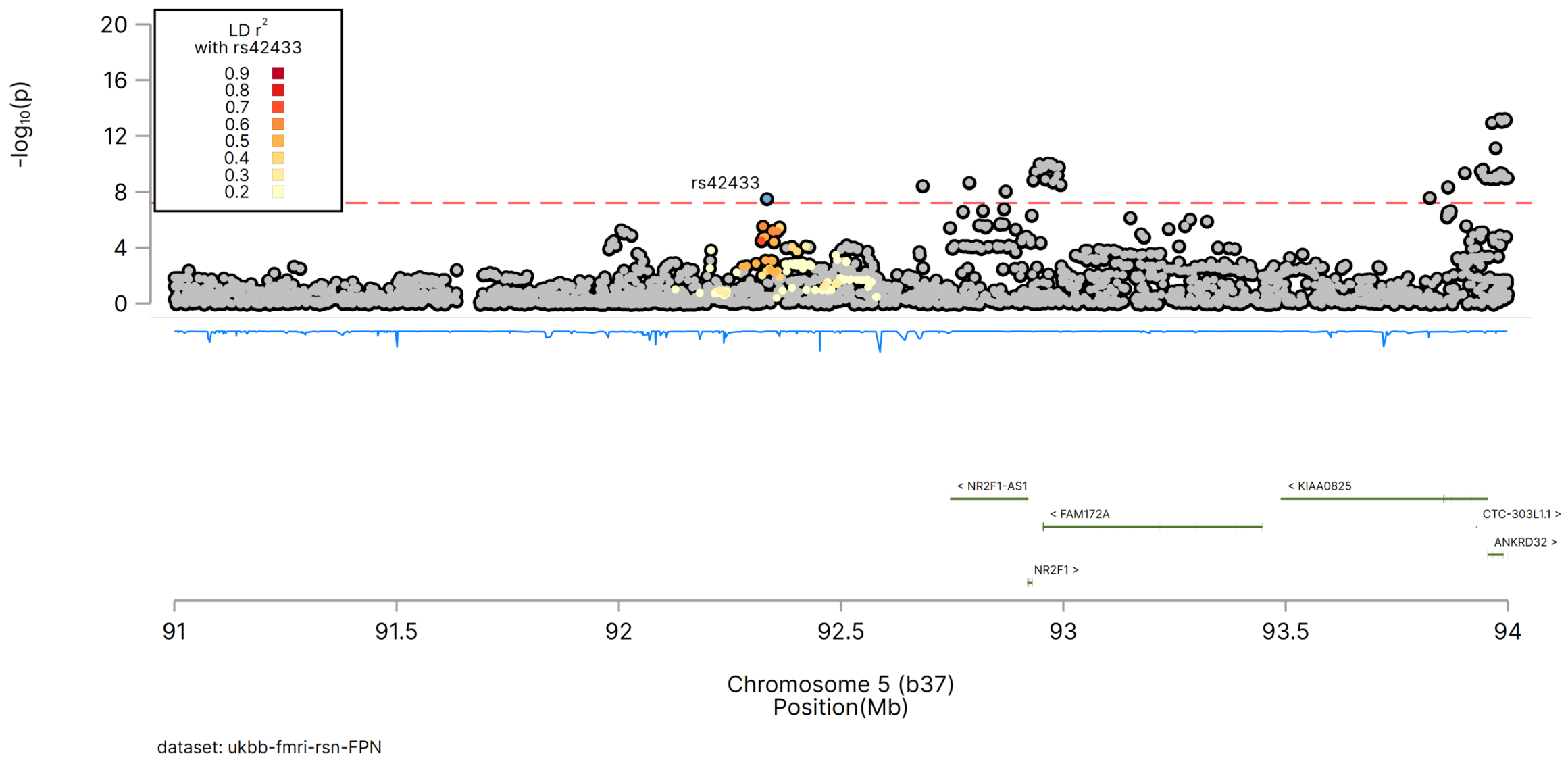

LD r²
with rs42433
0.9
0.8
0.7
0.6
0.5
0.4
0.3
0.2
-log10(p)
20
16
12
8
4
0
rs42433
< NR2F1-AS1
< FAM172A
NR2F1 >
< KIAA0825
CTC-303L1.1 >
ANKRD32 >
91
91.5
92
92.5
93
93.5
94
Chromosome 5 (b37)
Position(Mb)
dataset: ukbb-fmri-rsn-FPN

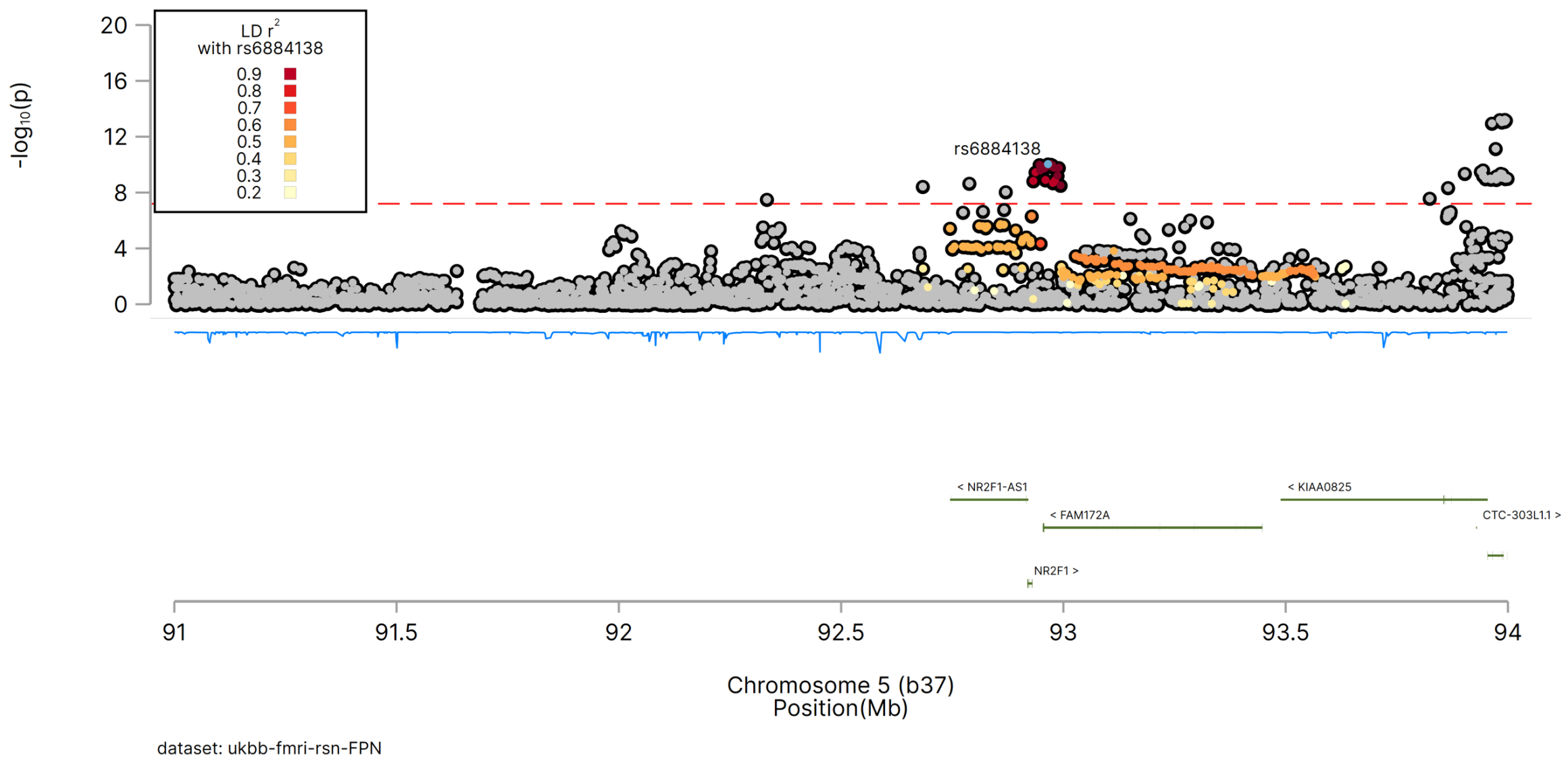

LD r²
with rs6884138
0.9
0.8
0.7
0.6
0.5
0.4
0.3
0.2
-log10(p)
20
16
12
8
4
0
rs6884138
< NR2F1-AS1
< FAM172A
NR2F1 >
< KIAA0825
CTC-303L1.1 >
91
91.5
92
92.5
93
93.5
94
Chromosome 5 (b37)
Position(Mb)
dataset: ukbb-fmri-rsn-FPN

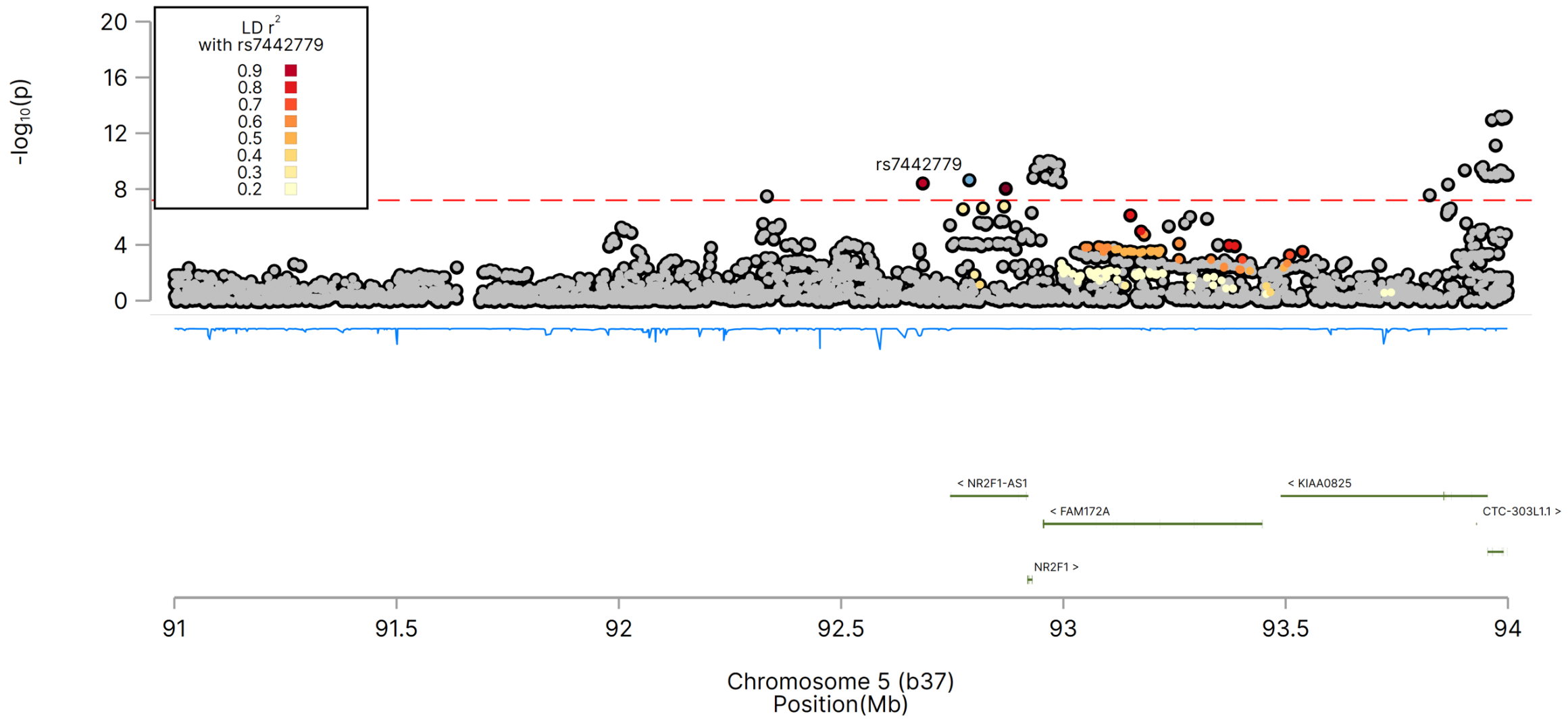

dataset: ukbb-fmri-rsn-FPN

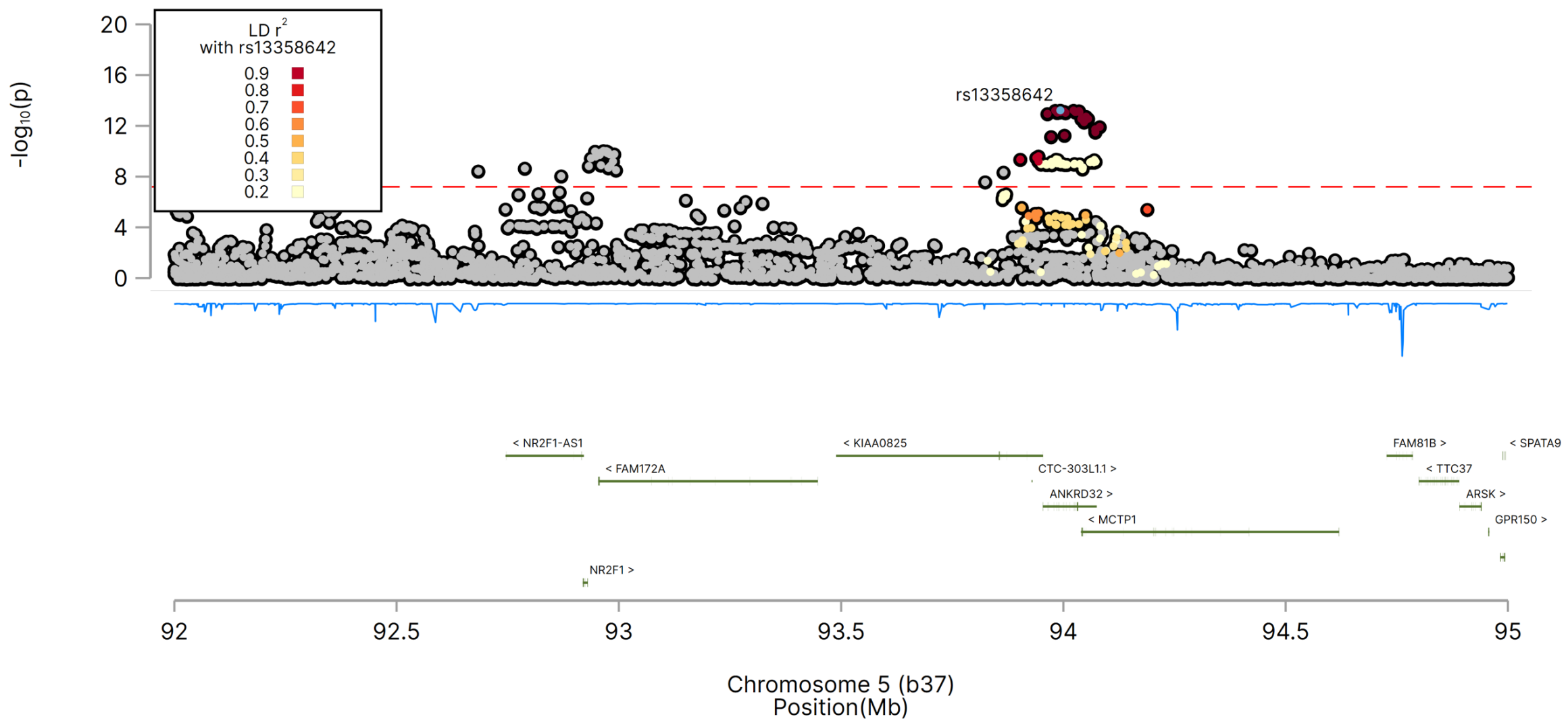

dataset: ukbb-fmri-rsn-FPN

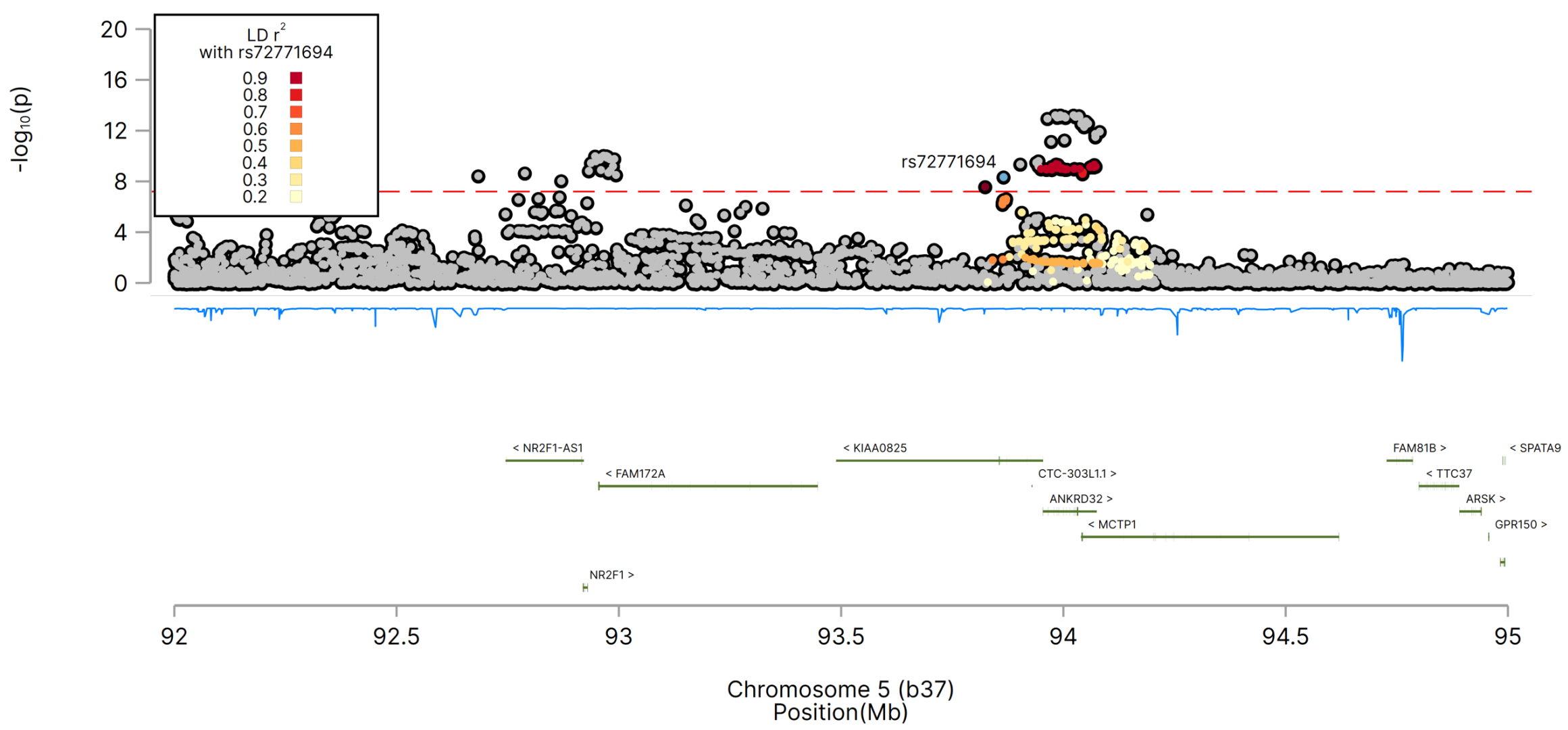

dataset: ukbb-fmri-rsn-FPN

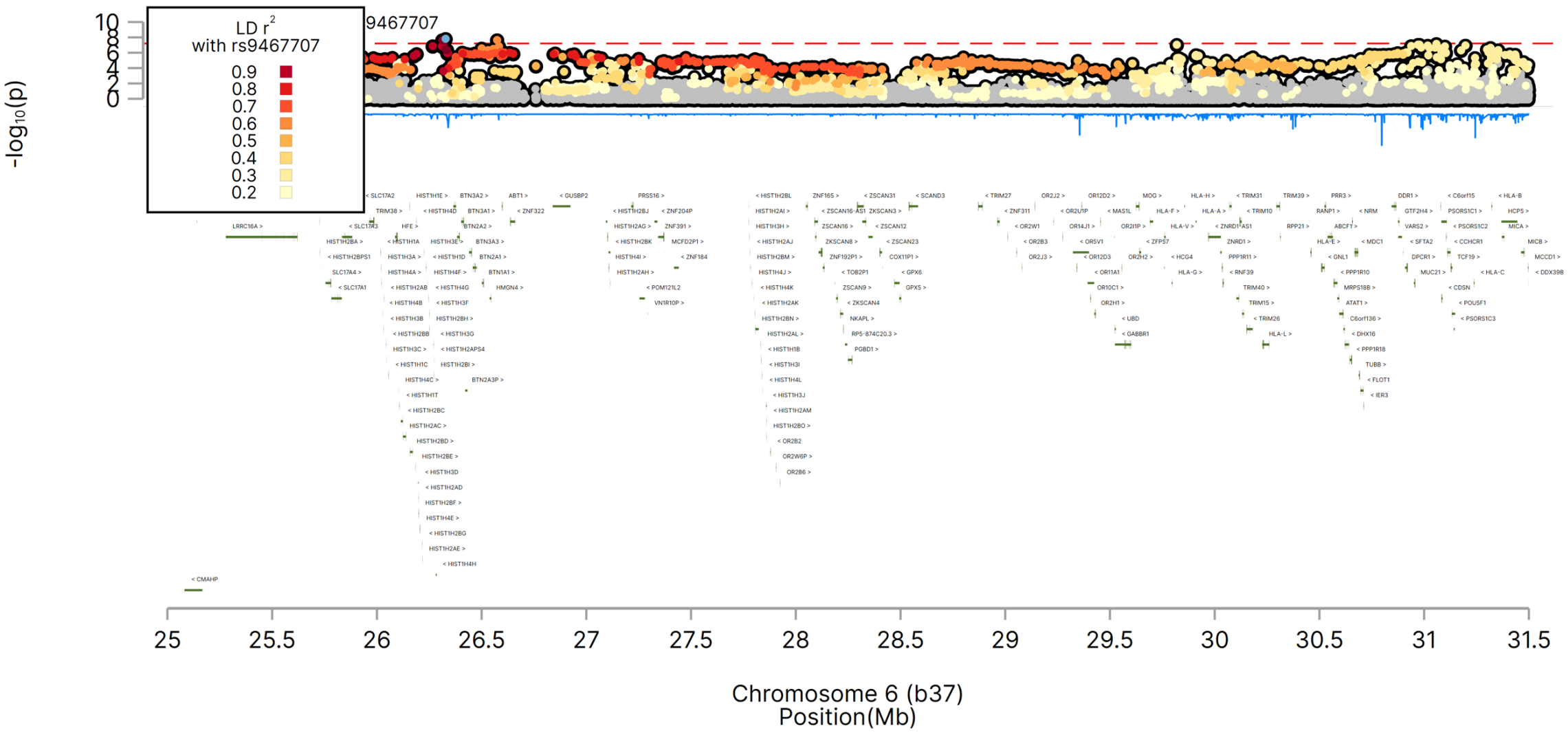

dataset: ukbb-fmri-rsn-FPN

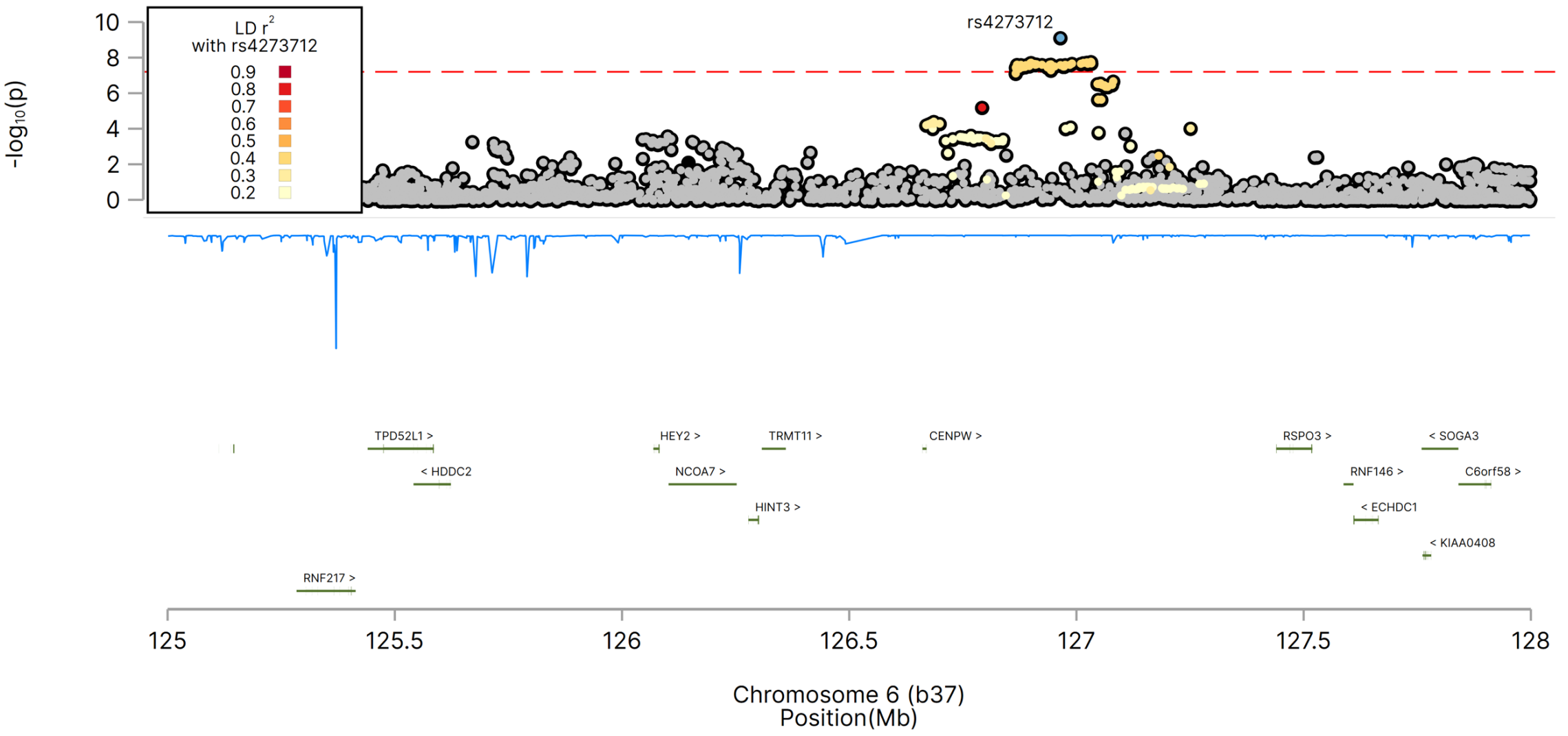

dataset: ukbb-fmri-rsn-FPN

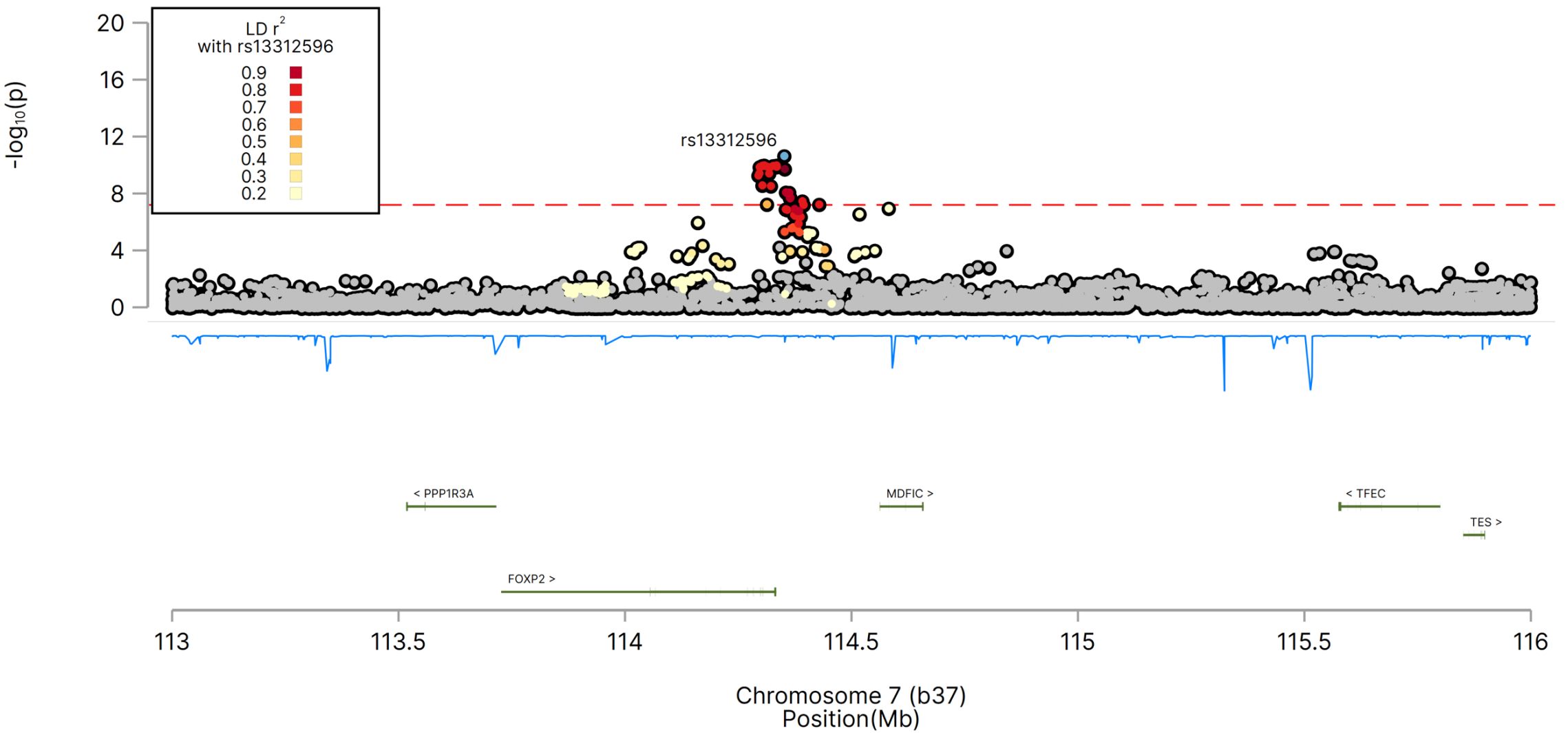

dataset: ukbb-fmri-rsn-FPN

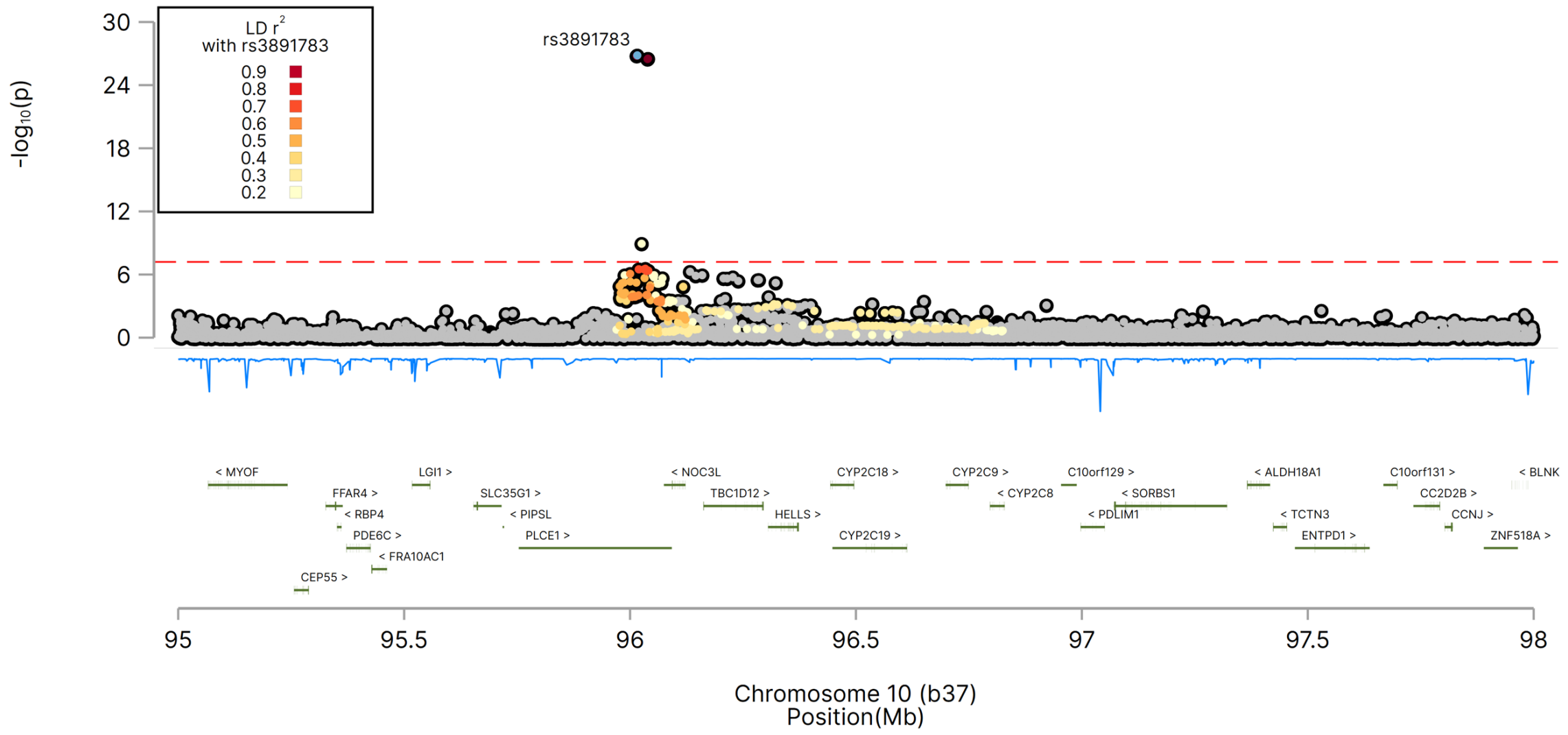

dataset: ukbb-fmri-rsn-FPN

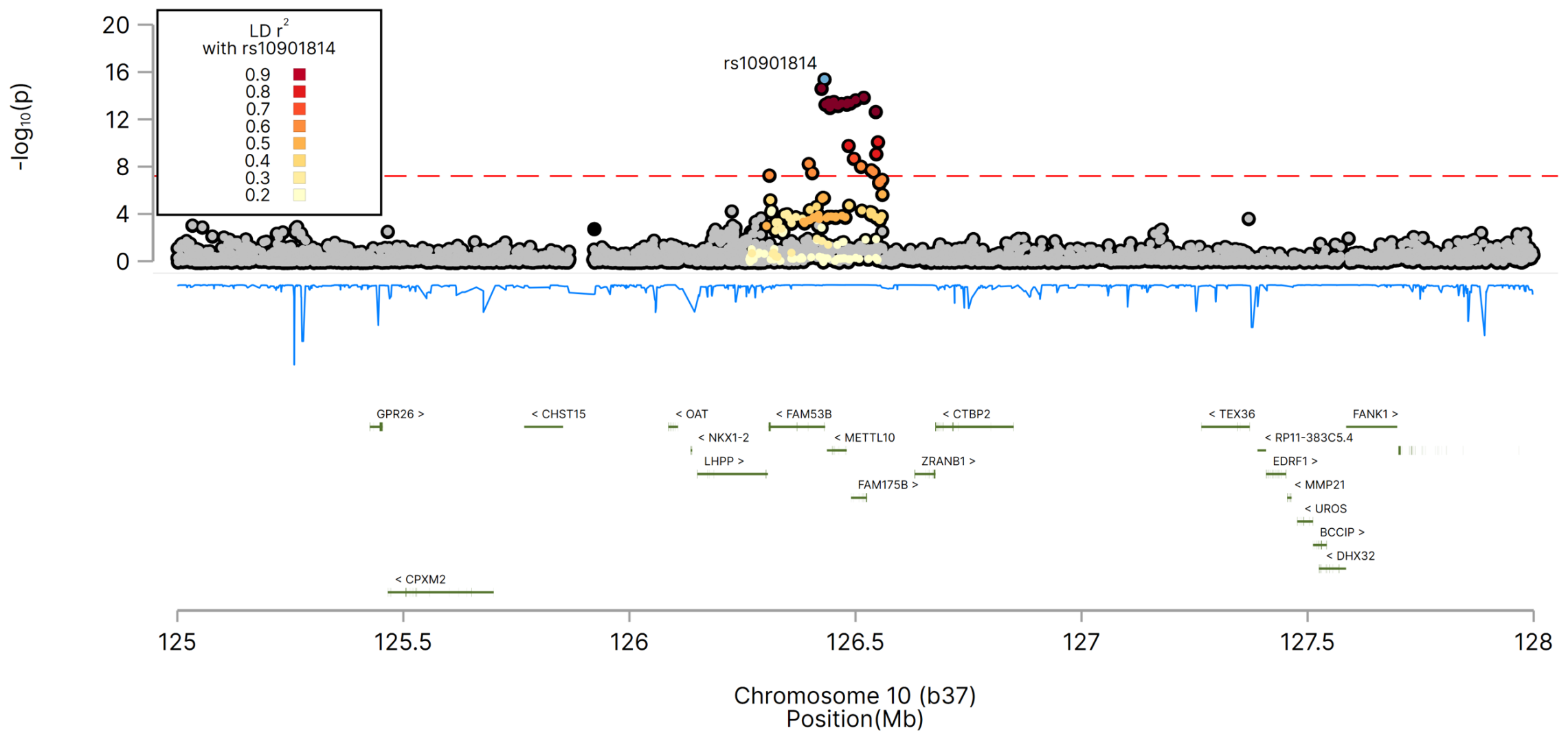

dataset: ukbb-fmri-rsn-FPN

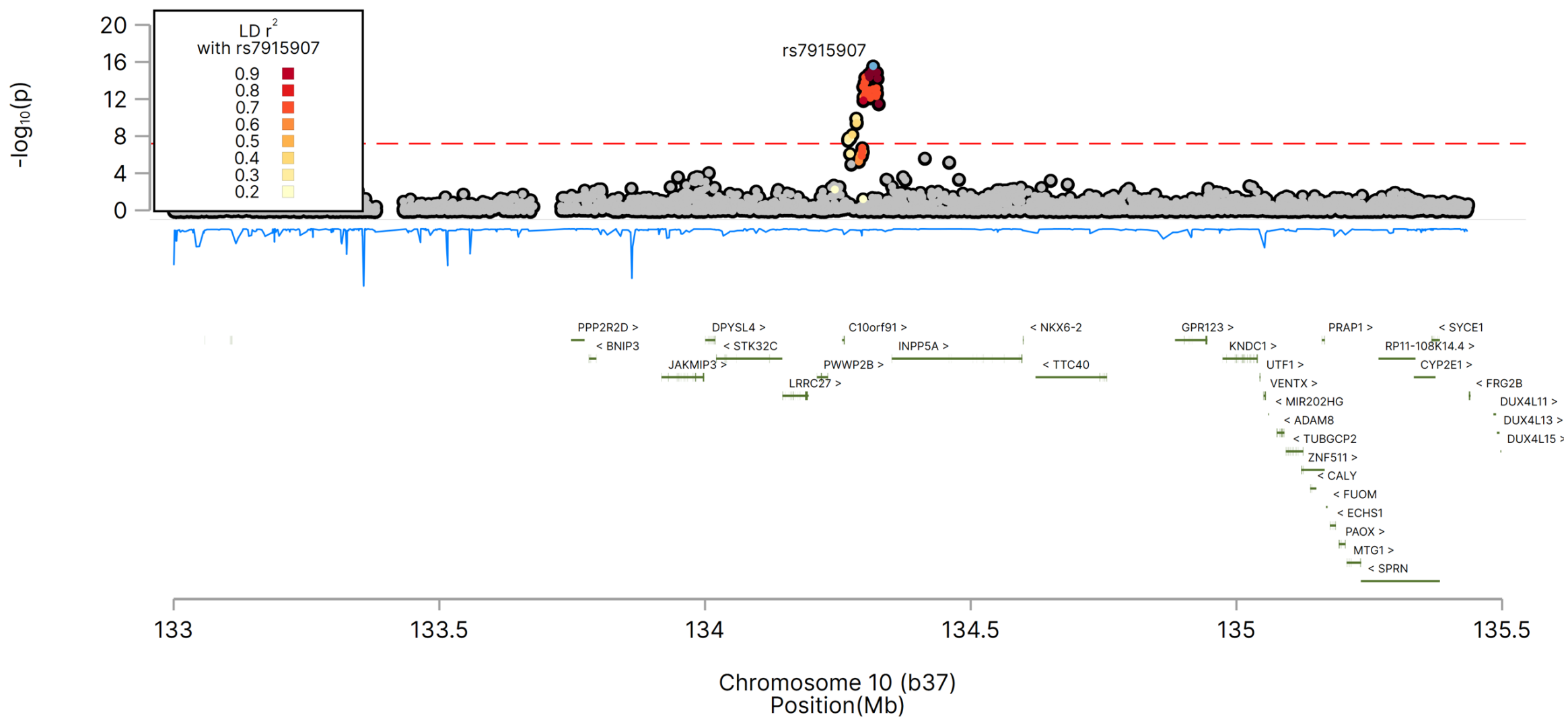

dataset: ukbb-fmri-rsn-FPN

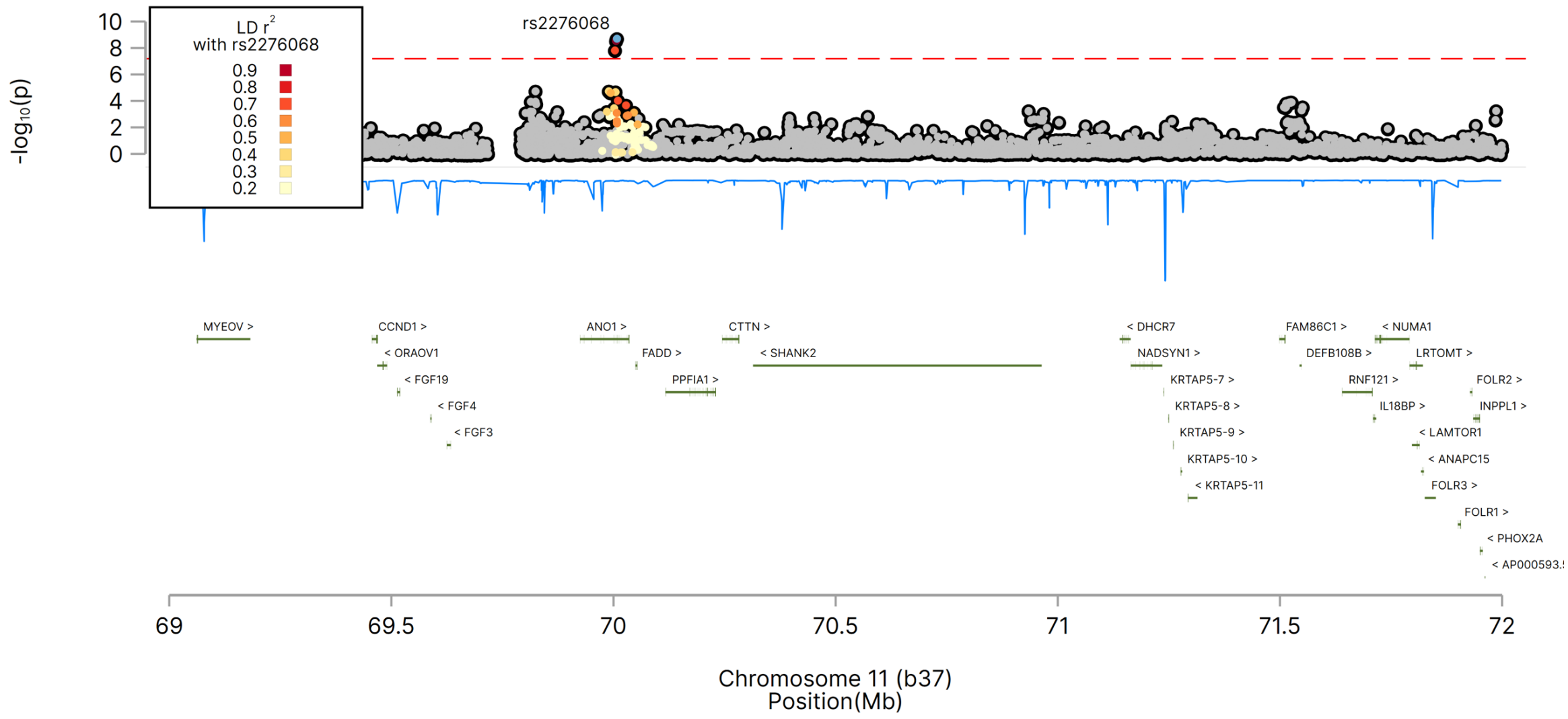

dataset: ukbb-fmri-rsn-FPN

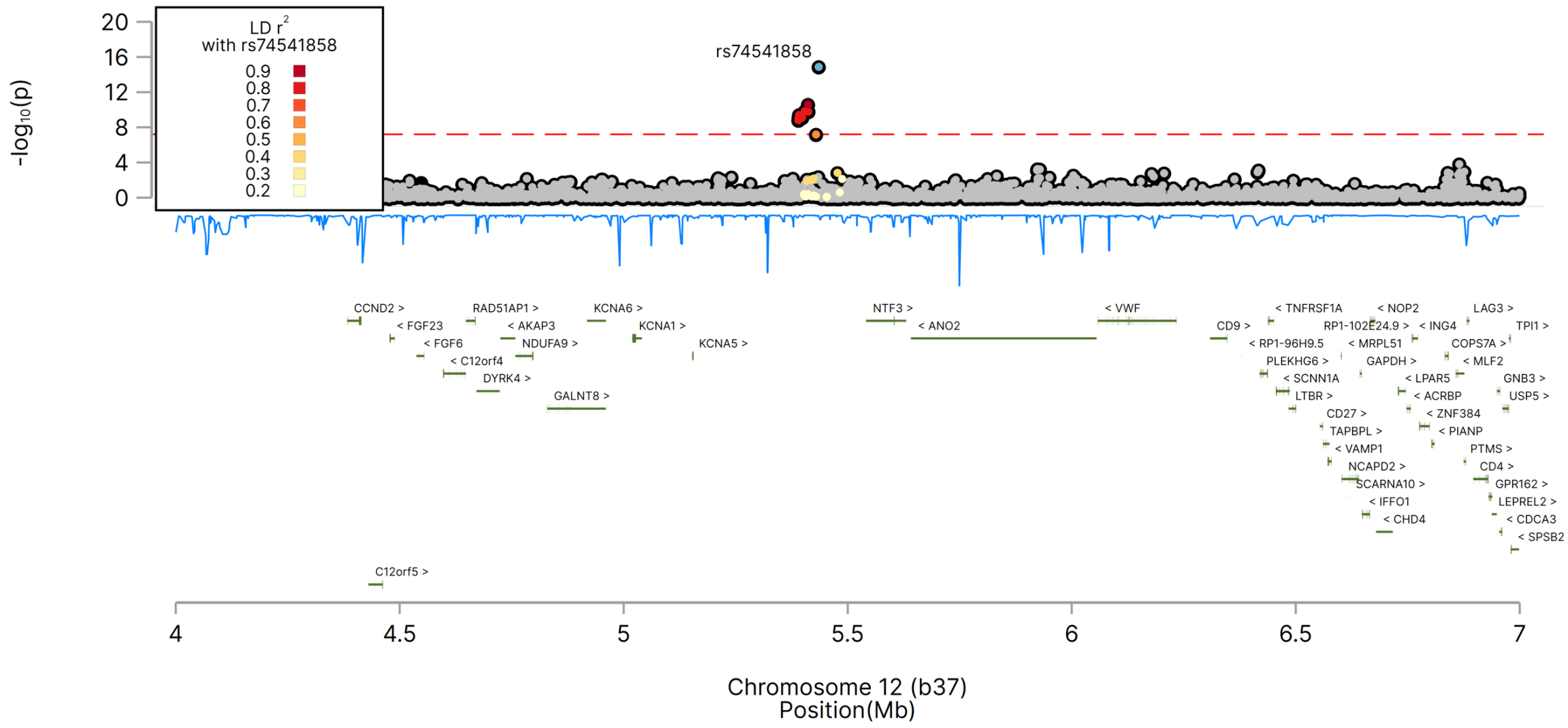

dataset: ukbb-fmri-rsn-FPN

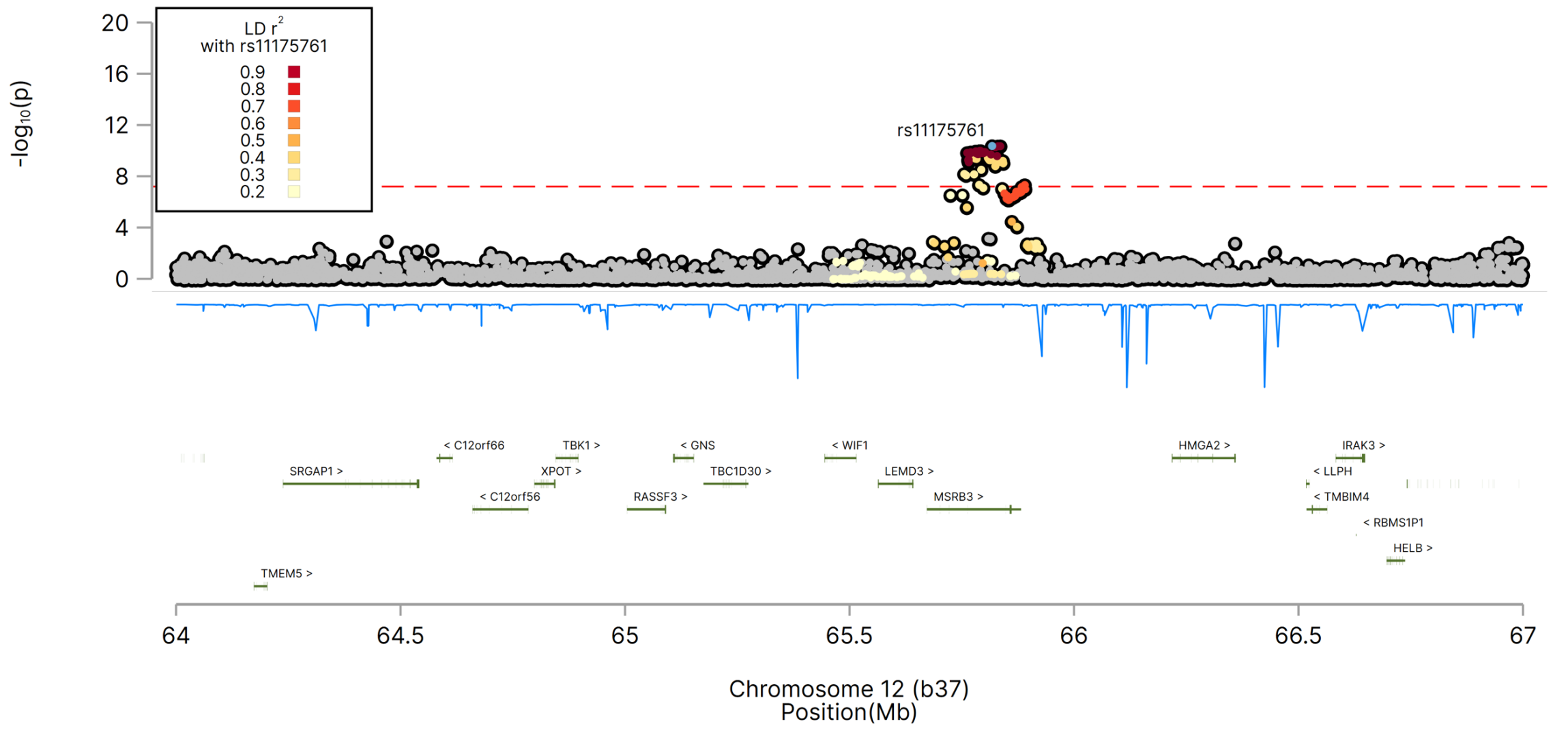

dataset: ukbb-fmri-rsn-FPN

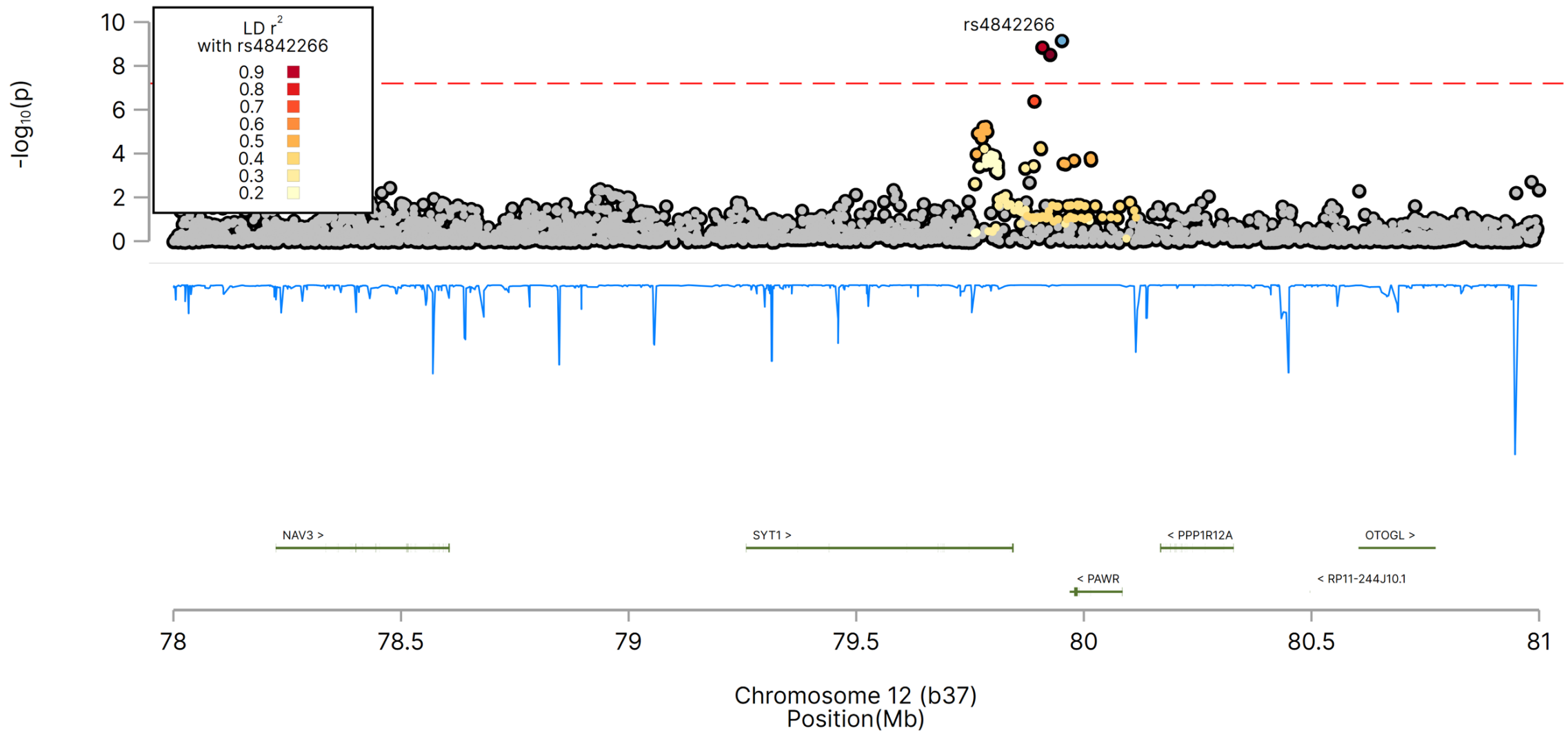

dataset: ukbb-fmri-rsn-FPN

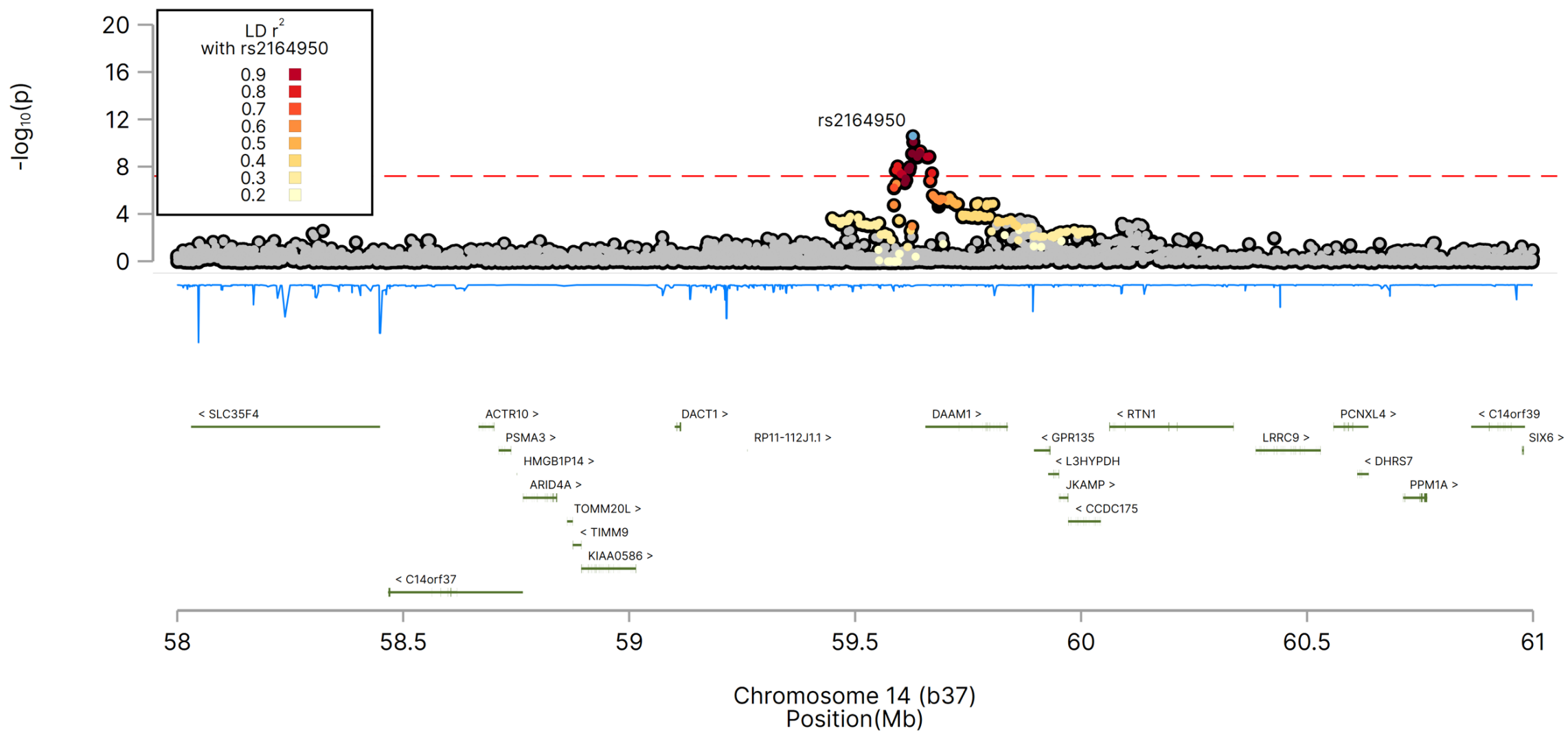

dataset: ukbb-fmri-rsn-FPN

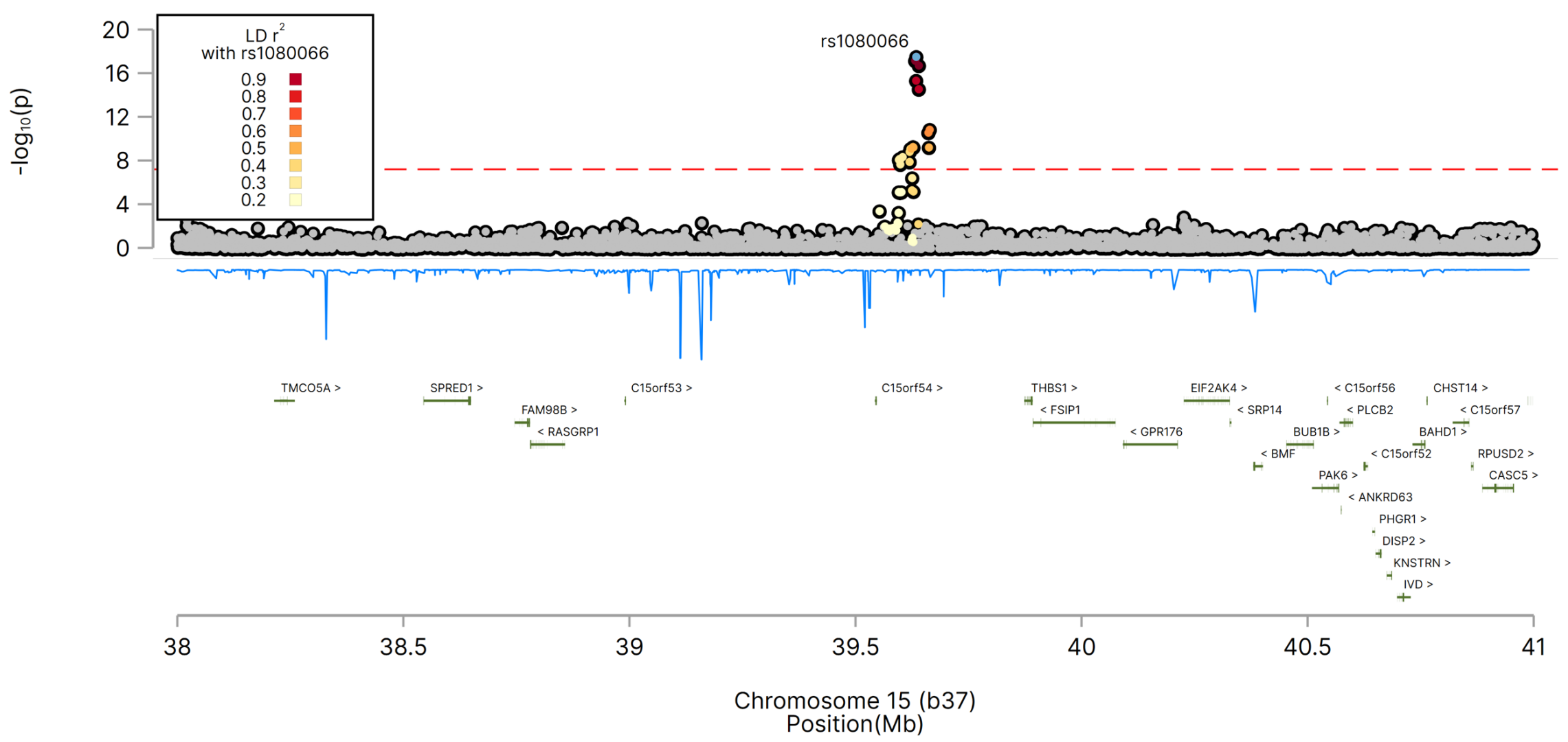

dataset: ukbb-fmri-rsn-FPN

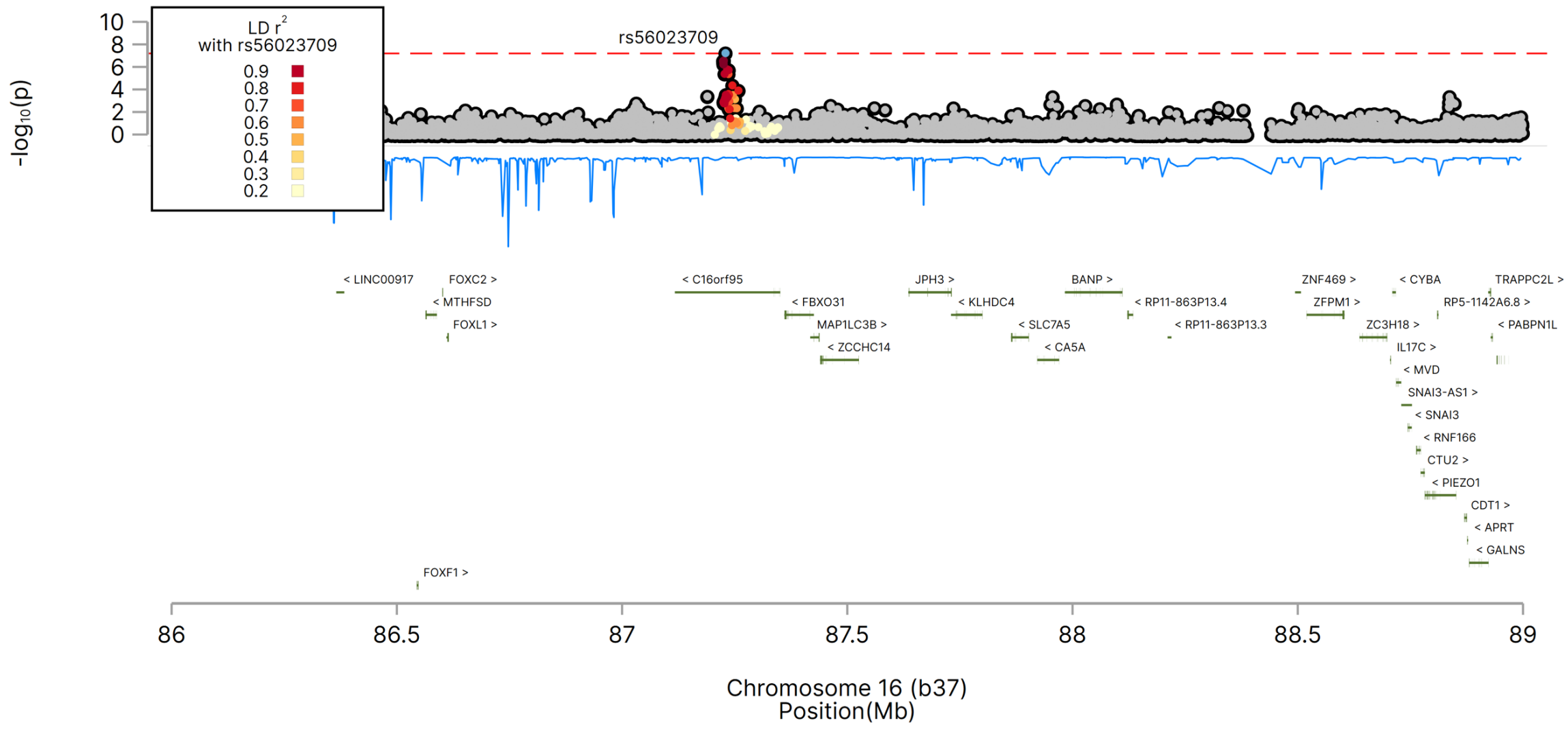

dataset: ukbb-fmri-rsn-FPN

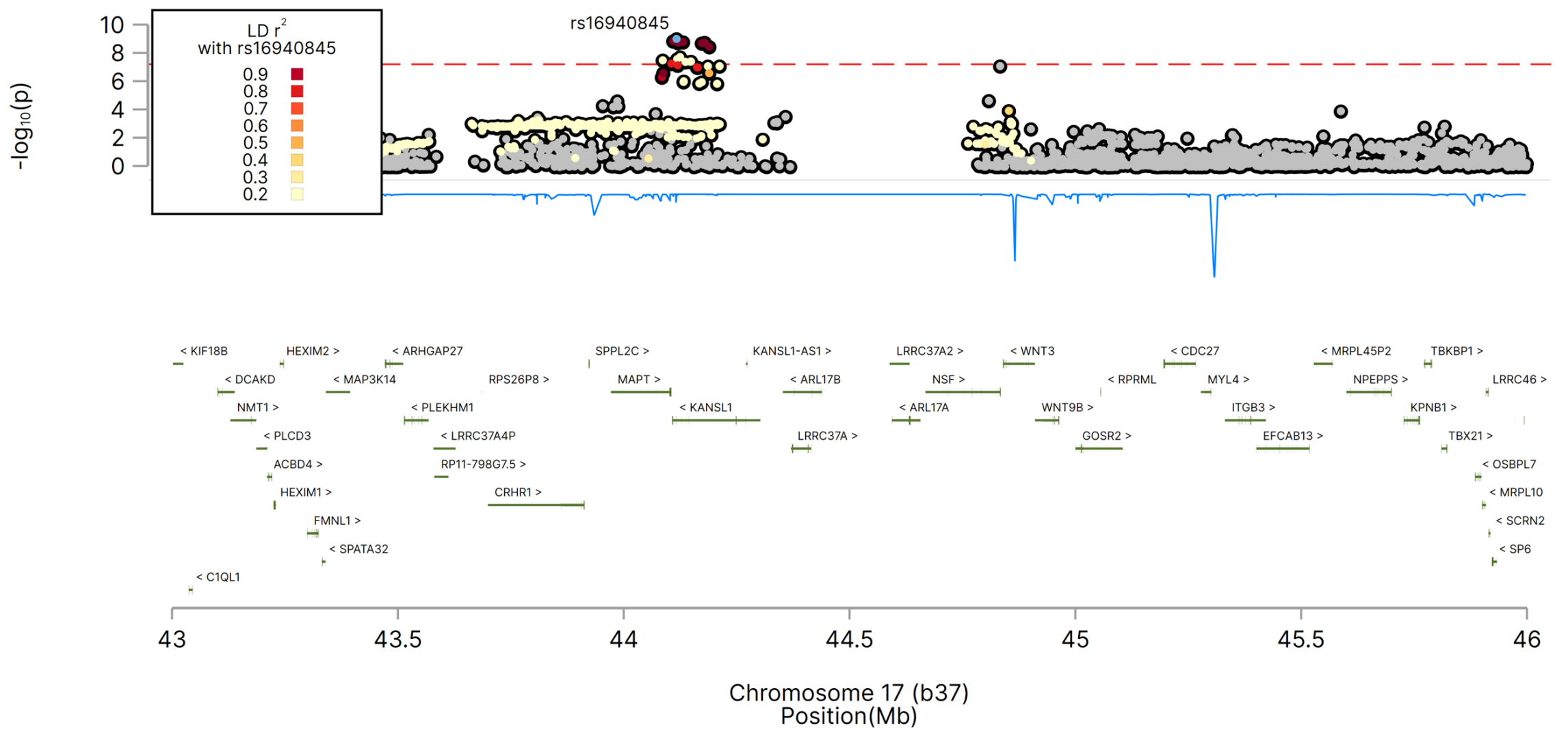

dataset: ukbb-fmri-rsn-FPN

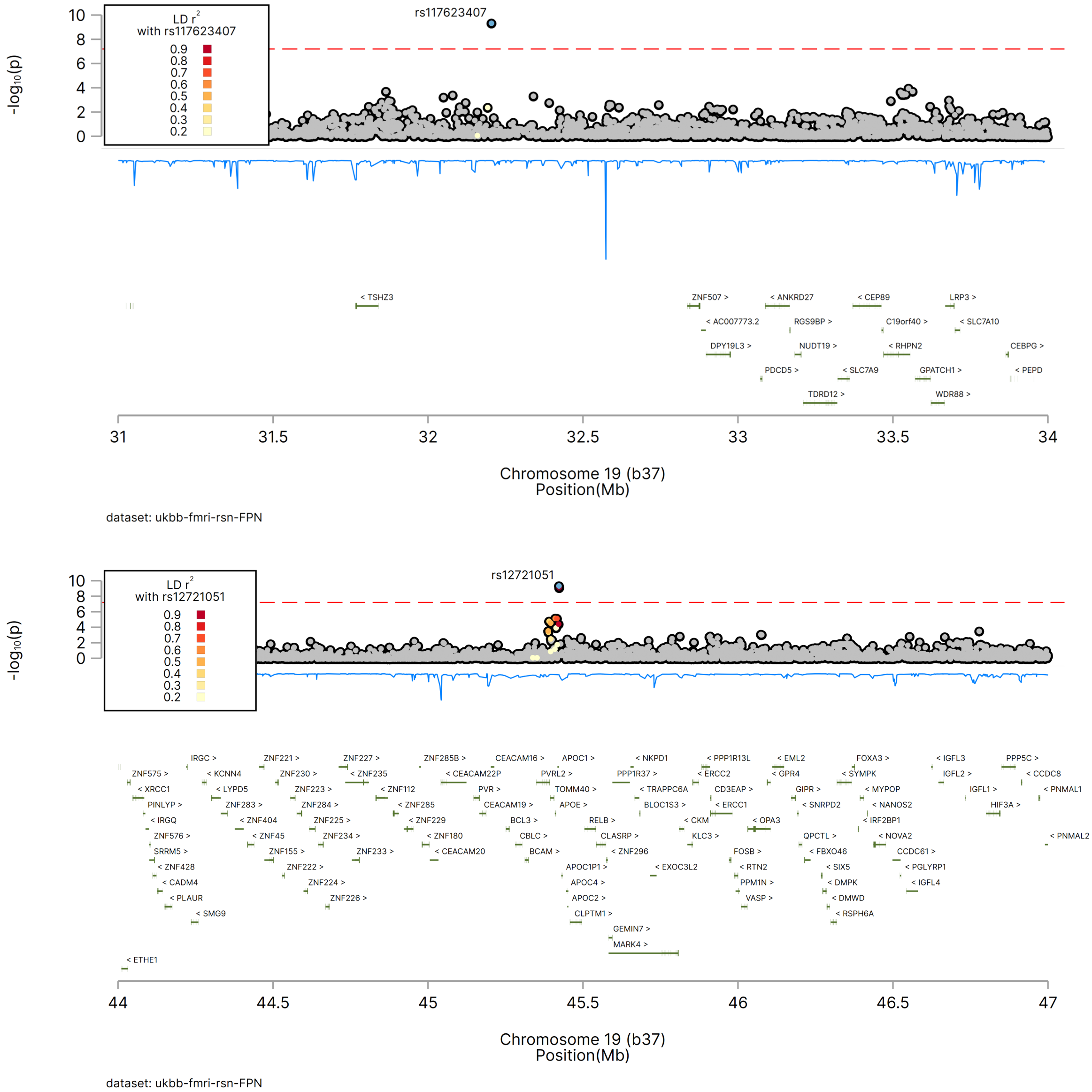

Supplementary Figure 5. Locus-zoom for all significant LD-independent signals coming from the MOST GWAS test for the Fronto-Parietal Network (FPN). Source data for this figure can be obtained from https://www.ebi.ac.uk/gwas/; hyperlinks to the data for each network are provided in the Data Availability section.

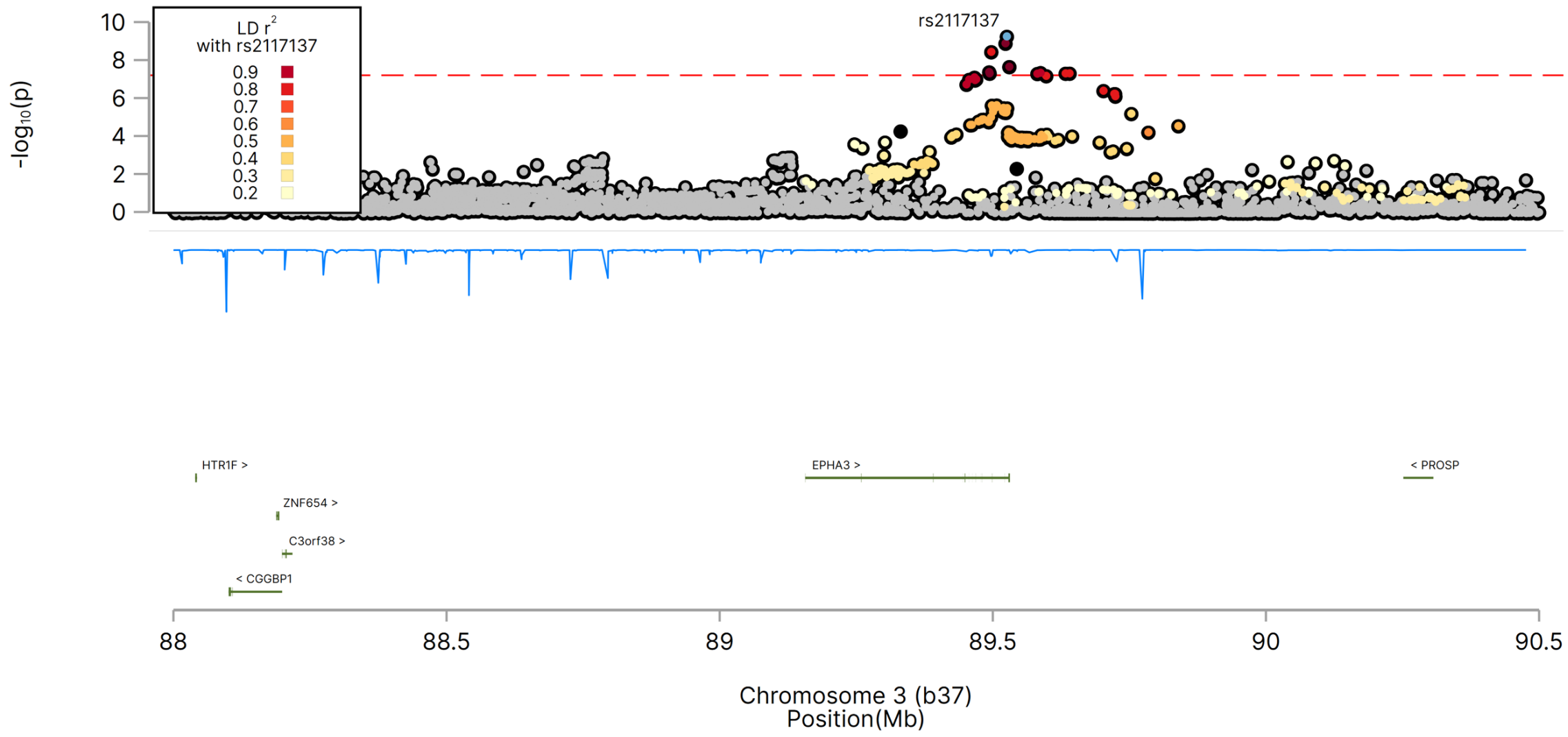

dataset: ukbb-fmri-rsn-LM

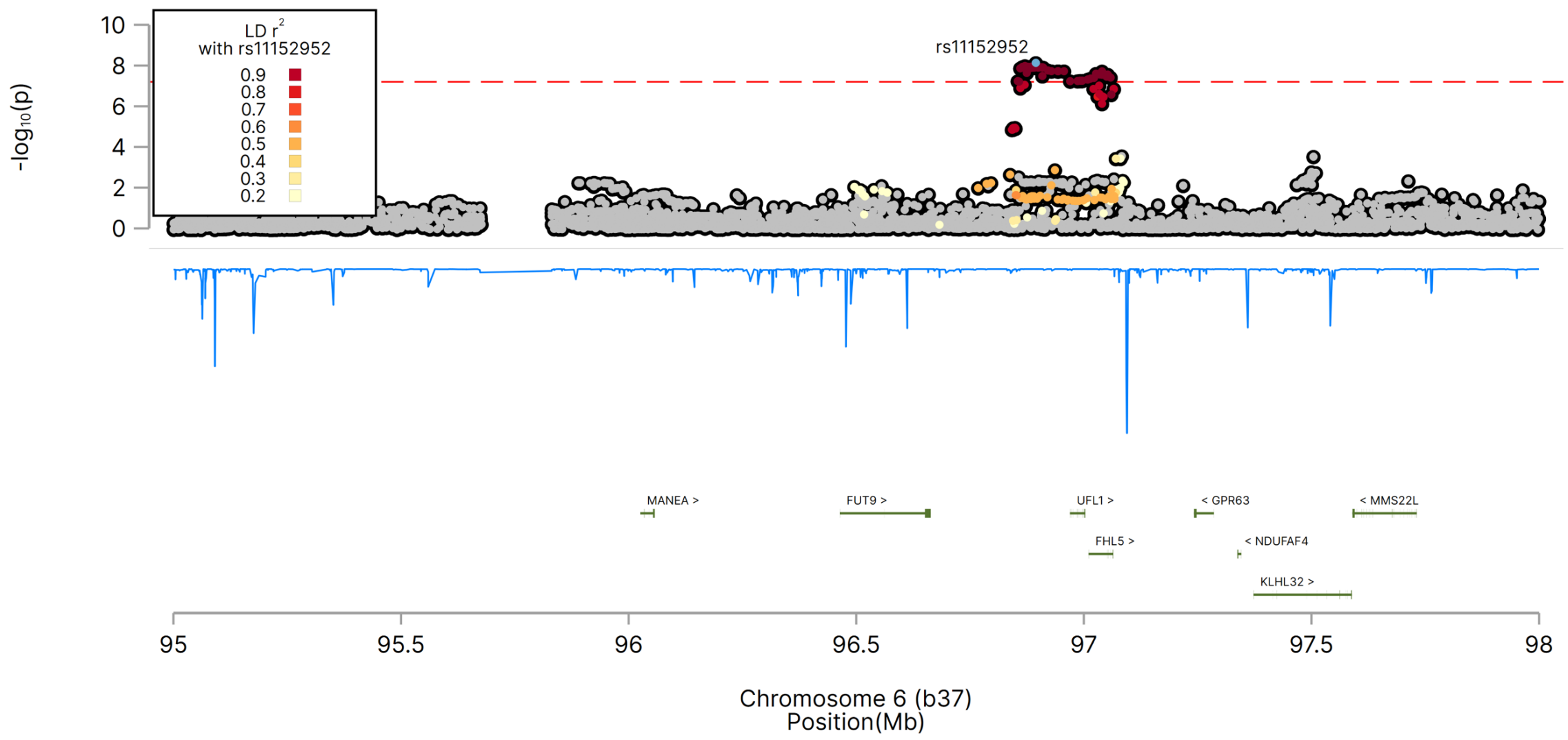

dataset: ukbb-fmri-rsn-LM

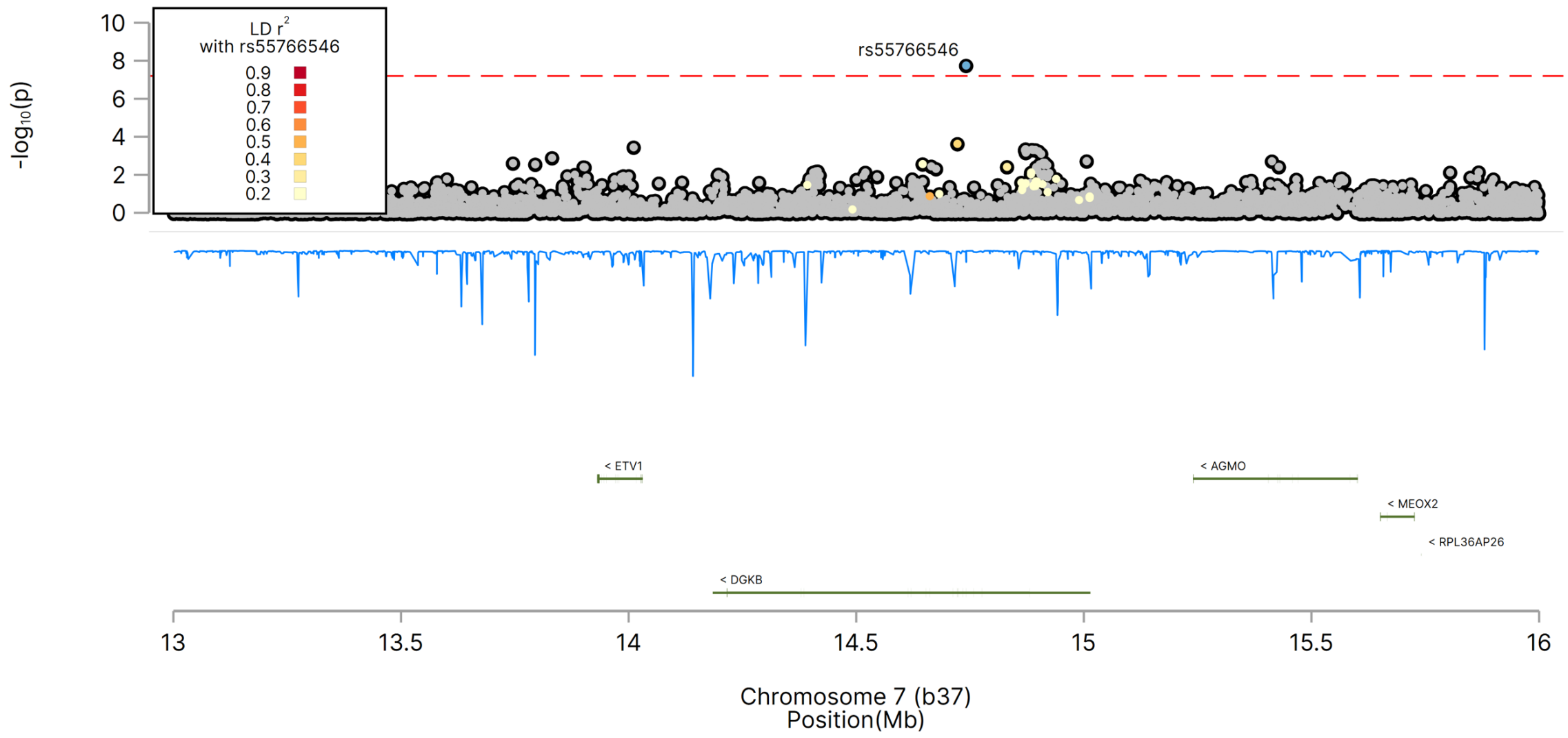

dataset: ukbb-fmri-rsn-LM

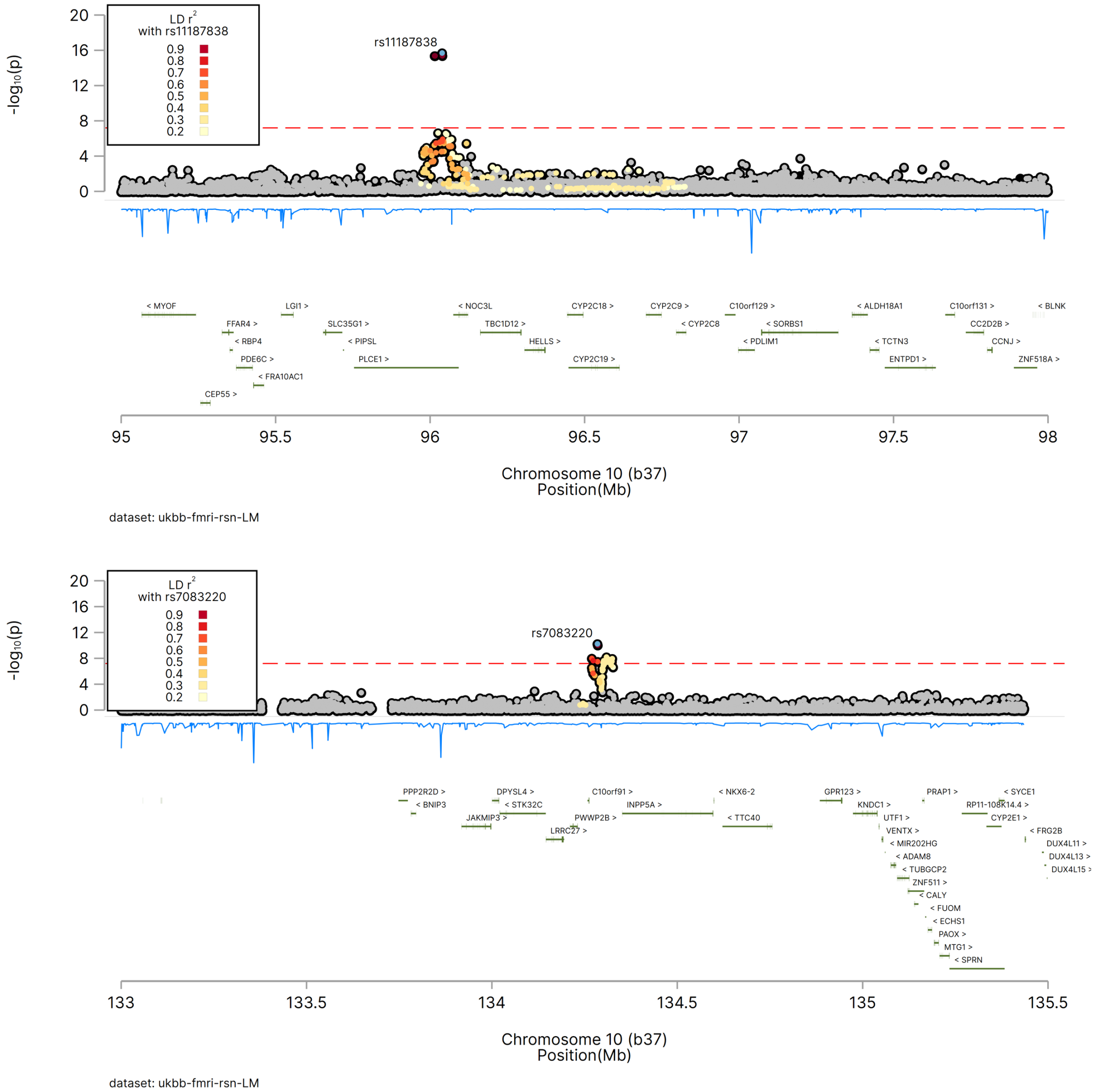

Supplementary Figure 6. Locus-zoom for all significant LD-independent signals coming from the MOST GWAS test for the Limbic Network (LN). Source data for this figure can be obtained from https://www.ebi.ac.uk/gwas/; hyperlinks to the data for each network are provided in the Data Availability section.

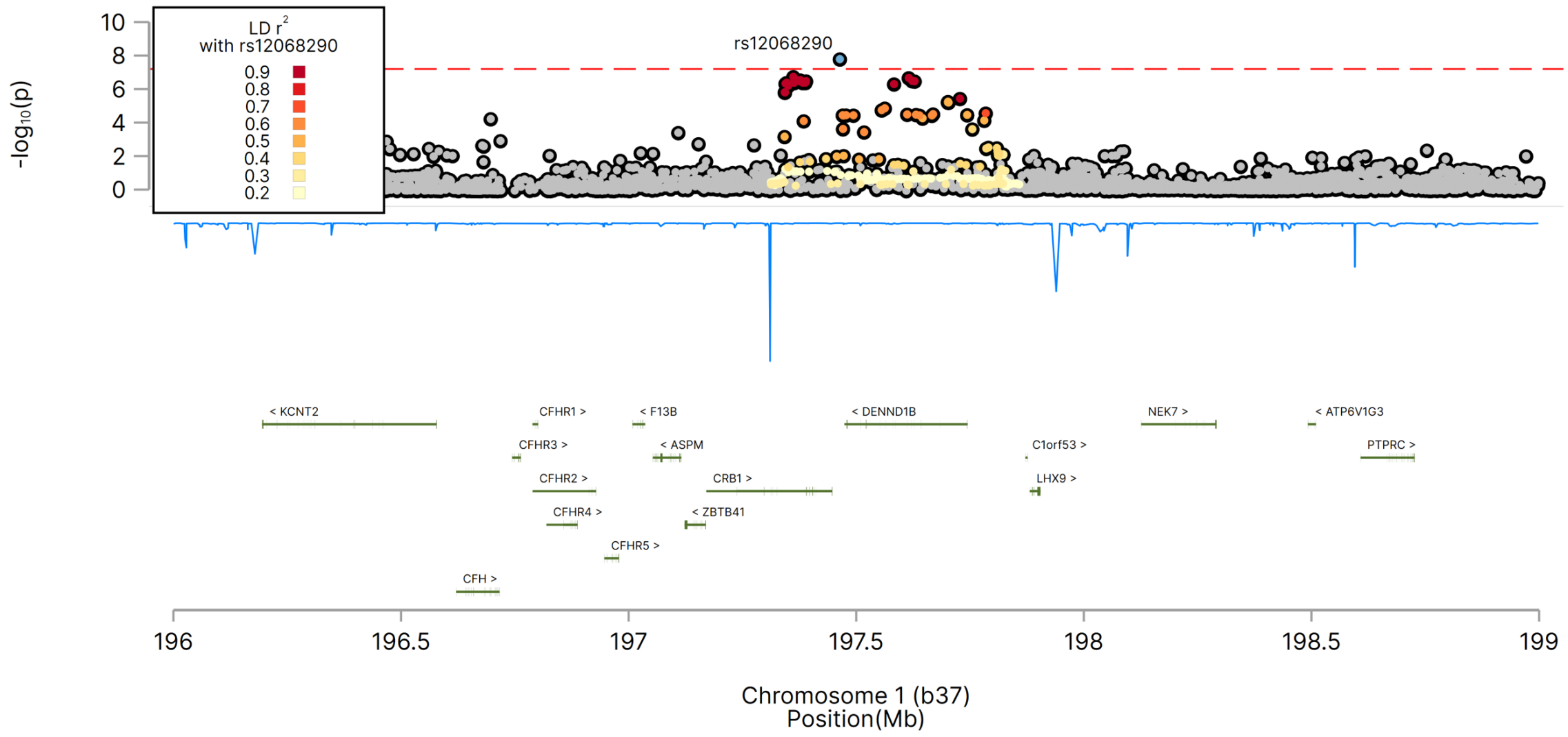

dataset: ukbb-fmri-rsn-SMN

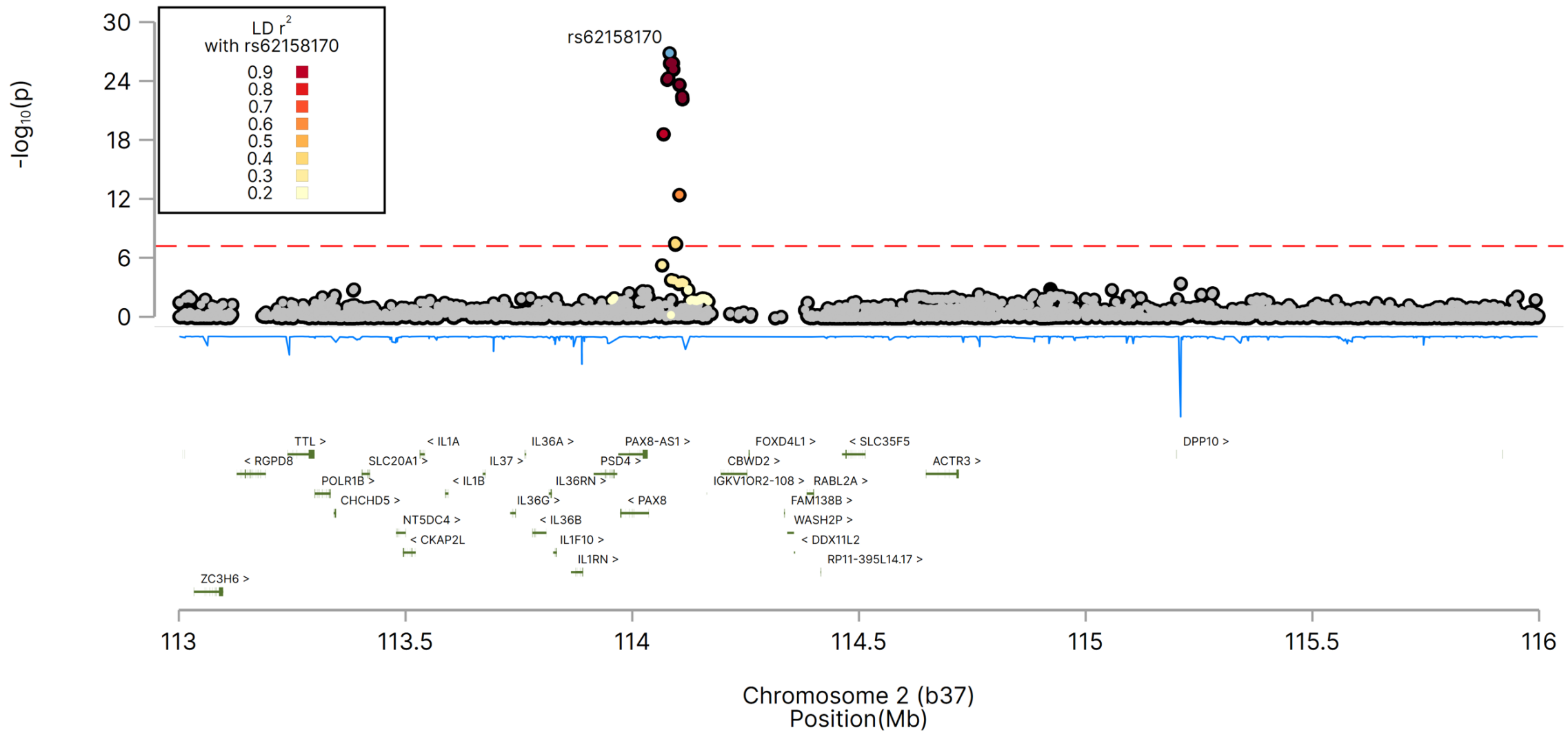

dataset: ukbb-fmri-rsn-SMN

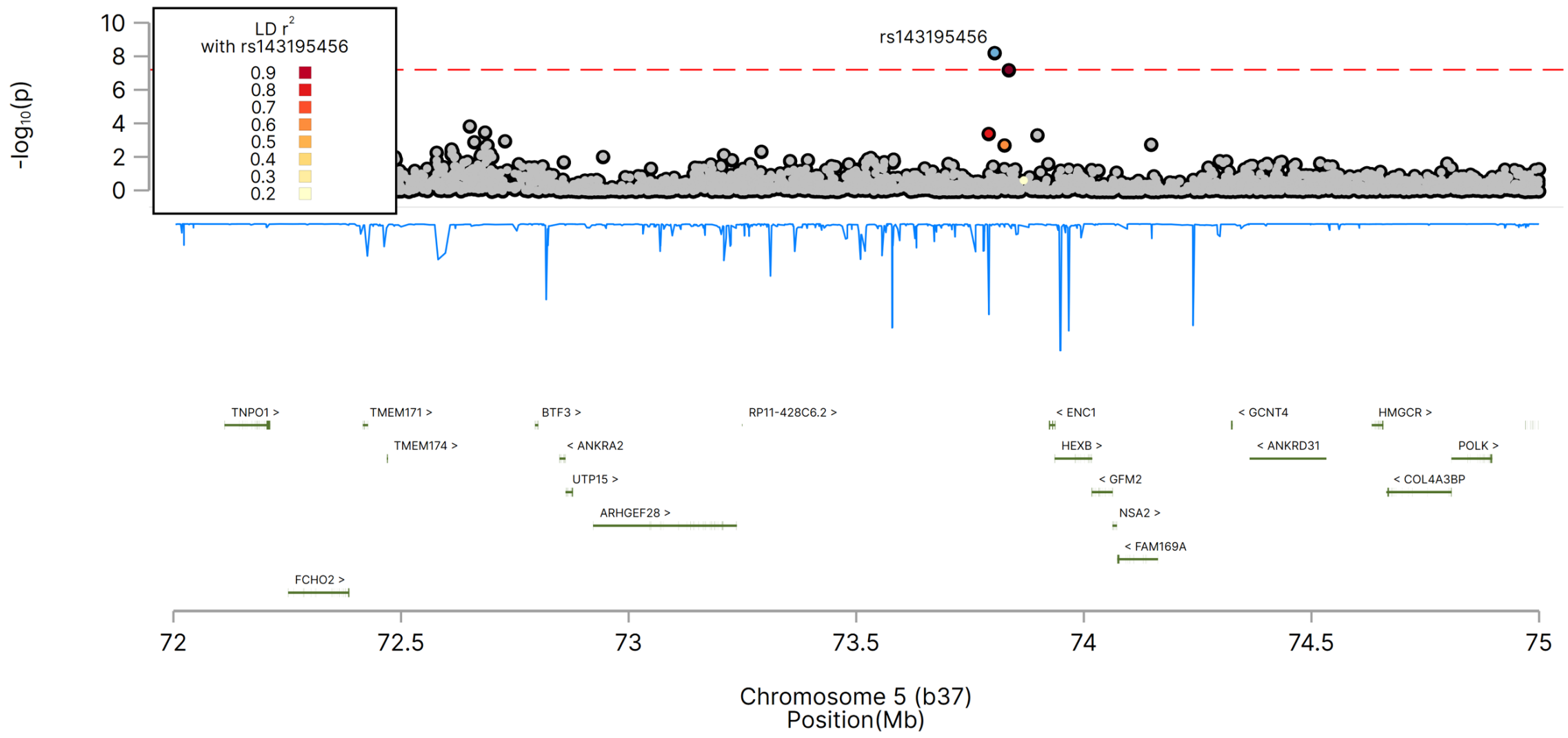

dataset: ukbb-fmri-rsn-SMN

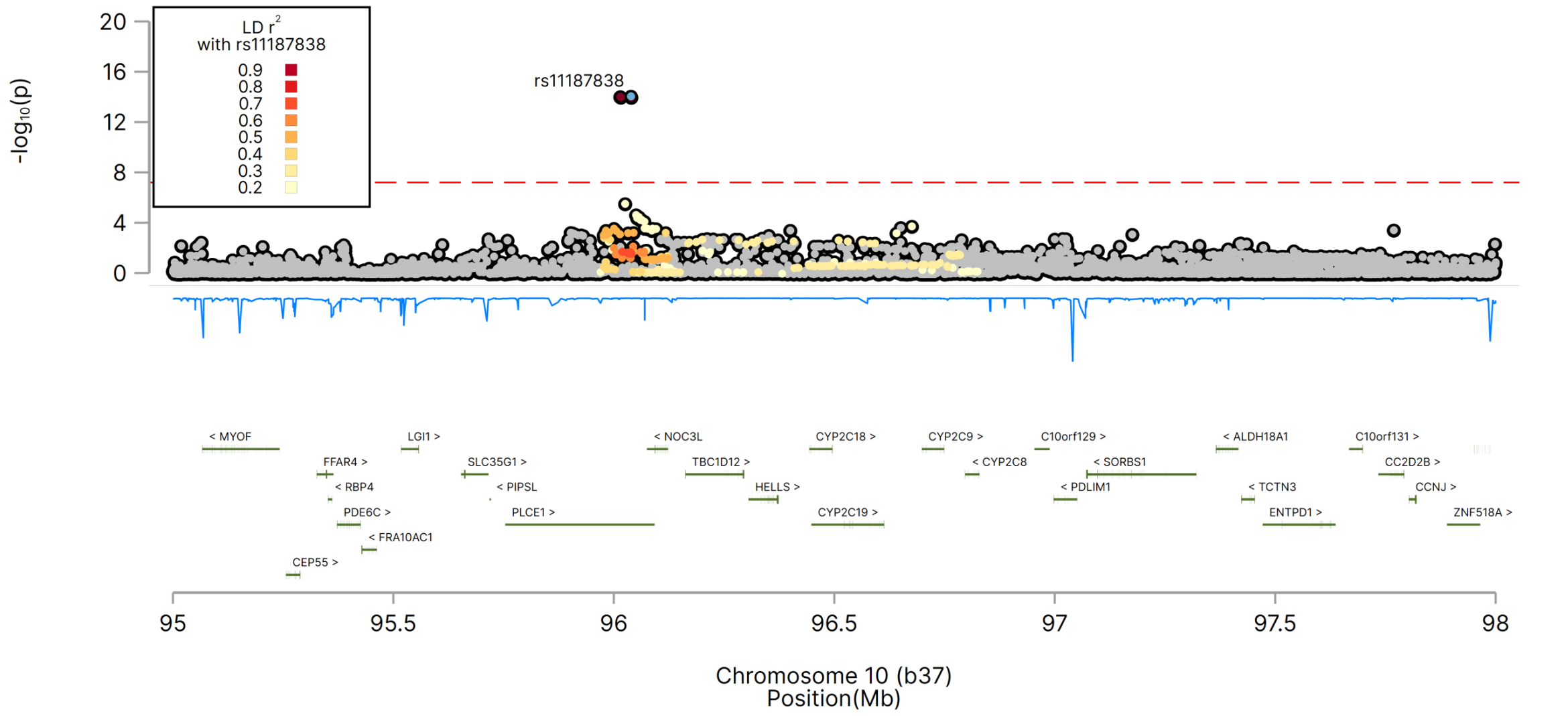

dataset: ukbb-fmri-rsn-SMN

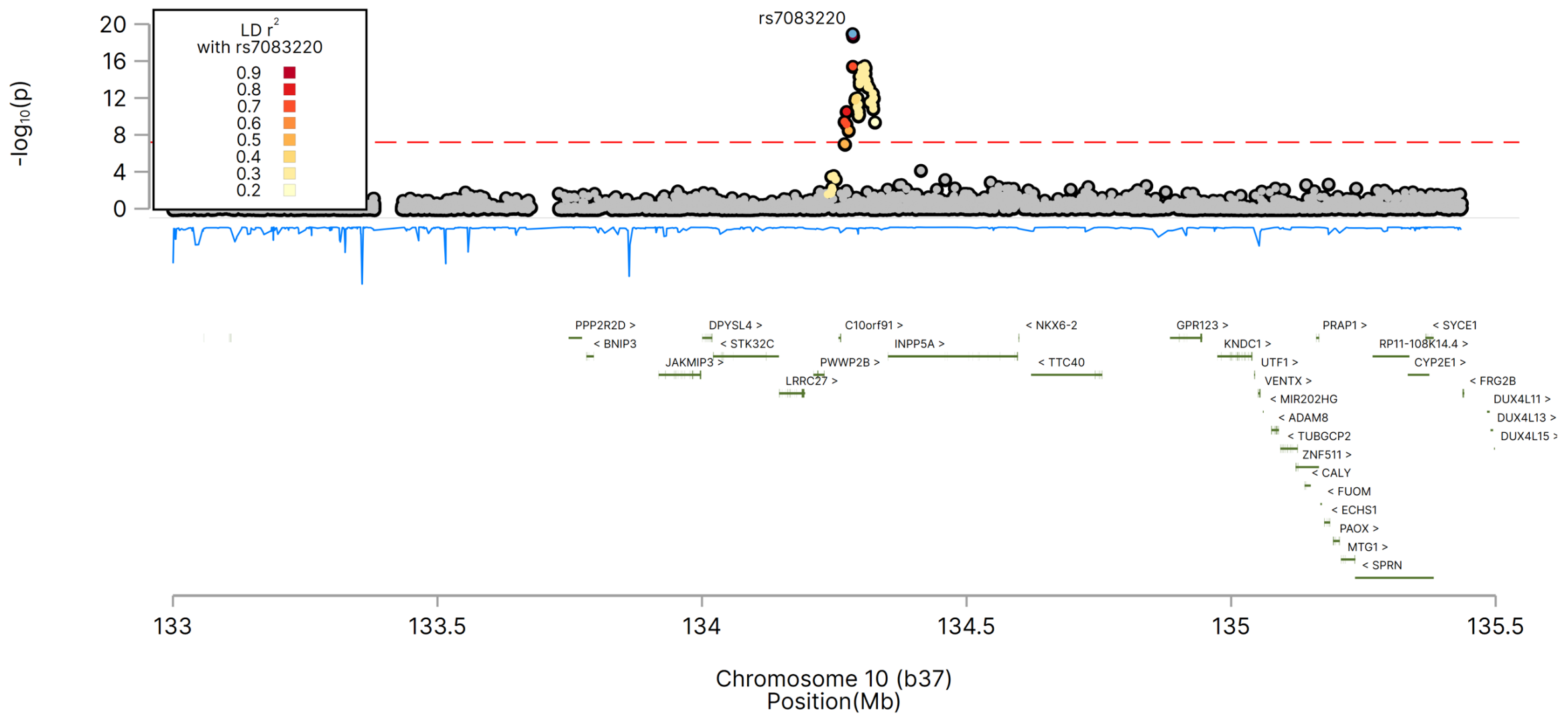

dataset: ukbb-fmri-rsn-SMN

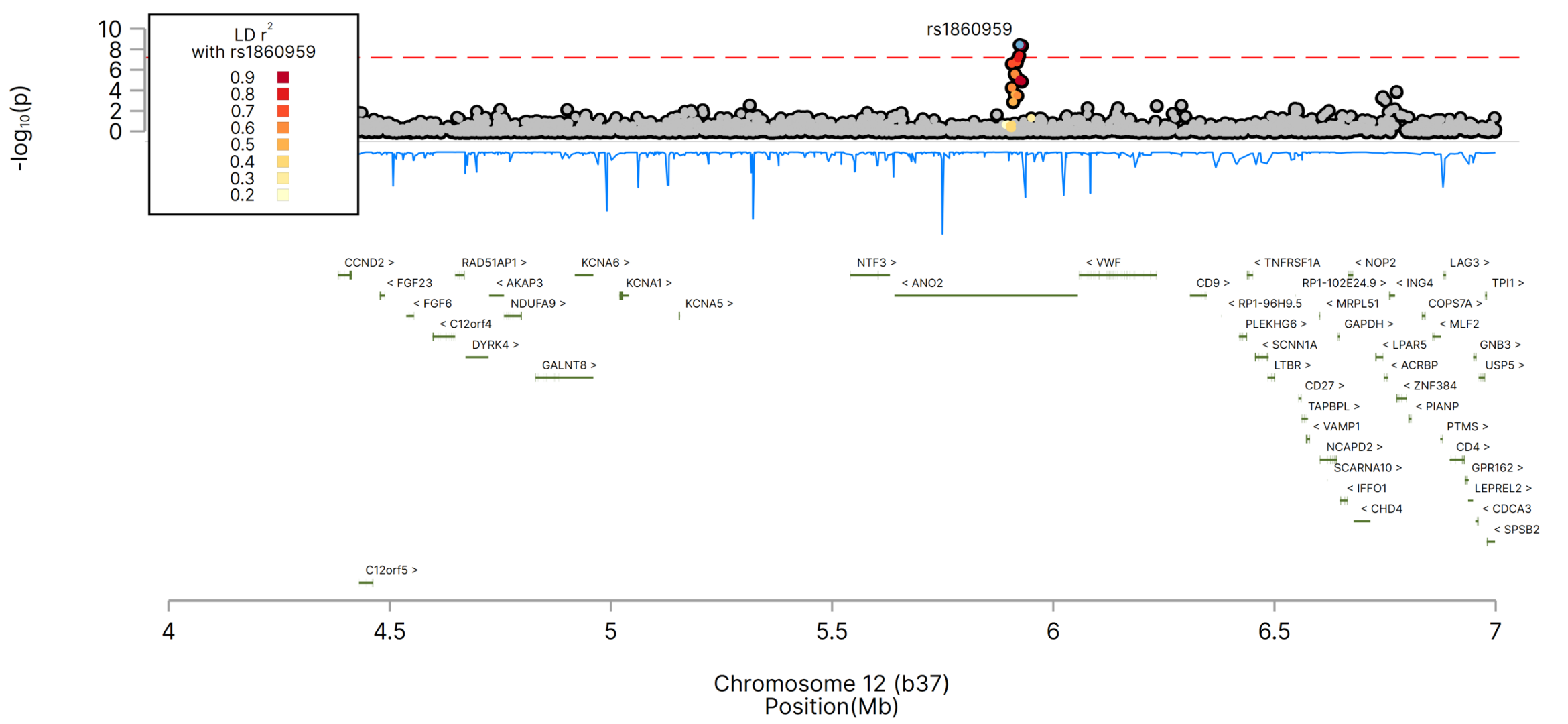

dataset: ukbb-fmri-rsn-SMN

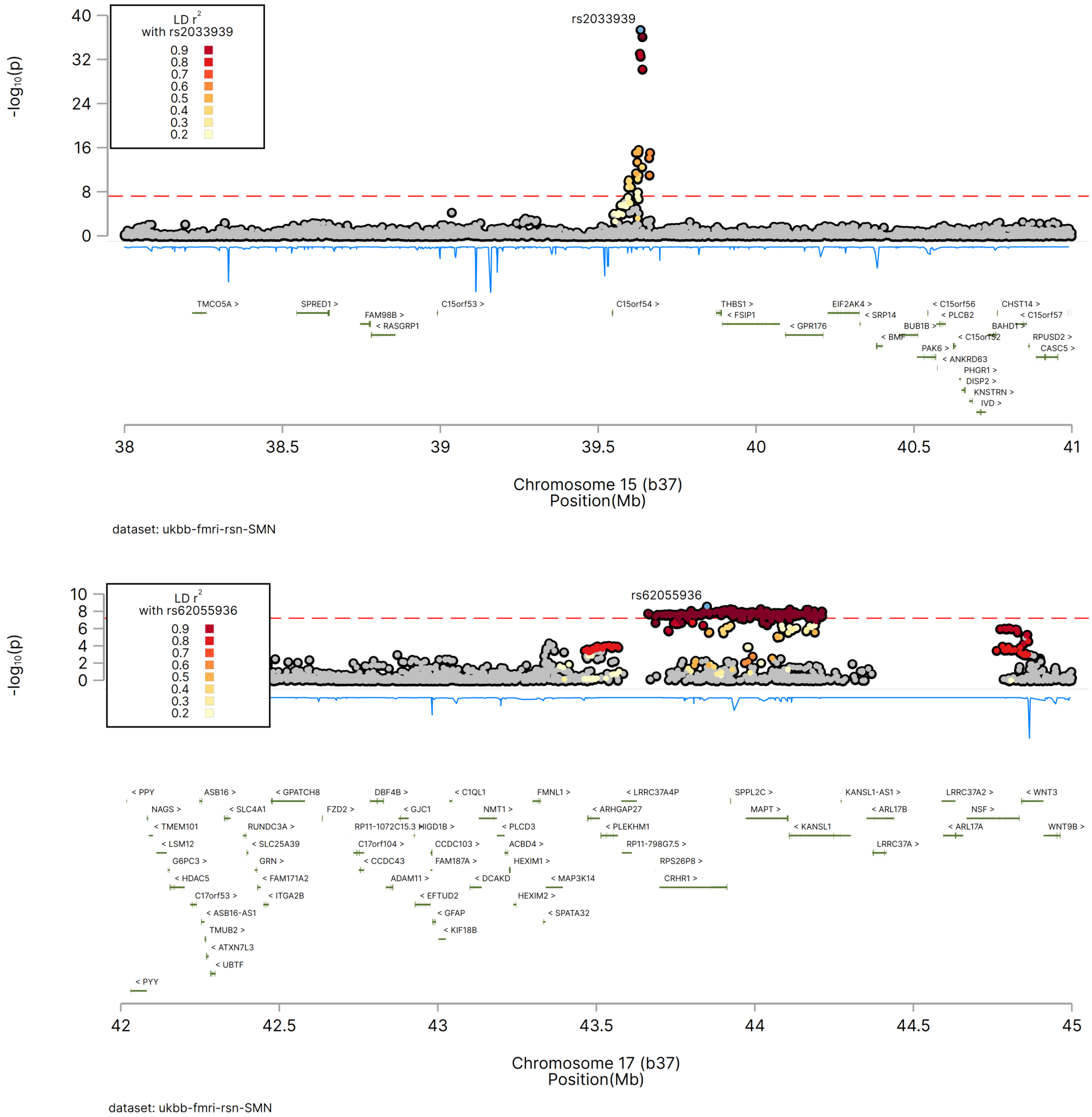

Supplementary Figure 7. Locus-zoom for all significant LD-independent signals coming from the MOST GWAS test for the Somatosensory Network (SMN). Source data for this figure can be obtained from https://www.ebi.ac.uk/gwas/; hyperlinks to the data for each network are provided in the Data Availability section.

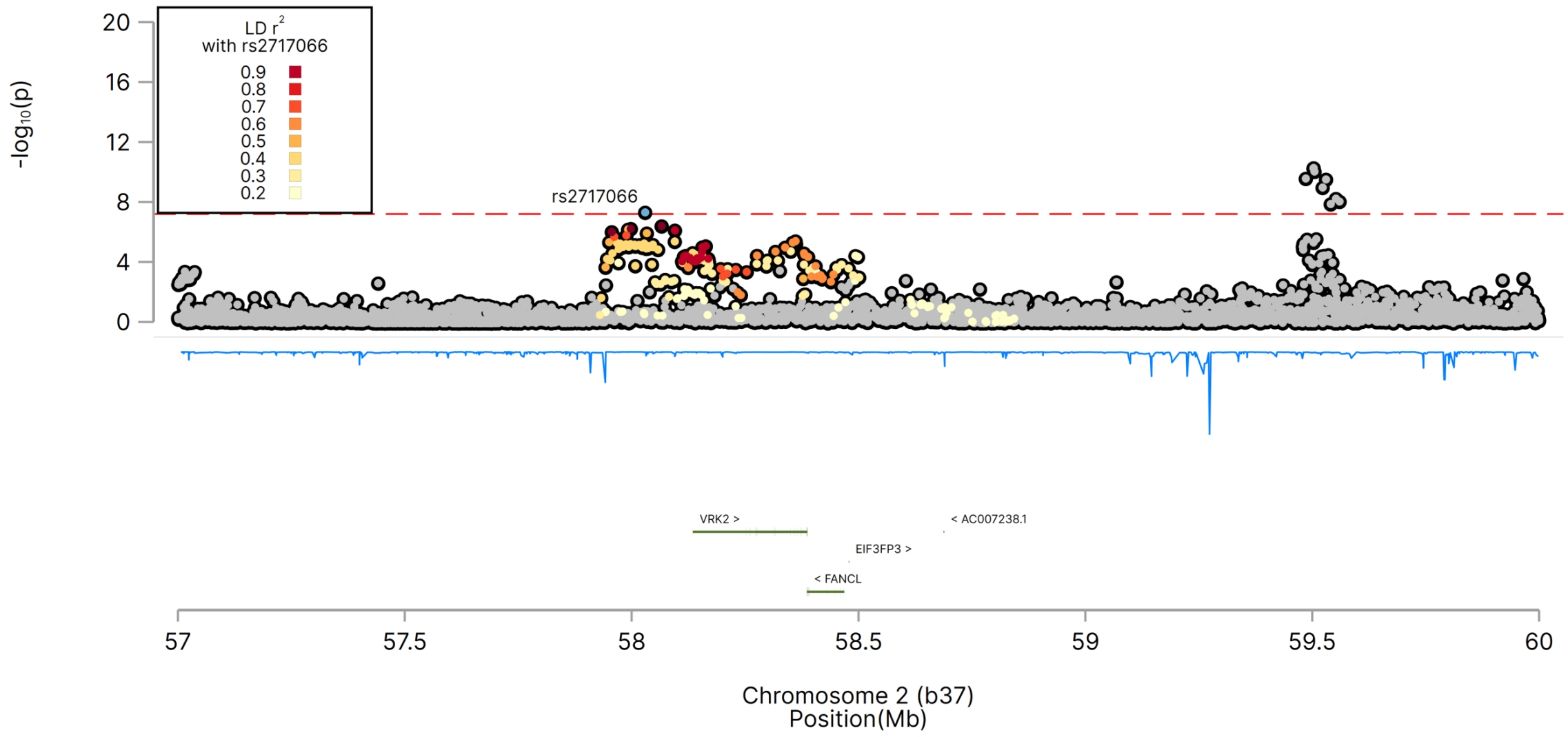

dataset: ukbb-fmri-rsn-VAN

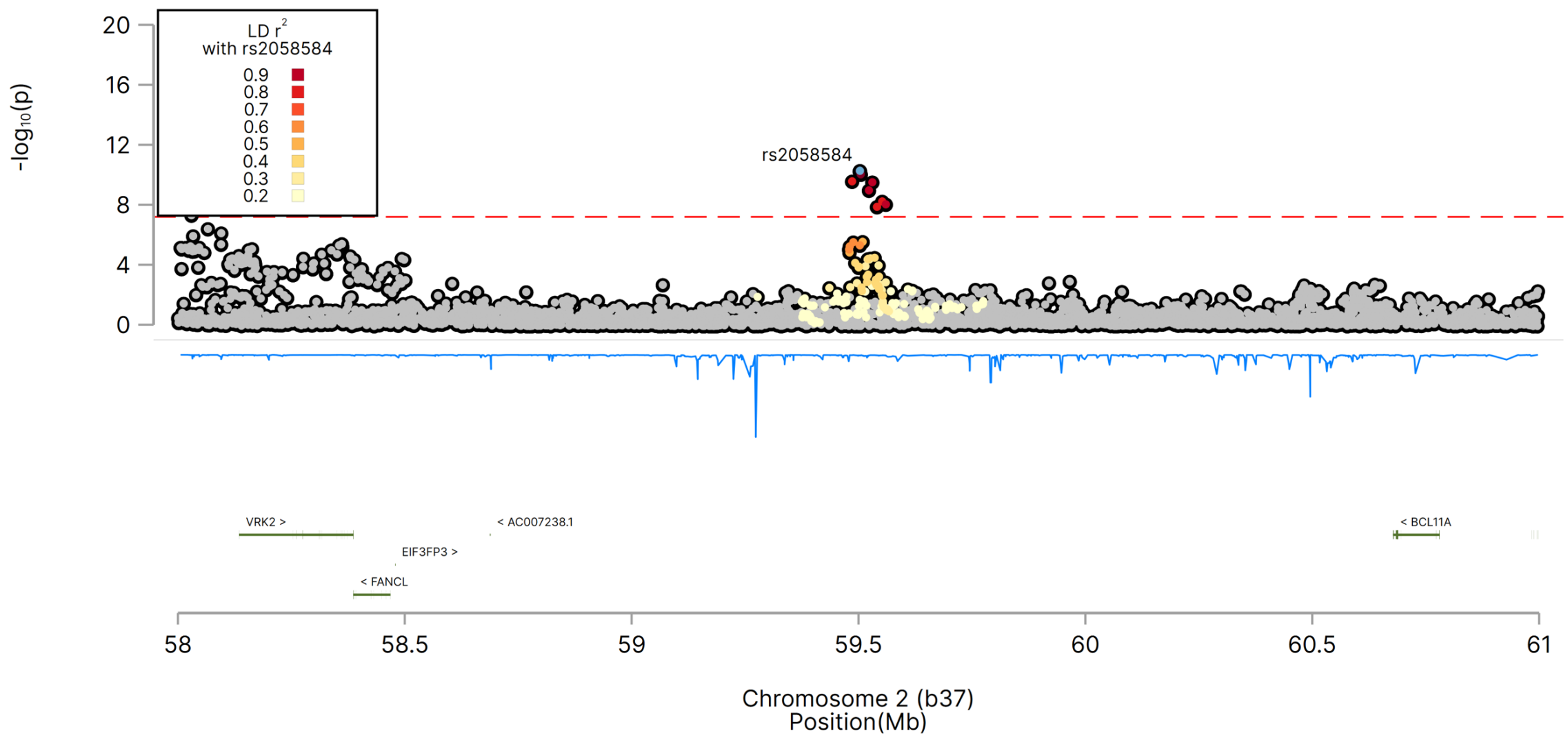

dataset: ukbb-fmri-rsn-VAN

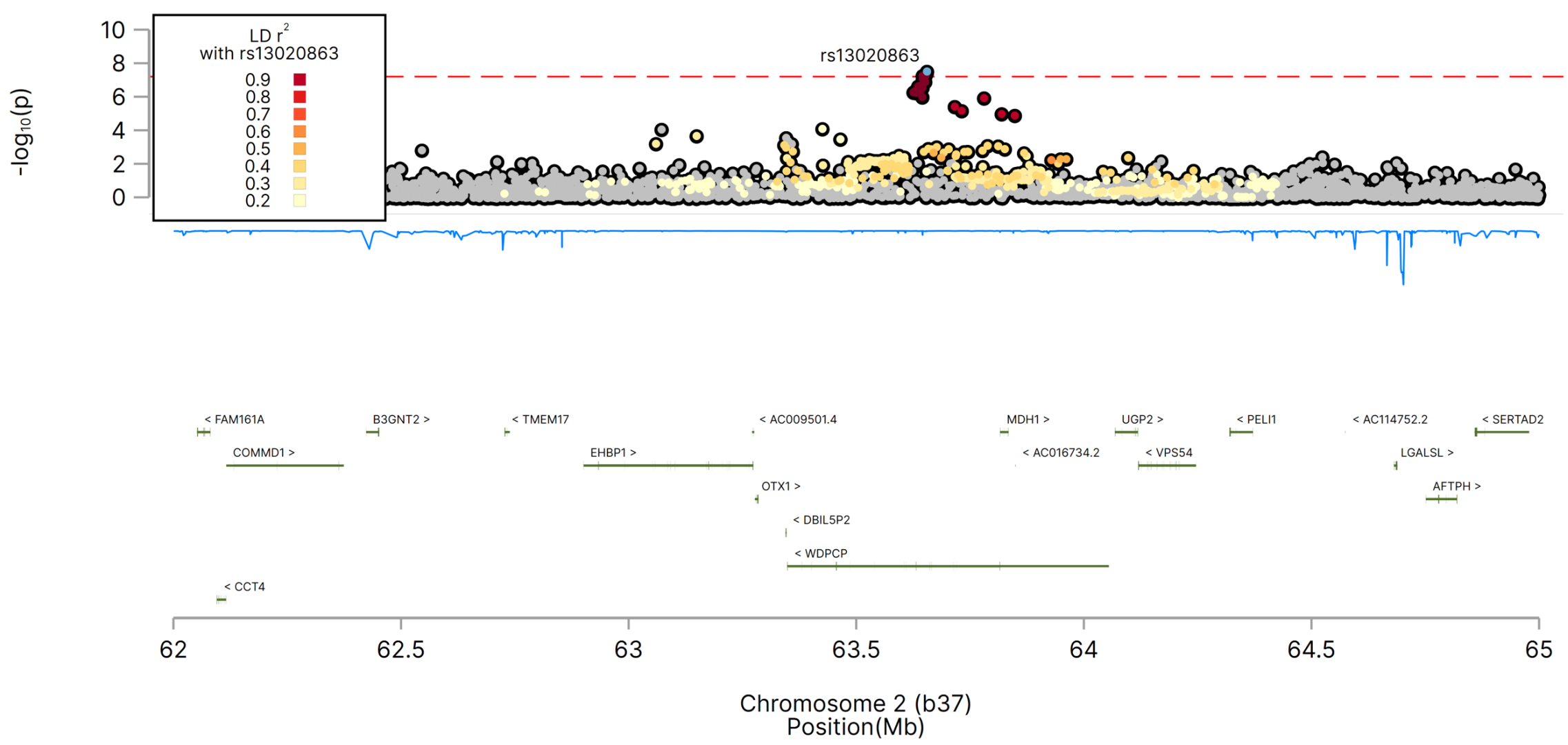

dataset: ukbb-fmri-rsn-VAN

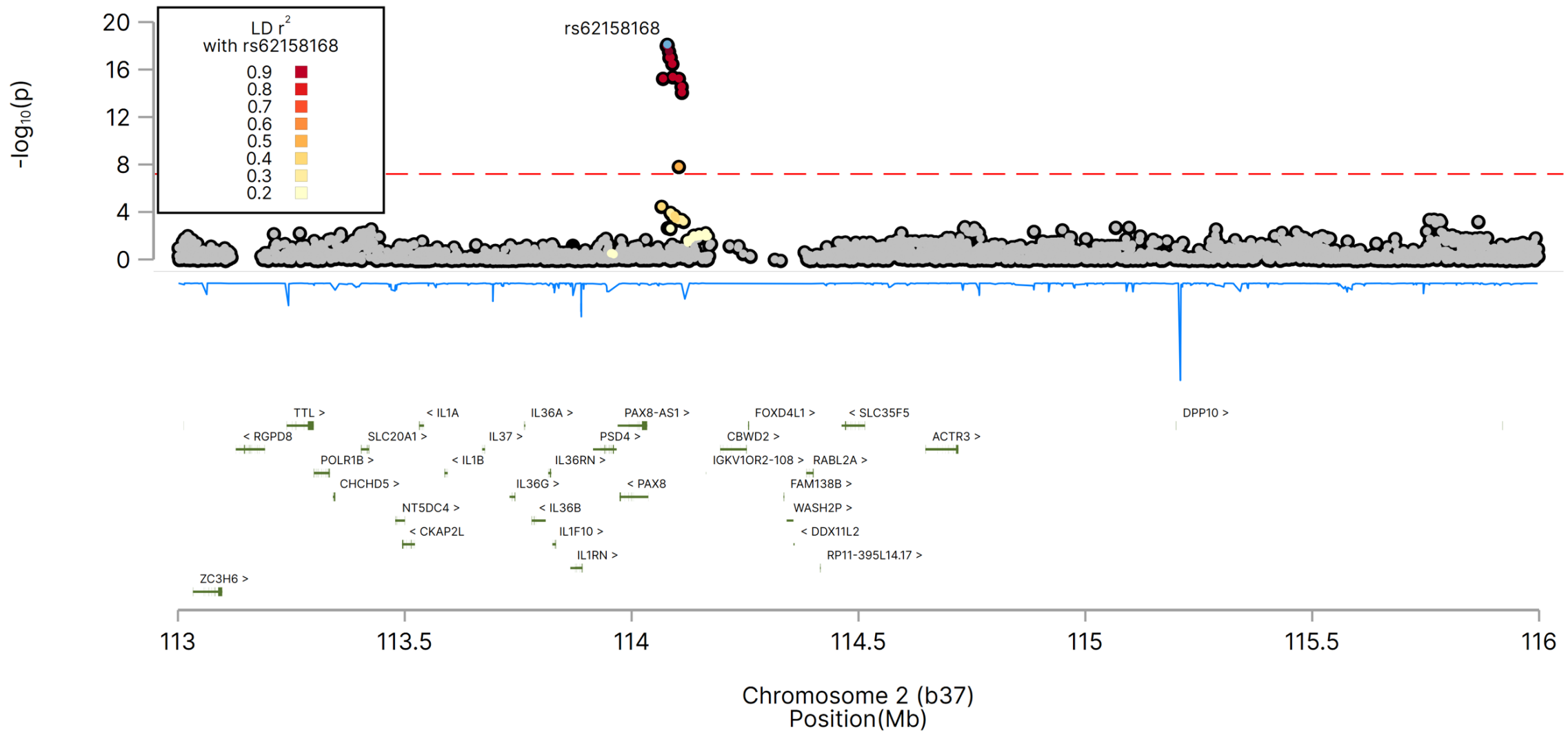

dataset: ukbb-fmri-rsn-VAN

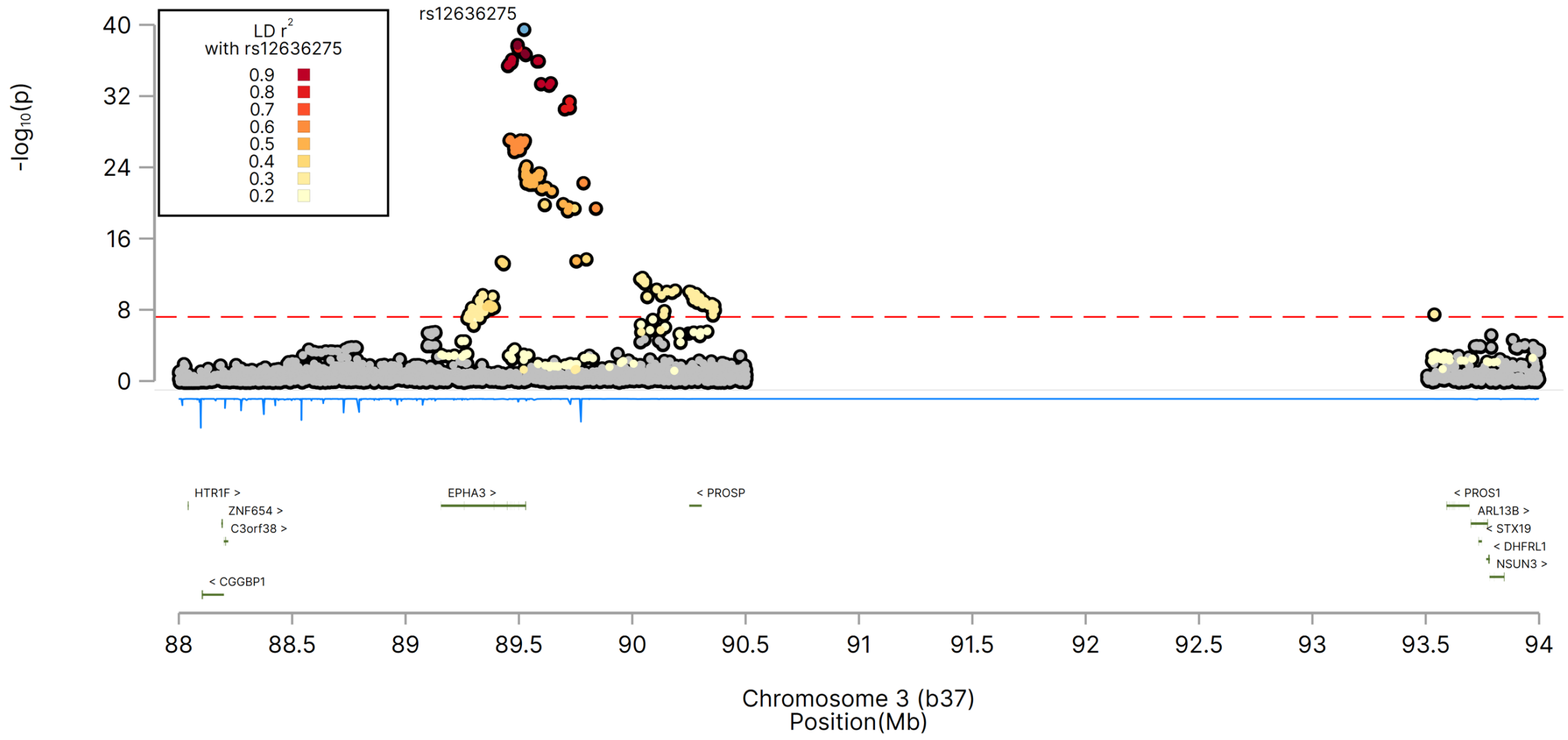

dataset: ukbb-fmri-rsn-VAN

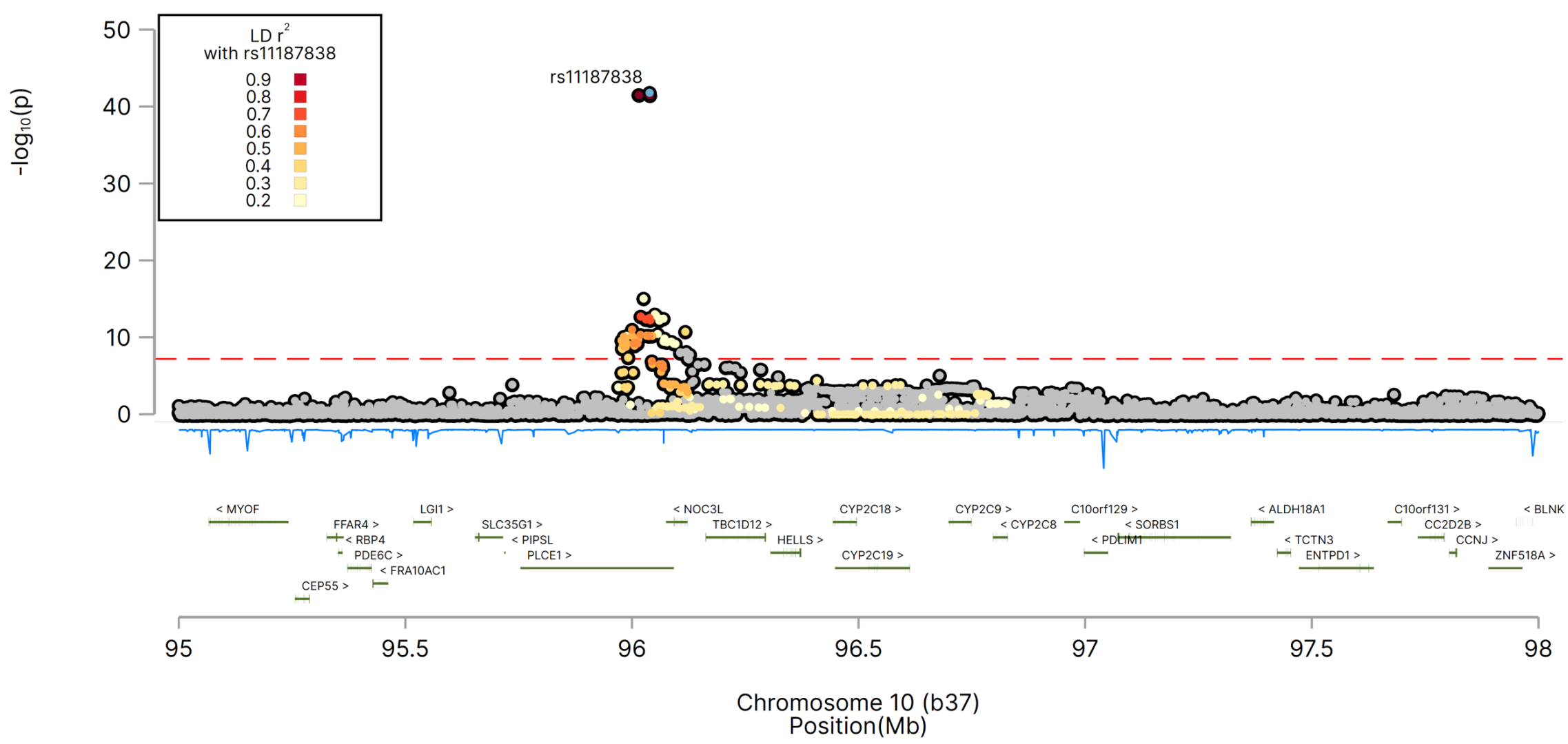

dataset: ukbb-fmri-rsn-VAN

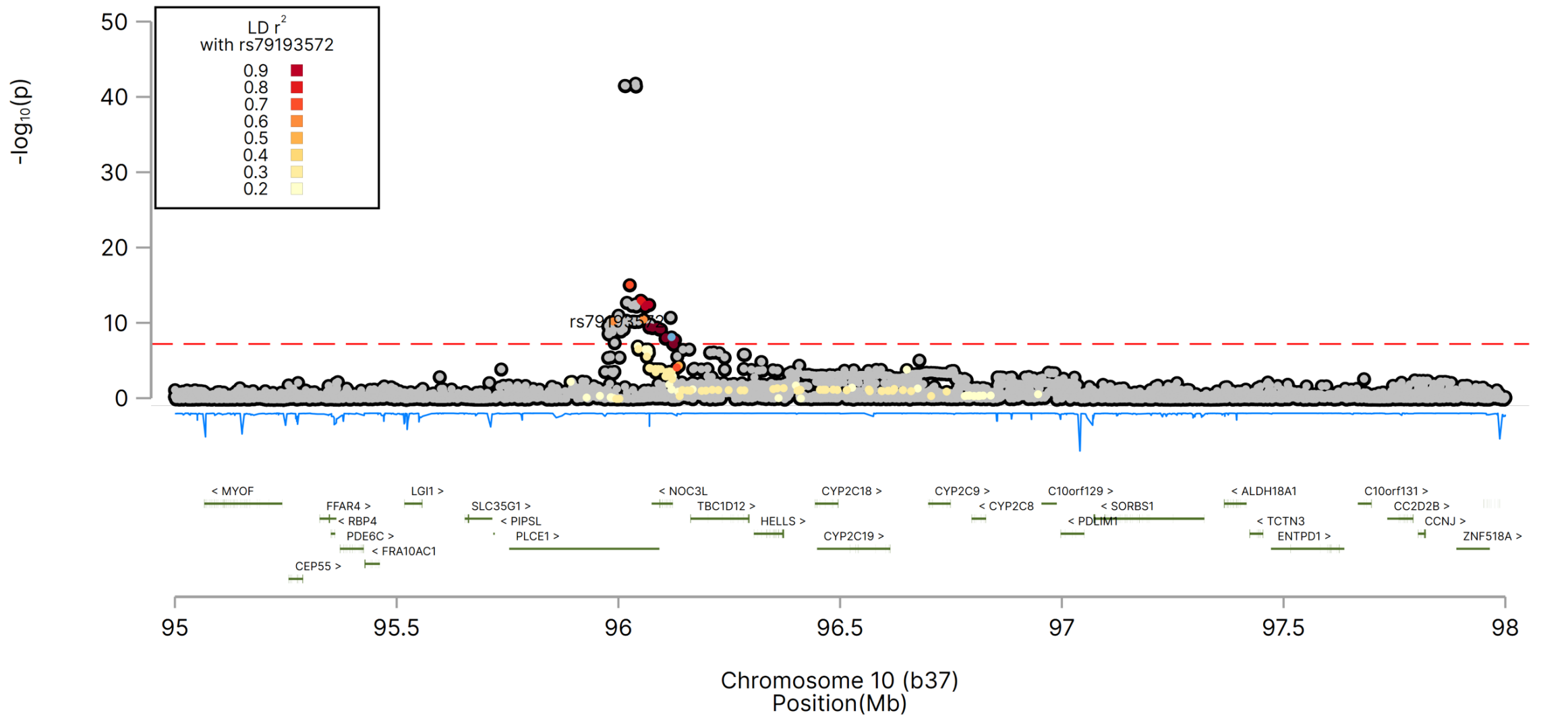

dataset: ukbb-fmri-rsn-VAN

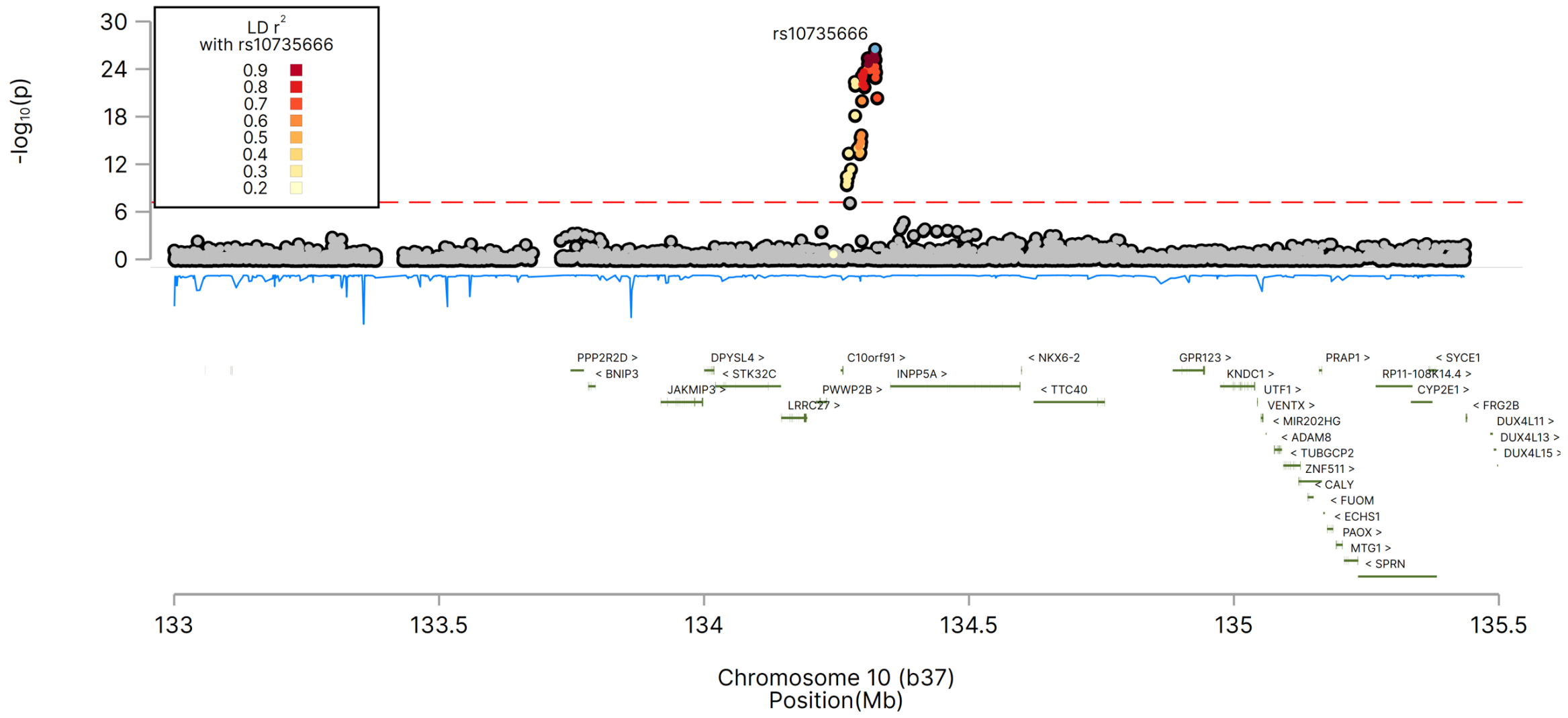

dataset: ukbb-fmri-rsn-VAN

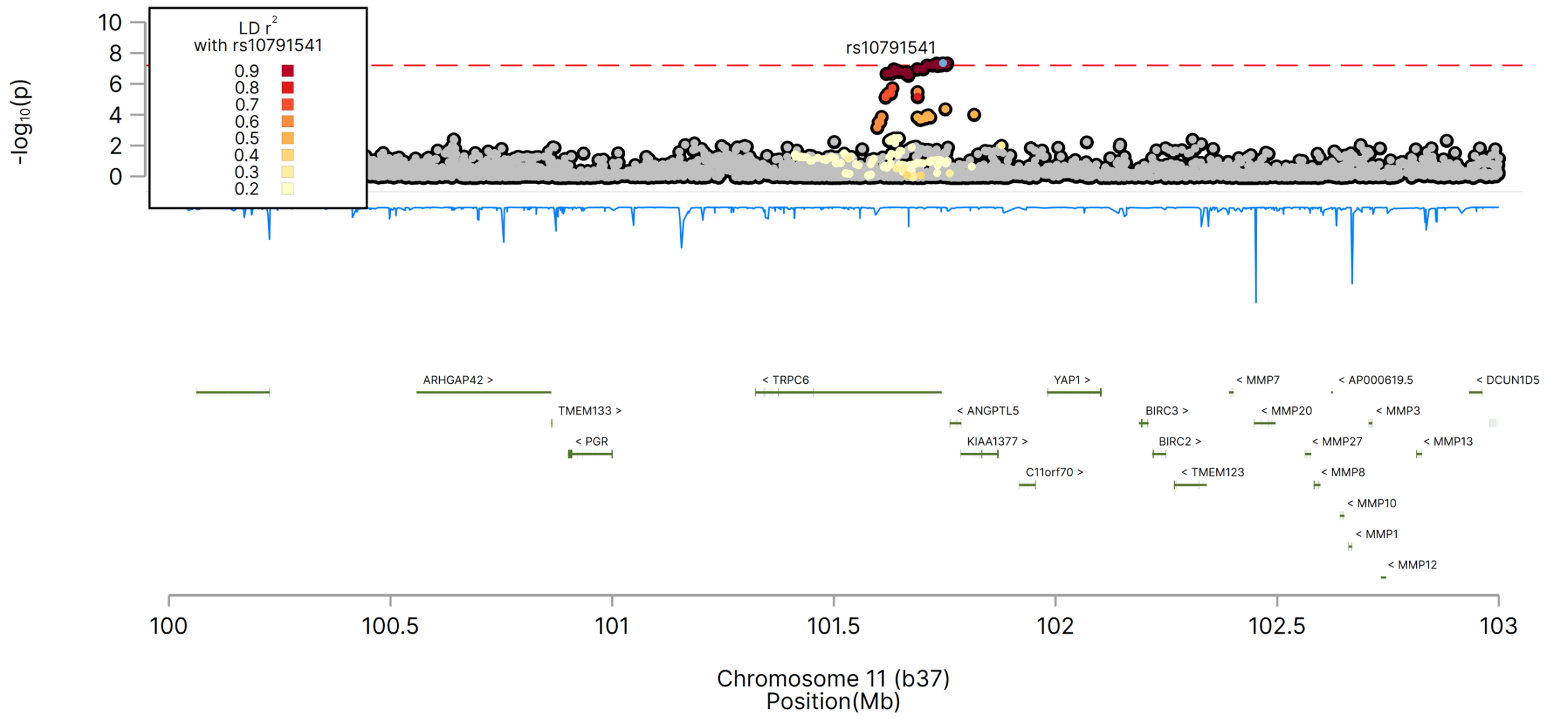

dataset: ukbb-fmri-rsn-VAN

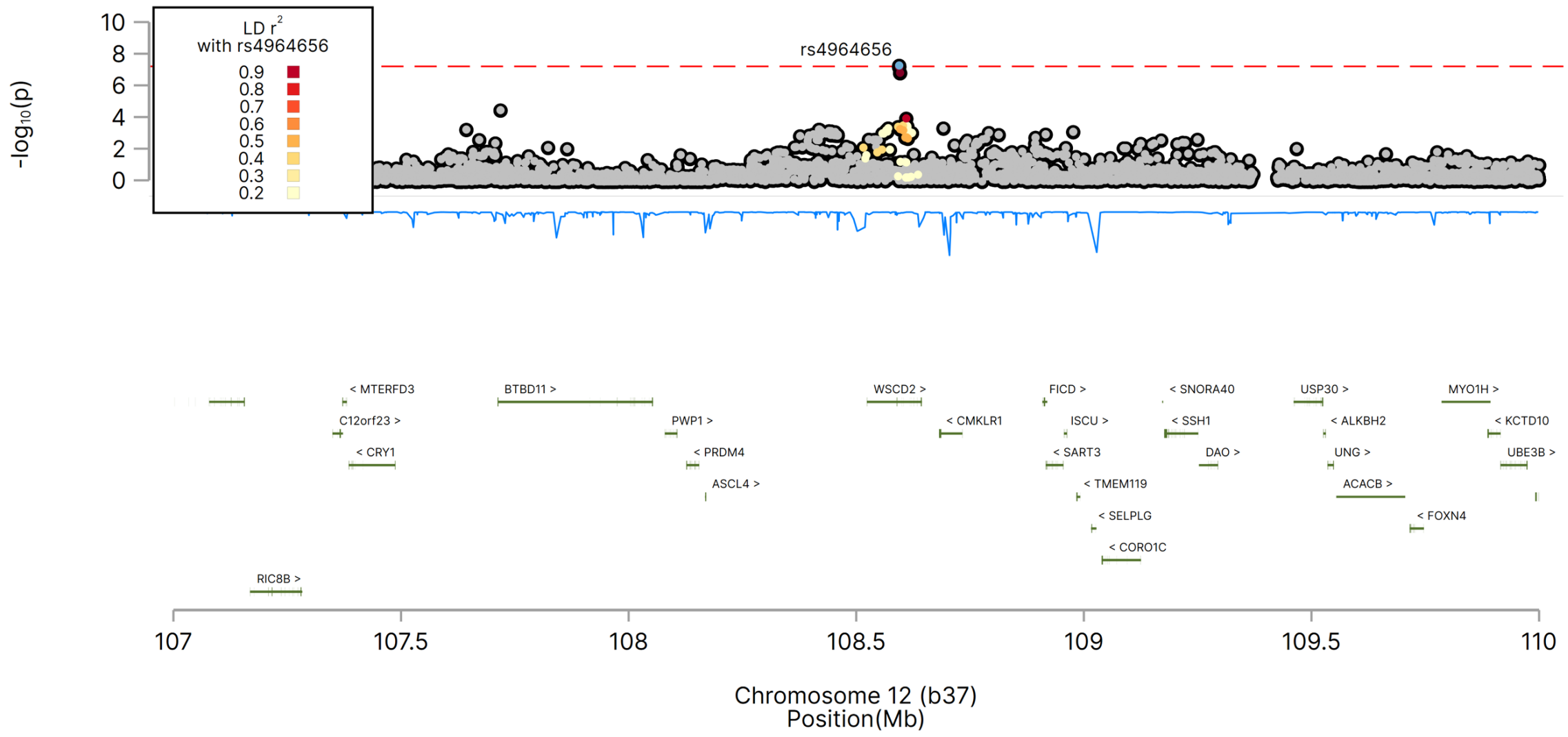

dataset: ukbb-fmri-rsn-VAN

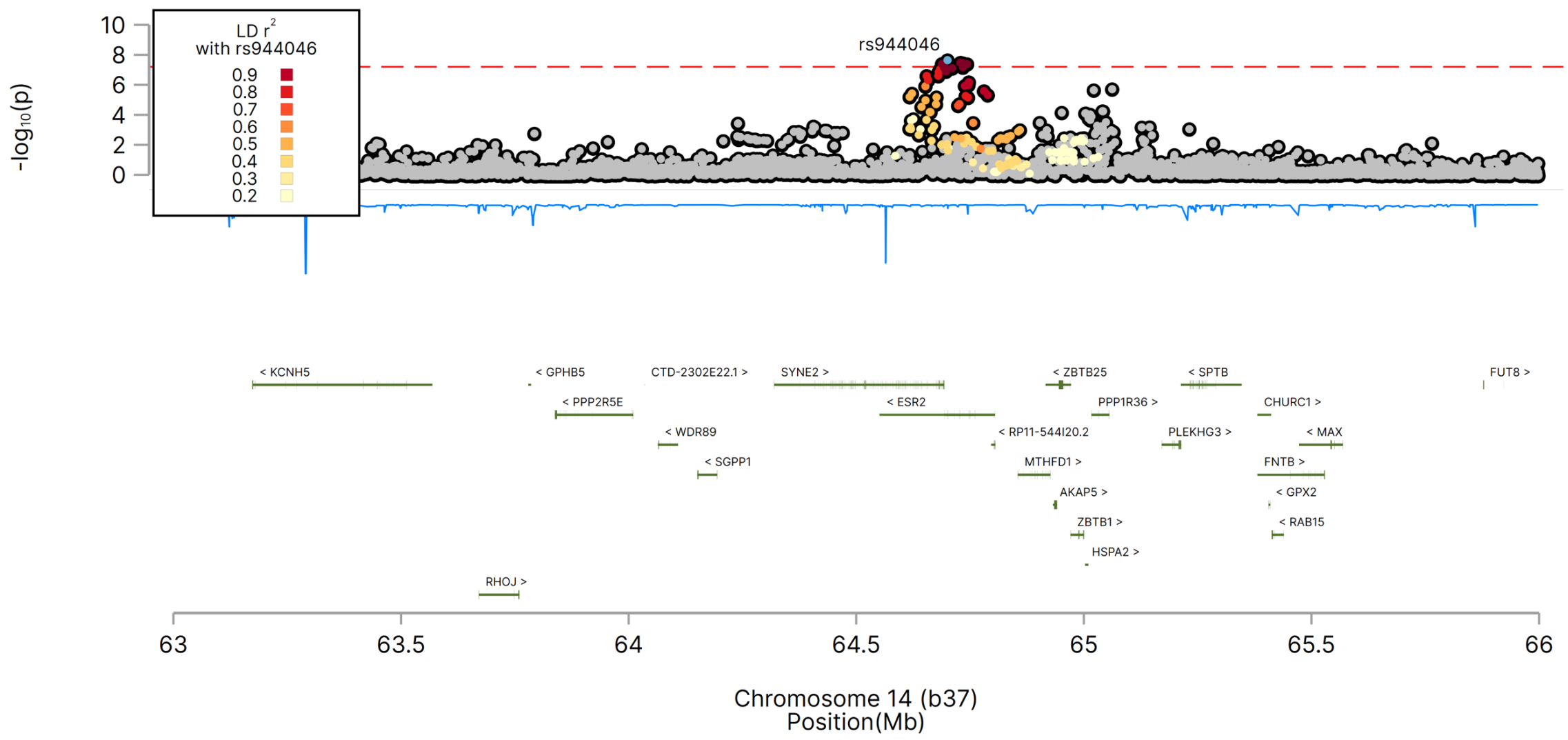

dataset: ukbb-fmri-rsn-VAN

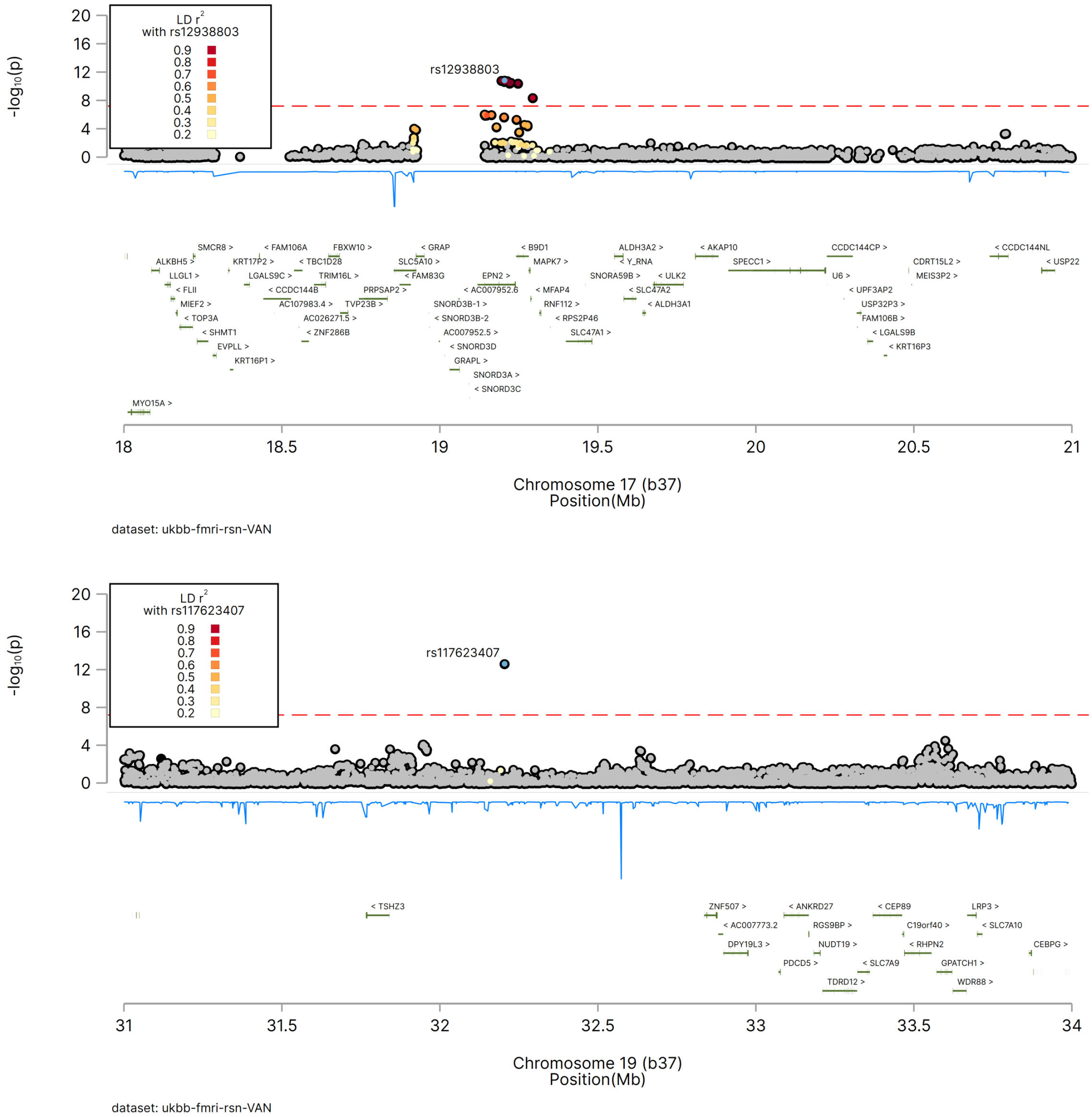

Supplementary Figure 8. Locus-zoom for all significant LD-independent signals coming from the MOST GWAS test for the Ventral-Attention Network (VAN). Source data for this figure can be obtained from https://www.ebi.ac.uk/gwas/; hyperlinks to the data for each network are provided in the Data Availability section.

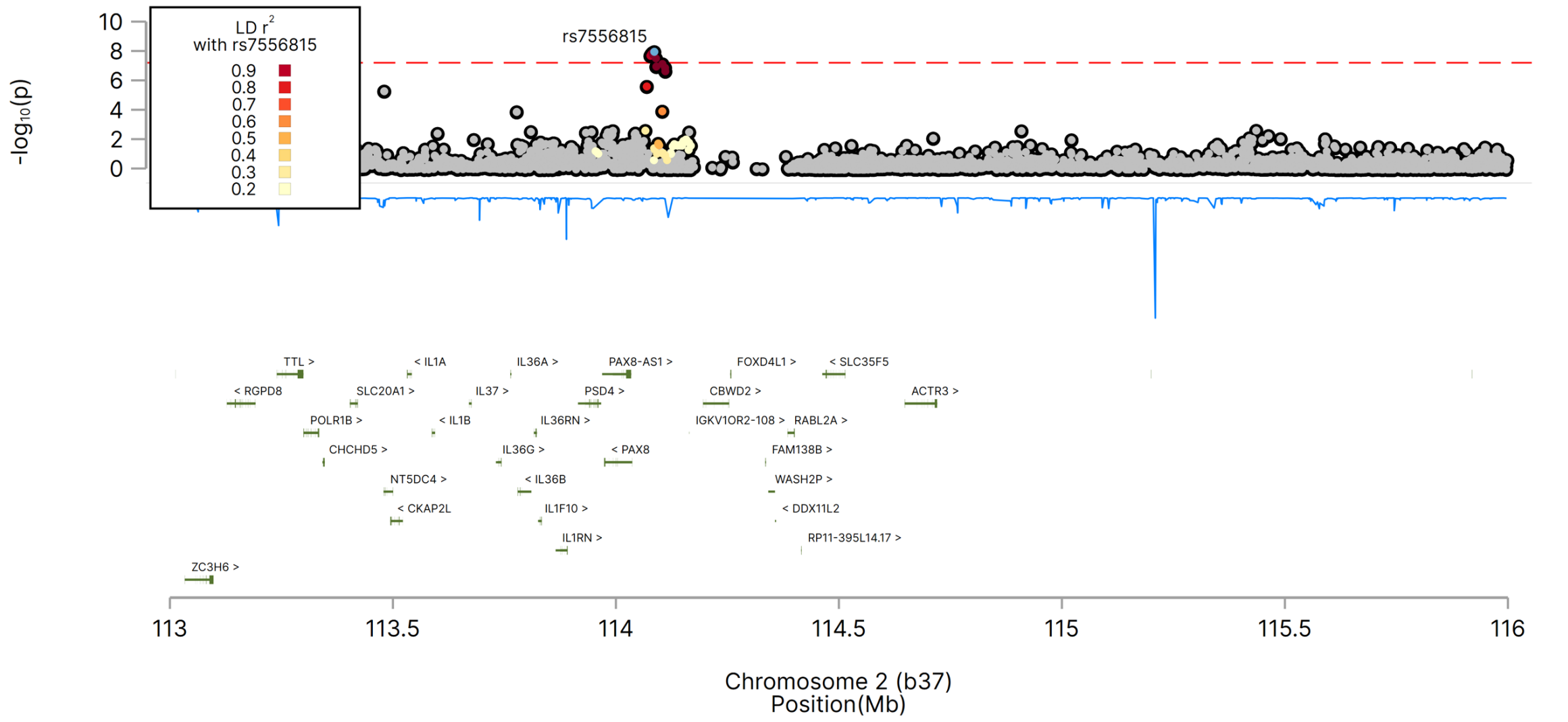

dataset: ukbb-fmri-rsn-VN

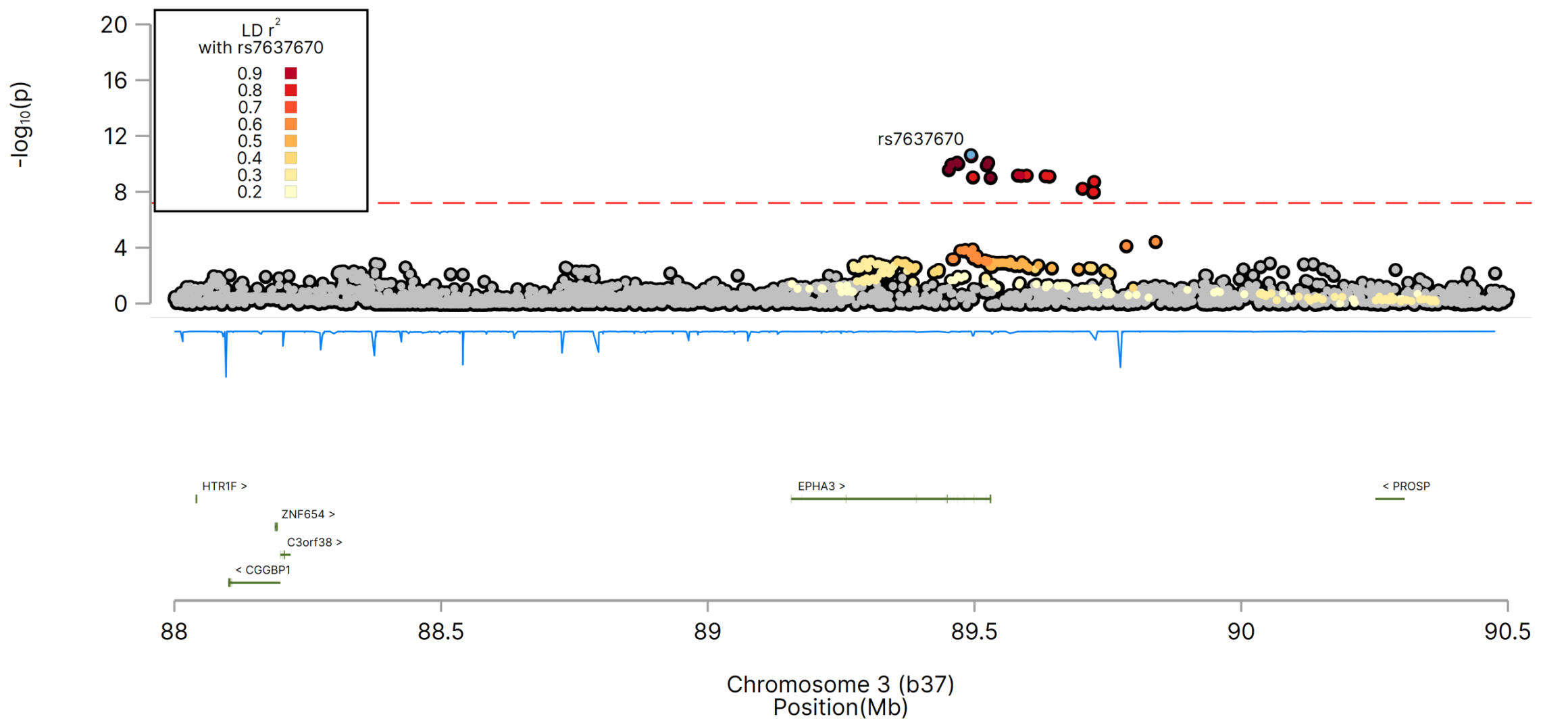

dataset: ukbb-fmri-rsn-VN

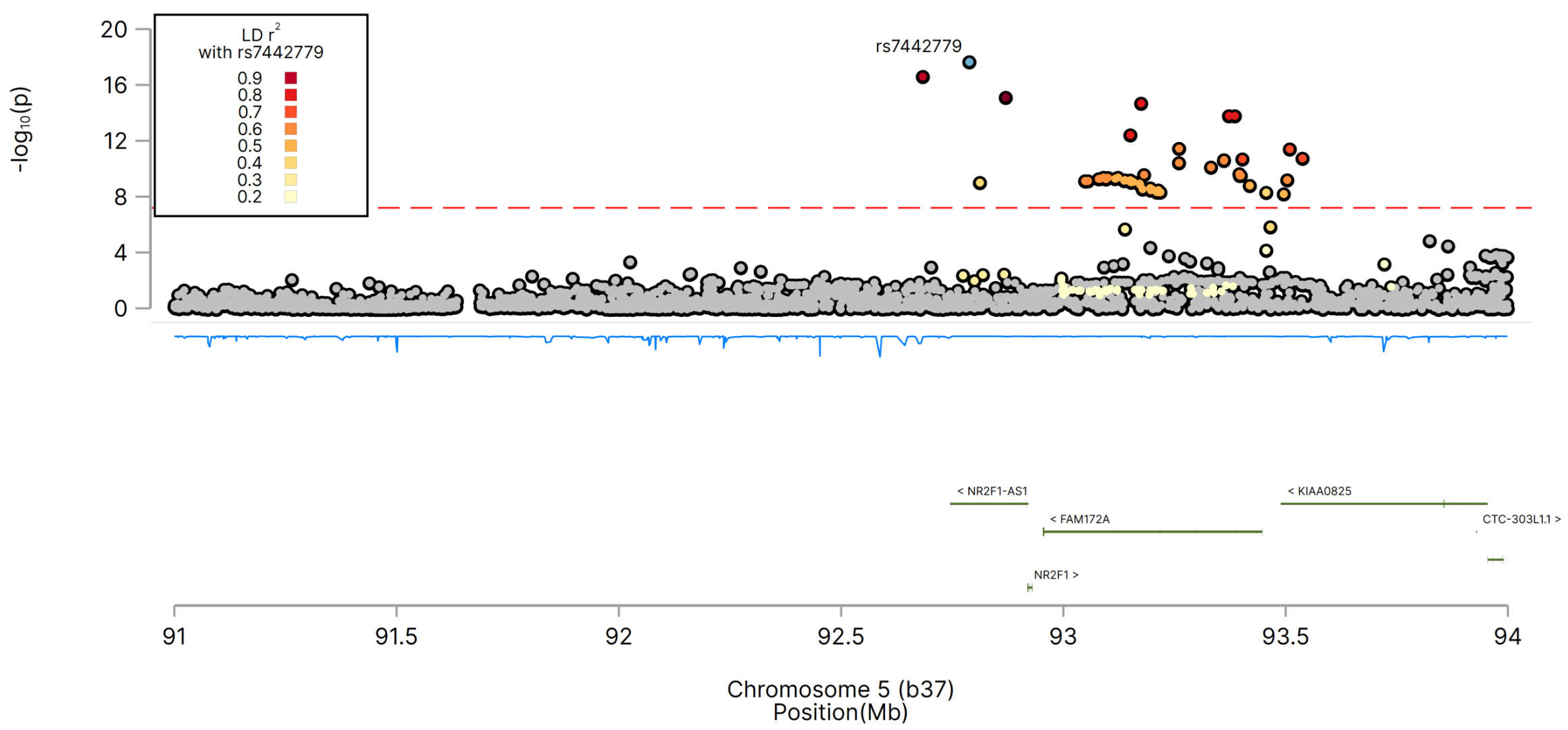

dataset: ukbb-fmri-rsn-VN

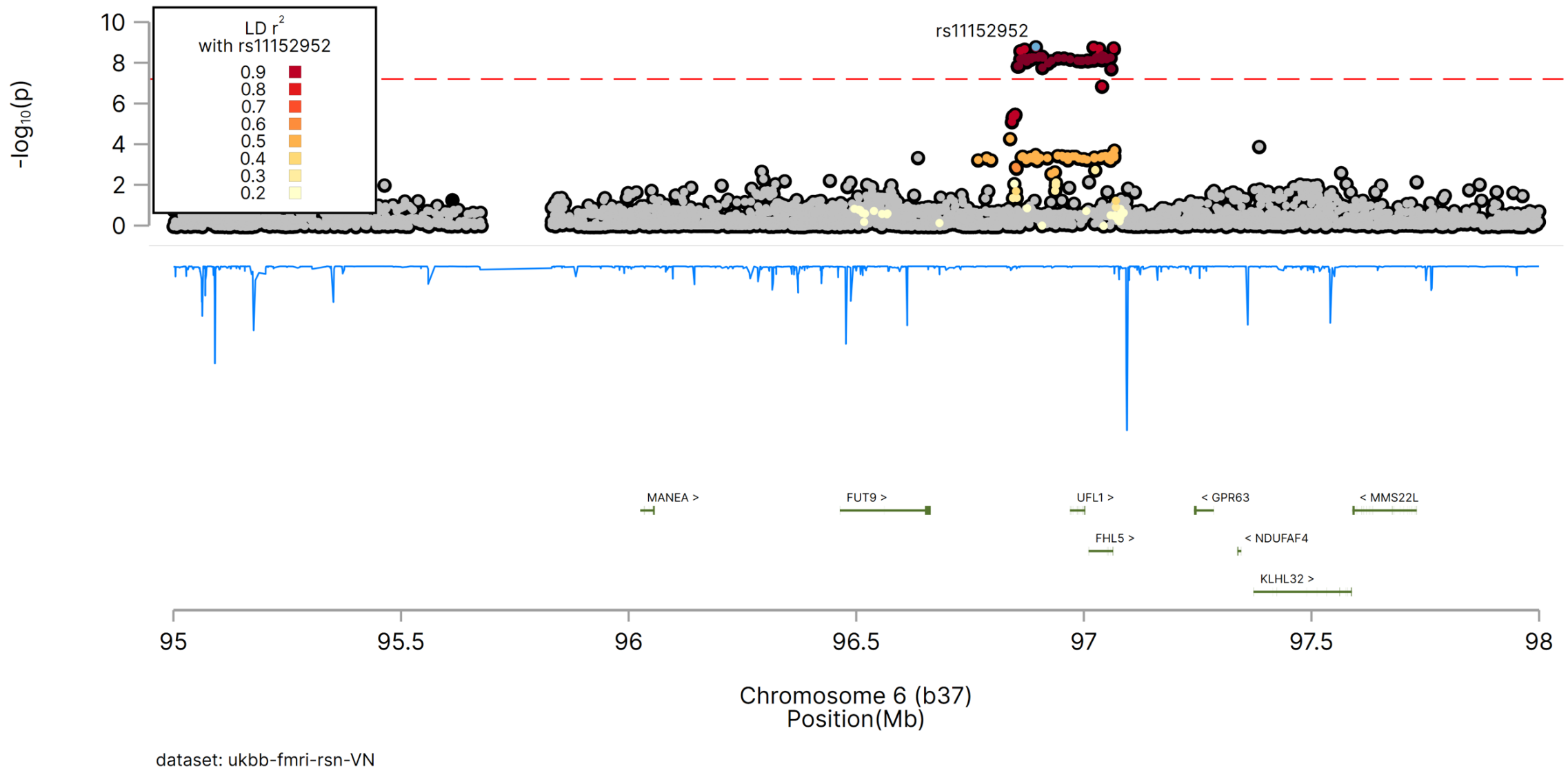

-log10(p)
LD r2
with rs11152952
0.9
0.8
0.7
0.6
0.5
0.4
0.3
0.2
rs11152952
MANEA >
FUT9 >
UFL1 >
< GPR63
< MMS22L
FHL5 >
< NDUFAF4
KLHL32 >
95
95.5
96
96.5
97
97.5
98
Chromosome 6 (b37)
Position(Mb)
dataset: ukbb-fmri-rsn-VN

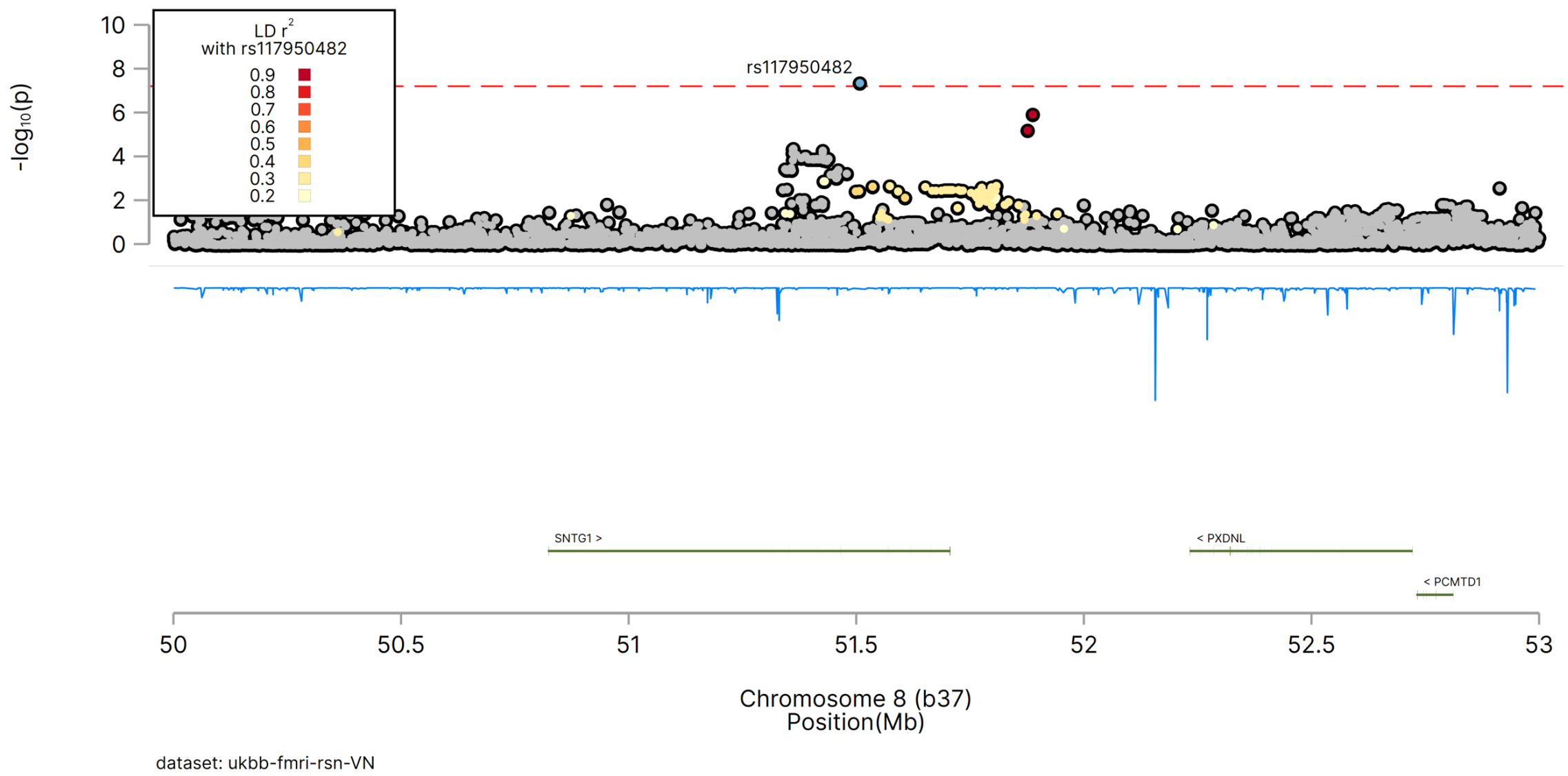

-log10(p)
LD r2
with rs117950482
0.9
0.8
0.7
0.6
0.5
0.4
0.3
0.2
rs117950482
SNTG1 >
< PXDNL
< PCMTD1
50
50.5
51
51.5
52
52.5
53
Chromosome 8 (b37)
Position(Mb)
dataset: ukbb-fmri-rsn-VN

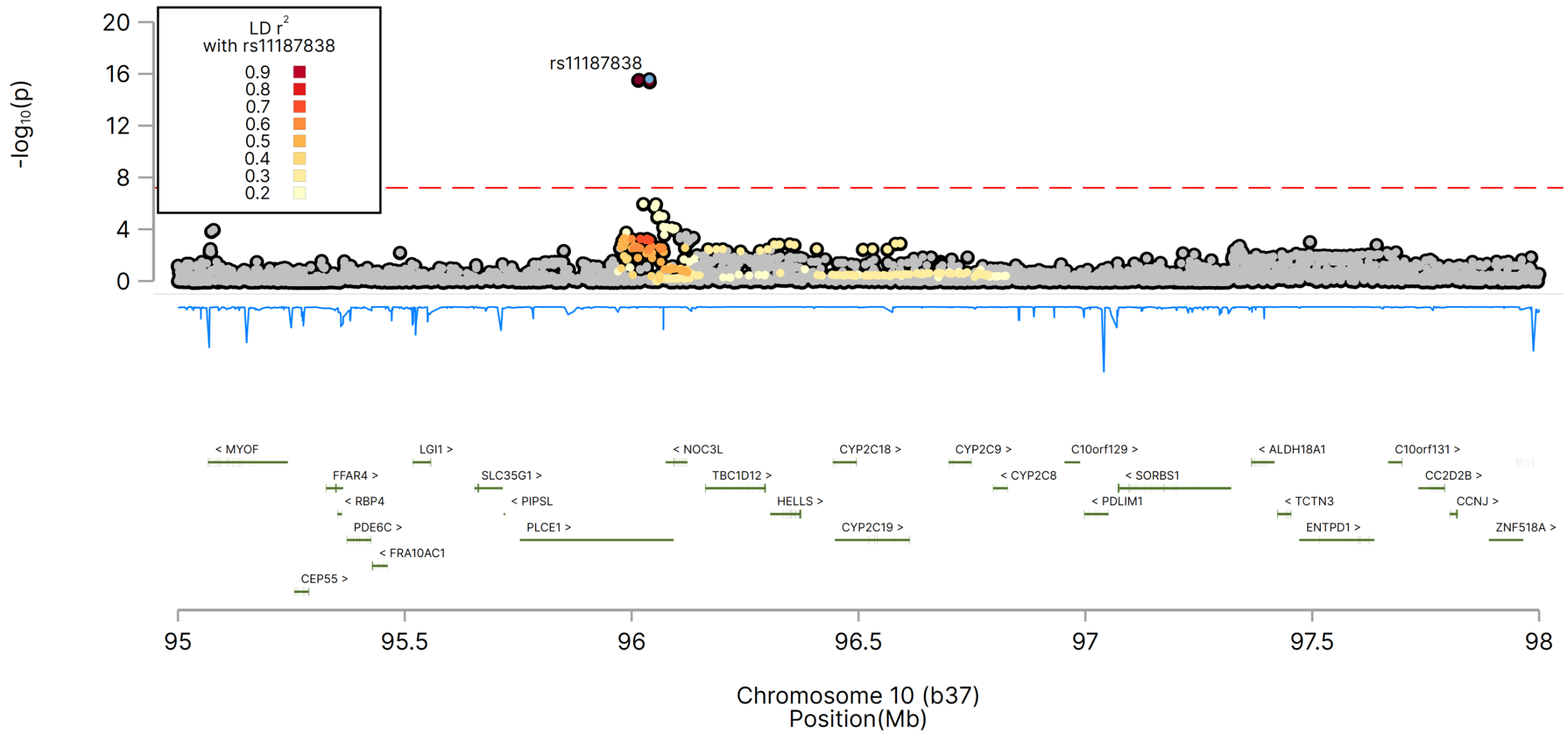

dataset: ukbb-fmri-rsn-VN

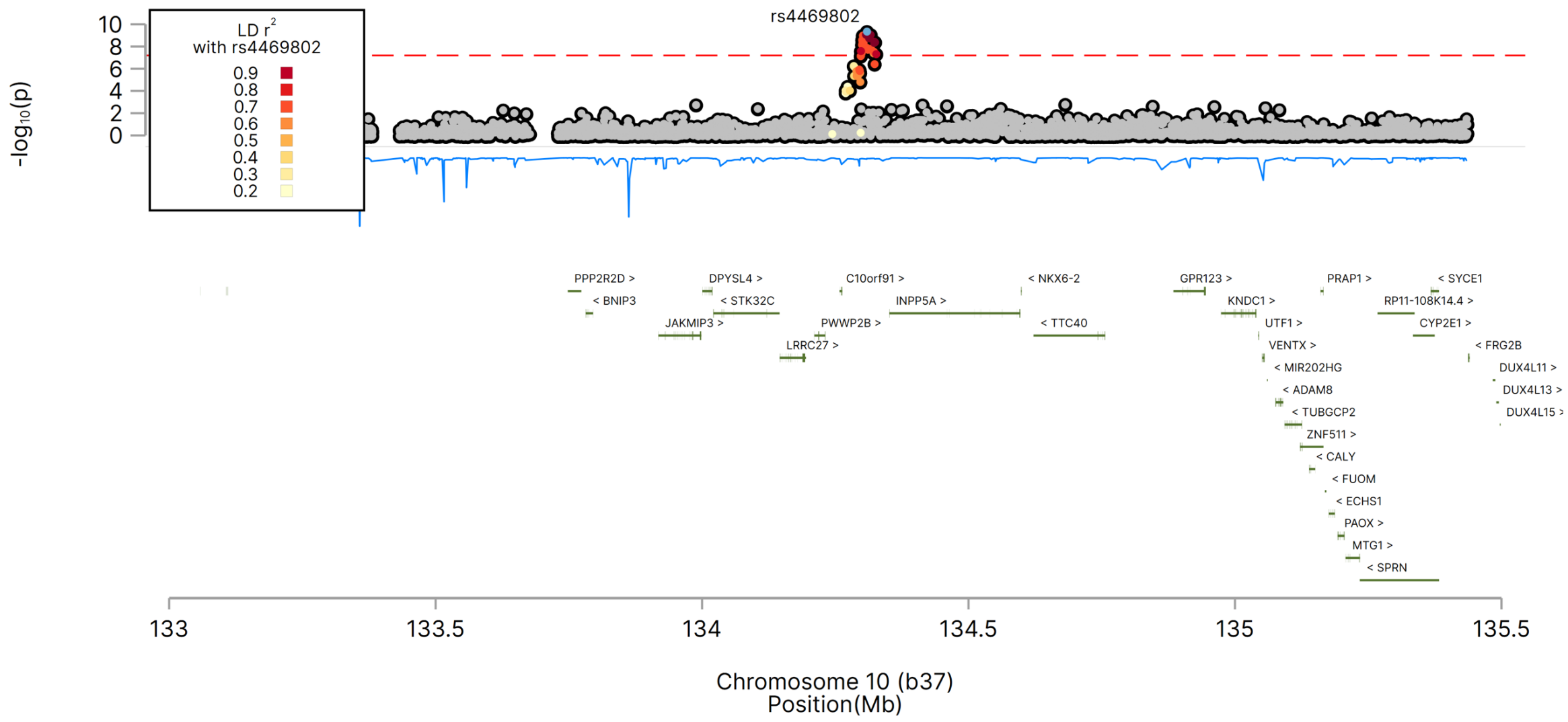

dataset: ukbb-fmri-rsn-VN

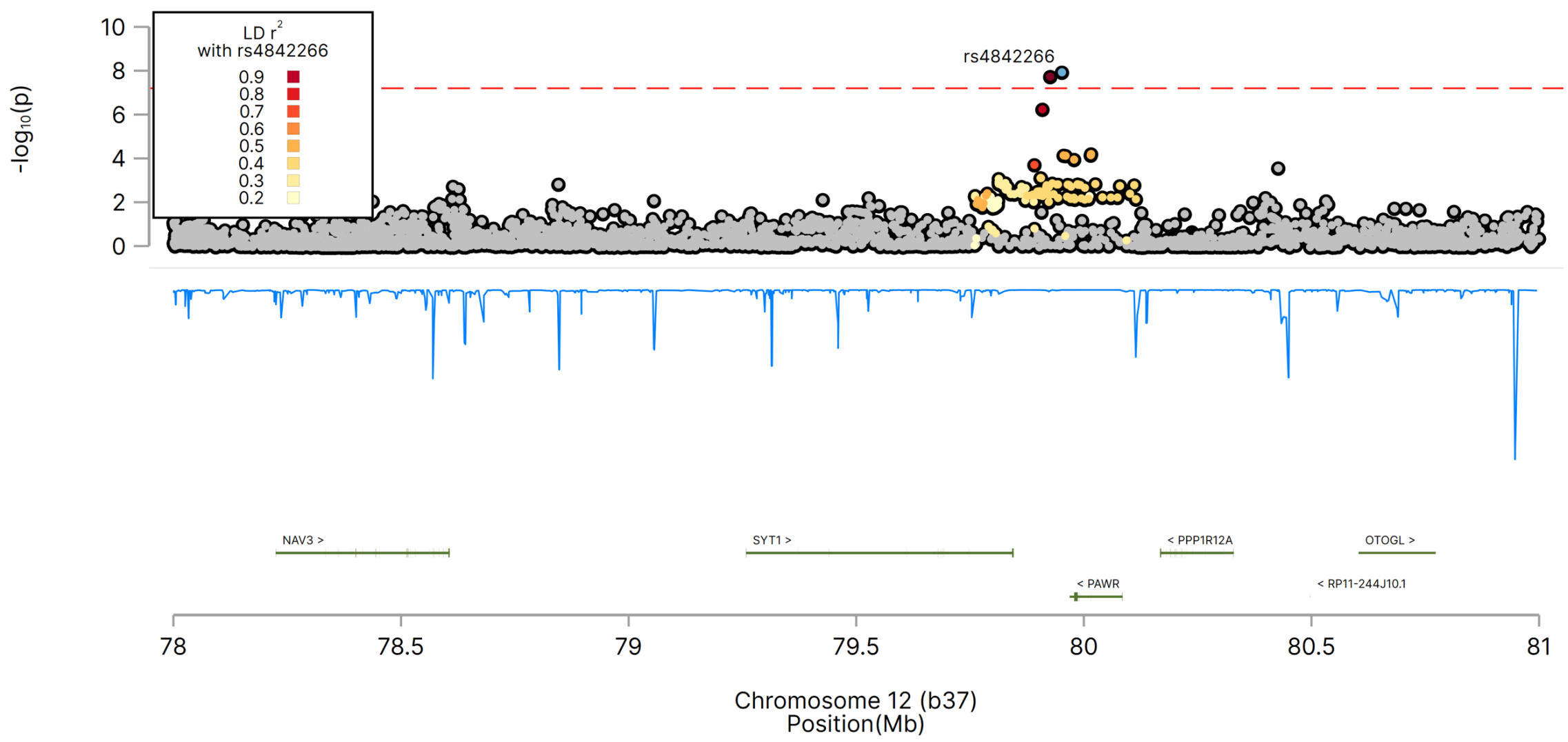

dataset: ukbb-fmri-rsn-VN

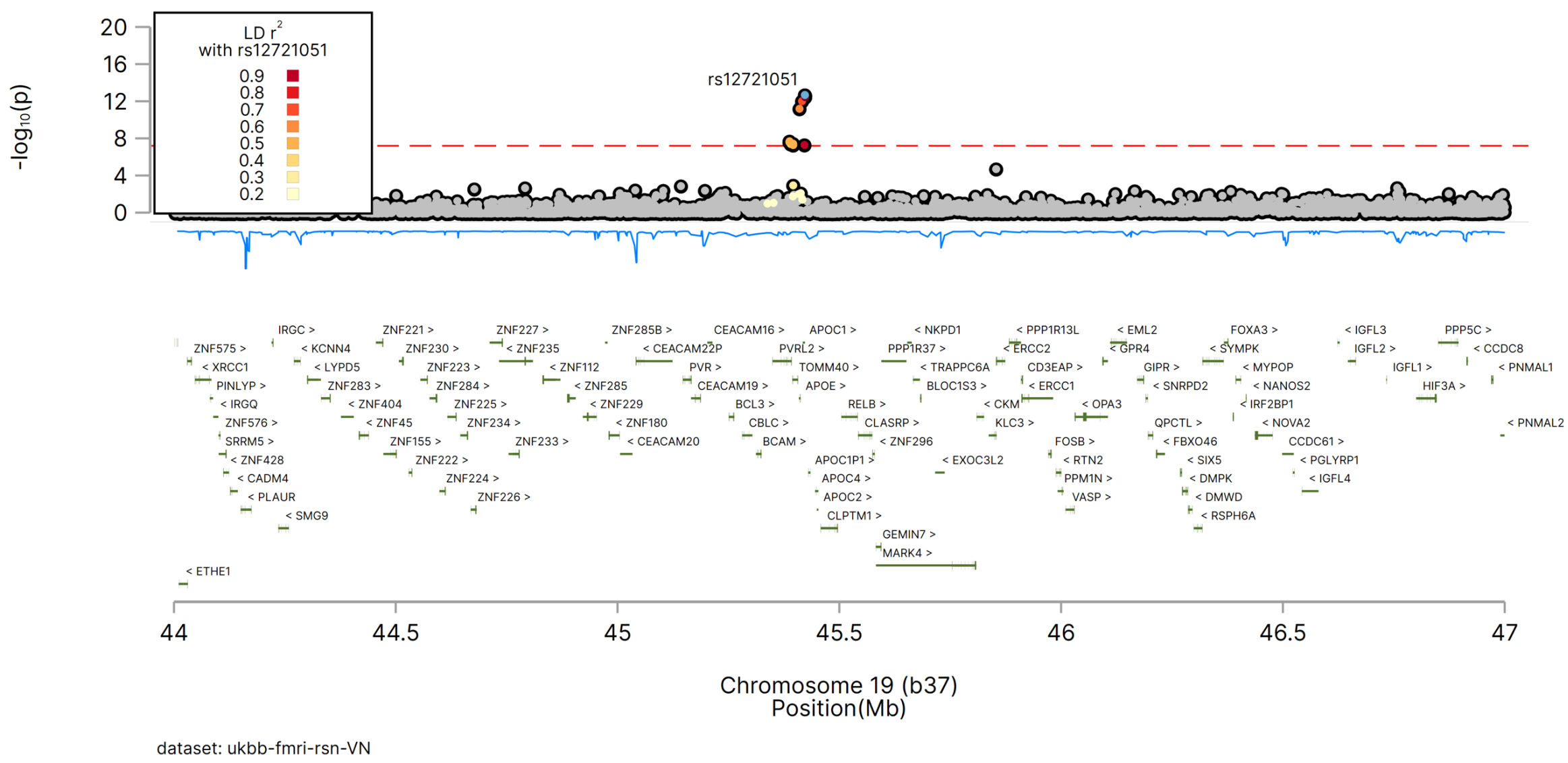

Supplementary Figure 9. Locus-zoom for all significant LD-independent signals coming from the MOST GWAS test for the Visual Network. Source data for this figure can be obtained from https://www.ebi.ac.uk/gwas/; hyperlinks to the data for each network are provided in the Data Availability section.

Chromosome 1, position: 71684405 – 74326907

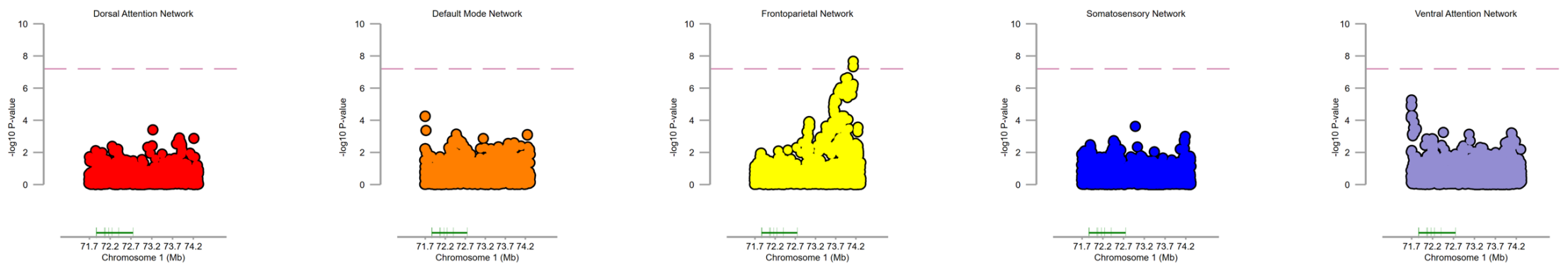

Chromosome 1, position: 197311514 – 199239884

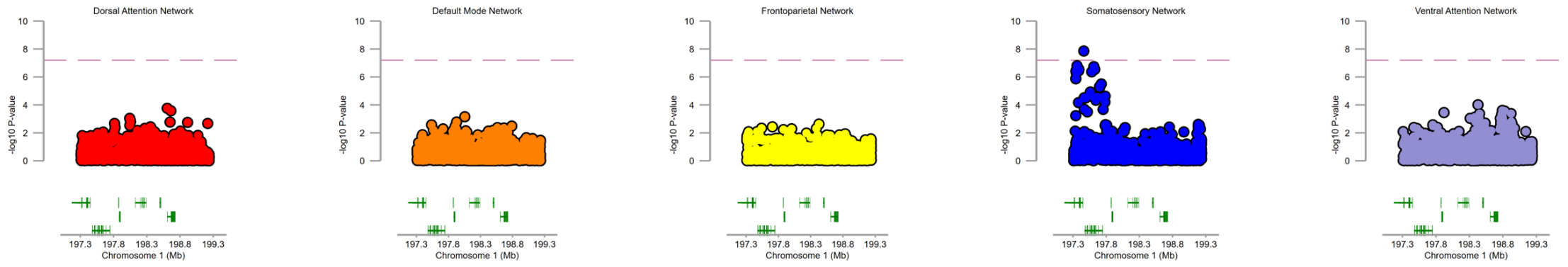

Chromosome 2, position: 40281483 - 43309590

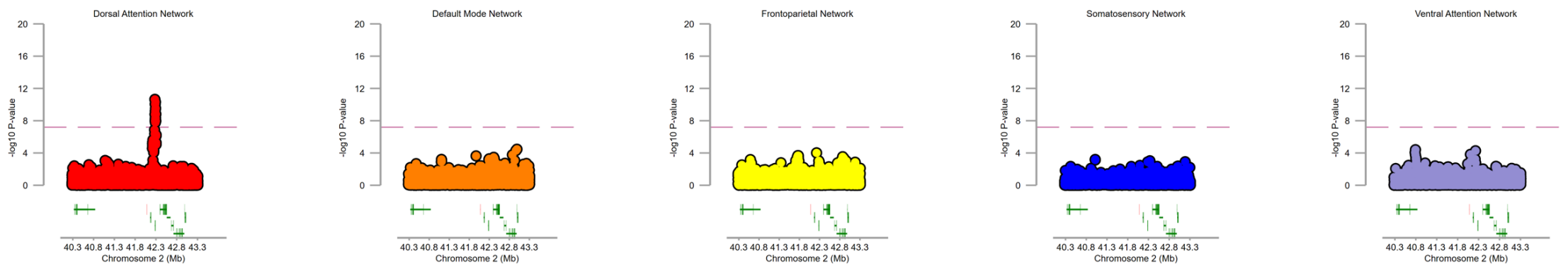

Chromosome 2, position: 48213001 - 50022257

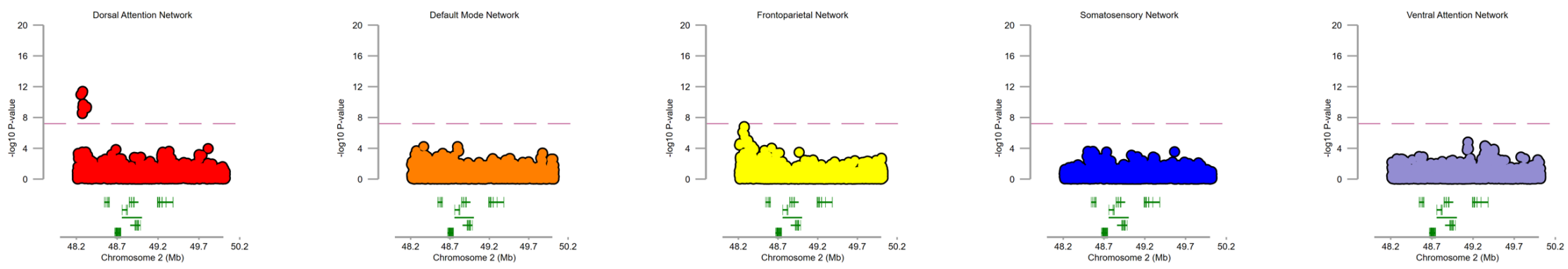

Chromosome 2, position: 57429100 - 58297315

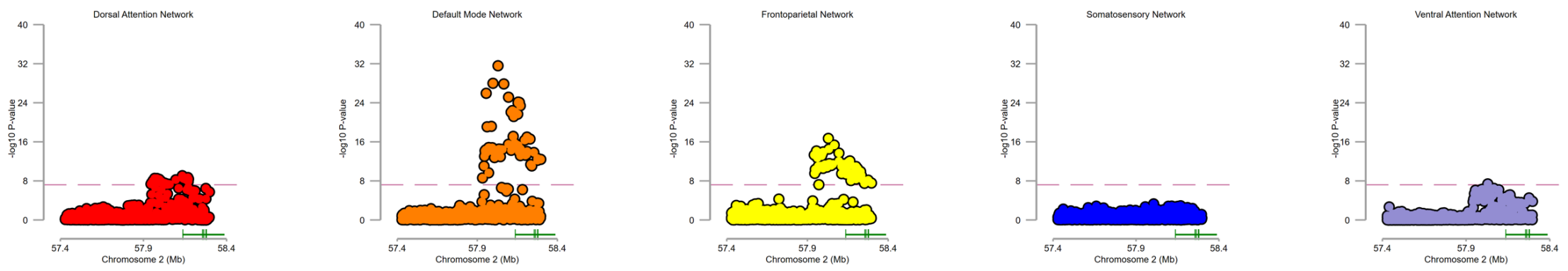

Chromosome 2, position: 58297315 - 60292000

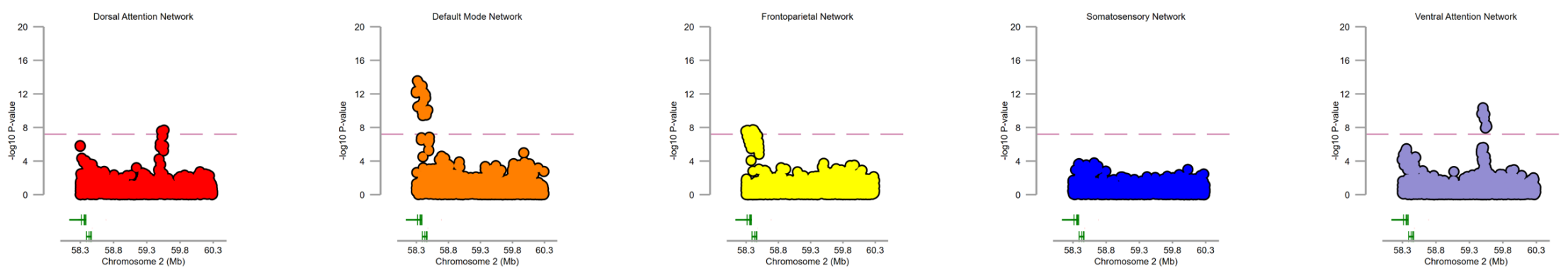

Chromosome 2, position: 62429044 - 64625913

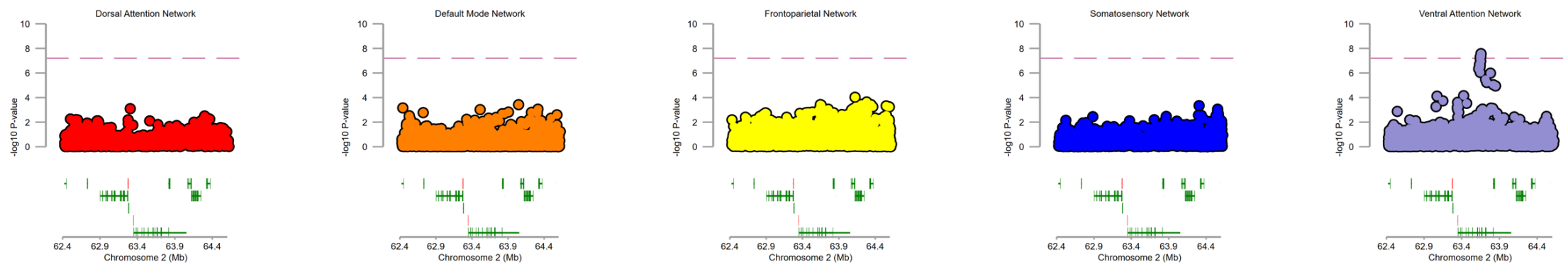

Chromosome 2, position: 113921856 - 116772470

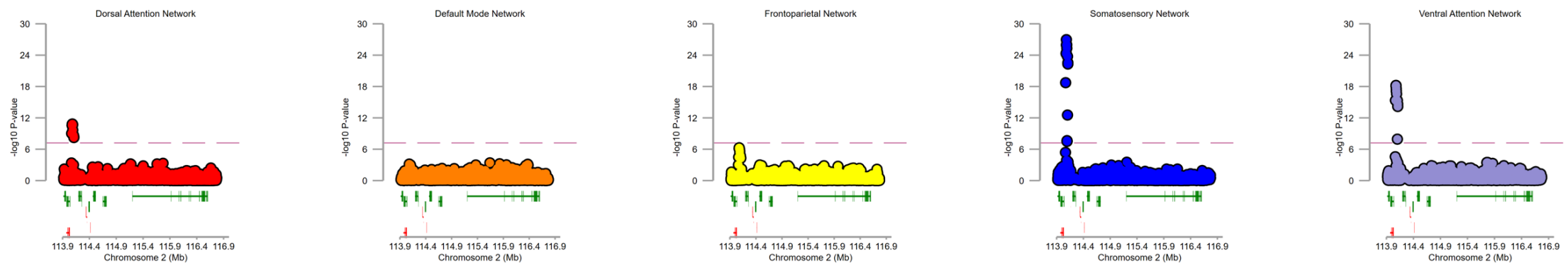

Chromosome 2, position: 161769733 - 163503551

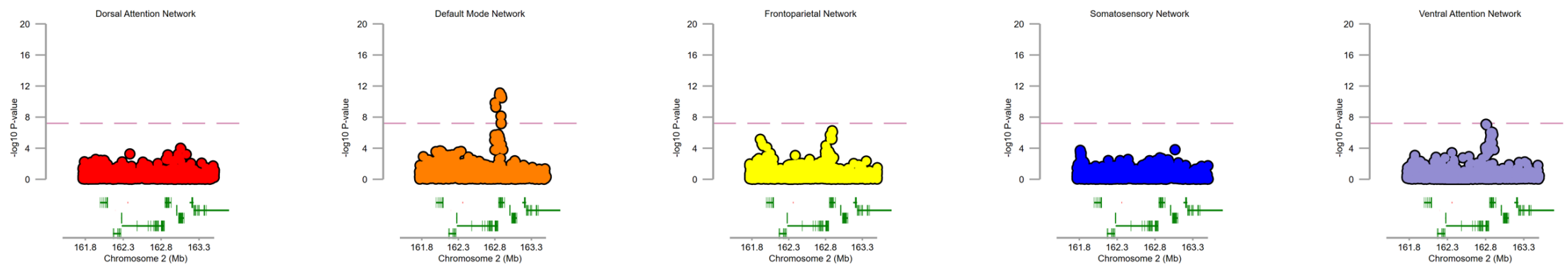

Chromosome 3, position: 16282442 - 17891118

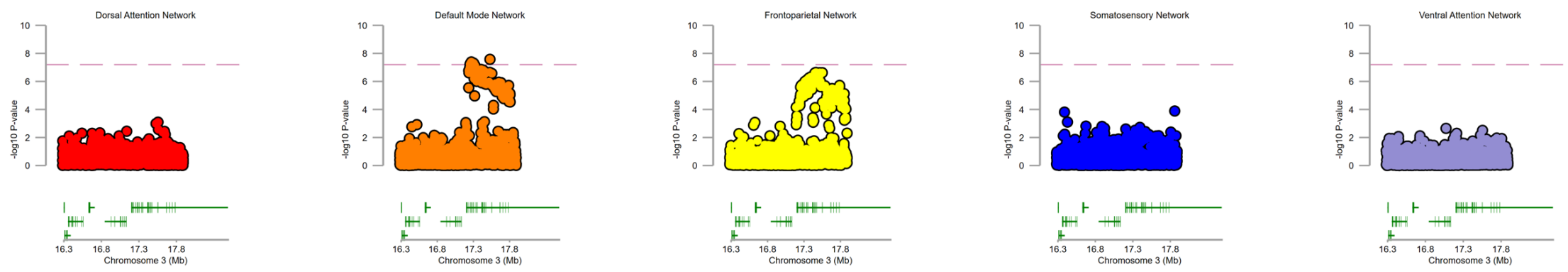

Chromosome 3, position: 84367479 - 85582231

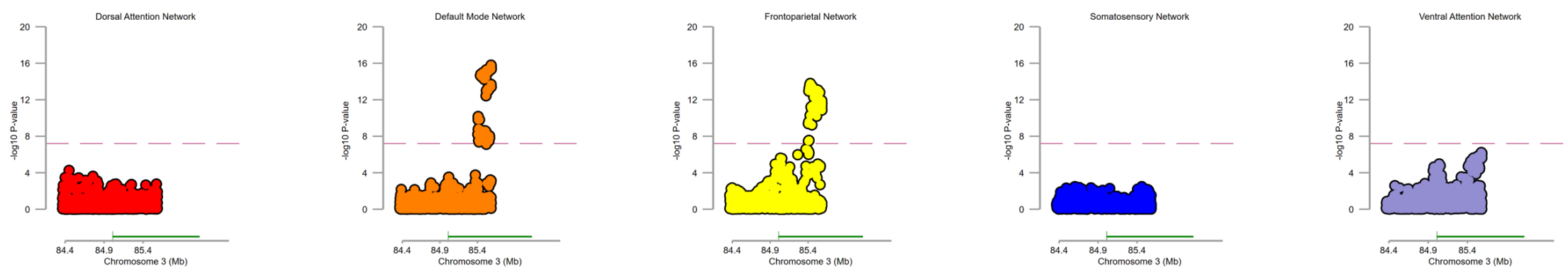

Chromosome 3, position: 85582231 - 87409732

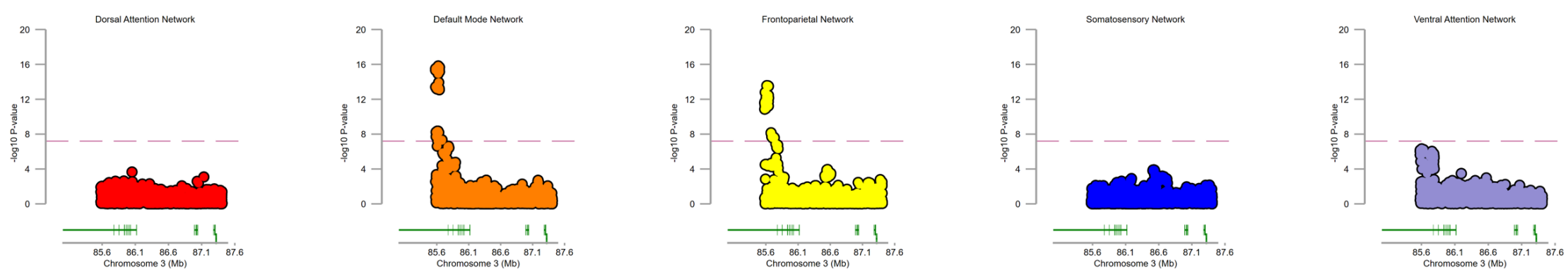

Chromosome 3, position: 88298373 - 94255316

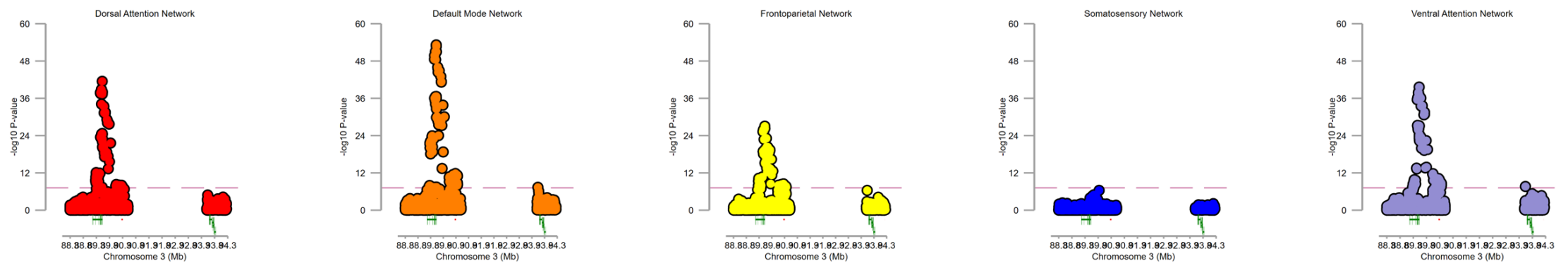

Chromosome 3, position: 146726629 - 149043237

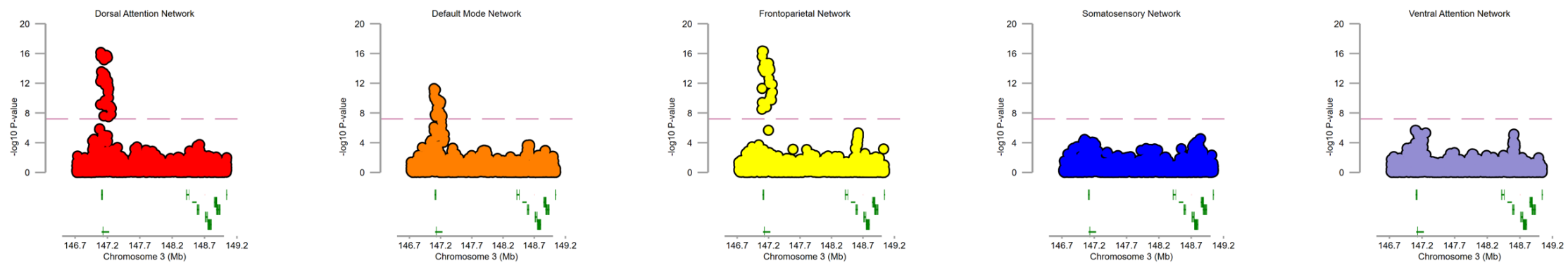

Chromosome 4, position: 27965868 - 29762208

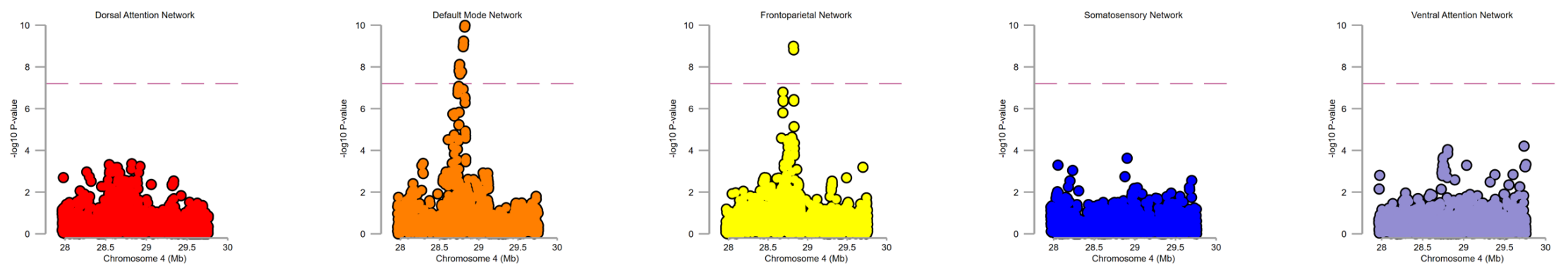

Chromosome 5, position: 73759326 - 75798866

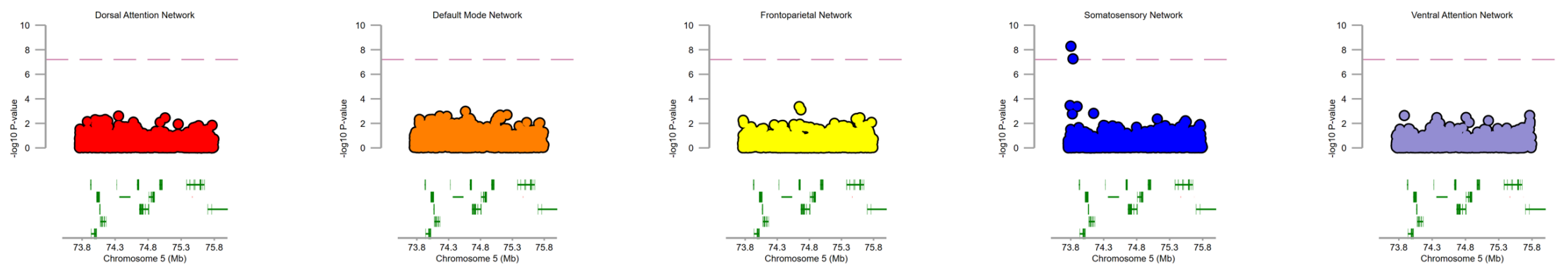

Chromosome 5, position: 87389991 - 88891530

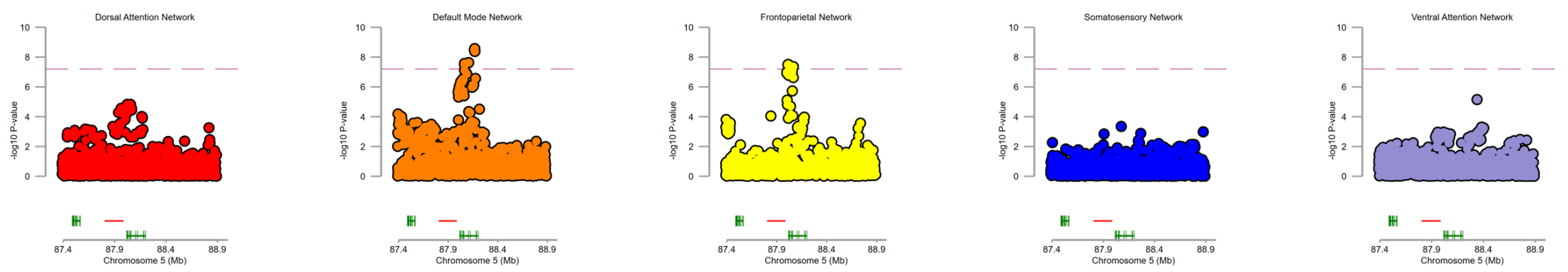

Chromosome 5, position: 91840542 - 93809984

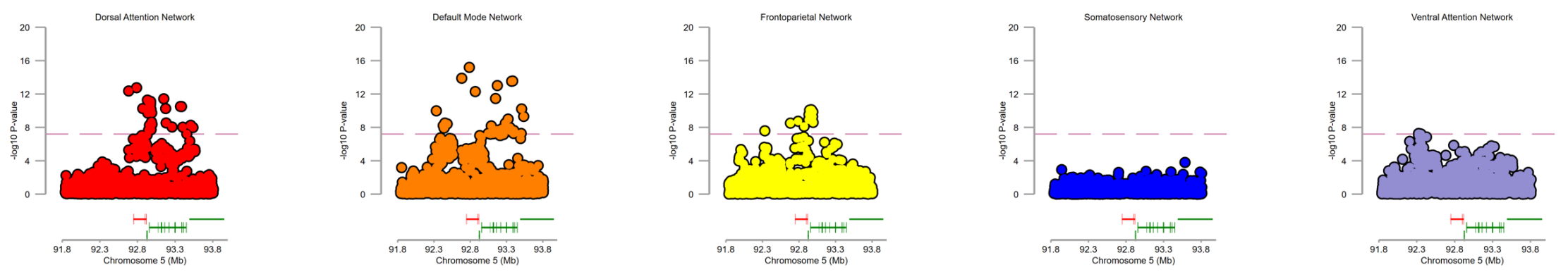

Chromosome 5, position: 93809984 - 95963519

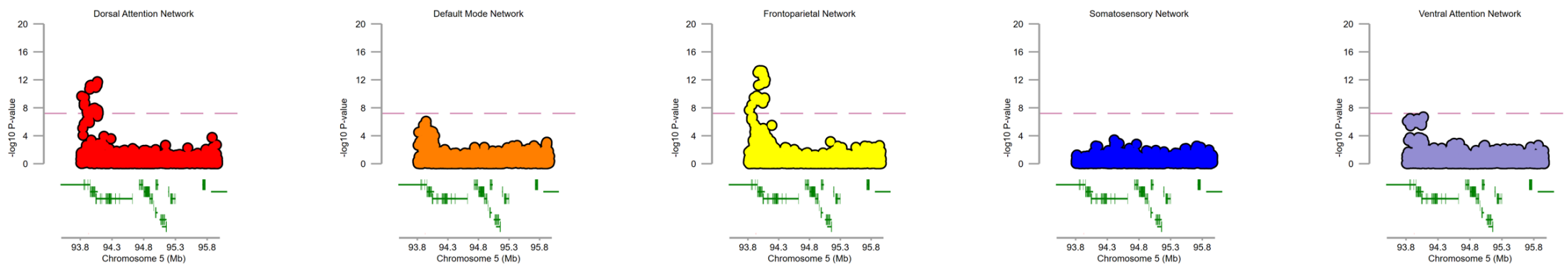

Chromosome 6, position: 25684587 - 26791233

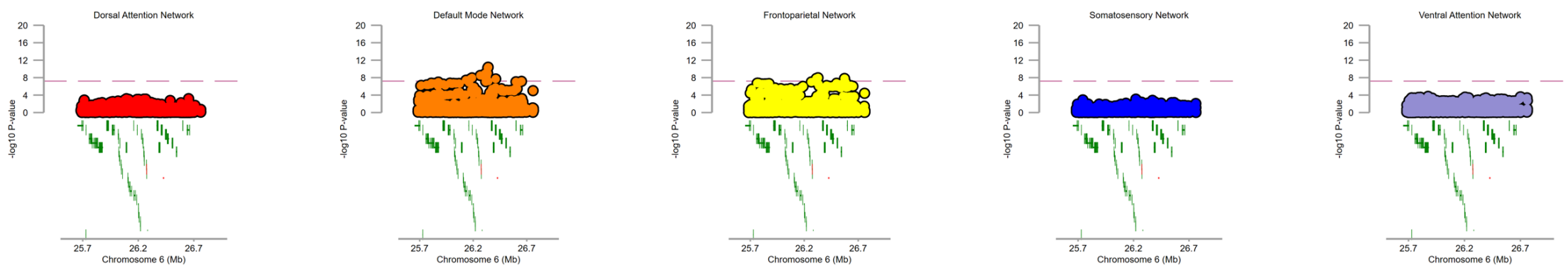

Chromosome 6, position: 26791233 - 28017819

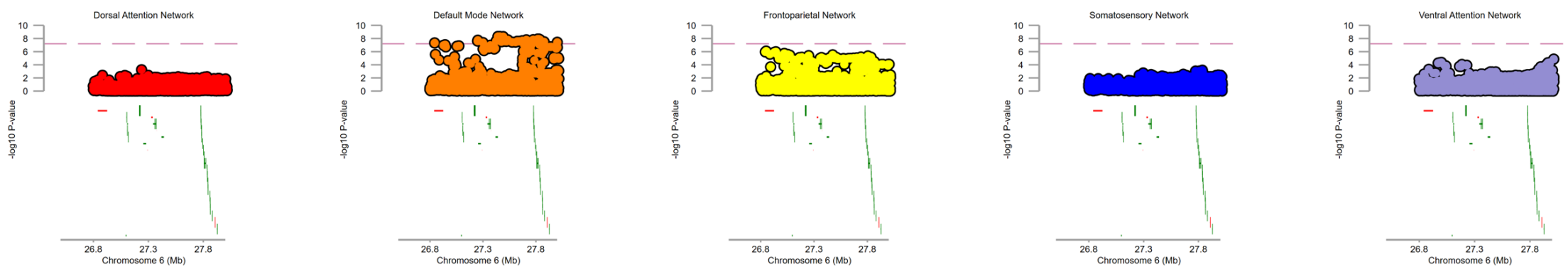

Chromosome 6, position: 29737971 - 30798168

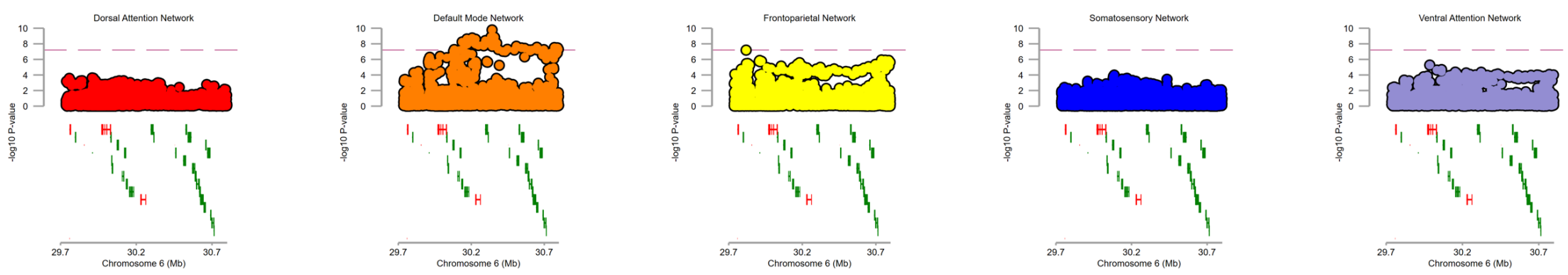

Chromosome 6, position: 30798168 - 31571218

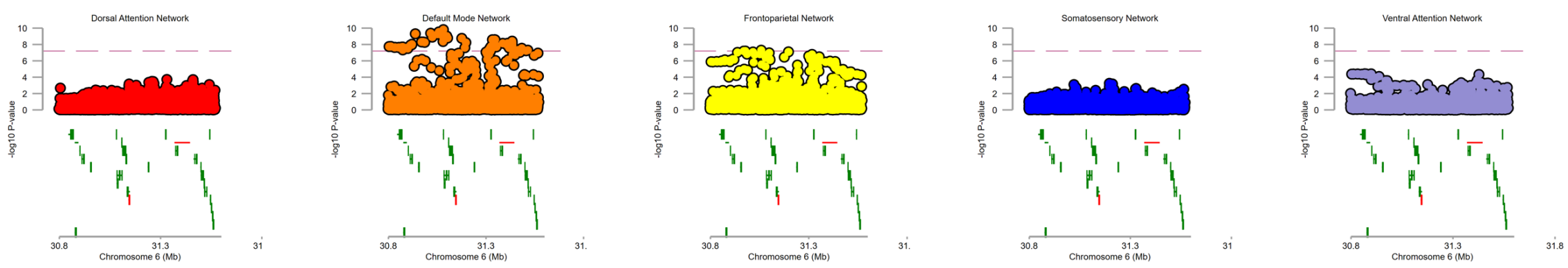

Chromosome 6, position: 94441175 - 97093511

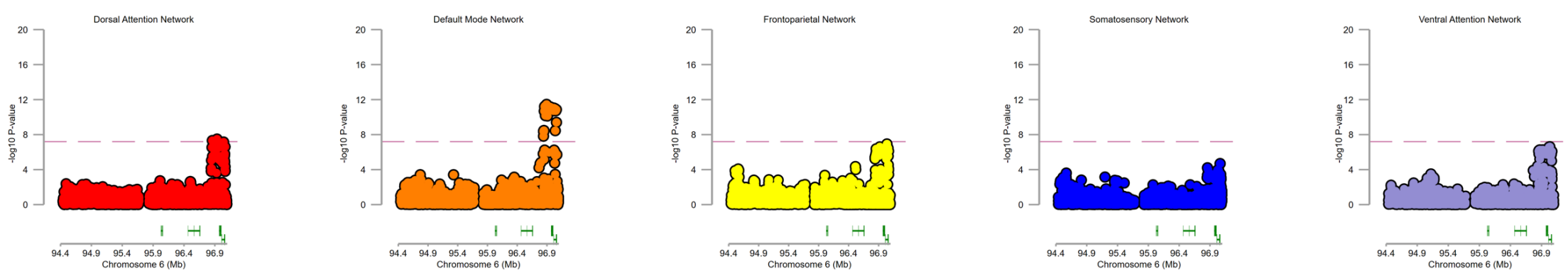

Chromosome 6, position: 97842284 - 100630146

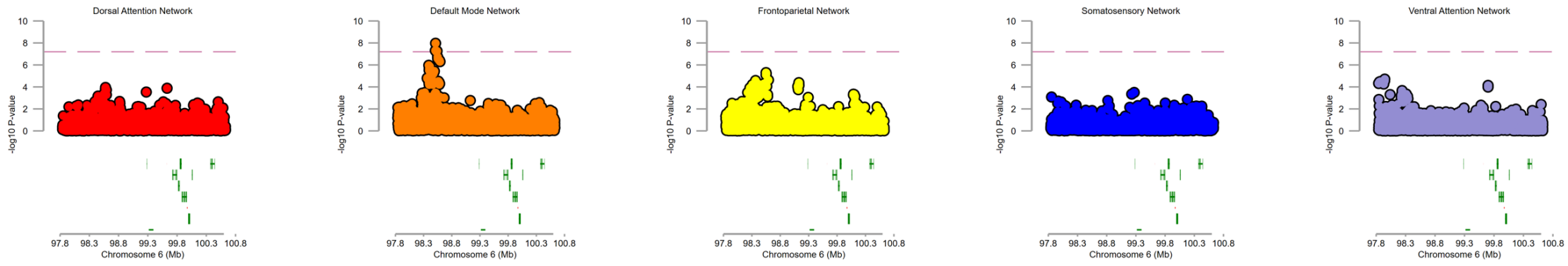

Chromosome 6, position: 125424383 - 127540461

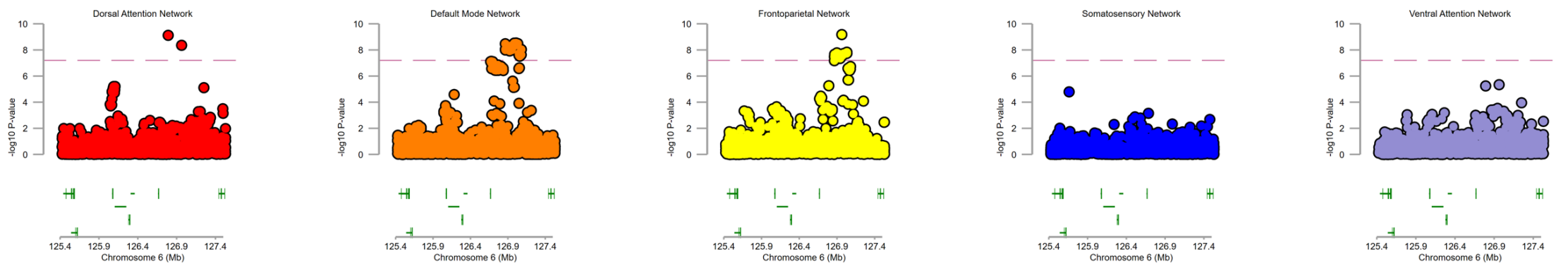

Chromosome 7, position: 113712387 - 116780178

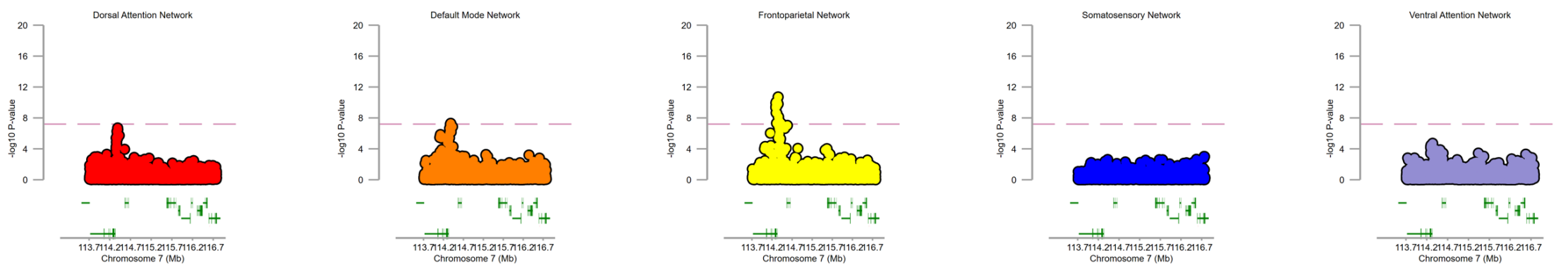

Chromosome 7, position: 130422414 - 132805848

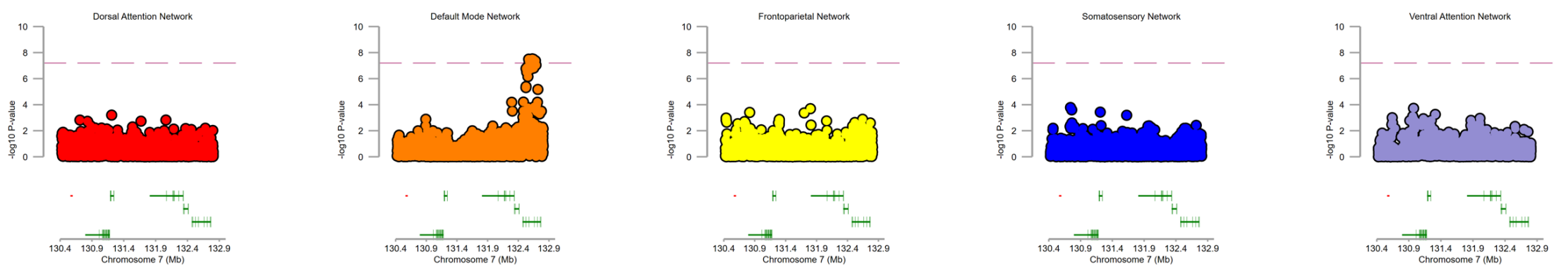

Chromosome 8, position: 7153079 - 9154694

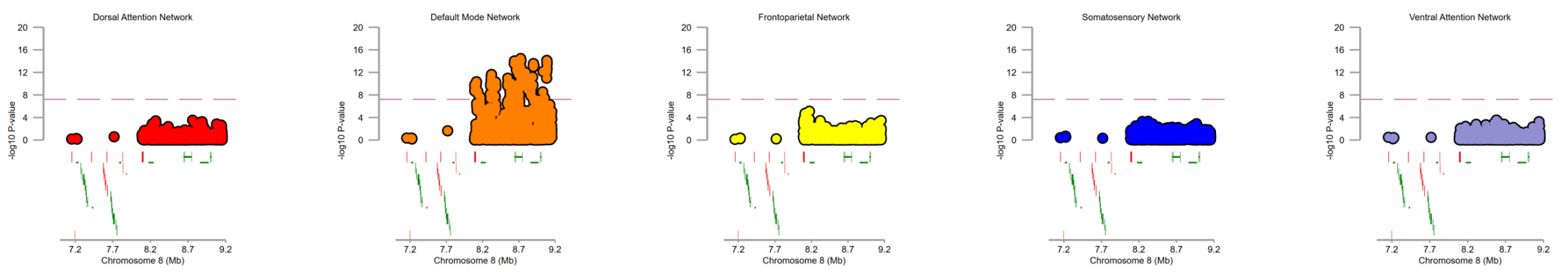

Chromosome 8, position: 9154694 - 9640787

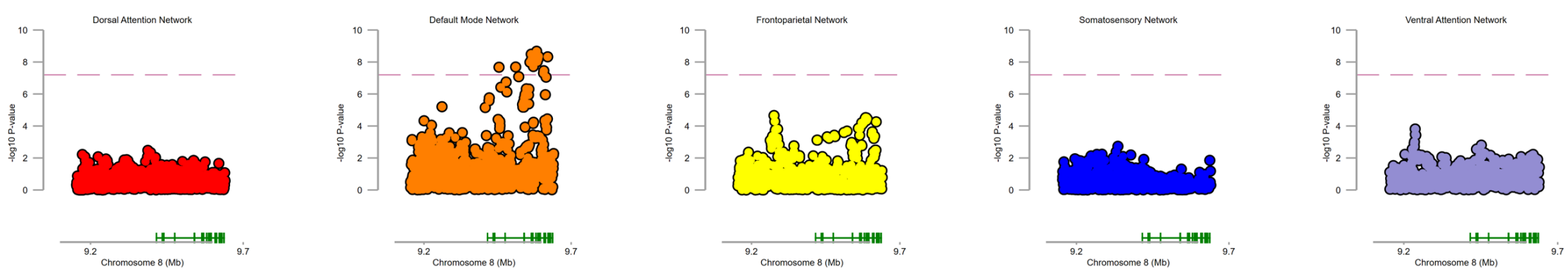

Chromosome 8, position: 9640787 - 10463197

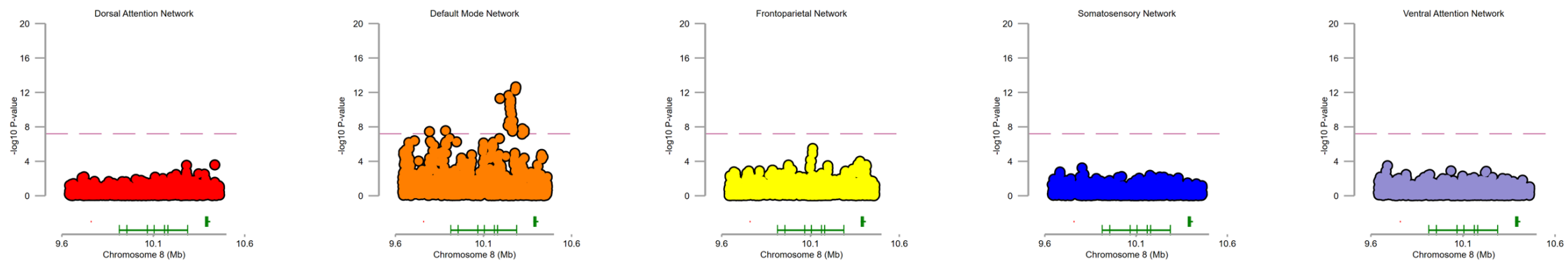

Chromosome 8, position: 10463197 - 11278998

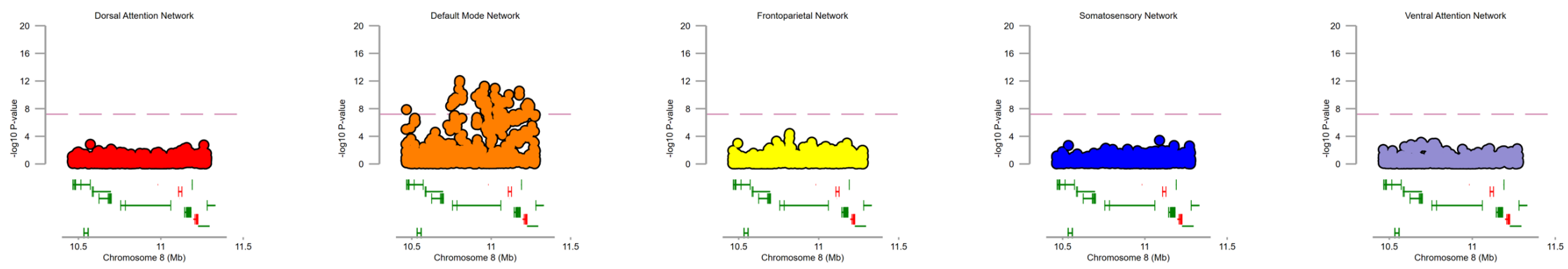

Chromosome 8, position: 11278998 - 13491775

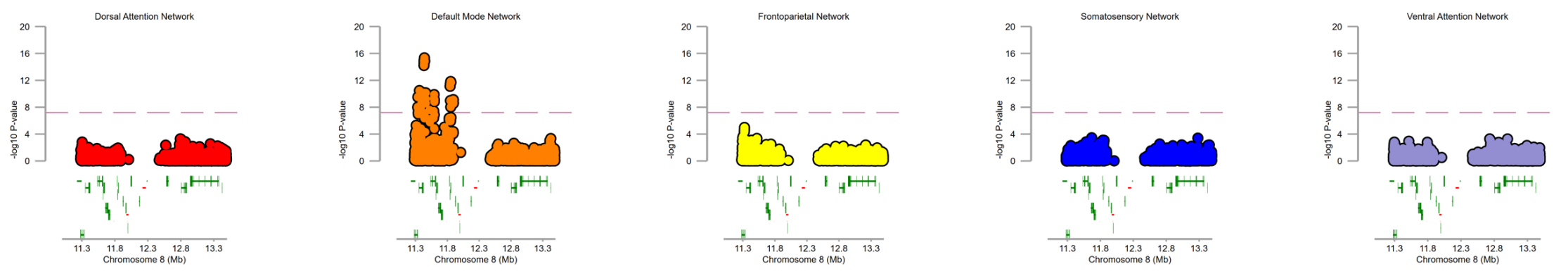

Chromosome 10, position: 19716878 - 22772283

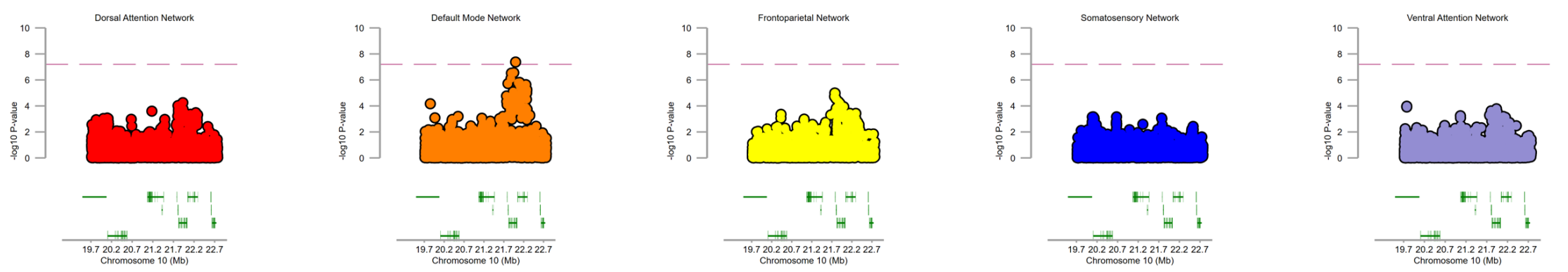

Chromosome 10, position: 95396368 - 96221243

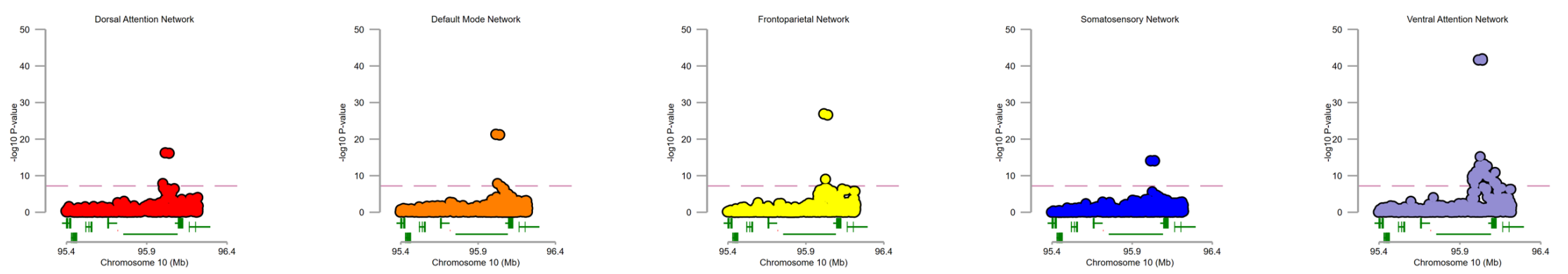

Chromosome 10, position: 116421406 - 119523934

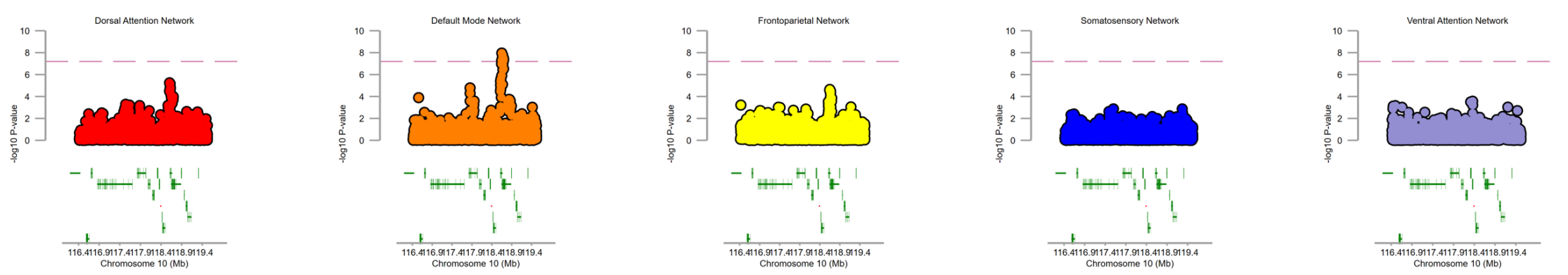

Chromosome 10, position: 125869346 - 128001098

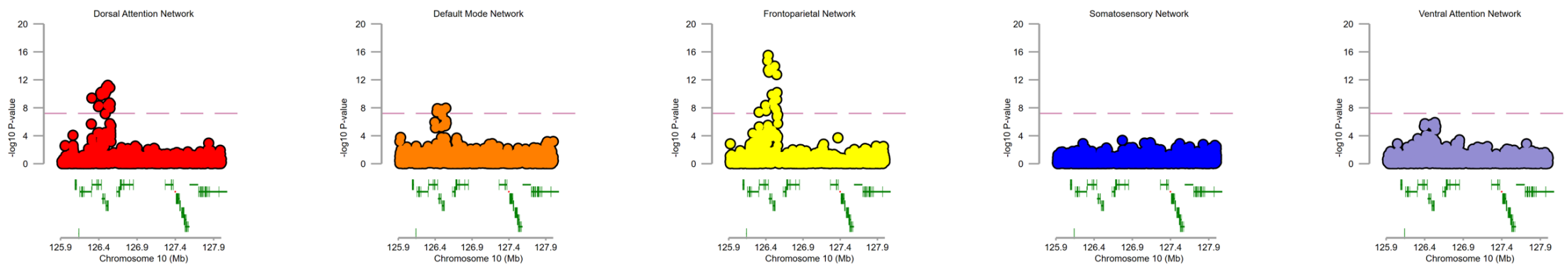

Chromosome 10, position: 132580245 - 134336329

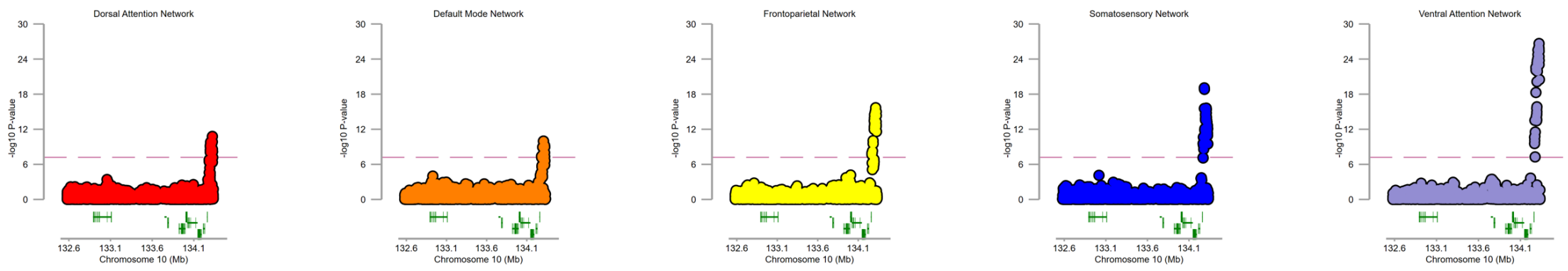

Chromosome 11, position: 69516130 - 70926292

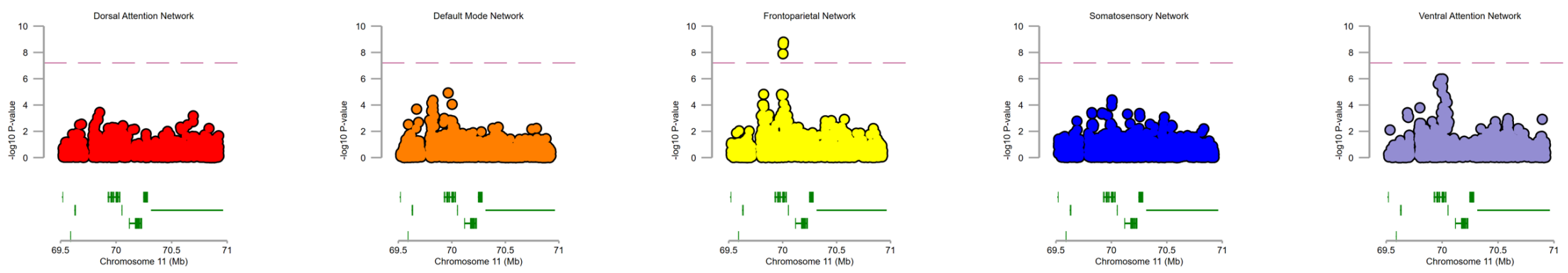

Chromosome 11, position: 101331121 - 103959636

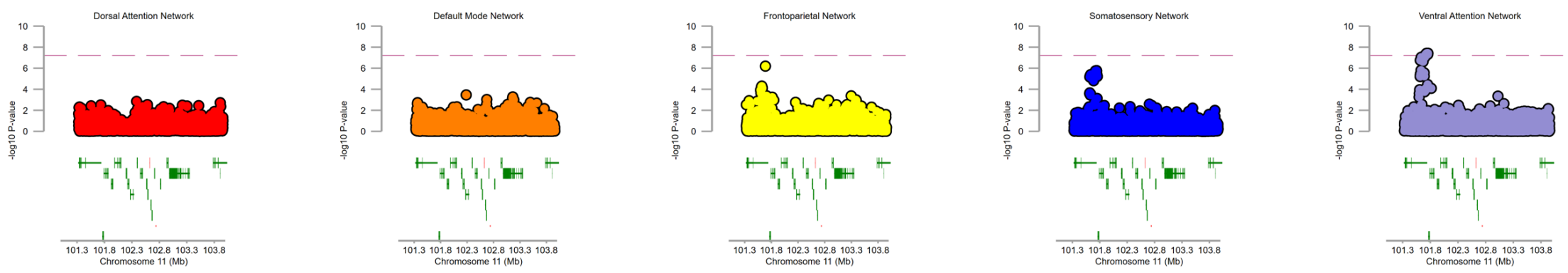

Chromosome 12, position: 5321472 - 6419753

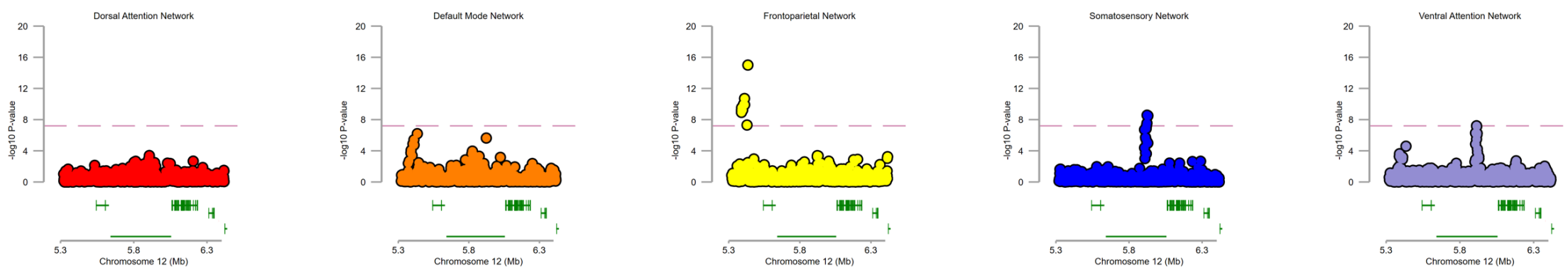

Chromosome 12, position: 65559695 - 67181144

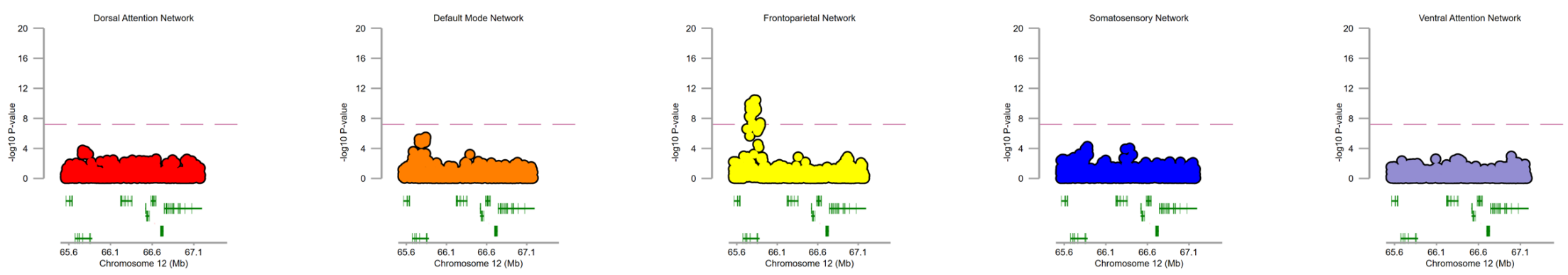

Chromosome 12, position: 78570570 - 80448150

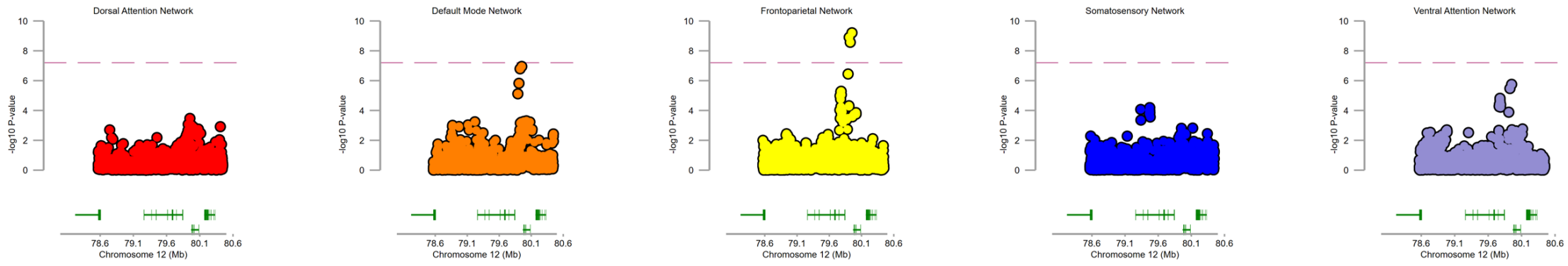

Chromosome 12, position: 89682122 - 92066155

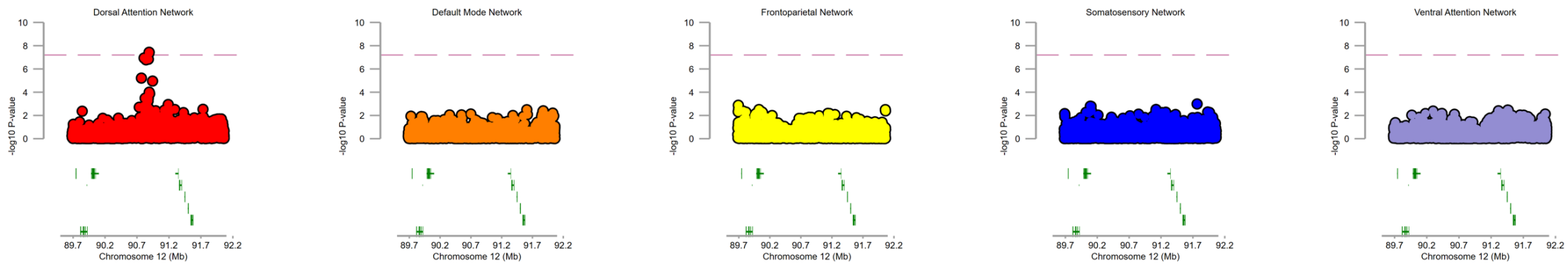

Chromosome 12, position: 106958748 - 109025901

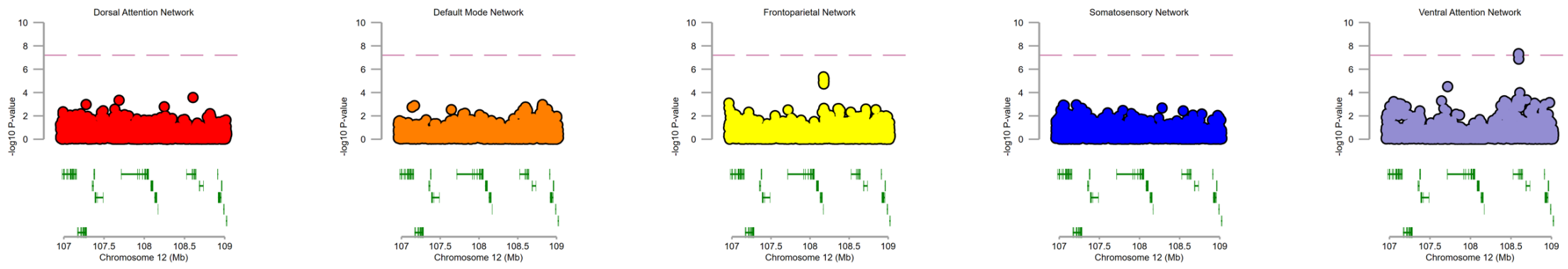

Chromosome 14, position: 57481262 - 59448336

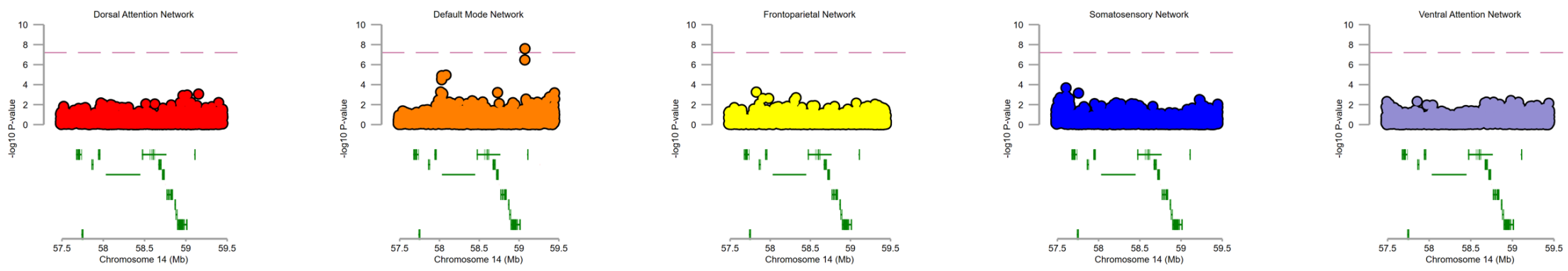

Chromosome 14, position: 59448336 - 61680424

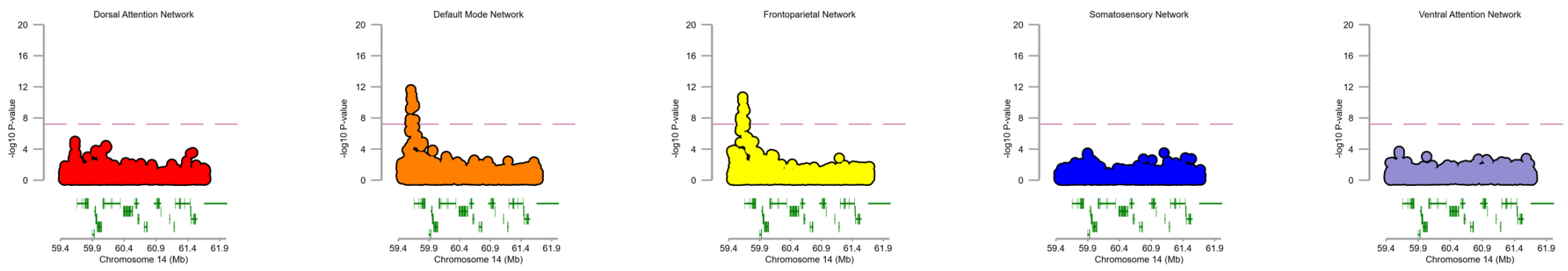

Chromosome 14, position: 63790015 - 65220298

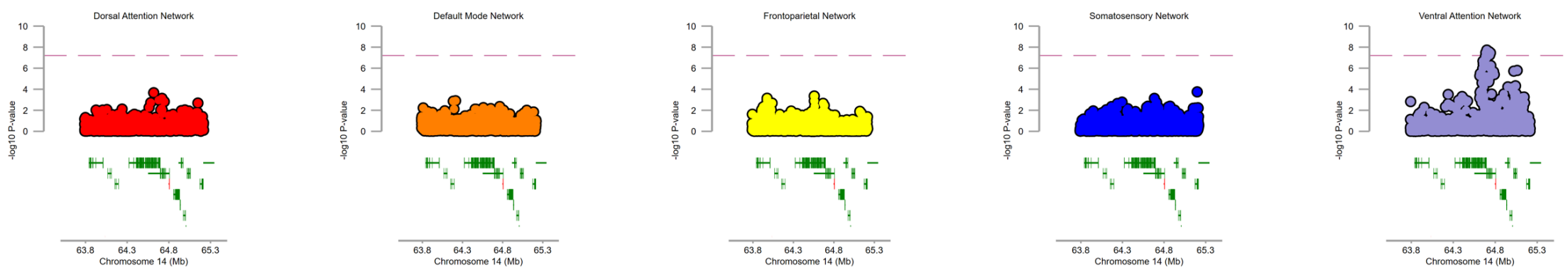

Chromosome 15, position: 38530777 - 40384132

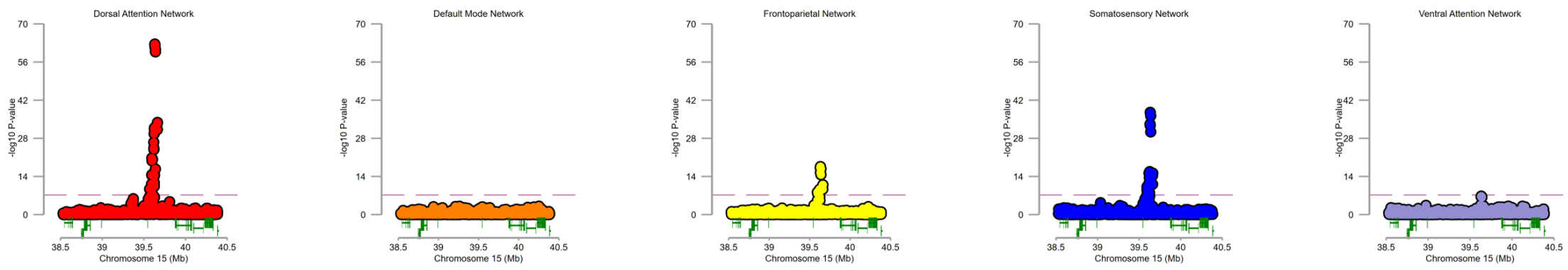

Chromosome 16, position: 86815411 - 87647349

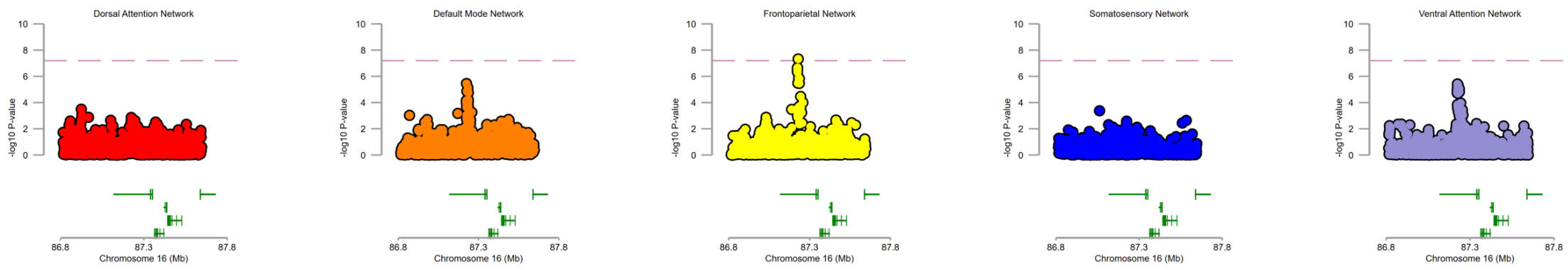

Chromosome 17, position: 18856320 - 21290357

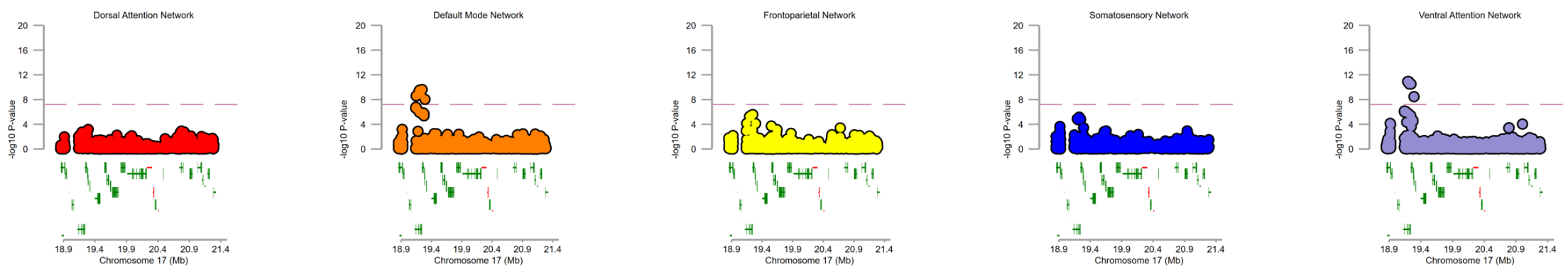

Chromosome 17, position: 27334244 - 29786491

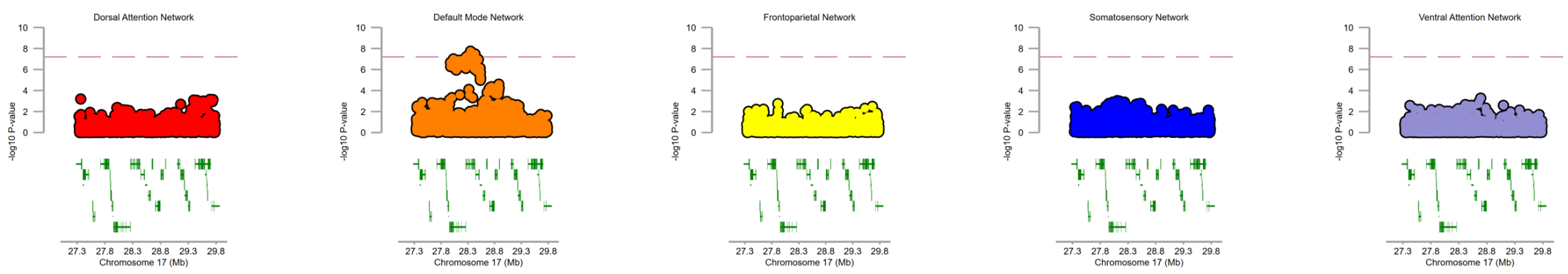

Chromosome 17, position: 43056905 - 45876022

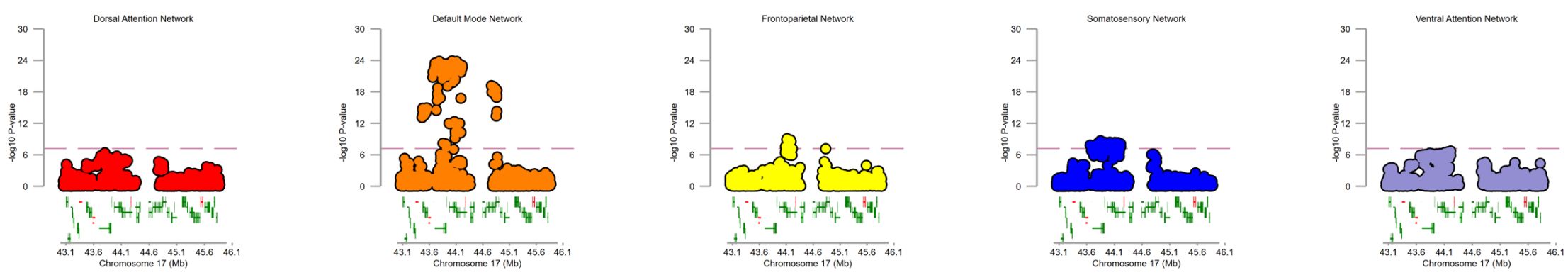

Chromosome 19, position: 30727954 - 32746520

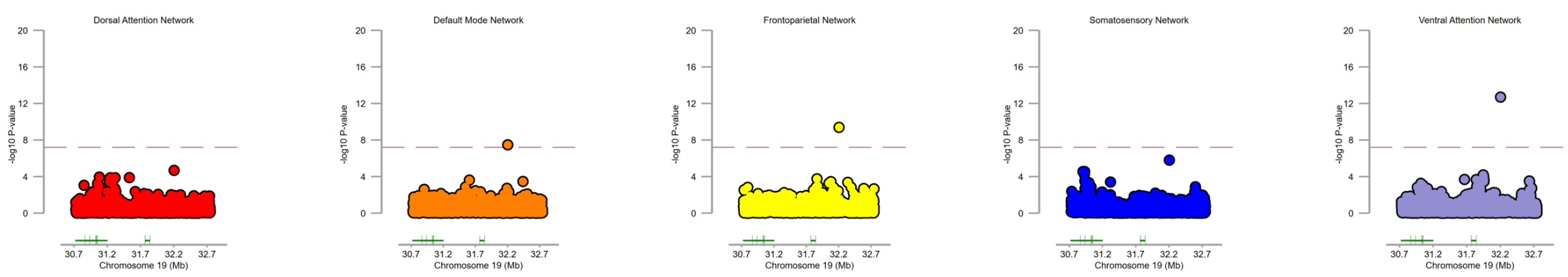

Chromosome 19, position: 44744108 - 46102697

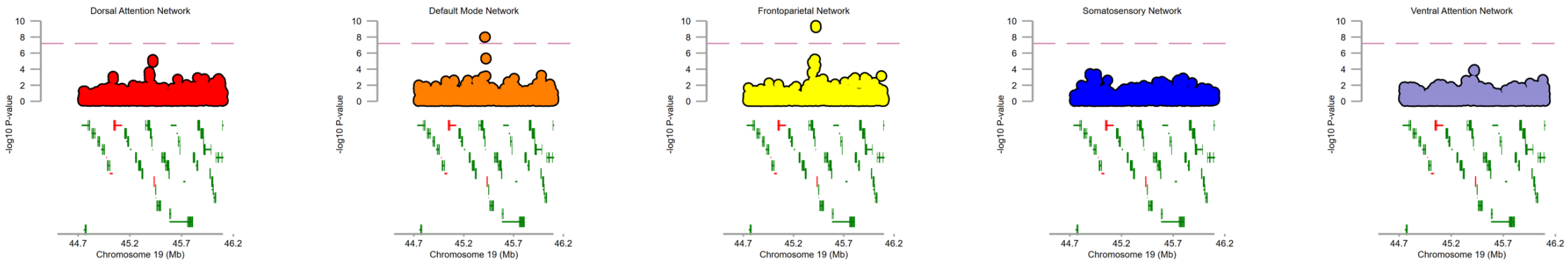

Chromosome 22, position: 46470495 – 47596318

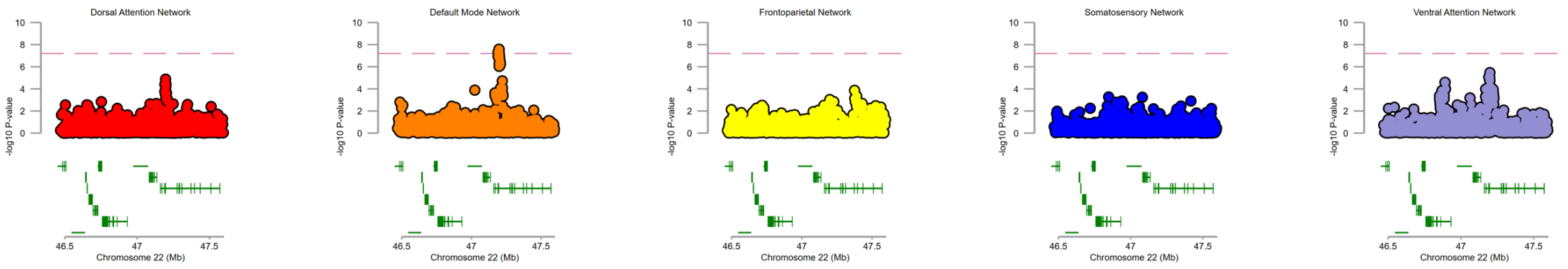

Supplementary Figure 10. Locus-zoom of the 56 LD-regions based on Berisa & Prickell (2016) containing at least one GWAS significant hit across all networks analysed. Red: Dorsal Attention Network; Orange: Default Mode Network; Yellow: Frontoparietal Network; Blue: Somatosensory Network; Purple: Ventral Attention Network. Source data for this figure can be obtained from https://www.ebi.ac.uk/gwas/; hyperlinks to the data for each network are provided in the Data Availability section.

Supplementary tables

Supplementary Table 1: Demographic table of major covariates (n= 30820)

Sex	n (%)	
Female	16,411 (53.2%)	
Male	14,409 (46.8%)	
Imaging Centre		
Cheadle	18,999 (61.6%)	
Reading	3,896 (12.6%)	
Newcastle	7,925 (25.7%)	
	Mean (SD)	Median [min, max]
Age at MRI (yrs)	63.7 (7.50)	64.0 [45.0, 81.0]
rs-fMRI head motion (log)	-2.20 (0.406)	-2.22 [-3.54, -0.189]
MRI head position (X)	0.709 (2.97)	0.590 [-41.1, 17.3]
MRI head position (Y)	65.9 (5.87)	65.0 [52.0, 95.0]
MRI head position (Z)	-27.3 (27.3)	-34.2 [-93.0, 124]
MRI table position (z)	-1060 (25.7)	-1040 [-1200, -1020]
Genetic principal component 1	-0.000903 (0.000660)	-0.00102 [-0.00223, 0.00815]
Genetic principal component 2	0.0000898 (0.000437)	0.000106 [-0.0104, 0.00735]
Genetic principal component 3	0.000262 (0.000861)	0.000242 [-0.0184, 0.00245]
Genetic principal component 4	-0.0000101 (0.000863)	0.0000364 [-0.0146, 0.0207]
Genetic principal component 5	-0.000171 (0.000711)	-0.000174 [-0.00720, 0.0170]
Genetic principal component 6	-0.00000542 (0.00104)	-0.00000281 [-0.0213, 0.0175]
Genetic principal component 7	0.0000980 (0.000650)	0.000102 [-0.0153, 0.00956]
Genetic principal component 8	0.000139 (0.000617)	0.000165 [-0.0127, 0.00796]
Genetic principal component 9	-0.0000485 (0.000483)	-0.0000564 [-0.00708, 0.0135]
Genetic principal component 10	0.0000244 (0.000434)	0.0000445 [-0.0106, 0.00923]

Supplementary Table 2: The number of nodes and edges per resting-state network

rfMRI network	Nodes	Edges
DMN	24	276
VN	17	136
DAN	15	105
SMN	14	91
FPN	13	78
VAN	12	66
LN	5	10

*number of edges corresponds to number of partial correlations in between pairs of nodes included in each MOSTest

Supplementary Table 3: Estimated total number of causal SNPs per trait. Standard error in parenthesis

rfMRI 1	rfMRI 2	Casual SNPs rfMRI 1	Casual SNPs rfMRI 2	Causal SNPs shared
DAN	DMN	0 (0)	60203 (6292)	23776 (5008)
DAN	FPN	12024 (4727)	44 (133)	11751 (799)
DAN	SMN	12640 (7913)	411 (822)	11135 (5216)
DAN	VAN	18817 (5217)	0 (0)	4959 (1098)
DMN	FPN	72182 (5300)	0 (0)	11796 (797)
DMN	SMN	72432 (7905)	0 (0)	11547 (5435)
DMN	VAN	79020 (5398)	0 (0)	4959 (1098)
FPN	SMN	2984 (3910)	2734 (1895)	8813 (3868)
FPN	VAN	6838 (1185)	1 (3)	4958 (1095)
SMN	VAN	7293 (3893)	705 (1664)	4254 (2086)

Supplementary Table 4: Unique and shared estimated causal SNPs between rfMRI networks and neuropsychiatric disorders. Standard error in parenthesis

rfMRI network	neuropsychiatric condition	Unique Causal SNPs rfMRI	Unique Causal SNPs neuropsychiatric	Causal SNPs shared
DMN	SZ	24,179 (2,023)	213 (181)	8,078 (179)
DMN	BD	25,053 (1,958)	268 (279)	7,203 (362)
DMN	MDD	15,347 (2,711)	1,170 (1,681)	16,909 (1,963)
DMN	ASD	25,205 (3,001)	1,169 (1,871)	7,051 (1,955)
DMN	ADHD	25,314 (2,089)	147 (133)	6,942 (239)
DMN	AD	32,242 (2,034)	0 (0)	14 (2)
DAN	SZ	1,426 (1,407)	585 (800)	7,706 (803)
DAN	BD	2,383 (1,663)	721 (709)	6,749 (756)
DAN	MDD	1,423 (1,798)	10,370 (3,121)	7,709 (2,678)
DAN	ASD	3,318 (2,400)	2,406 (2,248)	5,814 (2,209)
DAN	ADHD	2,820 (1,515)	776 (948)	6,312 (922)
DAN	AD	9,118 (1,924)	0 (0)	14 (2)
VAN	SZ	143 (128)	6,530 (428)	1,761 (448)
VAN	BD	159 (221)	5,725 (459)	1,745 (462)
VAN	MDD	255 (299)	16,429 (1,763)	1,650 (424)
VAN	ASD	689 (516)	7,005 (1,091)	1,215 (662)
VAN	ADHD	147 (158)	5,331 (371)	1,757 (316)
VAN	AD	1,890 (421)	0 (0)	14 (2)
FPN	SZ	101 (90)	3,862 (299)	4,430 (307)
FPN	BD	322 (256)	3,262 (484)	4,208 (454)
FPN	MDD	201 (276)	13,750 (1,839)	4,330 (385)
FPN	ASD	342 (590)	4,031 (1,016)	4,189 (740)
FPN	ADHD	86 (81)	2,644 (299)	4,445 (303)
FPN	AD	4,516 (305)	0 (0)	14 (2)
SMN	SZ	823 (1,100)	4,680 (1,801)	3,611 (1,833)
SMN	BD	447 (639)	3,483 (2,026)	3,987 (2,082)
SMN	MDD	901 (1030)	14,545 (2,201)	3,534 (2,050)
SMN	ASD	1,292 (1,344)	5,077 (2,286)	3,143 (2,053)
SMN	ADHD	828 (974)	3,482 (1,974)	3,607 (2,004)
SMN	AD	4,421 (2,086)	1 (2)	13 (3)

References

1. Collins, R. What makes UK Biobank special? *The Lancet* **379**, 1173–1174 (2012).
2. Jenkinson, M. *et al.* Improved Optimisation for the Robust and Accurate Linear Registration and Motion Correction of Brain Images. *NeuroImage* **17**, 825-841 (2002)
3. Beckmann CF & Smith SM. Probabilistic independent component analysis for functional magnetic resonance imaging. I*EEE Trans. Med. Imag*. **23**, 137-152 (2004)
4. Salimi-Khorshidi, G. *et al.* Automatic denoising of functional MRI data: combining independent component analysis and hierarchical fusion of classifiers. *Neuroimage* **90**, 449-468 (2014)
5. Griffanti, L. *et al.* ICA-based artefact removal and accelerated fMRI acquisition for improved resting state network imaging. *Neuroimage* **95**, 232-247 (2014)
6. Andersson JLR *et al.* Non-linear registration, aka spatial normalisation. FMRIB technical report TR07JA2. (2010)
7. Schaefer, A. *et al*. Local-Global Parcellation of the Human Cerebral Cortex from Intrinsic Functional Connectivity MRI. *Cerebral Cortex* **28**, 3095–3114 (2018)
8. Yeo, BTT. et al. The organization of the human cerebral cortex estimated by intrinsic functional connectivity. *Journal of Neurophysiology* **106**, 1125–1165 (2011)
9. McCarthy, S *et al.* A reference panel of 64,976 haplotypes for genotype imputation. *Nature Genetics* **48**, 1279–1283 (2016).
10. Huang, J. *et al.* Improved imputation of low-frequency and rare variants using the UK10K haplotype reference panel. *Nature Communications* **6**, 8111 (2015) https://doi.org/10.1038/ncomms9111
11. Auton, A. *et al.* A global reference for human genetic variation. *Nature* **526**, 68–74 (2015)
12. Frei, O. *et al.* Bivariate causal mixture model quantifies polygenic overlap between complex traits beyond genetic correlation. *Nature Communications* **10**, 2417 (2019)
