# Supplementary material for: Common alleles associated with connectivity within and across the brain’s main functional networks
Source: Nat Commun. 2026 Sep 2;17:10427. doi: 10.1038/s41467-026-77476-2 (PMC13631237; doi:10.1038/s41467-026-77476-2)
Supplement: Supplementary file 4 [file 41467_2026_77476_MOESM2_ESM.pdf]

Description of Additional Supplementary Files

File Name: Supplementary Data 1
Description: Significant loci following MOST GWAS for the Dorsal Attention Network

File Name: Supplementary Data 2
Description: Significant loci following MOST GWAS for the Default Mode Network

File Name: Supplementary Data 3
Description: Significant loci following MOST GWAS for the Fontal Parietal Network

File Name: Supplementary Data 4
Description: Significant loci following MOST GWAS for the Limbic Network

File Name: Supplementary Data 5
Description: Significant loci following MOST GWAS for the Somatosensory Network

File Name: Supplementary Data 6
Description: Significant loci following MOST GWAS for the Ventral Attention Network

File Name: Supplementary Data 7
Description: Significant loci following MOST GWAS for the Visual Network

File Name: Supplementary Data 8
Description: Results from the MOST GWAS for each rfMRI network

File Name: Supplementary Data 9
Description: List of LD-blocks based on Berisa & Prickell (2016) segmentation with at least 1 GWAS significant hit in any network or mental disorder investigated at p<1x10-5

File Name: Supplementary Data 10
Description: MAGMA Gene-Level Multi-Model P-values for the Dorsal Attention Network. Only genes surpassing the genome-wide significant p-value threshold are shown.

File Name: Supplementary Data 11
Description: MAGMA Gene-Level Multi-Model P-values for the Default Mode Network. Only genes surpassing the genome-wide significant p-value threshold are shown.

File Name: Supplementary Data 12
Description: MAGMA Gene-Level Multi-Model P-values for the Frontoparietal network. Only genes surpassing the genome-wide significant p-value threshold are shown.

File Name: Supplementary Data 13
Description: MAGMA Gene-Level Multi-Model P-values for Somatosensory Network. Only genes surpassing the genome-wide significant p-value threshold are shown.

File Name: Supplementary Data 14
Description: MAGMA Gene-Level Multi-Model P-values for the Ventral Attention Network. Only genes surpassing the genome-wide significant p-value threshold are shown.

File Name: Supplementary Data 15
Description: Gene Ontology Enrichment Analyses (for simplicity only gene sets where the FDR corrected p value is smaller or equal 1% are shown)
